# Global Genome and Transcriptome Analyses of *Magnaporthe oryzae* Epidemic Isolate 98-06 Uncover Novel Effectors and Pathogenicity-Related Genes, Revealing Gene Gain and Lose Dynamics in Genome Evolution

**DOI:** 10.1371/journal.ppat.1004801

**Published:** 2015-04-02

**Authors:** Yanhan Dong, Ying Li, Miaomiao Zhao, Maofeng Jing, Xinyu Liu, Muxing Liu, Xianxian Guo, Xing Zhang, Yue Chen, Yongfeng Liu, Yanhong Liu, Wenwu Ye, Haifeng Zhang, Yuanchao Wang, Xiaobo Zheng, Ping Wang, Zhengguang Zhang

**Affiliations:** 1 Department of Plant Pathology, College of Plant Protection, Nanjing Agricultural University, Nanjing, China; 2 Key Laboratory of Integrated Management of Crop Diseases and Pests, Ministry of Education, Nanjing, China; 3 BGI-Shenzhen, Beishan Industrial Zone, Yantian District, Shenzhen, China; 4 Department of Pediatrics, Louisiana State University Health Sciences Center, New Orleans, Louisiana, United States of America; Texas A&M University, UNITED STATES

## Abstract

Genome dynamics of pathogenic organisms are driven by pathogen and host co-evolution, in which pathogen genomes are shaped to overcome stresses imposed by hosts with various genetic backgrounds through generation of a variety of isolates. This same principle applies to the rice blast pathogen *Magnaporthe oryzae* and the rice host; however, genetic variations among different isolates of *M*. *oryzae* remain largely unknown, particularly at genome and transcriptome levels. Here, we applied genomic and transcriptomic analytical tools to investigate *M*. *oryzae* isolate 98-06 that is the most aggressive in infection of susceptible rice cultivars. A unique 1.4 Mb of genomic sequences was found in isolate 98-06 in comparison to reference strain 70-15. Genome-wide expression profiling revealed the presence of two critical expression patterns of *M*. *oryzae* based on 64 known pathogenicity-related (PaR) genes. In addition, 134 candidate effectors with various segregation patterns were identified. Five tested proteins could suppress BAX-mediated programmed cell death in *Nicotiana benthamiana* leaves. Characterization of isolate-specific effector candidates Iug6 and Iug9 and PaR candidate Iug18 revealed that they have a role in fungal propagation and pathogenicity. Moreover, Iug6 and Iug9 are located exclusively in the biotrophic interfacial complex (BIC) and their overexpression leads to suppression of defense-related gene expression in rice, suggesting that they might participate in biotrophy by inhibiting the SA and ET pathways within the host. Thus, our studies identify novel effector and PaR proteins involved in pathogenicity of the highly aggressive *M*. *oryzae* field isolate 98-06, and reveal molecular and genomic dynamics in the evolution of *M*. *oryzae* and rice host interactions.

## Author Summary

Genetic variations in pathogens, such as the causal agent of rice blast *Magnaporthe oryzae*, often lead to circumvention of disease-resistance cultivars. Previous genome-wide analyses of model organisms suggest that pathogen effectors are also rapidly evolving, especially in regions with high genome plasticity. However, genetic variations among different isolates remain largely unknown in *M*. *oryzae*, particularly at the genome and transcriptome levels. In this study, we provided a systematic genomic and interaction transcriptome profile for a dominant rice blast field isolate, resulting in identification of 134 candidate effectors. Two effectors, Iug6 and Iug9, and one pathogenicity-related (PaR) gene product, Iug18, were subjected to functional characterization. We found that Iug6 and Iug9 are located in the biotrophic interfacial complex (BIC) and their overexpression leads to suppression of defense-related gene expression in rice, while Iug18 appears to be a novel PaR protein. Our studies support the hypothesis that isolate-unique genes may serve as a source of genetic variability in the *M*. *oryzae* population encountering different environments. Our studies also facilitate further understanding of effectors and genomic variations in pathogenicity of *M*. *oryzae*.

## Introduction

Rice (*Oryza sativa*) is one of the most important staple food crops for more than half of the global human population [1]. However, the rice production is severely impacted by the blast disease caused by the fungus *Magnaporthe oryzae*, despite the cultivation of various disease-resistance cultivars [2,3,4]. One of the reasons is that new cultivars often lose their resistance within a few years of introduction. It is thought that genetic variability occurs rapidly within pathogen populations, especially in regions with high genome plasticity, and that the pathogenic traits such as secreted pathogen avirulence (AVR)/effectors evolve rapidly to counteract plant defense [5,6,7,8].

*M*. *oryzae* primarily infects rice but can also infects wheat or other small grains [9]. Recent advances in genetic and genomic technology have allowed *M*. *oryzae* to be a tractable model for studying the plant-microbe interaction [10]. Jantasuriyarat and colleagues used large-scale expressed sequence tag (EST) sequencing to profile gene expression at the early stage of the *M*. *oryzae*-rice interaction and identified four genes to be involved in the interaction out of 13,570 uniESTs [11]. Approximately 100 pathogenesis-related protein genes specific to infection were identified in other studies using EST analysis [10], and four biotrophy-associated secreted (BAS) proteins were also found in *M*. *oryzae* using microarray [4]. More recently, several studies have identified the presence of an infection structure, known as the biotrophic interfacial complex (BIC), that is involved in mediating the delivery of pathogen effectors into the rice host cytoplasm [4,12].

Interestingly, studies have also revealed that genome plasticity is closely linked to host-pathogen interaction specificities. Using genome-wide DNA polymorphism existed between *M*. *oryzae* field isolate Ina168 and isolate 70–15, one of the progenies from a cross between a weeping lovegrass isolate and the archetypical rice isolate Guy11 [13,14,15], three novel *AVR* genes were identified [16]. The presence of these AVRs correlates to a 1.68 Mb sequence in Ina168 that is absent from the assembled genome sequence of 70–15 [16]. Studies of two other field isolates, P131 and Y34, also revealed slightly larger genome contents with more genes. It is suggested that the presence of these isolate-specific genes play diverse roles, including conidiation, vegetative growth, or virulence [17].

Genome studies of additional *M*. *oryzae* field isolates and identification of novel *AVR* effectors will help us to further address the mechanism of pathogen and host coevolution. Here, we characterized the genome of the dominant blast isolate 98–06 on several different rice cultivars cultivated in Southeast China (S1 Table). We found that 98–06 contains seven known *AVR*s, corresponding to its wide field adaptability. *AVR PWL1*, *PWL2*, *Avr-Pita*, *Avr1-CO39*, and *ACE1* are highly polymorphic due to point mutations while *AVR Avr-Pia* and *Avr-Pii* are also polymorphic, but with complete deletions. *AvrPiz-t* and *Avr-Pik* allele (D) were also found and conserved. In addition, we found that isolate 98–06 contains 1.43 Mb of isolate-specific sequences encoding 238 isolate-specific genes, in comparison to isolate 70–15. In addition, genome-wide expression profiling revealed the presence of a defense network in rice and major expression patterns of pathogenesis-related genes during the *M*. *oryzae*-rice interaction. Moreover, we predicted 134 candidate effectors from 1,732 putative secreted proteins and provided evidence to demonstrate that *IUG6* and *IUG9* are novel effectors.

## Results

### Sequencing and assembling of the isolate 98–06 genome

The genome of the *M*. *oryzae* isolate 98–06 was sequenced using Illumina high-throughput sequencing technology. Four different insert-size libraries (500, 350, 5,000, and 10,000 bp) were generated, which represent 36.8-, 38.2-, 31.9-, and 27.6-fold coverage assemblies spanning 1,560, 1,618, 1,350, and 1,167 Mb, respectively. The sequence coverage that was approximately six-fold greater than that of P131 and Y34 [17], provided a near perfect assembly. The sequence reads were assembled into 1,161 contigs and placed into 284 scaffolds with a combined length of 42.1 Mb using SOAPdenovo [18,19]. The N50 and maximum lengths of scaffolds were 1,530 and 3,511 kb, respectively (Table 1). The sequences have been deposited at GenBank under the submission number of JRBC00000000.

**Table 1:** Genome assembly and annotation statistics from six *Magnaporthe oryzae* isolates.

		98–06	P131	Y34	FJ	HN	70–15
**Genome**							
	Estimated genome size (Mb)	45.3	-	-	-	-	-
	Coverage (fold)	135^a^	20	21	34	6	-
	Number of scaffolds	284	1,823	1,198	-	-	-
	N50 scaffold length (kb)	1530.0	12.3	11.6	-	-	
	Total scaffold length (Mb)	42.1	37.9	38.9	37.3	37.1	41.7
	Number of contigs	1,161	2,601	2,114	6,290	6,249	-
	N50 contig length (kb)	88.6	37.7	53.0	151.7	147.4	-
	GC content (%)	50.8	51.5	51.3	51.3	51.5	51.6
	Repeat (%)	9.3	10.3	10.8	-	-	10.6
**Genes**							
	Number of genes	14,019^b^	12,722	12,869	10,453	10,256	12,440
	Average gene length (bp)	1,410	1,334	1,332	2,085	1,745	1,356

a: Four different insert size (500 bp, 350 bp, 5,000 bp, and 10,000 bp) libraries were constructed to acquire enhanced coverage.

b: Multiple software were performed to predict genes of 98–06, as distinct from only FGENESH software used for P131 and Y34. The softwares used in this study were listed in materials and methods.

**Fig 1:**
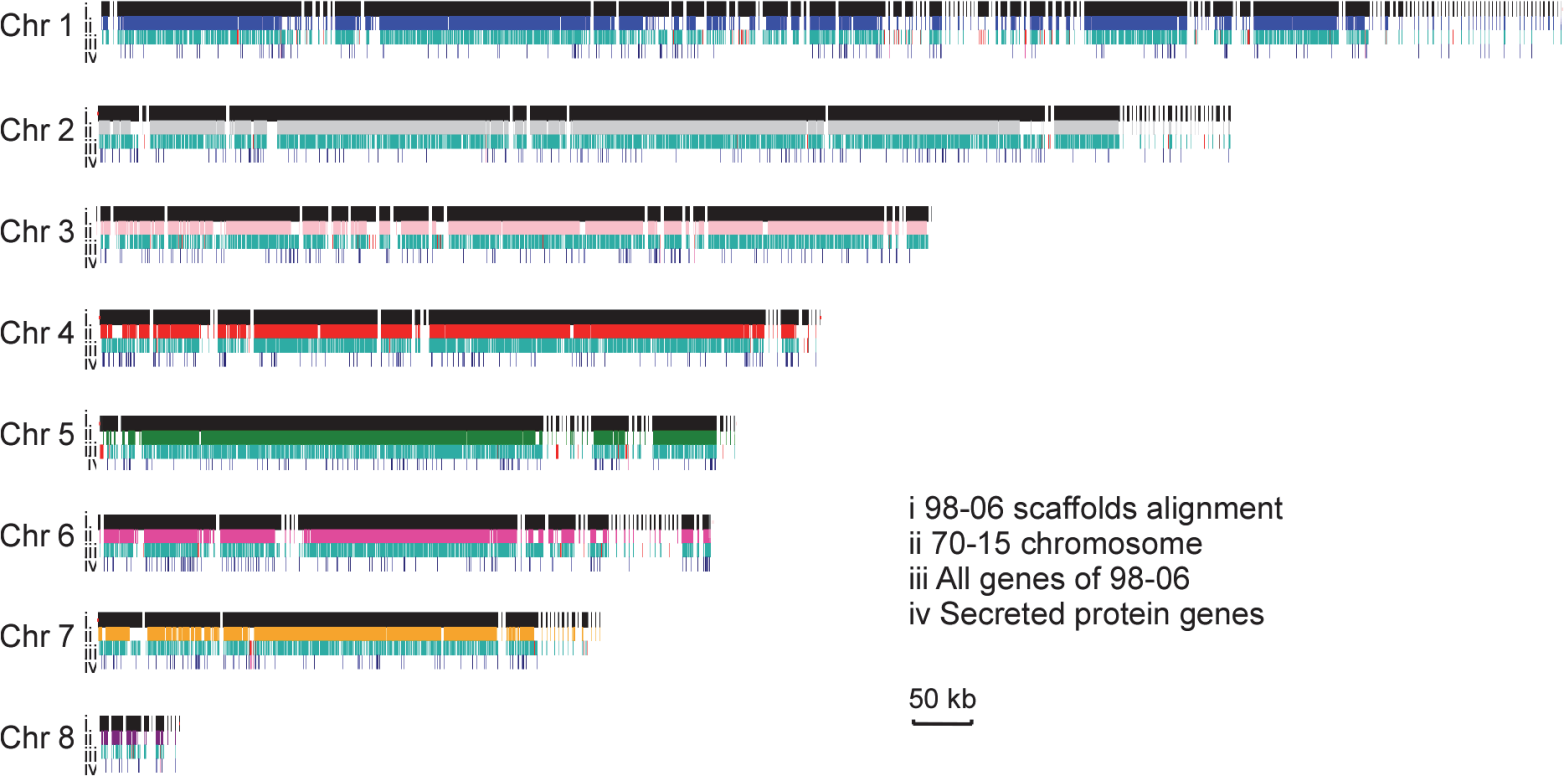
Global view of synteny alignments between isolates 98–06 and 70–15. Scaffolds, genes, and secreted protein genes distribution in isolate 98–06 compared to 70–15. For each chromosome, the first line (i) represents the genomic scaffolds of 98–06 alignment with 70–15; the second line (ii) displays the chromosmes of 70–15; the third line (iii) and fourth line (iv) show all genes and secreted protein genes, respectively. The red small vertical lines in line (iii) and line (iv) show the isolate-specific genes in 98–06 compared to 70–15. Chr, chromosome.

When the scaffolds of isolate 98–06 were aligned with the assembled genome of isolate 70–15, 98.83% of the genome was conserved. However, 98–06 contains 1.4 Mb isolate-specific sequences that are dispersed throughout the genome (Fig 1, S2 Table). Interestingly, blocks of the reverse-alignment sequences and chromosomal breakpoints were found at regions near the telomeres (S1 Fig).

### Genome annotation

A total of 14,019 genes including 1,732 secreted protein genes were identified from the annotated isolate 98–06 genome (Table 1). The average length of predicted proteins is ~470 amino acids, and the predicted genes comprise 46% of the assembly genome. In all, the 98–06 genome possesses more genes than isolates 70–15 [15], P131, Y34 [17], FJ, or HN [20]. Further analysis revealed that 238 genes are unique when compared with 70–15 (49 genes if compared with P131 and Y34), and of these 238 genes, 10% encode secreted proteins while 69% have no significant homologs in GenBank (S3 Table). We conducted a Clusters of Orthologous Groups (COG) study and found that some of these specific genes are associated with general biological functions, including secondary metabolism and energy generation processes (S2 Fig). Intriguingly, isolate-specific genes appear to be mainly located at some of the chromosomal ends.

To identify possible gene families, we clustered the predicted proteins of isolates 98–06, 70–15, P131, and Y34 using the OrthoMCL algorithm. A total of 52,177 proteins were grouped into 13,236 clusters, with each cluster containing at least two putative protein orthologs/paralogs (S3 Fig, S4 Table). The mean gene number of each cluster for 98–06, 70–15, P131, and Y34 was 1.11, 1.04, 1.02, and 1.02, respectively. Among these clusters, 12.86% of all 14,019 genes in 98–06 had at least one paralog, while 6.28%, 3.27%, and 3.08% of all genes for 70–15, P131, and Y34 respectively (S4 Table). These results suggested that there are more paralogs in 98–06 than in the other isolates. Meanwhile, there are 455, 310, 165, and 150 gene families with at least two paralogs in 98–06, 70–15, P131, and Y34, respectively. Comparative analysis indicated that 227 gene families are expanded in 98–06 (S4 Table), which is consistent with its larger genome content.

### Repeated sequences and transposable elements

Repeated sequences and transposable elements (TEs) often account for a large portion of genomes and therefore are important in genome annotation. Using multiple approaches, we found that 9.3% of the 98–06 genome consists of repetitive sequences. This is consistent with other fungi in which repetitive sequences comprise ≤ 10% proportion of the genomes [21]. However, transposable elements predicted by RepeatMasker [22] are more abundant in isolate 98–06 (2,776) than in isolates 70–15 (2,297), P131 (2,055), or Y34 (2,322) (S5 Table). In a follow-up examination of the larger number of genes (up to 1,732) that encode putative secreted proteins in 98–06, 132 genes containing putative signal peptide sequences were disrupted by TEs (S6 Table).

### Identification of effector candidates

Proteins secreted by fungal pathogens during host colonization are generally referred to as effectors [23,24], which are often less than 200 amino acids in length and cysteine-rich. Therefore, we principally focused on small secreted proteins less than 200 amino acids in length, and identified 645 effector candidates out of 1,732 secreted proteins in 98–06 (S7 Table). These candidates, including known *AVR* genes *Avr-Pik* (allele D) and *AvrPiz-t*, have an average cysteine content of 3.11% in comparison to 1.26% of all 1,732 putative secreted proteins. Isolate 98–06 is known to exhibit an incompatible interaction with various rice cultivars harboring 12 known resistance (R-) genes: *Pi7*, *Pi1*, *Pik*, *Pik-m*, *Pi20*, *Pi9*, *Pita2*, *Piz-t*, *Pik-p*, *Pish*, *Piz5*, and *Pik-h* (S1 Table), and the *Pi7*, *Pi1*, *Pik*, *Pik-m*, *Pi-kp*, and *Pik-h* are alleles that could all recognize *Avr-Pik* (allele D). We focused on the remaining 643 (645–2 = 643) secreted proteins that provide a rich repertoire for the identification of novel effectors.

### Interactive transcriptome analyses

Despite that conserved motifs such as the RxLR domain of oomycetes [25] were not found in some *M*. *oryzae* effectors, the *M*. *oryzae* effectors are often planta-specific and secreted proteins [4]. To study the host-pathogen interaction, we performed RNA sequencing (RNA-Seq) for six stages: mycelium (MY) and conidial infection at 0 h, 8 h, 24 h, 48 h, and 72 h post inoculation (CO-0h to CO-72h). After discarding low-quality raw reads, we obtained 12.8~13.7 million clean reads from each of the six samples, and aligned these reads against the reference genes and genomes (S8 Table). Almost all of the genes in isolate 98–06 and rice (*O*. *sativa*) were transcribed (11,075 and 26,452, respectively, S9 Table). The majority of the differentially expressed genes (log_2_Ratio ≥1 and GFOLD (0.01) >1) in *M*. *oryzae* were up-regulated during host-pathogen interaction in comparison to conidial infection at 0 h, suggesting a strong interaction between *M*. *oryzae* and rice (S4 Fig).

To confirm the RNA-Seq profiles, qRT-PCR was conducted on nine randomly selected *M*. *oryzae* and rice genes that were either induced or repressed. The average match between RNA-Seq and qRT-PCR data of the original sequenced samples was 30 of 41 (73%) (S5 Fig), indicating basic consistency between the two approaches. In addition, the average match between qRT-PCR of another independent sampling and the original sampling, or the RNA-Seq, was 76% and 63%, respectively (S5 Fig). There also appear certain correlations among the sequencing data of samples belonging to different stages, as distinct from the comparative RNA-Seq between wild type and mutant. Moreover, the expression patterns of several previously characterized genes, such as *MoACTIN*; *MoAP1*, *MoVAM7*, and *MoBAS1* [4,26,27], in the transcriptome (see S10 Table) were similar to previous studies. These validation tests rectified to certain degree the potential limitation resulting from lack of sample duplication in RNA-Seq analyses.

**Fig 2:**
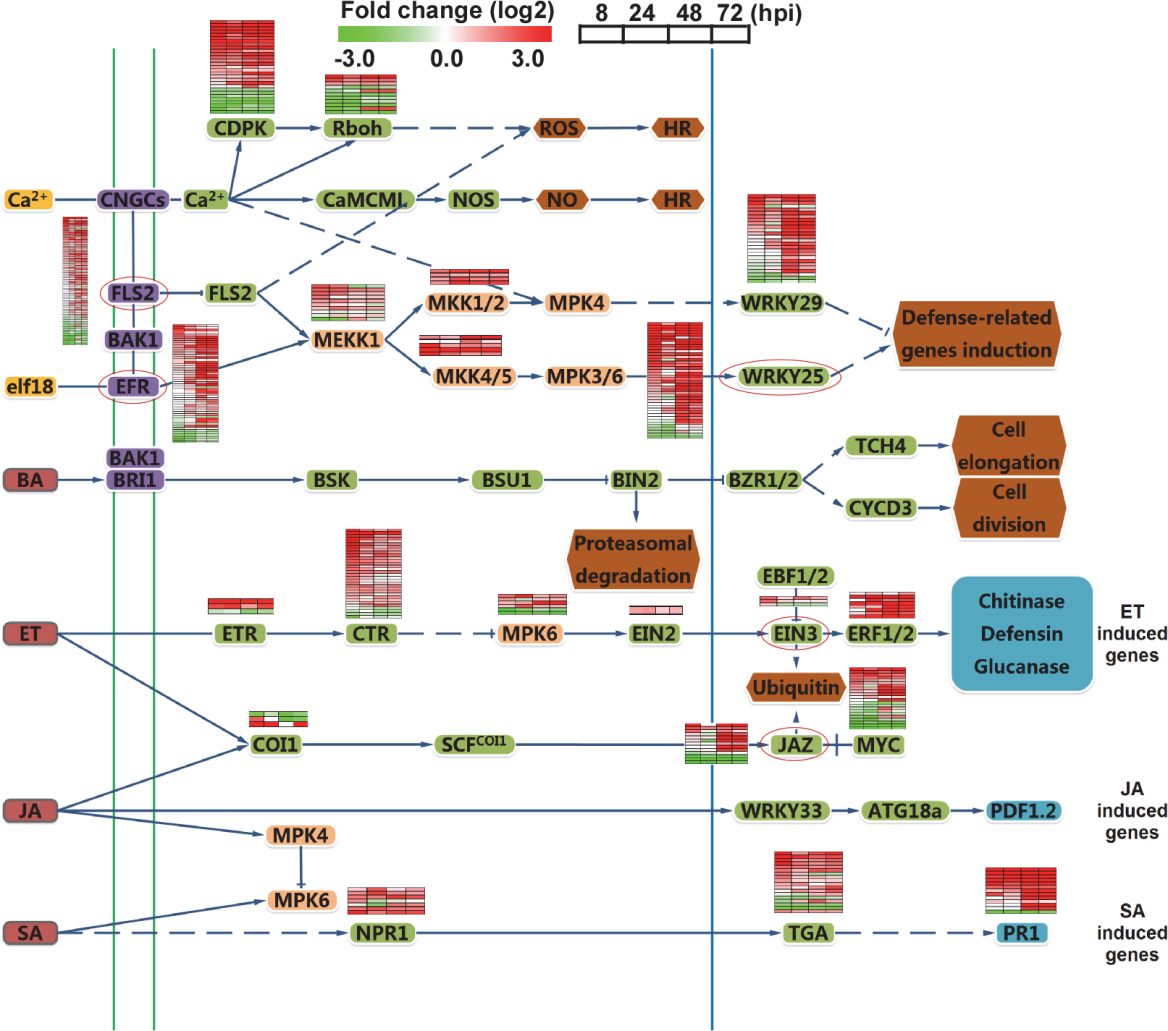
Analysis of different signal pathways gene expression in rice. Heat maps in boxes indicate the expression of individual genes, and the chart plots indicate the aggregate expression levels of the pathway genes. Green box, transcription factors. Red circle, genes for the nearest heat map.

Since plant hormone responses play an important role in the host defense network during the rice-pathogen interaction [28], we examined the expression of genes involved in salicylic acid (SA), jasmonic acid (JA), ethylene (ET), and mitogen-activated protein kinase (MAPK) signaling cascades. Most of the genes in the SA and ET signaling pathways were up-regulated, meanwhile, JAZ genes that act as transcriptional repressors of JA [29] were induced after 48 hpi. A comparison of aggregate expression levels at 0 hpi versus 8, 24, 48 and 72 hpi in planta is shown in Fig 2. Transcription for the most of the MAPK genes was increased during infection. In addition, several WRKY transcription factors and pattern recognition receptors (PRRs) FLS2 [30] and EFR were also significantly induced at 48 hpi (Fig 2). These findings revealed that multiple host defense signaling pathways were involved in the response to the infection of isolate 98–06.

**Fig 3:**
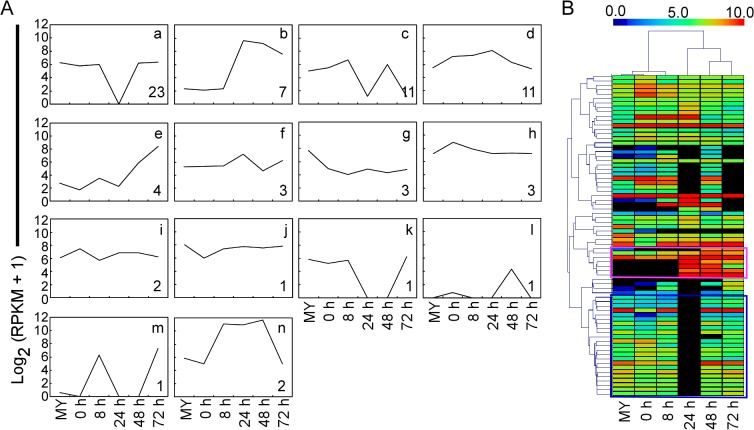
Clustering analysis of gene expression patterns of pathogenicity-related genes and effectors through RNA-Seq. **(A)** CAST assay shows 14 *M*. *oryzae* expression clusters of 64 reported pathogenicity genes and ten effectors of *M*. *oryzae* at different stages. The y-axis stands for the log2 average gene expression levels. The quantity of cluster member is marked at the right bottom of each pattern line. **(B)** Heat map showing expression levels of known pathogenicity genes and effectors of *M*. *oryzae* during compatible interactions. The color bar represents the log2 of (RPKM +1) value, ranging from blue (0.0) to red (10.0). Top, stage tree; left, gene tree. The pink rectangle indicates expression pattern of effectors. The blue rectangle indicates expression pattern relevant to pathogenicity.

Finally, to investigate how the *M*. *oryzae* genetic program is deployed during infection, we explored 64 known genes relevant to pathogenicity and 10 known effectors of *M*. *oryzae* in our interaction transcriptome (S10 Table), and determined their expression patterns by the clustering affinity search technique (CAST) assay through MultiExperiment Viewer 4.6 software package [31]. The distance metric is the default Pearson correlation, and the threshold affinity value is 0.8. Fourteen clusters were generated, and the cluster-a with the most members (23 genes) was illustrated, which provided the major expression pattern of pathogenesis-related genes (Fig 3A, a; S10 Table). The expression pattern-a represents high expression throughout the infection process, with the exception of sharp reduction at 24 hpi. In general, the virulence factors were up-regulated with different waves of expression during infection (Fig 3A). Interestingly, another expression pattern-b for effectors was also distinguished (Fig 3A and 3B; S10 Table).

### Expressions of SNARE and effector genes

In transcriptome analysis described above, two genes *MoVAM7* and *MoSSO1* encoding soluble N-ethylmaleimide-sensitive factor attachment protein receptor (SNARE) were also identified. SNARE proteins mediate intracellular transport that is an essential biological process in fungi [32]. Consistent with this, MoVam7 and MoSso1 were found to be important in effector secretion and pathogenesis [27,33]. We gathered 21 SNARE genes and 35 other endocytosis-related (ER) genes through bioinformatics analysis, and found five expression patterns of SNARE genes and eight patterns of ER genes using the CAST method (S6 Fig; S11 and S12 Tables). It is surprising that the key expression patterns of SNARE and ER genes are similar to the pathogenicity pattern-a, suggesting that this typical pathogen–host interaction pattern could be used to explore novel pathogenicity genes.

In a broader definition, effectors are specific types of proteins secreted into the plant by pathogens to interfere with plant defenses, and they most likely play other roles in promoting infection as well [34]. To date, 15 effectors were identified that include nine Avr effectors, six newly identified effectors including four BAS proteins, Slp1, and MC69. In this analysis, *AvrPiz-t*, *BAS1*, *BAS3*, *BAS4*, and *SLP1* genes were clustered into expression pattern-b (Fig 3A), indicating that the expression is highly inducible during the biotrophic invasion. Hierarchical clustering (HCL) analysis also showed two distinct expression patterns that are similar to pattern-a and pattern-b, respectively (Fig 3B).

**Fig 4:**
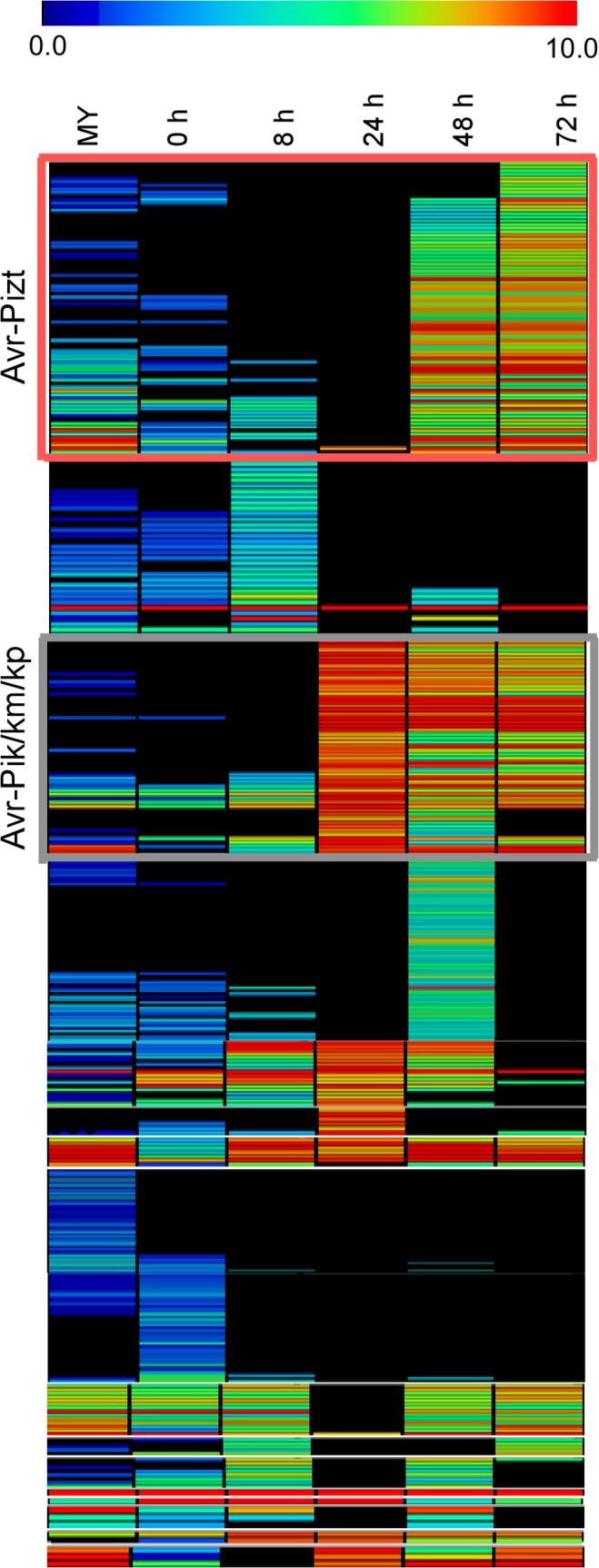
Heat map of 645 small candidate effectors of 98–06. 645 candidate effectors were added to employ the hierarchical clustering, and divided into two groups (red rectangle and gray rectangle) according to the expression pattern of *Avr-Pik* and *AvrPiz-t*, providing 134 candidate effectors. The color bar represents the log2 of (RPKM +1) value, ranging from blue (0.0) to red (10.0).

Since *Avr-Pik* and *AvrPiz-t* genes are highly expressed during infection, and studies of two *M*. *oryzae* isolate-specific sequences and *Verticillium dahliae* indicated that lineage-specific genomic regions are enriched in genes encoding new effectors [35], we explored the expression patterns of 645 small candidate effectors in 98–06 through CAST assay. 134 candidate effectors were found to be co-regulated with *Avr-Pik* and *AvrPiz-t* (Fig 4; S13 Table). Six known effectors *BAS1*, *BAS2*, *BAS3*, *BAS4*, *SLP1*, and *PWL1* were among these candidates, providing a compelling argument for the presence of additional effectors among these candidates.

### Five candidate effectors suppress the hypersensitive cell death induced by BAX

Many bacterial and fungal pathogen effectors can suppress innate plant immunity, particularly that triggered by pathogen-associated molecular patterns [34,36,37]. For example, *M*. *oryzae* AvrPiz-t suppresses the BAX-mediated programmed cell death in tobacco leaves [38]. Among 134 putative secreted proteins identified, most have no predicted functions, including several 98–06 isolate-unique genes (IUG) that were not found in 70–15. We queried the nucleic acid sequences against the genomic sequences of P131 and Y34 and found that *IUG6* (Mo_GLEAN_10000617) and *IUG9* (Mo_GLEAN_10000765) are specific to 98–06.

**Fig 5:**
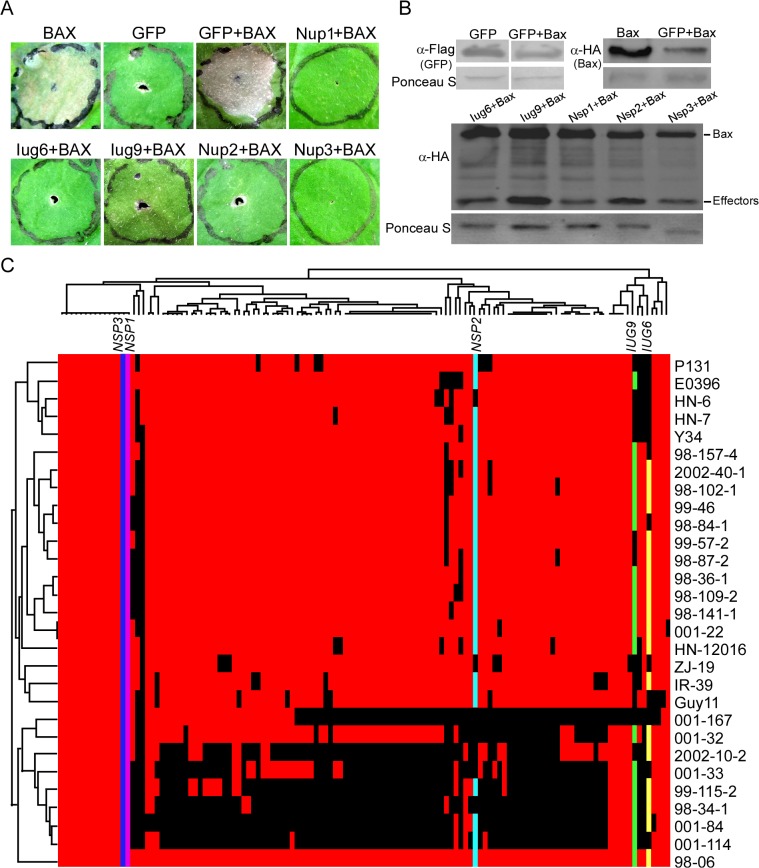
Iug6, Iug9, Nup1, Nup2, and Nup3 suppress the cell death triggered by BAX. **(A)** Agroinfiltration sites in *N*. *benthamiana* leaves expressing Iug6, Iug9, Nup1, Nup2, or Nup3 were challenged with *A*. *tumefaciens* expressing the BAX elicitin. The BAX-induced cell death was scored at 3 and 4 DAI. *A*. *tumefaciens* strain carrying pGR106-GFP was used as a negative control, and pGR106-BAX as a positive control. **(B)** Western blot analysis of GFP, Iug6, Iug9, Nup1, Nup2, Nup3, and Bax protein levels in plant tissues treated above. Proteins were extracted 60 h after the last infiltration. Equal amounts of protein lysate were loaded in each lane, as verified by Ponceau S staining. **(C)** Presence or absence polymorphisms of each candidate effectors are indicated by a colored or black tile across the 29 field isolates. Colored tile, presence; black tile, absence. Presence /absence patterns (top) and isolates (left) were hierarchically clustered.

To identify Iug6 and Iug9 functions, we used a PVX-based high-throughput transient plant expression system in *Nicotiana benthamiana*. We also included three randomly selected non-specific proteins Nup1, Nup2, and Nup3 (MGG_07900, MGG_08024, and MGG_04546) for controls. We first removed the signal peptides of *IUG6*, *IUG9*, *NUP1*, *NUP2*, and *NUP3* to enable the genes to be expressed stably in plant cells before cloning into the PVX vector pGR106. Infiltration of *N*. *benthamiana* leaves with *Agrobacterium tumefaciens* cells carrying pGR106:*IUG6*, pGR106:*IUG9*, pGR106:*NUP1*, pGR106:*NUP2*, pGR106:*NUP3*, and the negative control pGR106:*GFP* did not cause any obvious cell-death symptoms (S7 Fig), whereas obvious cell death was observed in *N*. *benthamiana* leaves infiltrated with *A*. *tumefaciens* cells carrying pGR106:BAX. *N*. *benthamiana* leaves infiltrated with *A*. *tumefaciens* cells harboring *IUG6*, *IUG9*, *NUP1*, *NUP2*, and *NUP3* genes 24 h prior to infiltration with the pGR106:BAX-harboring cells did not produce symptoms (Fig 5A). The expression of BAX was detected at 48 h after infiltration (Fig 5B), which ruled out the possibility that BAX failed to express. Iug6, Iug9, Nup1, Nup2, and Nup3 all conferred BAX cell-death suppression activity. By analogy, additional effectors may also present in these 134 candidate effectors.

Presence or absence polymorphisms of these 127 candidate effectors (minus six known effectors and one failed to be amplified by PCR) were tested by PCR using 29 *M*. *oryzae* field isolates collected from China (Fig 5C). The candidate effectors showed different segregation patterns. Among them, *IUG6*, *IUG9*, and *NUP2* have varied presence in 29 field stains, whereas *NUP1* and *NUP3* are detected in all isolates (Fig 5C). A recent report indicates that the presence or absence of putative secreted proteins shows a good correlation with *AVR* genes in *M*. *oryzae* [16]. Since half of the characterized blast *AVR* effector genes are not present in the isolate 70–15 genome, it may suggest that gene gain or loss events could be a major factor in the evolution of AVR effectors [39].

### Functional analysis of isolate-unique genes (IUGs)

Functional characterization of IUGs may reveal novel effectors or other factors significant in the host-pathogen interaction. We thus further characterized the functions of *IUG6* and *IUG9*, along with *IUG17*, *IUG18*, *IUG34*, and *IUG37*. We found that *IUG6* and *IUG9* are located near the chromosomal ends (S14 Table), which is consistent with the hypothetic rapid development of novel effectors in plastic regions [40]. *IUG17* (Mo_GLEAN_10000632) and *IUG18* (Mo_GLEAN_10001404) are predicted to each contain the aminoglycoside phosphotransferase (APH) domain (145–223 amino acids) and the RelA_SpoT domain (96–234 amino acids), while *IUG34* (Mo_GLEAN_10000877) and *IUG37* (Mo_GLEAN_10002374) are predicted to be associated with pathogenicity-related expression pattern through CAST assays. We also generated respective deletion mutants in 98–06 using the hygromycin-resistance marker gene and complemented the mutants with the respective wild type gene alleles including the endogenous promoter of approximately 1-kb (S8 Fig).

Disruption of *IUG6* and *IUG9*, as well as *IUG18*, resulted in defects in vegetative hyphal growth and virulence, whereas disruption of *IUG17*, *IUG34*, and *IUG37* had no obvious effects on colony morphology, conidiation, or virulence (S9 Fig). Iug6 is relatively specific to *M*. *oryzae*, since only one homolog was identified in the fungus *Gaeumannomyces graminis* (GL385397.1, 53% identity, E = 4e-17). Iug9 homologs can be found in *Colletotrichum higginsianum* (CCF43986.1, 63% identity, E = 7e-29) and *C*. *graminicola* (EFQ33033.1, 63% identity, E = 4e-26). In contrast, Iug18 contain the RelA_SpoT domain that is well conserved among other filamentous fungi. Microsynteny analysis revealed that all three genes are located at the genomic inserted regions comparable to 70–15 (S10A Fig). However, the parallel result was unfavorable, probably because the laboratory strain 70–15 was generated from a cross between rice and weeping lovegrass isolates [13,14]. To exclude the possibility that this was an assembly gap leading to gene deletion in the other three isolates, we amplified the genes by PCR from isolates 70–15, Guy11, P131, and Y34 and performed a Southern blotting analysis (S10B Fig). Expression of these three genes was validated by qRT-PCR, which also showed that *IUG6* and *IUG9* exhibit high expression levels during the pathogen-host interaction (S11 Fig).

**Fig 6:**
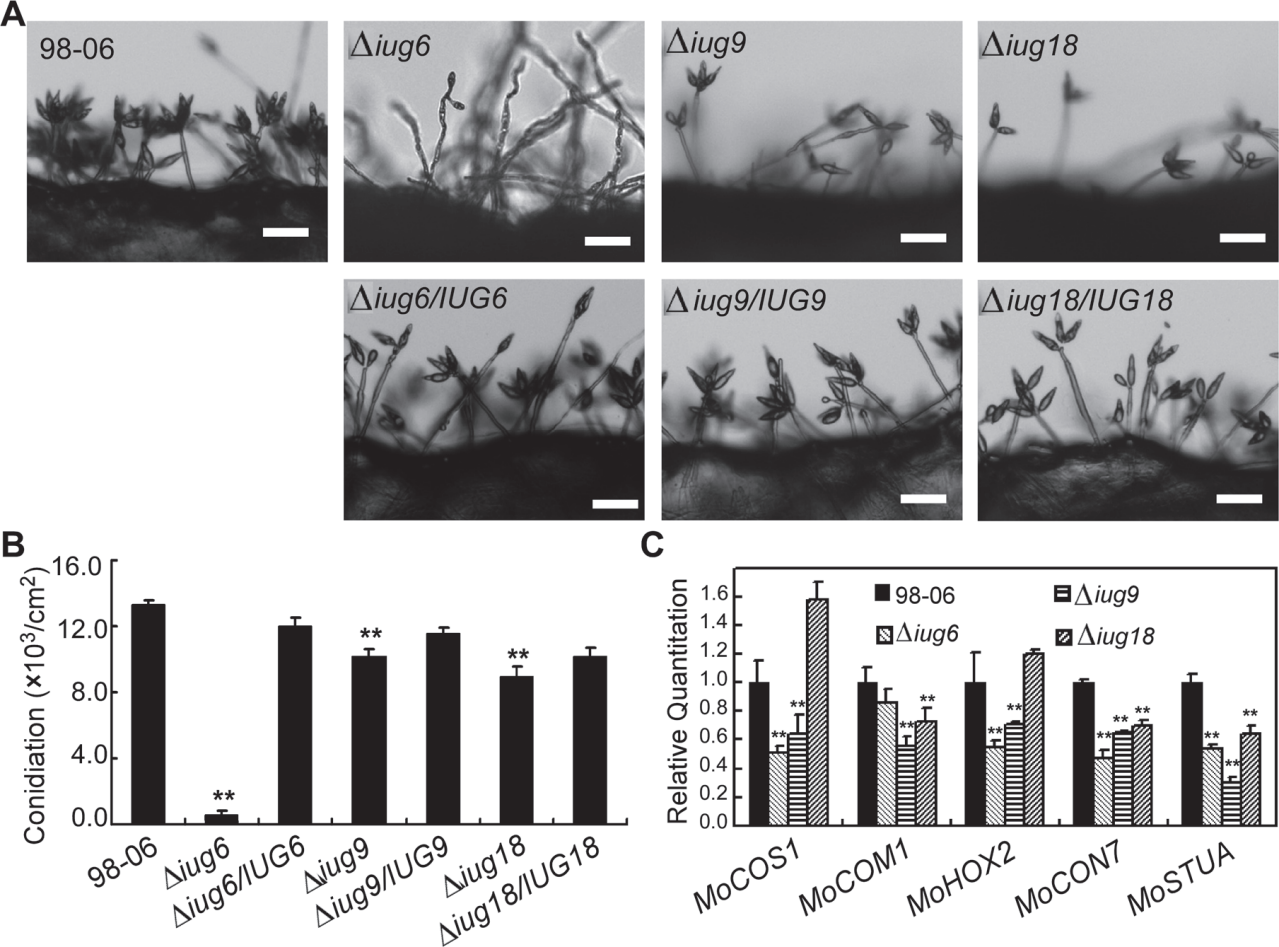
Disruption of *IUG* genes lead to reduced conidiation. **(A)** Development of conidia on conidiophores is affected by *IUG* genes deletion. Light microscopy was observed on strains grown on SDC medium for 7 days. Bars = 100 μm. **(B)** Statistical analysis of conidial production by wild type, Δ*iug6*, Δ*iug9*, Δ*iug18*, and complemented strains. **(C)** Reduced expression patterns are found in five genes encoding conidiation-associated genes in all mutants. RNA was extracted from mycelia grown in liquid CM for 2 days. Error bars represent the standard deviation and asterisks represent significant differences (*P*<0.01).

**Fig 7:**
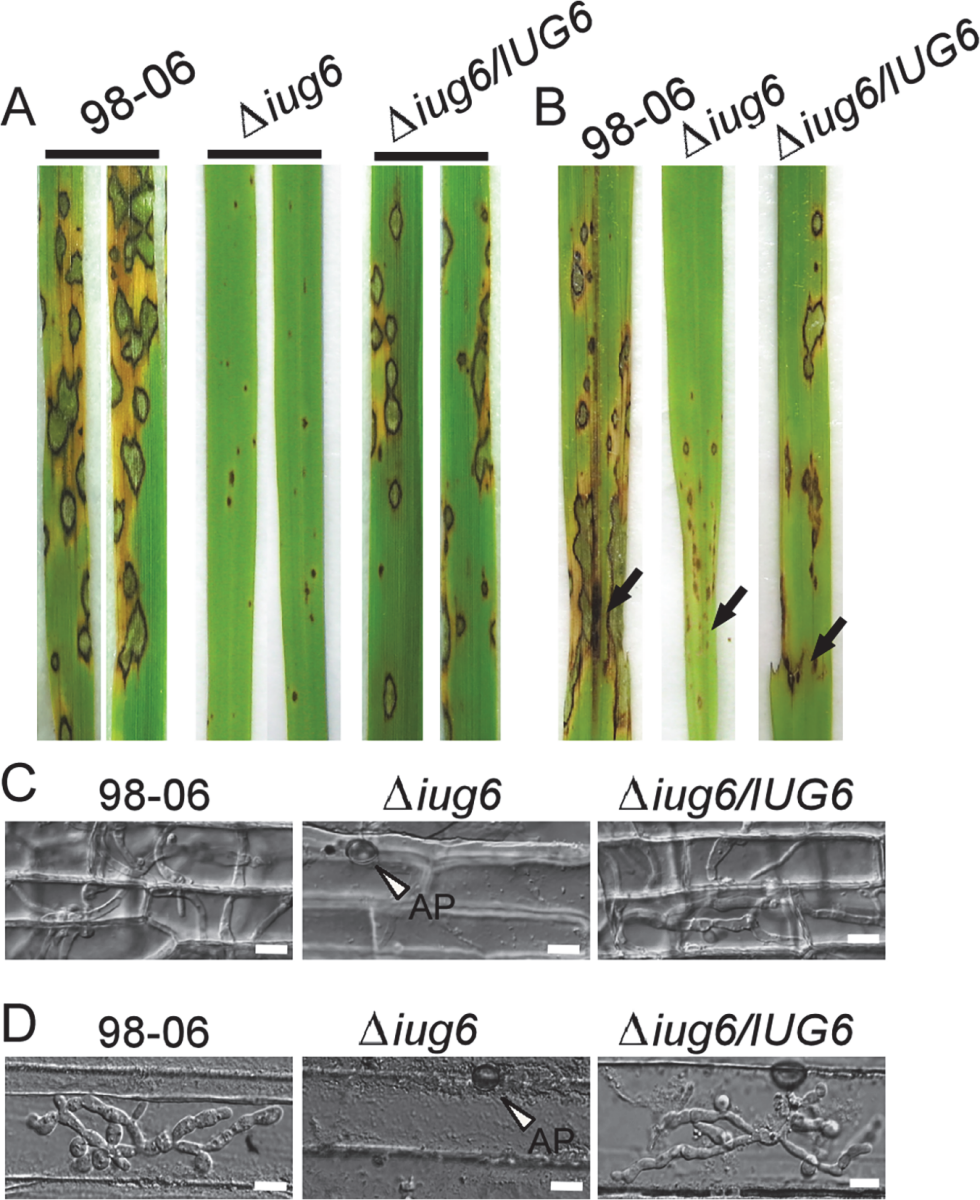
*IUG6* is involved in pathogenicity of *M*. *oryzae*. **(A)** The *IUG6* deletion leads to a significant reduction in pathogenicity on rice leaves. 4 ml of conidial suspension (5 x 10^4^ spores /ml) for each strain was sprayed on two-week-old rice seedlings (*O*. *sativa* cv CO-39) and 60 healthy rice plants were used in each independent experiment. Diseased leaves were harvested 7 days after inoculation. **(B)** Leaves of 4-week-old rice seedlings were injected with conidial suspensions of 98–06, Δ*iug6* and complemented strain. Diseased leaves were photographed 7 days after inoculation. **(C)** Rice leaf sheath penetration assay indicates severely confined growth of the Δ*iug6* mutant hyphae at 48 hpi compared to 98–06. 50 infection sites were examined for each experiment and experiments were repeated twice with similar results. Bars = 20 μm. AP: appressoria. **(D)** Infection hyphae were observed in the cells on the back side of barley leaves at 48 hpi. 50 infection sites were examined for each experiment and experiments were repeated twice with similar results. Bars = 20 μm.

**Fig 8:**
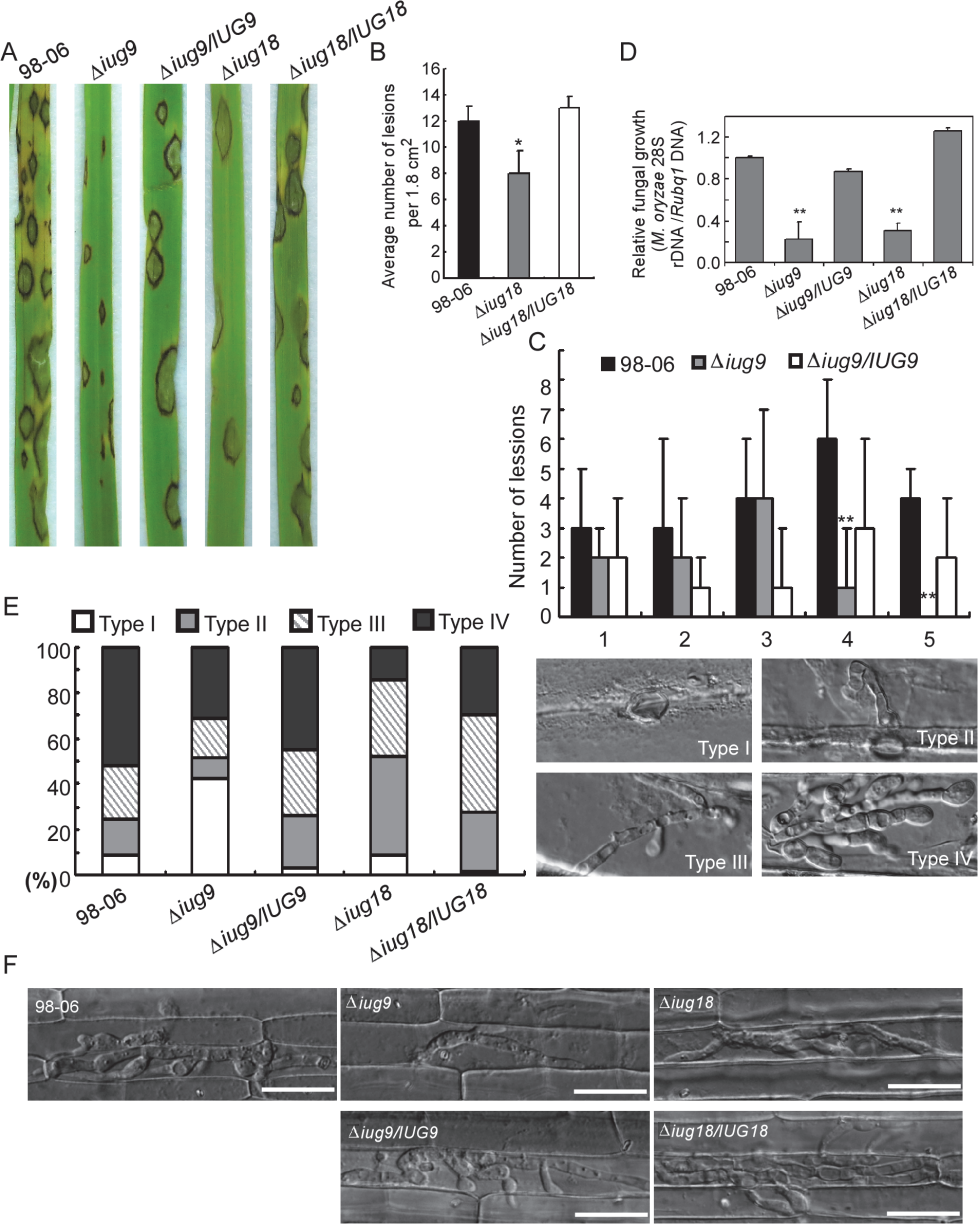
*IUG9* and *IUG18* are involved in pathogenicity of *M*. *oryzae*. **(A)** Disease symptoms were reduced on rice leaves inoculated with Δ*iug9* and Δ*iug18* mutants. Conidial suspension (5 x 10^4^ spores/ml) of the wild-type strain 98–06, mutants and complemented strains were inoculated on rice (cv. LTH), and incubated for 7 days. **(B)** Bar chart of mean lesion density of seedlings infected with isolate 98–06 and the Δ*iug18* mutant per unit area. Mean lesion density was significantly reduced in Δ*iug18* mutant infections. Error bars represent the standard deviation and asterisks represent significant differences (*P*<0.01). **(C)** Quantification of lesion types (0, no lesion; 1, pinhead-sized brown specks; 2, 1.5-mm brown spots; 3, 2–3-mm grey spots with brown margins; 4, many elliptical grey spots longer than 3 mm; 5, coalesced lesions infecting 50% or more of the leaf area) reveals no difference in lesion types 1–3; however, the Δ*iug9* mutant make rarely lesions of types 4 and 5. Lesions were photographed and measured or scored at 7 days post-inoculation (dpi) and experiments were repeated twice with similar results. **(D)** Severity of blast disease was evaluated by quantifying *M*. *oryzae* genomic 28S rDNA relative to rice genomic *Rubq1* DNA (7 days post-inoculation). Mean values of three determinations with standard deviations are shown. The asterisks indicate a significant difference from the 98–06 (*P* < 0.01). **(E)** Percentage of difference infection hyphae type (I = no infection hyphae; II = only one infection hyphae; III = two or three branches of the infection hyphae; IV = more than three branches of infection hyphae), occupied by each strain in the reverse side cells of barley 32 h after inoculation. The total number of appressorium-mediated penetration and infection is indicated (top right corner, N = 100). **(F)** Typical infection sites of rice leaf sheath inoculated with 98–06 strain, Δ*iug18*, Δ*iug9* mutants, and complemented strains, showing greater fungal proliferation and tissue invasion by the wild-type strain. Infectious growth was observed at 30 hpi. Bars = 50 μm.

The Δ*iug6*, Δ*iug9*, and Δ*iug18* mutants were examined for phenotypes including conidiogenesis. Conidiation in 10-d-old cultures of the *iug6* mutant was reduced dramatically, by ~17-fold, compared with WT (Fig 6B). The Δ*iug9* and Δ*iug18* mutants showed approximately 14% and 32% reduction in conidiation on SDC medium (Fig 6B). Microscopic observations showed that Δ*iug6*, Δ*iug9*, and Δ*iug18* mutants produced significantly fewer conidia than the WT strain (Fig 6A). To determine whether *IUG6*, *IUG9*, and *IUG18* affect the expression of conidiation-related genes including *MoCOM1*, *MoHOX2*, *MoCON7*, *MoCOS1*, and *MoSTUA* [41,42,43,44,45], we measured and found that their expression was decreased (Fig 6C). To determine whether Δ*iug6*, Δ*iug9*, and Δ*iug18* have defects in pathogenicity, conidial suspensions (5 x 10^4^ spores /ml) were sprayed onto 2-wk-old susceptible rice seedlings (CO-39 & LTH). Only small, necrotic-like dark brown spots were observed in Δ*iug6*-infected rice leaves in comparison to controls (Fig 7A and 7B). When the lesions were excised, surface sterilized with 70% ethanol for 1 min, and incubated with light and humidity on 4% water agar for 2 days, as described in a previous study [46], no fungal growth or conidia occurred. The Δ*iug9* and Δ*iug18* mutants also resulted in a reduction in disease symptoms on rice 7 days after inoculation (Fig 8A). The mean lesion density per unit area of the mutants was significantly lower than that of WT (Fig 8B). Disease symptoms of three approximately 6 cm long rice blades from the same parts of plants infected by Δ*iug9* mutant or WT were also quantified using a ‘lesion-type’ scoring assay [9], which showed that lesion types 4 and 5 (severe, coalescing) were rarely produced by the Δ*iug9* mutant (Fig 8C). In addition, fungal DNA in rice was significantly lower in infection by Δ*iug9* and Δ*iug18* mutants than that by the WT as determined by *M*. *oryzae* 28S rDNA quantitation [47] (Fig 8D).

Finally, we performed a detailed phenotypic analysis to investigate the infectious hyphae within the host cells. At 24 hpi in barley epidermal cells and 48 hpi in rice leaf sheaths, both the WT and the Δ*iug6* mutant strain formed normal appressoria, but the invasive hyphae (IH) of the WT strain freely expanded into host cells. On the other hand, appressoria of Δ*iug6* failed to develop any penetration structures among 50 examined infection sites in rice and barley, respectively (Fig 7C and 7D). We also analyzed invasive hyphal growth of Δ*iug9* and Δ*iug18* mutants at 100 appressorial penetration sites in barley tissues by rating the hyphal growth into four types (type I, no penetration; type II, with penetration peg; type III, with a single invasive hypha; type IV, with extensive hyphal growth) after inoculation with the spore suspensions for 30 hours (Fig 8E). 75% of penetration sites of WT showed III or IV invasive growth type, by contrast 42% of cells were limited in type I invasive hyphal growth in the Δ*iug9* mutant, and 77% penetration sites of the Δ*iug18* mutant showed type II or III hyphal growth. A similar result was also observed in rice leaf sheaths, with the WT and complemented strains displaying faster hyphal growth extension to neighboring cells while the Δ*iug9* and Δ*iug18* mutants showing lower hyphal growth limited to one cell (Fig 8F).

### Iug6 and Iug9 are accumulated in BICs

To further characterize these Iug proteins, we expressed *IUG6*:*GFP*, *IUG9*:*GFP*, and *IUG18*:*GFP* fusion genes under the control of their native promoters in Guy11, respectively. GFP fluorescence was observed in the conidial septum expressing Iug6:GFP and Iug9:GFP, while faint fluorescence was seem in the cytoplasm of conidia expressing Iug18:GFP (S12 Fig).

**Fig 9:**
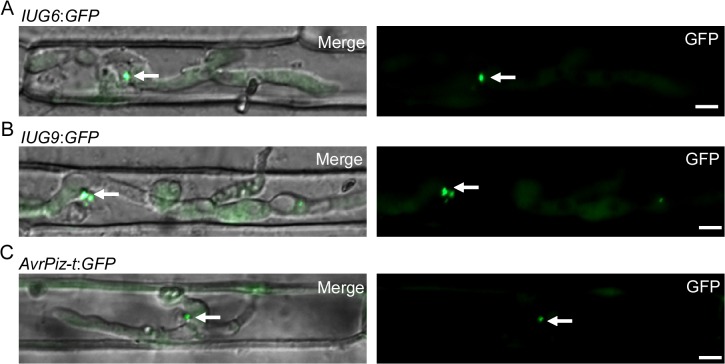
Iug6 and Iug9 proteins accumulate at BICs in sheath epidermal cells. **(A)** Cellular localization of Iug6:GFP in *M*. *oryzae* during biotrophic growth on epidermal rice cells at 27 hpi. Fluorescence was observed accumulating preferentially at BICs. Merged DIC and GFP images and GFP fluorescence alone are shown. BICs are indicated by arrows. Bars = 10 μm. **(B)** Secretion of Iug9:GFP at 27 hpi. Fluorescence was at BICs. Bars = 10 μm. **(C)** AvrPiz-t:GFP was observed preferential BIC localization at 30 hpi. Bars = 10 μm.

We next detected whether any GFP signals can be detected in the biotrophic interfacial complex (BIC). To suppress host immunity during biotrophic intracellular growth, fungal effectors of *M*. *oryzae* are secreted and accumulated at the BIC or more generally within the Extra-Invasive Hyphal Membrane (EIHM) [4]. When transformants invaded rice sheath cells at ~27 hpi, the fluorescence of Iug6 can be observed in BIC (Fig 9A). Microscopy of the secreted Iug9:GFP protein showed fluorescence that outlines the primary hyphae and BICs at ~27 hpi (Fig 9B), similar to the BIC localization control (Fig 9C), AvrPiz-t:GFP [48]. Both findings suggested that Iug6 and Iug9 could be delivered into the rice cytoplasm and accumulated in BICs to facilitate biotrophic invasion.

To validate the signal peptide prediction of Iug6 and Iug9, we used an assay based on the method previously described by Oh et al. [49]. The constructs with the signal peptides of Iug6 and Iug9 allowed the invertase secretion-deficient yeast strain YTK12 to grow on YPRAA medium, and this invertase activity was confirmed by inclusion of the triphenyl tetrazolium chloride dye (S13 Fig). These results showed that the signal peptides of *IUG6* and *IUG9* are indeed functional.

We further transformed the *IUG6* gene into the virulent isolate Guy11 and used the Δ*iug9* mutant for pathogenicity comparison tests in four resistant rice cultivars: IRBLz5-CA (*Pi-z5*), IRBLsh-S (*Pi-sh*), IRBL20-IR24 (*Pi-20*), IRBLta2-Re (*Pi-ta2*), and susceptible cultivar LTH. All of the transformants showed disease symptoms (S14 Fig), excluding the possibility that *IUG6* and *IUG9* are conventional *AVR* genes corresponding to these specific resistance genes. However, further studies are underway to address whether or not *IUG6* and *IUG9* are unknown avirulence genes that are involved in the evolutionary host-pathogen arms race.

### SA and ET signaling of rice is suppressed in the presence of Iug6 and Iug9

**Fig 10:**
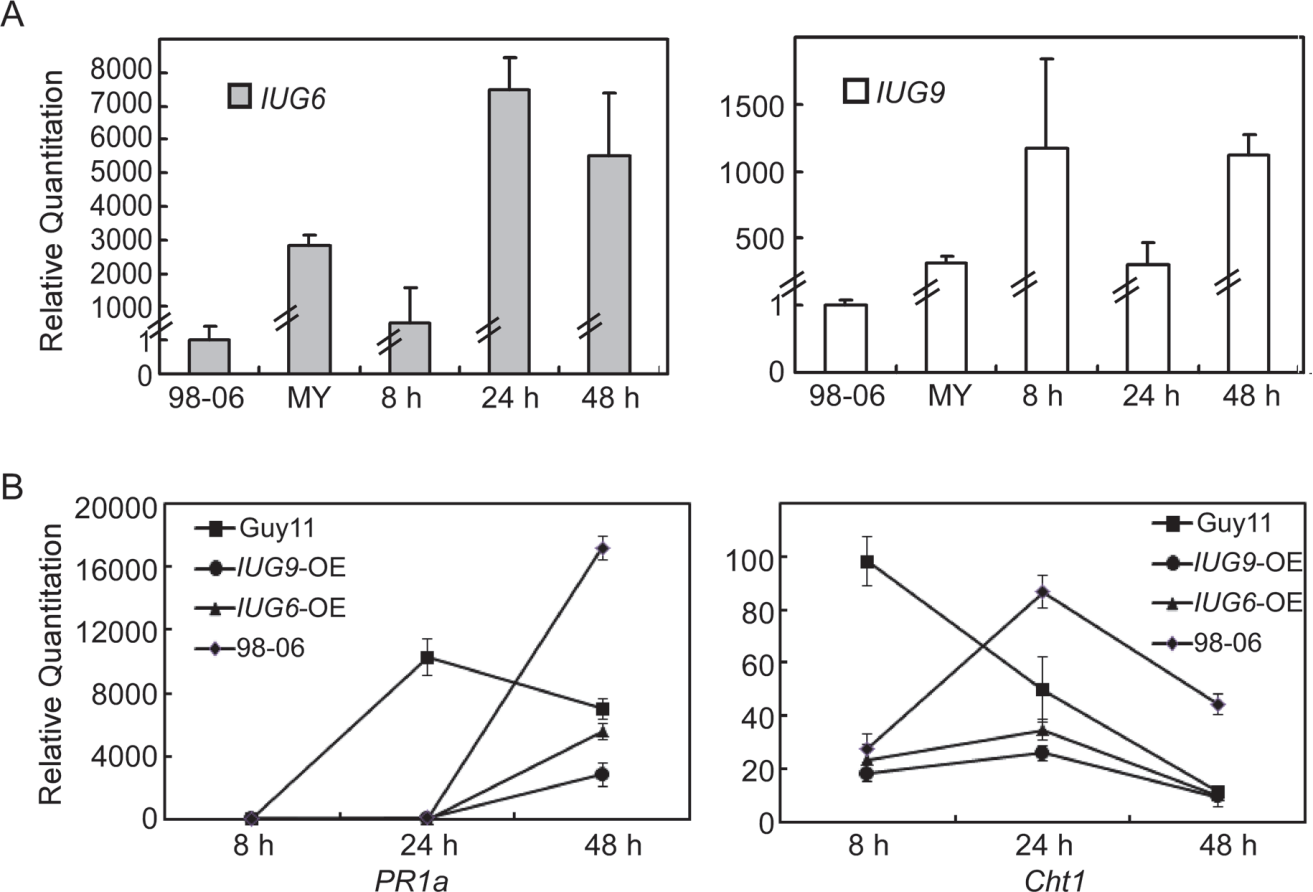
Over-expression of *IUG6* or *IUG9* in Guy11 suppresses defense-related genes in rice. **(A)** qRT-PCR on *IUG6* or *IUG9* at different fungal developmental stages in Guy11 overexpression transformants in comparison with mycelium phase of isolate 98–06. RNA was extracted from mycelia and infectious stages (8, 24, and 48 hpi), respectively. **(B)** Expression of *PR1a* and *Cht1* marker genes in the infected rice is suppressed in the presence of Iug6 or Iug9. RNA samples were collected from rice plants (*O*. *sativa* cv CO-39) 0, 24, and 48 h after inoculation with the Guy11, 98–06, or OE strains. The average threshold cycle (Ct) of triplicate reactions was normalized by the stable expressions *ACTIN* gene in *O*. *sativa*. Three independent biological experiments were performed and yielded similar results. Error bars in the figure represent the standard deviation.

As Iug6 and Iug9 can suppress the BAX-mediated programmed cell death in tobacco leaves, we investigated whether their expression affects the transcription of the rice defense-related genes. When infected with Guy11 over-expressing *IUG6* and *IUG9*, the pathogenicity of these transformants is not obviously improved, but the expression of *PR1a* and *Cht1* in rice was significantly less than that caused by Guy11 following qRT-PCR analysis (Fig 10B). Similarly, the expression levels of *PR1a* and *Cht1* in rice by infection with isolate 98–06 were also significantly lower than that infected with Guy11 at 24 hpi or 8 hpi, with delayed peak expression. The expression patterns of *PR1a* and *Cht1* in rice when infected with isolates over-expressing *IUG6* or *IUG9* is similar to that by isolate 98–06, with somewhat lowered peak expression. *PR1a* and *Cht1* are respective SA and ET signaling marker genes. SA is involved in establishing basal defenses, effector-triggered immunity, and systemic acquired resistance in many dicotyledonous species [50,51], as well as in modulating redox balance and protecting rice plants from the oxidative stress caused by *M*. *oryzae* [52]. Our above interaction transcriptome analysis also suggested that SA and ET signaling pathways might function positively on rice basal defense against *M*. *oryzae*. We hypothesized that Iug6 and Iug9 might target unknown factors in rice, leading to suppression of the SA and ET signaling and promotion of biotrophy.

## Discussion

*M*. *oryzae* is known for its natural genetic variation in the field, resulting in emergence of new epidemics threatening world food supply. Comparative analyses of multiple new epidemics not only steadily improved the assembly and annotation and the identification of variations in the *M*. *oryzae* genome, but also open a door to explore new virulence mechanism of the pathogen for effective blast control [2,9]. In this study, we reported the genome and interactive transcriptome analyses of isolate 98–06 that is known to be one dominant field isolate containing as many as seven *AVR* genes (*Avr-Pik/km/kp*, *AvrPiz-t*, *Avr-Piz5*, *Avr-Pita2*, *Avr-Pish*, *Avr-Pi20*, and *Avr-Pi9*). Among them, *Avr-Pik/km/kp* [16], *AvrPiz-t* [38], and *Avr-Pi9* [53] were characterized. *Avr-Pik/km/kp* (allele D) and *AvrPiz-t* were also identified in our study.

Our analysis indicated that isolate 98–06 contains an extra 1.4 Mb of genomic sequences not present in 70–15, while a whole-genome dot-plot alignment of 98–06 and P131 suggested good genome synteny (S15 Fig). To examine whether the extra genomic region is present in other isolates, we searched the genomes of isolates P131 and Y34 that found that 49 genes remain unique to isolate 98–06. COG classification revealed that, while the functions for most of these genes were unknown, those identified exhibit functions relevant to general characteristics, secondary metabolites, and energy metabolism (S2 Fig).

Yoshida et al. [16] observed that presence or absence of effector gene polymorphisms are often associated with unstable genomic regions near the chromosome ends. It is therefore hypothesized that isolate-specific regions located at the chromosomal ends supply new effectors and pathogenicity-related factors to drive genome evolution. The dynamic adaptation of *M*. *oryzae* may be primarily achieved by deletion or recovery of *AVR* genes [54]. Such hypotheses indicate that further genome sequencing of multiple *M*. *oryzae* isolates is warranted for characterizing rice pathogenic strains, as key information can be discovered only by exploring beyond the “core” genomes [16]. One good example is that the extensive chromosomal rearrangement in asexual fungus *V*. *dahliae* establishes dynamic, lineage-specific regions that provide new effectors as a general mechanism of adaptation [35]. Although sexual reproduction can occur in *M*. *oryzae* under laboratory conditions, it has not been observed in nature. Such asexual organisms are often considered to be less flexible than sexual organisms [55,56], so that the expanded genome is likely to represent examples of evolutionary tradeoffs, as the cost of maintaining the extra DNA is counterbalanced by the functional advantages it confers [57]. Since horizontal gene transfer (HGT) enables the acquisition of new genes and functions [57], we hypothesize that some of these isolate-specific genes might also be acquired by HGT to drive the evolutionary process.

Based on the genome data of isolate 98–06, our transcriptome analysis provides a further molecular view of *M*. *oryzae* and rice gene expression during infection. The major expression patterns of *M*. *oryzae* genes relevant to pathogenicity remain high throughout the infection process, with the exception of a sharp decline at 24 hpi (Fig 3A). The decreased expression pattern might due to low levels of transcript detection at 24 hpi, with only 2,426 genes (*M*. *oryzae*) compared with at least 5,161 genes for other infection stages. This is consistent with the previous discovery that expression of known pathogenicity genes was unchanged or down-regulated at 36 hpi [4]. We should point out that our RNA-Seq analyses were carried out without sample duplication that could result in potentially biased interpretation of the transcriptome data. Nevertheless, the result is agreeable with that from validation of several selected genes through qRT-PCR.

Effectors are known key pathogenicity determinants that modulate plant innate immunity and enable disease development in plant-pathogen interactions [58]. Several *AVR* genes have been previously isolated by map-based cloning, genetic association analysis, or loss-of-function approaches [7,16,59,60,61,62]. Most of these effectors are small secreted proteins lacking homology to known proteins [63,64,65]. In characterizing the isolate 98–06 genome, we found 645 small candidate effectors with an average 3.11% cysteine content from 1,732 putative secreted proteins. Biotrophic invasive-specific expression is one of the best indicators for identifying new blast effectors [65]. Based on the gene expression data, we found that *BAS1*, *BAS3*, *BAS4*, *SLP1*, and *AvrPiz-t* effectors all show the similar expression pattern during infection (Fig 3A). Based on the expression of *Avr-Pik* and *AvrPiz-t*, 134 candidate effectors were further explored using hierarchical clustering (Fig 4), including six characterized effectors *BAS1*, *BAS2*, *BAS3*, *BAS4*, *SLP1*, and *PWL1*. Among them, two isolate-unique effectors Iug6, Iug9, and three non-isolate unique proteins Nup1, Nup2, Nup3 were selected to show a similar function in blocking plant immunity (Fig 5). Our characterization of three *IUG* genes indicates that these 134 putative effectors were a rich source for additional effector identification. In our study, a high frequency in suppression of cell death induced by BAX in tobacco leaves was observed that may also be associated with effects of heterologous gene expression or the unfolded protein response (UPR) [66]. Therefore, additional studies are necessary to further characterize these effector candidates. Our functional analysis of *IUG6* and *IUG9* indeed showed that they are important for full virulence expression of isolate 98–06. In addition, *IUG6* and *IUG9* characterization revealed the gene absence/presence polymorphism among 29 field stains (Fig 5C), similar to six *AVR (Avr-Pita1*, *Avr-Pik*, *Avr-Pia*, *Avr-Pii*, *PWL2*, and *ACE1)* genes that exhibited a prevalence of presence/absence polymorphisms among 62 *M*. *oryzae* isolates [67]. The similarity is consistent with that *IUG6* and *IUG9* might be *AVR* effectors.

Functional characterization indicated that Iug6 and Iug9, as well as Iug18, are important for mycelial growth, sporulation, and pathogenicity (Fig 7 and Fig 8). Xue et al. [17] have also observed that several randomly selected isolate-specific genes play important and diverse roles, some affecting virulence while others affecting conidiation and vegetative growth. There are, however, several secreted proteins that are required for pathogenicity, such as MoMpg1, MoEmp1, MoMhp1, MoMsp1, MC69, and Slp1 [62,68,69,70,71]. MC69, which is potentially localized in BIC, is essential for successful appressorial penetration and pathogenicity [68]. Slp1 suppresses chitin-induced host defense responses, thereby facilitating rapid spread of the fungus within the host [71]. Targeted gene disruption of *BAS1*, *BAS2*, and *BAS3* did not show any effects on pathogenic phenotypes, whereas *BAS4* disruption did not provide any viable transformants [4]. Furthermore, over-expressing *IUG6* or *IUG9* in Guy11 can suppress the expression of *PR1a* and *Cht1* in rice, which are marker genes of SA and ET pathways, respectively. These results are also supportive of the effector role for Iug6 and Iug9. Meanwhile Iug18 appears to be a PaR protein based on the absence of a secretion signal peptide.

Gene deletion and pseudogenization, silencing, amino acid replacements, protein chimerization, and new gene addition are all important factors for altering virulence in filamentous plant pathogens [57]. Our microsyntenic and Southern blotting analyses showed that *IUG6*, *IUG9*, and *IUG18* are unique to isolate 98–06 compared to 70–15 and Guy11 (S10 Fig). Iug18 is present in several isolates belongs to the Rel-Spo like protein superfamily. Blast search indicates that Iug6 and Iug18 have homologs in *G*. *graminis*, while Iug9 has a homolog from *C*. *higginsianum*. An emerging question would be how some *M*. *oryzae* isolates obtained these extra genes and where these genes come from. An attractive model was proposed to explain how *M*. *oryzae* regains deleted avirulence effector genes, in which parasexual exchange of genetic material enabled the recovery of ‘lost’ genes in asexual lineages [54]. A comprehensive population study revealed that the *Avr-Pita* gene has experienced a number of translocations in *M*. *oryzae* and related *Pyricularia* species, most likely as the consequence of its recovery by lateral transfer (HGT) [54]. Another example of translocation was recently reported, in which the *ToxA* gene was transferred from *Stagonospora nodorum*, a wheat pathogen, to another (*Pyrenophora tritici-repentis*) [72]. All these findings point out the likely source of these *IUG* genes.

On the other hand, it has been reported that *M*. *oryzae* has evolved two distinct secretion systems to deliver apoplastic and cytoplasmic effectors, which outline the entire invasive hyphae (IH) or accumulate in the BIC [33]. Like AvrPiz-t, Iug6 and Iug9 proteins are accumulated at BICs (Fig 9), suggesting that they have high possibility to be translocated into rice cell as effectors. Fungal apoplastic effectors are invariably Cys rich, but cytoplasmic effectors accumulated in BICs are not necessarily Cys rich [73]. Especially, Iug6, a small, secreted protein with four Cys residues accumulating in BICs, is similar to other known Cys-rich blast effectors like Bas2 and AvrPiz-t [4,38]. Effector genes are also often located near the telomeres, which tend to evolve at higher rates than the rest of the genome [10, 64, 65]. *IUG6* and *IUG9* are located on the subtelomeric regions of chromosome II and chromosome I, respectively (S14 Table), revealing that they could be the result of rapid adaptation to environmental conditions. This discovery further promotes the hypothesis that isolate-specific regions of chromosomal plasticity serve as facilitators of genome evolution in *M*. *oryzae*.

Overall, this study provides a systematic genomic and interaction transcriptome analysis of the dominant rice blast field isolate 98–06. Based on bioinformatics and functional analysis, two novel effectors Iug6 and Iug9 and a pathogenicity-related gene *IUG18* were characterized. This knowledge prompts the hypothesis that the isolate-unique genes beyond the “core” genome may act as a source for *M*. *oryzae* adaptation to the environment. Our analyses will facilitate further study of the roles of effectors and molecular mechanisms of pathogenesis and pathogen-host evolution.

## Materials and Methods

### Genome sequencing and assembling

The genome of *M*. *oryzae* 98–06 isolate was sequenced using a whole-genome shotgun approach. The genomic Illumina paired-end libraries were constructed with insertion size of 350 bp, 500 bp, 5 kb, and 10 kb, respectively, and sequenced at the Beijing Genomic Institute (BGI, China). The short reads of sequencing data (135-fold coverage) were assembled into genome sequence using SOAP denovo (version 1.05, http://soap.genomics.org.cn/soapdenovo.html), and then reads are mapped to contigs for partial assembling and filling gap through paired-end and overlap relationship between the reads [22,74]. The *M*. *oryzae* 70–15 genome sequence version 6 was downloaded from the Broad Institute (http://www.broadinstitute.org/annotation/genome/magnaporthe_grisea/MultiDownloads.html). The P131 and Y34 genome sequences were downloaded from the NCBI Genome Database (http://www.ncbi.nlm.nih.gov/genome).

### Gene prediction and annotation

CEGMA software was used to predict the core genes of isolate 98–06, which is based on the eukaryotes conservative gene database. We used the core gene set as the training set to predict genes by the Augustus and SNAP softwares [75,76]. In addition, we used the Homolog software to predict genes by using *Magnaporthe oryzae* isolate 70–15 as the reference sequence [77]. The Glean software was then employed to integrate the above results. Gene functions were predicted by comparison with the NCBI NR protein database and the KEGG [78], COG [79], SwissProt [80], GO [81] databases. Protein domain was predicted using SMART database. Membrane and sub-cellular localization domains were predicted by TMHMM 2.0 [82], SignalP4.0 [83], and NLStradamus [84].

Nucleotide sequences of the predicted isolate 98–06 genes were compared separately with the genomic sequences of P131, Y34, and 70–15 isolates with TBLASTN [85]. Homologous genes with sequence identities of 100%, 80–100%, and 50–80% were defined as identical, similar, and divergent, respectively, while those below 50% were considered non-homologous.

Amino acids of 52,177 proteins from all four isolates were compared with each other using TBLASTN (version 2.2.23) [85], and orthologs/paralogs families were clustered through OrthoMCL (version 1.4) [74].

### Transposable elements (TE) analysis and synteny mapping

We used three programs (RepeatMasker, RepeatProteinMasker, and Denovo) to predict transposon sequences [22]. We also used TRF (Tandem Repeat Finder) to identify tandem repeat sequences [86]. To search for genes disrupted by TEs, unique flanking sequences of TEs were used to search against 98–06 genes. Masked genome sequences of the 98–06 isolates were compared with the MUMMER package [87] to construct chromosome sequences for isolate 98–06 based on isolate 70–15 data.

### Effector genes prediction

The putative secreted proteins were identified using several prediction algorithms. TargetP 1.1 (http://www.cbs.dtu.dk/services/TargetP/) was used to predict the cleavage sites of the predicted presequences with the ‘‘Perform cleavage site predictions” option. SignalP 3.0 (http://www.cbs.dtu.dk/services/SignalP-3.0/) was used to predict signal peptide cleavage sites. Transmembrane helices were predicted using TMHMM 2.0 (http://www.cbs.dtu.dk/services/TMHMM-2.0/). Proteins that contain signal peptide cleavage sites but not transmembrane helices were selected as putative secreted proteins. Proteins of less than 200 amino acids in length were retained, and the average percentage of cysteine content was calculated.

### Interaction transcriptome analysis

Three-week-old rice plants (cv. CO-39) were inoculated with *M*. *oryzae* isolates at 1 x 10^8^ spores /ml. The inoculated plants were placed in a sealed plastic box in the dark for 24 h at 25°C, and leaf tissues were collected at 0 h, 8 h, 24 h, 48 h and 72 h after inoculation. Mycelia were grown in shaking culture in complete medium for 36 h at 28°C, 150 rpm and harvested. Total RNA was extracted using the Invitrogen kit as described previously [88]. RNA was extracted from samples that were the mixtures of three independent experiments.

Poly(A) mRNA was isolated from total RNA using oligo(dT) magnetic beads (Invitrogen, Carlsbad, CA, USA). Using RNA as templates, random hexamer primed cDNA synthesis was performed using reverse transcriptase (Invitrogen). Second-strand cDNA was synthesized using RNase H (Invitrogen), DNA polymerase I (New England Biolabs), dNTPs and buffer. DNA was purified using the QIAquick PCR extraction kit and ligated to sequencing adaptors following end repairing and Ploy (A) addition. Finally, the cDNA libraries were loaded onto the flow cell channels of an Illumina HiSeq^TM^ 2000 platform for paired-end 90 bp x 2 sequencing at the Beijing Genomics Institute (BGI), Shenzhen, China [89].

After discarding low-quality raw reads, the clean reads from each library were assembled to *M*. *oryzae* and rice genomes separately, and gene sequences were annotated using SOAP2 [90]. Gene expression levels were measured in the RNA-Seq analysis as reads per kilobase of exon model per million mapped reads (RPKM) [91]. Differentially expressed genes and their corresponding P-values were determined using the recent GFOLD algorithm, which could give more stable and biological meaningful gene ranking in comparison with other methods especially for single biological replicate experiments [92]. Fold changes (log_2_Ratio) were estimated according to the normalized gene expression level in each sample. We used the absolute value of log_2_Ratio ≥1 and GFOLD (0.01) >1 as the threshold to judge differentially expressed genes.

For KEGG pathway analysis [93], all the differentially expressed genes in the pathways were examined to uncover common expression patterns. For hierarchical clustering, Pearson’s correlation coefficient and Spearman’s rank were used to measure similarities between gene expression profiles and between samples, respectively. The heat map of the clustered genes and samples was generated by complete linkage.

### Quantitative RT-PCR assay

For RT-PCR and quantitative real time RT-PCR (qRT-PCR), 5 mg of total RNA was reverse transcribed into first-strand cDNA using the oligo(dT) primer and M-MLV Reverse Transcriptase (Invitrogen). The qRT-PCR reactions were performed following previously established procedures [82]. RNA-Seq expression profiles were validated by quantitative RT-PCR. Primer pairs used in this section are listed in S15 Table.

### Culture conditions and plant infection assays

All strains were cultured on complete medium (CM) agar plates at 28°C [69]. The *M*. *oryzae* isolate 98–06 was used as the wild type strain for transformation in this study. Protoplasts were prepared and transformed as previously described [94]. Conidiation assay was performed also as previously described [95]. Mycelia were harvested from liquid CM for genomic DNA and RNA extraction. Vegetative growth was measured on CM, minimal medium (MM), straw decoction and corn medium (SDC) and oatmeal medium (OM) plates for 7 days at 28°C [27]. The radial growth was measured after incubation for 7 days and then photographed. All experiments were repeated three times, each with three replicates.

Plant infection and injection assays were performed as previously described by spraying 4 ml of conidial suspensions (5 x 10^4^ spores /ml in 0.2% gelatin) on rice [96]. In order to distinguish resistant levels, spore suspensions were adjusted to 1 x 10^6^ spores/ml for AVR candidate pathogenicity tests. For microscopic observation, rice was inoculated with 100 μl of conidial suspension (5 x 10^4^ spores /ml) on the inner leaf sheath epidermal cells. After 48 h incubation under humid conditions at 28°C, leaf sheaths were collected and observed under a microscope. All experiments were repeated three times.

### Gene disruption and complementation

To generate the *IUG6* gene replacement vector pCX62, approximately 1 kb upstream and 1 kb downstream fragments were amplified with primer pairs (S15 Table). The resulting PCR products were ligated to the hygromycin resistance cassette released from pCX62, as previously described [97]. Putative mutants were screened by PCR and confirmed by Southern blotting analysis. To complement the Iug6 mutant, a DNA fragment including the putative promoter and the coding sequence was amplified and inserted into pYF11 (bleomycin resistance) by homologous recombination in *Saccharomyces cerevisiae*. The plasmids were extracted and transformed into *E*. *coli* competent cells, and then the plasmids with correct inserts were transferred into protoplasts, as previously described [97]. The same approach was used to generate mutants for isolate-specific genes *IUG9*, *IUG17*, *IUG18*, *IUG34*, and *IUG37*. The primer pairs used are listed in S15 Table. To observe secretion in rice cells, the coding sequences of *IUG6*, *IUG9*, and *AvrPiz-t* with their native promoters were fused with *GFP* in pYF11. To generate over-expression transformants of *IUG6* and *IUG9* in Guy11, the coding sequences of *IUG6* and *IUG9* driven by the ribosomal protein P27 promoter were inserted into pYF11, respectively. DNA primers are also listed in S15 Table.

### Yeast signal sequence trap system

The yeast signal trap system is based on vector pSUC2, which carries a truncated invertase gene, *SUC2*, lacking both the initiation Met and the signal peptide [98]. DNA fragments coding for the signal peptides of Iug6 and Iug9 were PCR amplified and introduced into pSUC2 using *EcoR*I and *Xho*I restriction sites to create in-frame fusion with the invertase (primers listed in S15 Table). The pSUC2-derived plasmids were then transformed into the invertase negative yeast strain YTK12 through lithium acetate method [98,99]. Following transformation, yeast cells were plated on CMD-W (minus Trp) plates, and positive colonies were transferred to fresh CMD-W plates and incubated at 30°C for two days. For invertase secretion, positive colonies were replica plated on YPRAA plates, supplemented with raffinose instead of sucrose. Growth occurs only when the invertase is secreted. The invertase enzymatic activity was measured by the reduction of TTC to insoluble redcolored triphenylformazan as described previously by Oh et al [49].

### Agrobacterium tumefaciens infiltration assays

The *IUG* and *NUP* genes without signal peptides were amplified using combinations of primers for the PVX assay. The amplified fragments were cut and ligated into the PVX vector PVX::HA [100,101] before introduction into *A*. *tumefaciens* strain GV3101 by electroporation [102]. The PVX::HA transformants were selected using Tetracycline (12.5 μg /ml) and Kanamycin (50 μg /ml). Individual colonies were verified by PCR. For infiltration of PVX::HA and PVX::flag (negative control GFP) into leaves, recombinant strains of *A*. *tumefaciens* were grown in LB medium plus 50 μg /ml Kanamycin for 48 h, harvested, and infiltrated as previously described by Yu et al [103]. The experiment was repeated three times with each assay consisting of three plants each with three leaves inoculations.

### Microscopy

Green fluorescent protein fluorescence in rice cells was captured using an LSM 710 laser scanning microscope with a 40 x objective lens (Carl Zeiss), with an excitation 480 ± 10 nm and an emission 510 ± 10 nm.

### Accession numbers

The genome sequence data of 98–06 was deposited at the NCBI Genome Database (http://www.ncbi.nlm.nih.gov/assembly) under the accession number JRBC00000000. RNA-Seq reads were deposited at the GenBank SRA database under sample number SRS692257 and experiment number SRX689727. The GenBank accession numbers for *IUG6*, *IUG9*, and *IUG18* are KM522919, KM522920, and KM522921, respectively. The information of other genes with their reference gene ID in 70–15 and gene location in the 98–06 genome is provided in S9 Table.

## Supporting Information

S1 Fig Whole genome comparison between 98–06 and 70–15. Simple global view of syntenic alignments from eight chromosomes. The orange lines illustrate forward alignment, and the blue lines illustrate reverse alignment.
(TIF)

S2 Fig Clusters of Orthologous Groups (COG) classification of 98–06 isolate-specific genes. (TIF)

S3 Fig Venn diagram of gene clusters in different isolates. The numbers in the circles represent different sets of gene clusters, including orthologs of any two isolates, and common to all four isolates, respectively. A: 98–06; B: 70–15; C: P131; D: Y34
(TIF)

S4 Fig Differentially expressed genes at different infection stages during compatible interactions. **(A)** Number of up- or down-regulated genes under each condition for *M*. *oryzae* in comparison to conidial infection 0 h. **(B)** Number of up- or down-regulated genes under each condition for rice in comparison to conidial infection 0 h. (The sample of CO-0h contains uninfected rice leaves.)
(TIF)

S5 Fig Validation of detected genes by qRT-PCR. **(A)** Five genes of *M*. *oryzae* and four genes of rice were randomly selected and illuminated by the log_2_RPKM value in different libraries. The fold change was RPKM value of different stages in contrast with conidial infection 0 h, respectively. **(B)** Nine genes were calculated in the original sequenced samples by Ct value of qRT-PCR analysis in contrast with conidial infection 0 h. **(C)** Nine genes were calculated in the samples of another independent biological experiment by Ct value of qRT-PCR analysis in contrast with conidial infection 0 h.
(TIF)

S6 Fig Expression patterns of SNARE genes and endocytosis-related genes. **(A)** CAST assay of 21 SNARE genes showed five different expression patterns, indicating the typical process of pathogen-host interaction. **(B)** CAST assay of 35 endocytosis-related genes showed eight different expression patterns, distinguishing a similar interaction expression pattern. The y axis stands for the log2 average gene expression levels. The quantity of cluster member is marked at the right bottom of each pattern line.
(TIF)

S7 Fig Iug6, Iug9, Nup1, Nup2, and Nup3 did not trigger cell death symptoms in *N*. *benthamiana*. Leaves of *N*. *benthamiana* were infiltrated with *A*. *tumefaciens* carrying pGR106-BAX, pGR106-GFP, pGR106-Iug6, Iug9, Nup1, Nup2, or Nup3, respectively. Photographs were taken 8 DAI. The experiment was repeated three times.
(TIF)

S8 Fig Southern hybridization of *IUG* genes disruption. Southern blot analyses of the genes knockout mutants with gene specific probe and hygromycin phosphotransferase (*HPH*) probe2. **(A)** Construction strategies for gene knocking out vector. Thick arrows indicate orientations of the genes and *HPH* genes. Thin lines below the arrows indicate the probe sequence of each gene. **(B)** Southern hybridization was used to analyze *IUG* genes disruption. The restriction enzymes used for Southern blot were: *EcoR* I (*IUG37*), *EcoR* V (*IUG34*), *Cla* I (*IUG9* and *IUG17*), *Hind* III (*IUG6*, *IUG18*).
(TIF)

S9 Fig The growth rate of different strains on various media plates. **(A)** Colony morphology was observed on CM, MM, OM, and SDC medium for 7 days at 28°C. **(B)** The colony diameters were measured and subjected to statistical analysis. The experiment was performed in triplicate. Error bars represent standard deviation and double asterisks represent significant differences (*P*<0.01), one asterisk represents significant differences (*P*<0.05).
(TIF)

S10 Fig Compare the location and southern hybridization of isolate-specific *IUG* genes. **(A)** 10 kb sequences up- and down-stream of *IUG6*, *IUG9*, and *IUG18* are picked out to analyze synteny between 98–06 and 70–15. For each drawing, the above lines represent region of 70–15, and the below lines represent region of 98–06. Isolate-special genes and sequences are shaded in red, *IUG* genes shaded in green, homologous genes presented by blank arrows. Orange stands for reverse alignment. **(B)** Southern blot of *IUG6*, *IUG9*, and *IUG18*. Genomic DNA from 98–06, Guy11, P131, Y34, and 70–15 was digested respectively. The restriction enzymes used for Southern blot were *Hind* III (*IUG6*, *IUG18*) and *Cla* I (*IUG9*), respectively. The probe of each gene was used to validate their presence.
(TIF)

S11 Fig The phase specific expression of *IUG* genes. The expression was measured by quantitative real-time RT-PCR with cDNA from samplings for infectious growth, vegetative growth, and conidia. The relative abundance of *IUG* transcripts during infectious growth (from conidia to in planta fungal cells 72 hpi) was normalized by comparing with vegetative growth in liquid CM (Relative transcript level = 1). The lower RQ values are labeled on top of histogram. The error bar represents the standard deviation.
(TIF)

S12 Fig Microscopic analysis of the GFP fluorescence in conidia. Conidia from Iug6:GFP, Iug9:GFP, and Iug18:GFP were harvested. Bars = 10 μm.
(TIF)

S13 Fig Functional validation of the signal peptides of *IUG6* and *IUG9*. The experiment was performed using the yeast invertase secretion assay. Yeast YTK12 strains carrying the Iug signal peptide fragments fused in frame to the invertase gene in the pSUC2 vector are able to grow in both the CMD-W media and YPRAA media (with raffinose instead of sucrose, growth only when invertase is secreted), as well as reduce TTC to red formazan, indicating secretion of invertase. The controls include the untransformed YTK12 strain and YTK12 carrying the pSUC2 vector.
(TIF)

S14 Fig Pathogenicity tests of Guy11 transformed with *IUG6* and Δ*iug9* mutant on different rice cultivars. The concentrations of spore suspension were adjusted to 1 x 10^6^/ml for spray inoculation on on four resistant rice cultivars and susceptible cultivar LTH. Inoculated plants were placed in a moist chamber at 28°C for first 24 h in darkness, and then transferred back to another moist chamber with a photoperiod of 12 h under fluorescent lights. The disease severity was assessed at 7 days after inoculation.
(TIF)

S15 Fig Whole-genome dot-plot comparison between 98–06 and P131. (TIF)

S1 Table Pathotypes of 98–06 based on their infectivity towards different monogenic rice cultivars. (DOC)

S2 Table Isolate-specific sequences in 98–06 compared to 70–15. (DOC)

S3 Table Isolate-unique genes in 98–06 compared to 70–15. (DOC)

S4 Table Summary of gene clusters in all four isolates; gene paralogs; expanded gene families in 98–06. (XLS)

S5 Table Transposable elements identified in isolate 98–06, P131, Y34, and 70–15. (DOC)

S6 Table Secreted protein genes of 98–06 disrupted by TE. (DOC)

S7 Table 645 small candidate effector proteins. (DOC)

S8 Table Summary of RNA-Seq reads and mapping work. (XLS)

S9 Table List of RPKM values for all detected fungal genes in 98–06 and rice genes. (XLS)

S10 Table Transcriptome data of 64 known pathogenicity genes and 10 known effectors. (DOC)

S11 Table CAST assay of 21 SNARE genes. (DOC)

S12 Table CAST assay of 35 endocytosis-related genes. (DOC)

S13 Table 134 candidate effectors. (DOC)

S14 Table The subtelomeic locations of *IUG6* and *IUG9*. The locations of *IUG6* and *IUG9* are based on their flank sequences corresponding to chromosome of 70–15. *IUG6* and *IUG9* locate on the subtelomeric positions of chromosome II and chromosome I, respectively.
(XLS)

S15 Table Primers used in this study. (DOC)
