# Supplementary figures and images for: Global Genome and Transcriptome Analyses of Magnaporthe oryzae Epidemic Isolate 98-06 Uncover Novel Effectors and Pathogenicity-Related Genes, Revealing Gene Gain and Lose Dynamics in Genome Evolution
Source: PLoS Pathog. 2015 Apr 2;11(4):e1004801. doi: 10.1371/journal.ppat.1004801 (PMC4383609; doi:10.1371/journal.ppat.1004801)

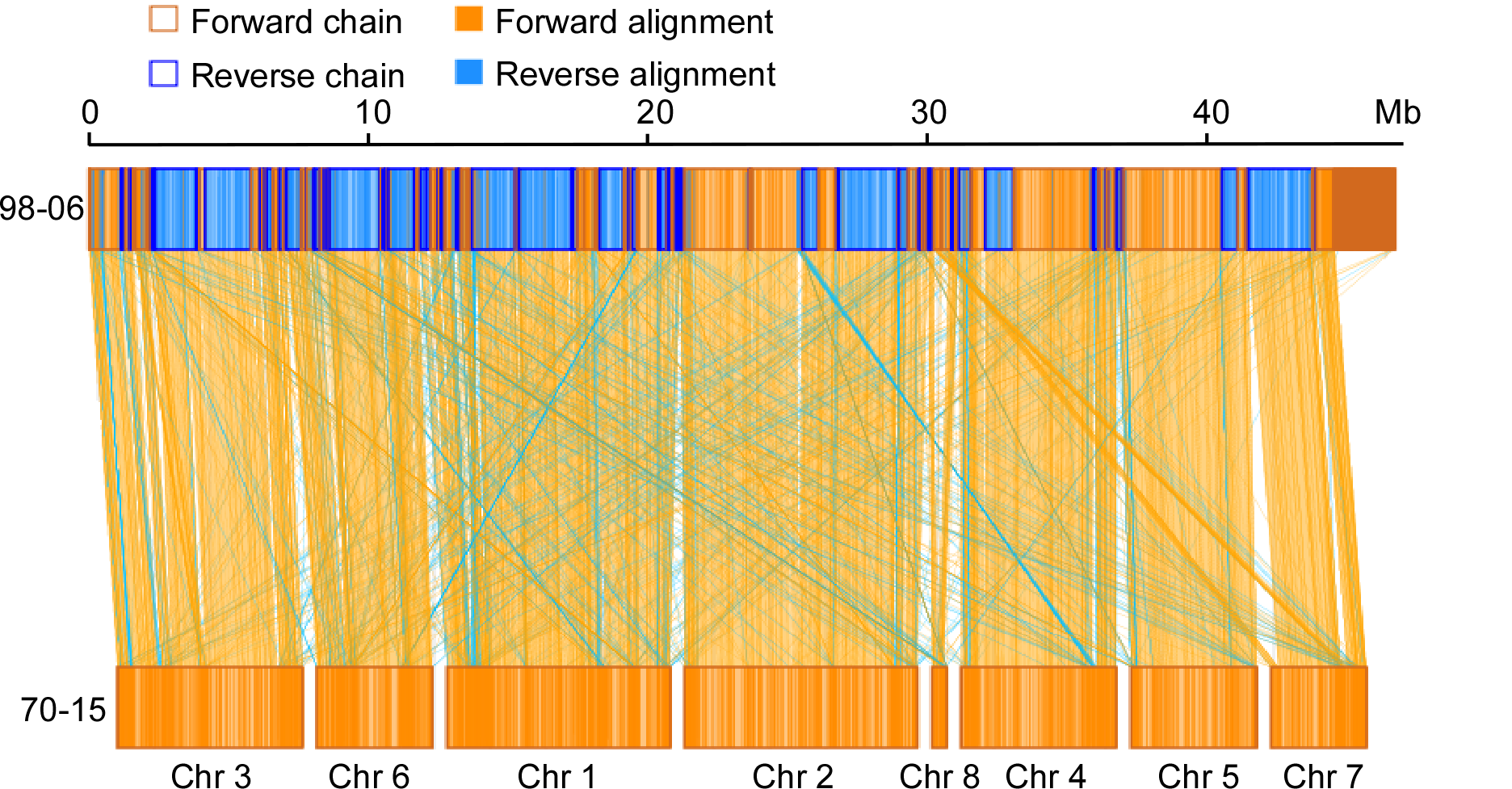

Supplement: S1 Fig — Simple global view of syntenic alignments from eight chromosomes. The orange lines illustrate forward alignment, and the blue lines illustrate reverse alignment. (TIF) [file ppat.1004801.s001.tif]

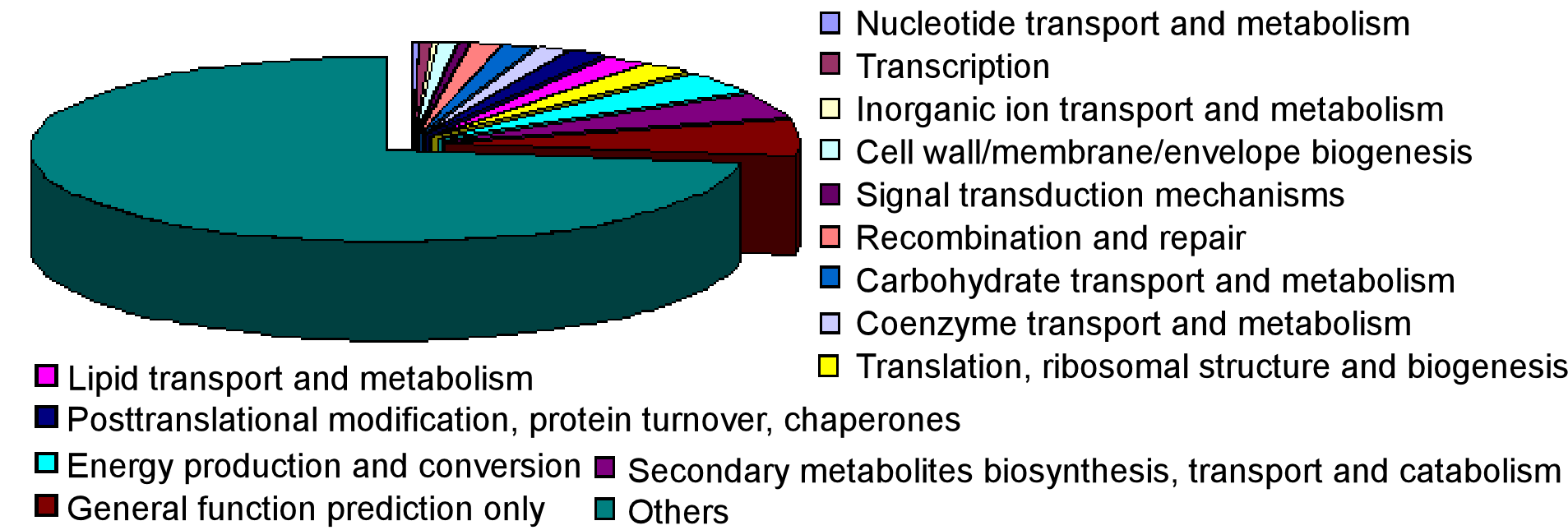

Supplement: S2 Fig — (TIF) [file ppat.1004801.s002.tif]

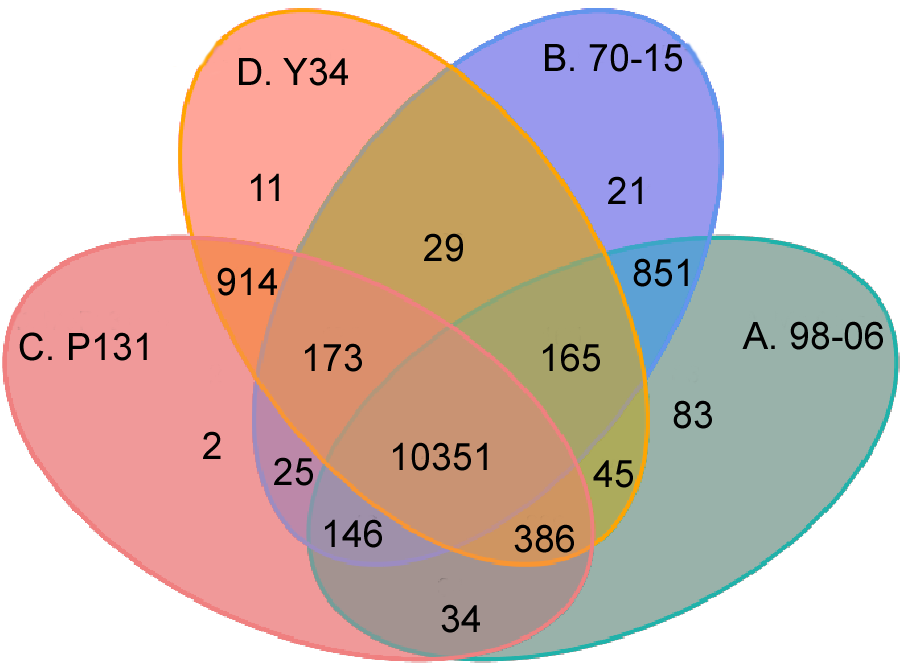

Supplement: S3 Fig — The numbers in the circles represent different sets of gene clusters, including orthologs of any two isolates, and common to all four isolates, respectively. A: 98–06; B: 70–15; C: P131; D: Y34 (TIF) [file ppat.1004801.s003.tif]

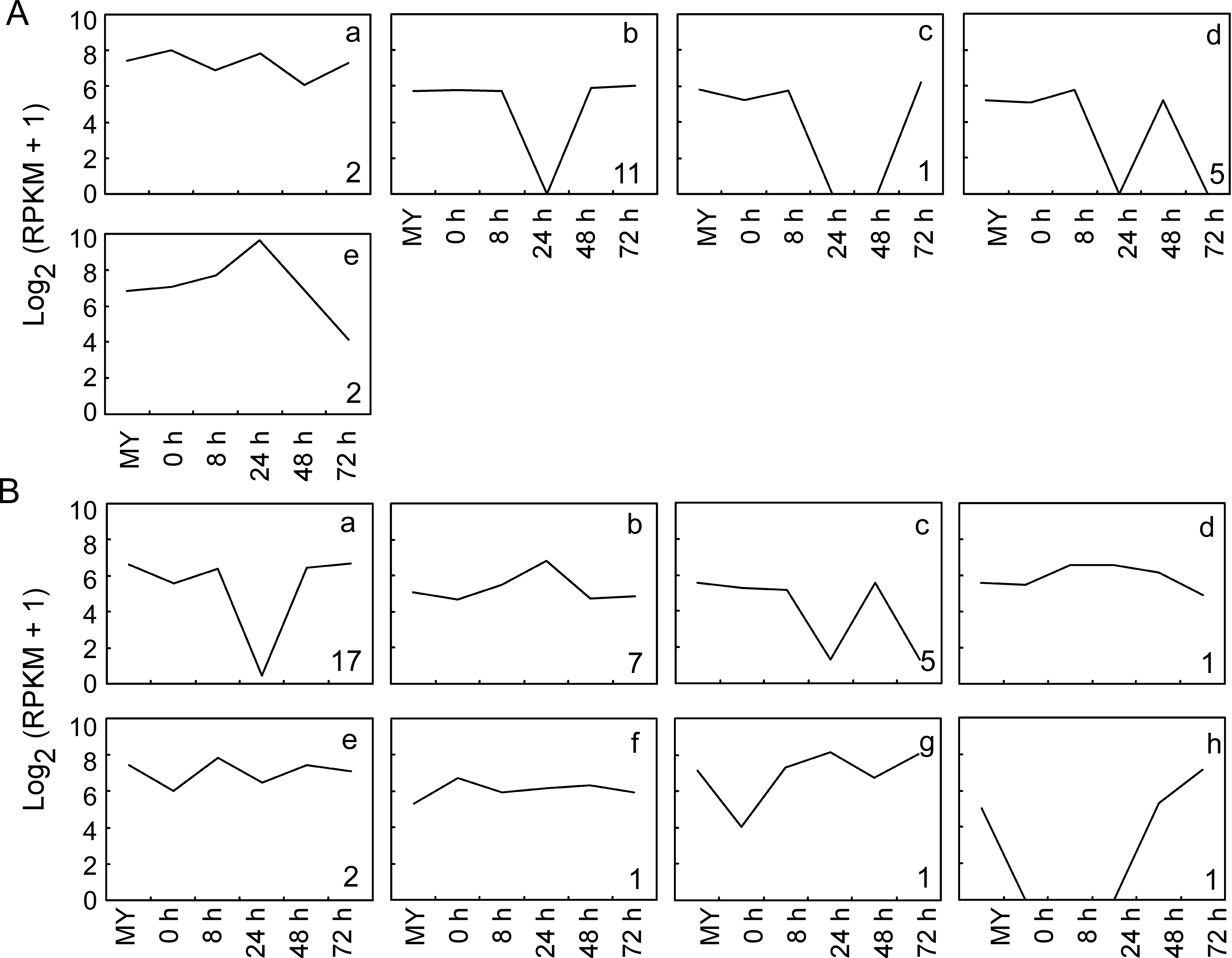

Supplement: S6 Fig — (A) CAST assay of 21 SNARE genes showed five different expression patterns, indicating the typical process of pathogen-host interaction. (B) CAST assay of 35 endocytosis-related genes showed eight different expression patterns, distinguishing a similar interaction expression pattern. The y axis stands for the log2 average gene expression levels. The quantity of cluster member is marked at the right bottom of each pattern line. (TIF) [file ppat.1004801.s006.tif]

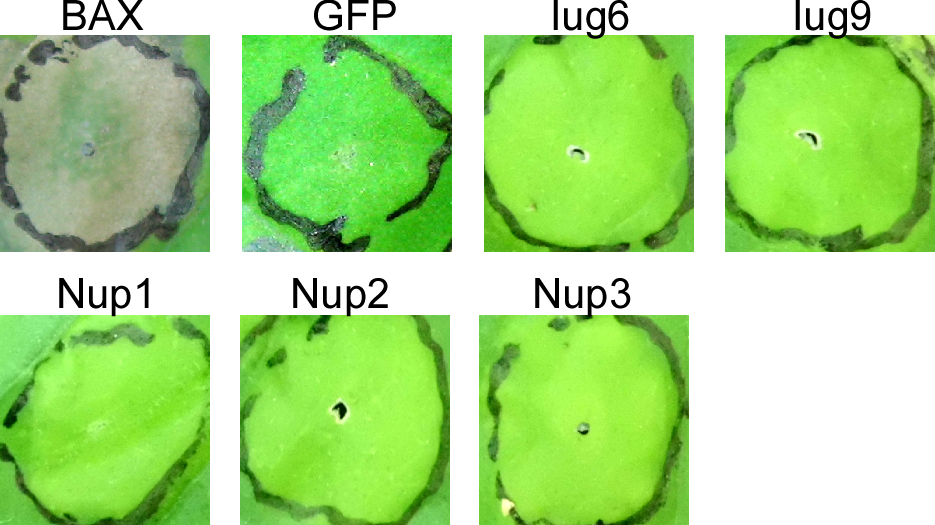

Supplement: S7 Fig — Leaves of N. benthamiana were infiltrated with A. tumefaciens carrying pGR106-BAX, pGR106-GFP, pGR106-Iug6, Iug9, Nup1, Nup2, or Nup3, respectively. Photographs were taken 8 DAI. The experiment was repeated three times. (TIF) [file ppat.1004801.s007.tif]

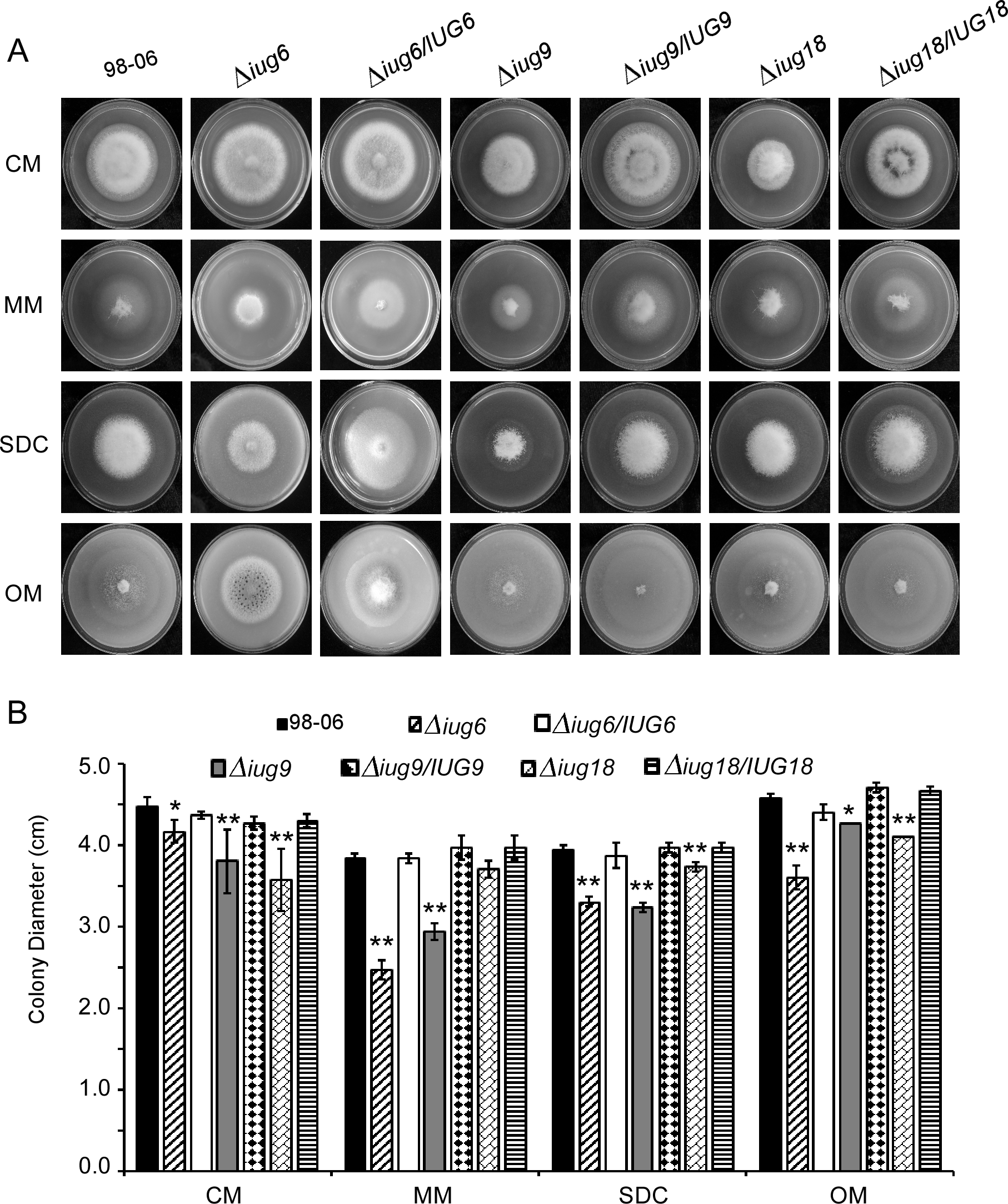

Supplement: S9 Fig — (A) Colony morphology was observed on CM, MM, OM, and SDC medium for 7 days at 28°C. (B) The colony diameters were measured and subjected to statistical analysis. The experiment was performed in triplicate. Error bars represent standard deviation and double asterisks represent significant differences (P<0.01), one asterisk represents significant differences (P<0.05). (TIF) [file ppat.1004801.s009.tif]

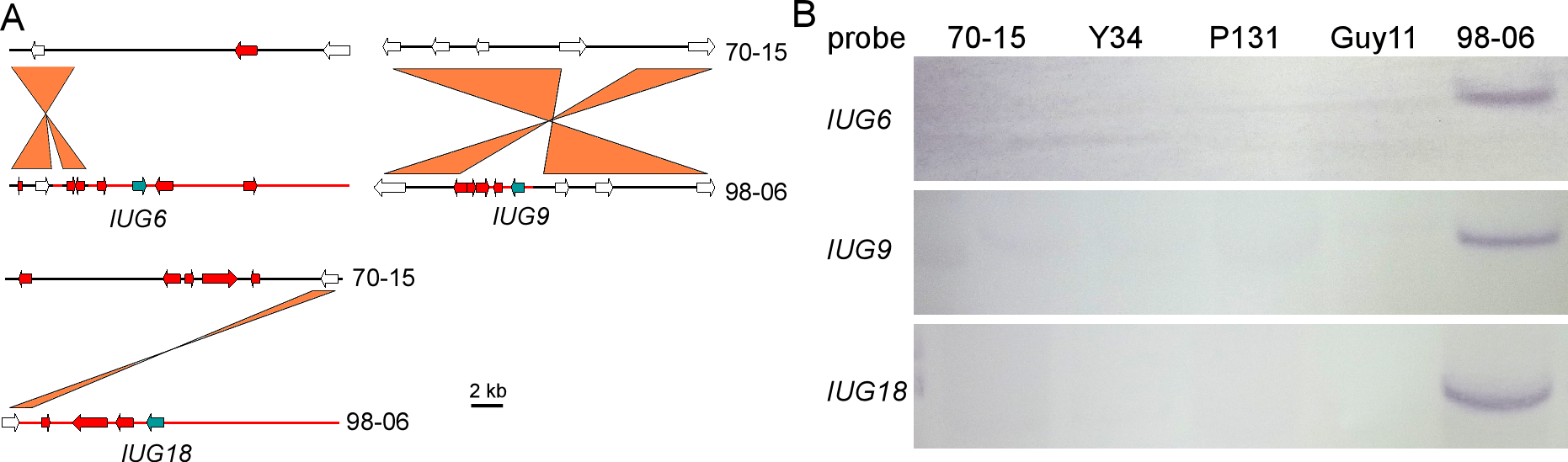

Supplement: S10 Fig — (A) 10 kb sequences up- and down-stream of IUG6, IUG9, and IUG18 are picked out to analyze synteny between 98–06 and 70–15. For each drawing, the above lines represent region of 70–15, and the below lines represent region of 98–06. Isolate-special genes and sequences are shaded in red, IUG genes shaded in green, homologous genes presented by blank arrows. Orange stands for reverse alignment. (B) Southern blot of IUG6, IUG9, and IUG18. Genomic DNA from 98–06, Guy11, P131, Y34, and 70–15 was digested respectively. The restriction enzymes used for Southern blot were Hind III (IUG6, IUG18) and Cla I (IUG9), respectively. The probe of each gene was used to validate their presence. (TIF) [file ppat.1004801.s010.tif]

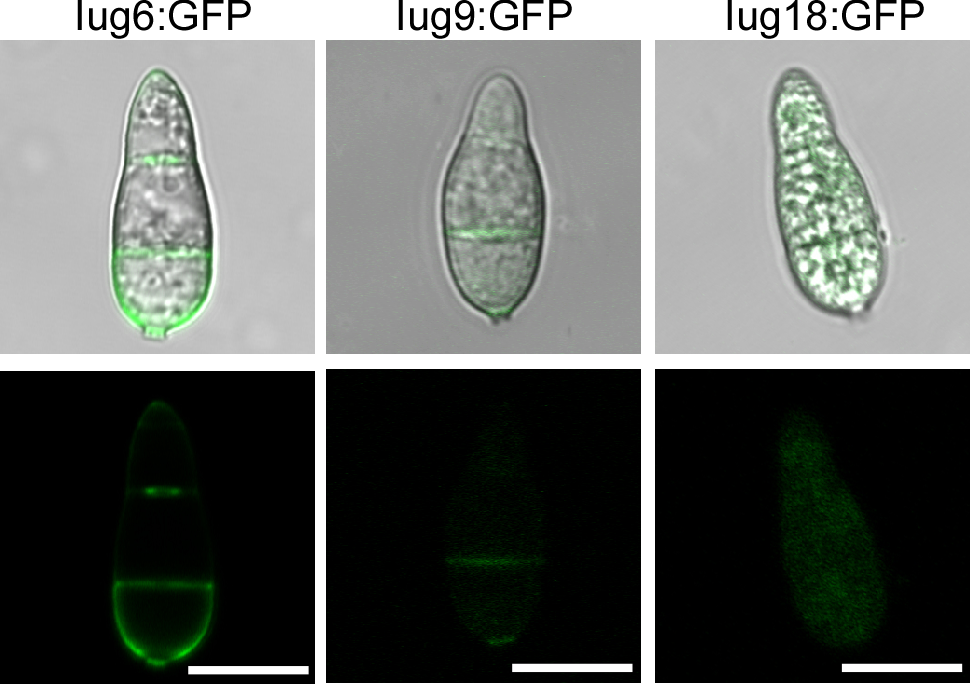

Supplement: S12 Fig — Conidia from Iug6:GFP, Iug9:GFP, and Iug18:GFP were harvested. Bars = 10 μm. (TIF) [file ppat.1004801.s012.tif]

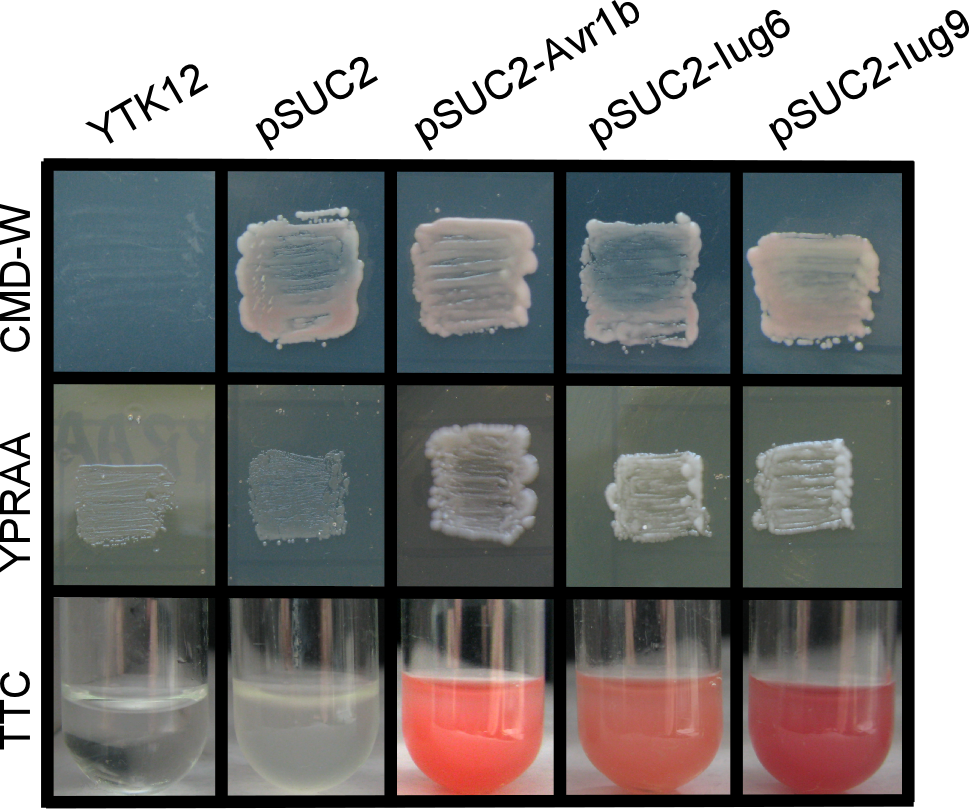

Supplement: S13 Fig — The experiment was performed using the yeast invertase secretion assay. Yeast YTK12 strains carrying the Iug signal peptide fragments fused in frame to the invertase gene in the pSUC2 vector are able to grow in both the CMD-W media and YPRAA media (with raffinose instead of sucrose, growth only when invertase is secreted), as well as reduce TTC to red formazan, indicating secretion of invertase. The controls include the untransformed YTK12 strain and YTK12 carrying the pSUC2 vector. (TIF) [file ppat.1004801.s013.tif]

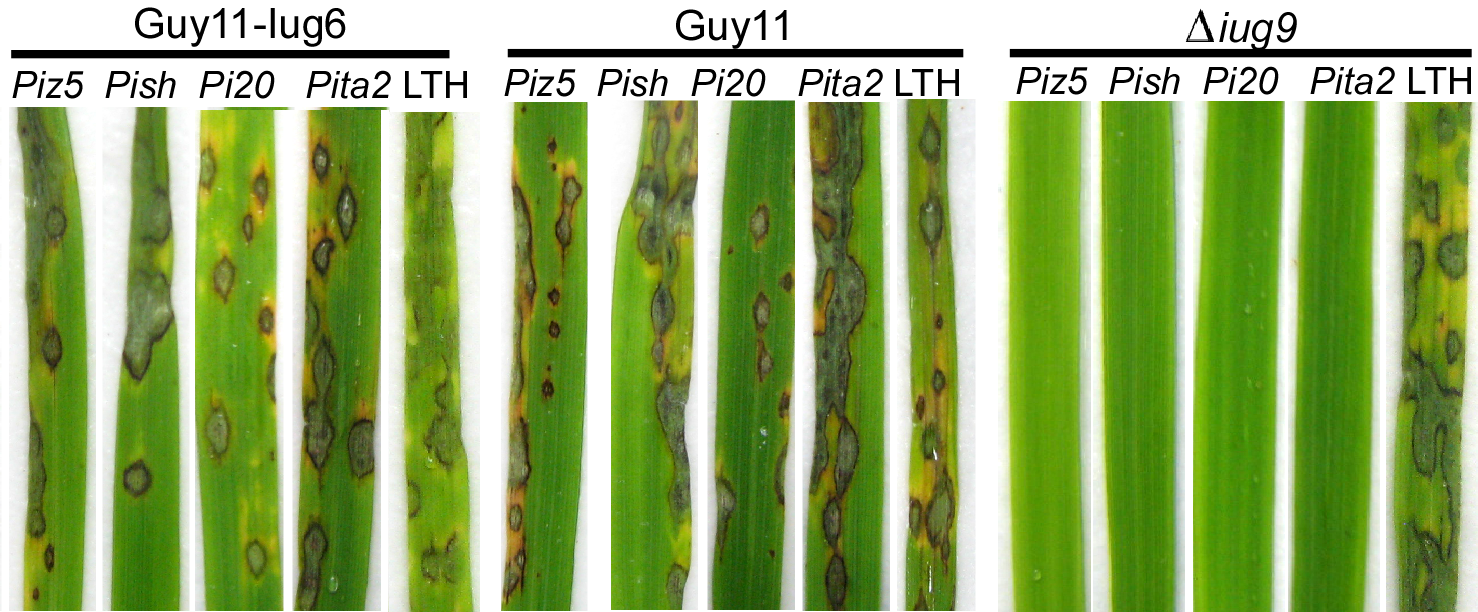

Supplement: S14 Fig — The concentrations of spore suspension were adjusted to 1 x 10^6^/ml for spray inoculation on on four resistant rice cultivars and susceptible cultivar LTH. Inoculated plants were placed in a moist chamber at 28°C for first 24 h in darkness, and then transferred back to another moist chamber with a photoperiod of 12 h under fluorescent lights. The disease severity was assessed at 7 days after inoculation. (TIF) [file ppat.1004801.s014.tif]

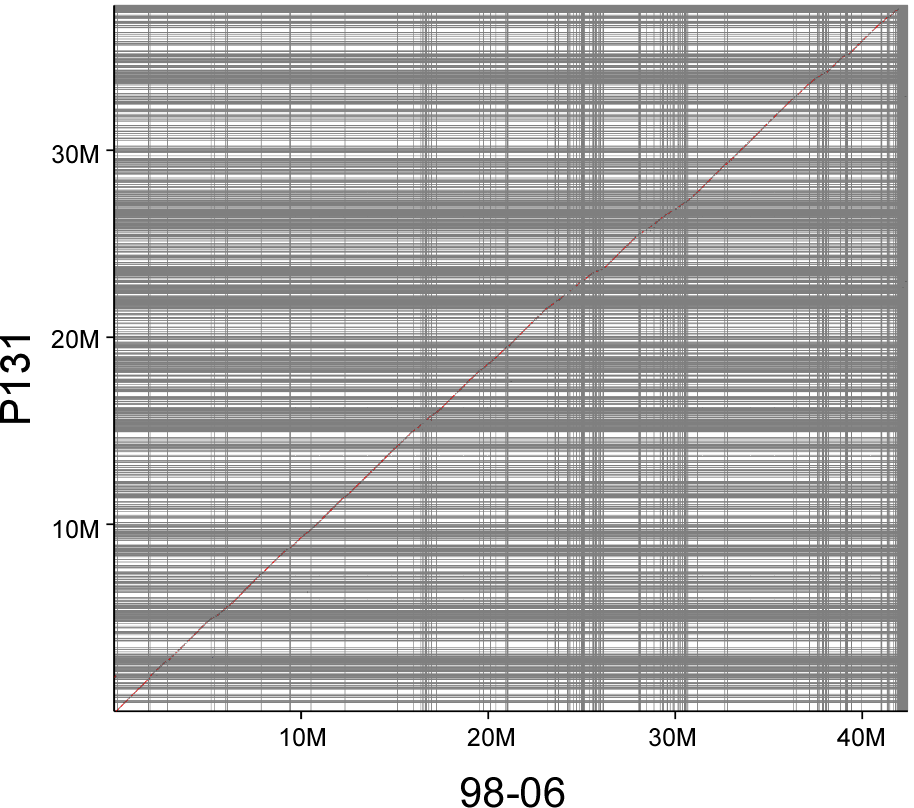

Supplement: S15 Fig — (TIF) [file ppat.1004801.s015.tif]
