# Supplementary material for: Global Genome and Transcriptome Analyses of Magnaporthe oryzae Epidemic Isolate 98-06 Uncover Novel Effectors and Pathogenicity-Related Genes, Revealing Gene Gain and Lose Dynamics in Genome Evolution
Source: PLoS Pathog. 2015 Apr 2;11(4):e1004801. doi: 10.1371/journal.ppat.1004801 (PMC4383609; doi:10.1371/journal.ppat.1004801)
Supplement: S1 Table — (DOC) [file ppat.1004801.s016.doc]

**Omic analyses on one epidemic rice blast isolate result in discovery of novel virulence factors**

Yanhan Dong^1^, Ying Li^1^, Miaomiao Zhao^1^, Maofeng Jing^1^, Muxing Liu^1^, Xinyu Liu^1^, Xianxian Guo^1^, Xing Zhang^1^, Yue Chen^1^, Yongfeng Liu^2^, Yanhong Liu^2^, Wenwu Ye^1^, Haifeng Zhang^1^, Xiaobo Zheng^1^, Ping Wang^3^, and Zhengguang Zhang^1^*

**^1^**Department of Plant Pathology, College of Plant Protection, Nanjing Agricultural University, and Key Laboratory of Integrated Management of Crop Diseases and Pests, Ministry of Education, Nanjing 210095, China

^2^BGI-Shenzhen, Main Building 11/F, Beishan Industrial Zone, Yantian District , Shenzhen 518083, China

**^3^**Department of Pediatrics and the Research Institute for Children, Louisiana State University Health Sciences Center, New Orleans, Louisiana 70118, USA

*Corresponding author:

Zhengguang Zhang, Email: [zhgzhang@njau.edu.cn](mailto:zhgzhang@njau.edu.cn)

Tel: 86-25-84396972

Fax: 86-25-84395325

**Table S1** **Pathotypes of 98-06 based on their infectivity towards different monogenic rice cultivars generated by the International Rice Research Institute.**

| **Monogenic cultivar^a^** | **R gene** | **98-06** | **Donor cultivar** |
| --- | --- | --- | --- |
| IRBLa-A | *Pia* | S | Aichi Asahi |
| IRBLi-F5 | *Pii* | S | Fujisaki5 |
| IRBLks-F5 | *Pik-s* | S | Fujisaki5 |
| IRBLk-Ka | *Pik* | R | Kanto51 |
| IRBLkp-K60 | *Pik-p* | R | K60 |
| IRBLkh-K3 | *Pik-h* | R | K3 |
| IRBLz-Fu | *Piz* | S | Fukunishiki |
| IRBLz5-CA | *Piz5* | R |  |
| IRBLzt-T | *Piz-t* | R | Toride 1 |
| IRBLta-K1 | *Pita* | S | K1 |
| IRBLb-B | *Pib* | S | BL1 |
| IRBLt-K59 | *Pit* | S | K59 |
| IRBLsh-S | *Pi-sh* | R | Shin2 |
| IRBL1-CL | *Pi1* | R | C101LAC |
| IRBL3-CP4 | *Pi3* | S | C104PKT |
| IRBL5-M | *Pi5* | S | Moroberekan(RIL249) |
| IRBL7-M | *Pi7* | R | Moroberekan(RIL29) |
| IRBL9-W | *Pi9* | R | WHD-1S-75-1-127 |
| IRBL12-M | *Pi12* | S | Moroberekan(RIL10) |
| IRBL19-A | *Pi19* | S | Aichi Asahi |
| IRBLkm-Ts | *Pik-m* | R | Tsuyuake |
| IRBL20-IR24 | *Pi-20* | R | IR24 |
| IRBLta2-Re | *Pita2* | R | Reiho |
| IRBL11-Zh | *Pi11* | S | Zhaiyeqing 8 |

a: The monogenic rice lines were generated by crossing ‘LTH’ with individual donor cultivars containing R gene.

S, susceptible; R, resistant.
