# Supplementary material for: Global Genome and Transcriptome Analyses of Magnaporthe oryzae Epidemic Isolate 98-06 Uncover Novel Effectors and Pathogenicity-Related Genes, Revealing Gene Gain and Lose Dynamics in Genome Evolution
Source: PLoS Pathog. 2015 Apr 2;11(4):e1004801. doi: 10.1371/journal.ppat.1004801 (PMC4383609; doi:10.1371/journal.ppat.1004801)
Supplement: S2 Table — (DOC) [file ppat.1004801.s017.doc]

**Table S2** Isolate-specific sequences in 98-06 compared to 70-15.

| **Location** | **Start** | **End** | **Chain (+/-)** |
| --- | --- | --- | --- |
| scaffold_1 | 109796 | 109797 | + |
| scaffold_1 | 117564 | 118101 | + |
| scaffold_1 | 118439 | 118707 | + |
| scaffold_1 | 119225 | 119421 | + |
| scaffold_1 | 1202900 | 1202900 | + |
| scaffold_1 | 120717 | 120728 | + |
| scaffold_1 | 121021 | 121085 | + |
| scaffold_1 | 124095 | 124786 | + |
| scaffold_1 | 126192 | 126225 | + |
| scaffold_1 | 126347 | 130701 | + |
| scaffold_1 | 130817 | 131798 | + |
| scaffold_1 | 1319737 | 1319749 | + |
| scaffold_1 | 132029 | 132886 | + |
| scaffold_1 | 1321994 | 1321996 | + |
| scaffold_1 | 132984 | 133157 | + |
| scaffold_1 | 134207 | 134648 | + |
| scaffold_1 | 134889 | 135379 | + |
| scaffold_1 | 135449 | 136000 | + |
| scaffold_1 | 136118 | 136428 | + |
| scaffold_1 | 136783 | 137851 | + |
| scaffold_1 | 138754 | 139979 | + |
| scaffold_1 | 1396720 | 1396727 | + |
| scaffold_1 | 1396860 | 1396866 | + |
| scaffold_1 | 140367 | 142499 | + |
| scaffold_1 | 142610 | 142964 | + |
| scaffold_1 | 143089 | 144287 | + |
| scaffold_1 | 144389 | 144972 | + |
| scaffold_1 | 145039 | 145308 | + |
| scaffold_1 | 145536 | 146194 | + |
| scaffold_1 | 146358 | 150040 | + |
| scaffold_1 | 1477301 | 1477304 | + |
| scaffold_1 | 1477315 | 1477332 | + |
| scaffold_1 | 150388 | 151119 | + |
| scaffold_1 | 151156 | 151490 | + |
| scaffold_1 | 151541 | 152471 | + |
| scaffold_1 | 1516848 | 1516853 | + |
| scaffold_1 | 152811 | 152821 | + |
| scaffold_1 | 1547566 | 1547569 | + |
| scaffold_1 | 15615 | 15615 | + |
| scaffold_1 | 158143 | 158315 | + |
| scaffold_1 | 158377 | 164198 | + |
| scaffold_1 | 1602370 | 1602384 | + |
| scaffold_1 | 164265 | 164456 | + |
| scaffold_1 | 165460 | 165528 | + |
| scaffold_1 | 166283 | 166308 | + |
| scaffold_1 | 168189 | 168409 | + |
| scaffold_1 | 1700613 | 1700613 | + |
| scaffold_1 | 172211 | 172213 | + |
| scaffold_1 | 1722746 | 1722746 | + |
| scaffold_1 | 1748372 | 1748375 | + |
| scaffold_1 | 1750918 | 1750922 | + |
| scaffold_1 | 1805907 | 1805920 | + |
| scaffold_1 | 1805953 | 1805958 | + |
| scaffold_1 | 1839732 | 1839733 | + |
| scaffold_1 | 1840203 | 1840211 | + |
| scaffold_1 | 1927825 | 1927832 | + |
| scaffold_1 | 1930136 | 1930136 | + |
| scaffold_1 | 2063405 | 2063410 | + |
| scaffold_1 | 2065814 | 2065816 | + |
| scaffold_1 | 2166808 | 2166817 | + |
| scaffold_1 | 2174218 | 2174226 | + |
| scaffold_1 | 2249852 | 2249854 | + |
| scaffold_1 | 2464627 | 2464627 | + |
| scaffold_1 | 2504807 | 2504809 | + |
| scaffold_1 | 2504820 | 2504828 | + |
| scaffold_1 | 2588563 | 2588568 | + |
| scaffold_1 | 2768018 | 2768028 | + |
| scaffold_1 | 2929356 | 2929356 | + |
| scaffold_1 | 2992643 | 2992662 | + |
| scaffold_1 | 3029379 | 3029382 | + |
| scaffold_1 | 3044466 | 3044471 | + |
| scaffold_1 | 3071400 | 3071403 | + |
| scaffold_1 | 3088502 | 3088510 | + |
| scaffold_1 | 3157078 | 3157098 | + |
| scaffold_1 | 3179452 | 3179453 | + |
| scaffold_1 | 3219419 | 3219454 | + |
| scaffold_1 | 3229473 | 3229473 | + |
| scaffold_1 | 3229555 | 3229562 | + |
| scaffold_1 | 3245683 | 3245687 | + |
| scaffold_1 | 3245810 | 3245815 | + |
| scaffold_1 | 3260899 | 3260924 | + |
| scaffold_1 | 3273556 | 3273559 | + |
| scaffold_1 | 3462161 | 3462164 | + |
| scaffold_1 | 3503580 | 3503603 | + |
| scaffold_1 | 413700 | 413700 | + |
| scaffold_1 | 416576 | 416583 | + |
| scaffold_1 | 422633 | 422640 | + |
| scaffold_1 | 470576 | 470576 | + |
| scaffold_1 | 470607 | 470611 | + |
| scaffold_1 | 476326 | 476329 | + |
| scaffold_1 | 591179 | 591188 | + |
| scaffold_1 | 623123 | 623126 | + |
| scaffold_1 | 652243 | 652356 | + |
| scaffold_1 | 656606 | 656611 | + |
| scaffold_1 | 656831 | 656836 | + |
| scaffold_1 | 659770 | 660054 | + |
| scaffold_1 | 663253 | 663254 | + |
| scaffold_1 | 663726 | 663736 | + |
| scaffold_1 | 664191 | 664199 | + |
| scaffold_1 | 664573 | 664574 | + |
| scaffold_1 | 687339 | 687344 | + |
| scaffold_1 | 687412 | 687415 | + |
| scaffold_1 | 755877 | 755899 | + |
| scaffold_1 | 755998 | 755998 | + |
| scaffold_1 | 771309 | 771311 | + |
| scaffold_1 | 781339 | 781345 | + |
| scaffold_1 | 80238 | 80241 | + |
| scaffold_1 | 805541 | 805560 | + |
| scaffold_1 | 813597 | 813606 | + |
| scaffold_1 | 813647 | 813651 | + |
| scaffold_1 | 907986 | 907988 | + |
| scaffold_1 | 908028 | 908039 | + |
| scaffold_1 | 911344 | 911349 | + |
| scaffold_1 | 924462 | 924475 | + |
| scaffold_10 | 1000028 | 1000068 | + |
| scaffold_10 | 1007051 | 1007061 | + |
| scaffold_10 | 1009132 | 1009313 | + |
| scaffold_10 | 1021552 | 1021557 | + |
| scaffold_10 | 1034753 | 1034755 | + |
| scaffold_10 | 1034790 | 1034797 | + |
| scaffold_10 | 1097097 | 1097097 | + |
| scaffold_10 | 11909 | 11923 | + |
| scaffold_10 | 1205414 | 1205415 | + |
| scaffold_10 | 1212413 | 1212413 | + |
| scaffold_10 | 12180 | 12356 | + |
| scaffold_10 | 1230062 | 1230064 | + |
| scaffold_10 | 1233145 | 1233152 | + |
| scaffold_10 | 1233409 | 1233411 | + |
| scaffold_10 | 1269475 | 1269475 | + |
| scaffold_10 | 1269635 | 1269643 | + |
| scaffold_10 | 1311468 | 1311483 | + |
| scaffold_10 | 1339360 | 1339388 | + |
| scaffold_10 | 1360600 | 1361576 | + |
| scaffold_10 | 1364178 | 1364181 | + |
| scaffold_10 | 137576 | 137577 | + |
| scaffold_10 | 137629 | 137631 | + |
| scaffold_10 | 1426017 | 1426170 | + |
| scaffold_10 | 1475027 | 1475028 | + |
| scaffold_10 | 1479934 | 1480029 | + |
| scaffold_10 | 1481007 | 1482059 | + |
| scaffold_10 | 1482566 | 1482926 | + |
| scaffold_10 | 1482959 | 1483106 | + |
| scaffold_10 | 149828 | 149832 | + |
| scaffold_10 | 1514214 | 1514217 | + |
| scaffold_10 | 1516421 | 1516534 | + |
| scaffold_10 | 1521288 | 1521289 | + |
| scaffold_10 | 1521756 | 1521757 | + |
| scaffold_10 | 1522224 | 1522225 | + |
| scaffold_10 | 1522692 | 1522693 | + |
| scaffold_10 | 1523160 | 1523161 | + |
| scaffold_10 | 1523628 | 1523629 | + |
| scaffold_10 | 1524096 | 1524097 | + |
| scaffold_10 | 1524564 | 1524565 | + |
| scaffold_10 | 1525032 | 1525033 | + |
| scaffold_10 | 1525500 | 1525501 | + |
| scaffold_10 | 1525968 | 1525969 | + |
| scaffold_10 | 1526436 | 1526437 | + |
| scaffold_10 | 1526904 | 1526905 | + |
| scaffold_10 | 164481 | 164515 | + |
| scaffold_10 | 181653 | 181718 | + |
| scaffold_10 | 2724 | 3126 | + |
| scaffold_10 | 290836 | 290846 | + |
| scaffold_10 | 321870 | 321876 | + |
| scaffold_10 | 333087 | 333091 | + |
| scaffold_10 | 3397 | 3709 | + |
| scaffold_10 | 3913 | 3950 | + |
| scaffold_10 | 3995 | 4258 | + |
| scaffold_10 | 453080 | 453122 | + |
| scaffold_10 | 576281 | 576289 | + |
| scaffold_10 | 58283 | 58287 | + |
| scaffold_10 | 709285 | 709291 | + |
| scaffold_10 | 721670 | 721673 | + |
| scaffold_10 | 721744 | 721749 | + |
| scaffold_10 | 83308 | 83310 | + |
| scaffold_10 | 897019 | 897025 | + |
| scaffold_10 | 9109 | 9109 | + |
| scaffold_10 | 914540 | 914551 | + |
| scaffold_10 | 9210 | 9221 | + |
| scaffold_10 | 92789 | 92796 | + |
| scaffold_10 | 937575 | 937577 | + |
| scaffold_10 | 990820 | 990821 | + |
| scaffold_10 | 991288 | 991289 | + |
| scaffold_10 | 991443 | 991482 | + |
| scaffold_100 | 13736 | 15412 | + |
| scaffold_100 | 15761 | 15830 | + |
| scaffold_100 | 16082 | 16226 | + |
| scaffold_100 | 1697 | 5523 | + |
| scaffold_100 | 17990 | 18924 | + |
| scaffold_100 | 25164 | 25480 | + |
| scaffold_100 | 25723 | 25939 | + |
| scaffold_100 | 261 | 371 | + |
| scaffold_100 | 26827 | 27795 | + |
| scaffold_100 | 5566 | 5677 | + |
| scaffold_100 | 564 | 1069 | + |
| scaffold_100 | 5800 | 7311 | + |
| scaffold_100 | 7434 | 7545 | + |
| scaffold_100 | 7588 | 13016 | + |
| scaffold_100 | 86 | 187 | + |
| scaffold_102 | 10671 | 10754 | + |
| scaffold_102 | 10871 | 10925 | + |
| scaffold_102 | 11031 | 11477 | + |
| scaffold_102 | 11547 | 11927 | + |
| scaffold_102 | 12018 | 12033 | + |
| scaffold_102 | 12144 | 12147 | + |
| scaffold_102 | 1266 | 3080 | + |
| scaffold_102 | 12904 | 13046 | + |
| scaffold_102 | 13098 | 13111 | + |
| scaffold_102 | 13563 | 13997 | + |
| scaffold_102 | 14268 | 14433 | + |
| scaffold_102 | 14482 | 16977 | + |
| scaffold_102 | 17017 | 17101 | + |
| scaffold_102 | 17255 | 18368 | + |
| scaffold_102 | 18795 | 20002 | + |
| scaffold_102 | 20826 | 20832 | + |
| scaffold_102 | 21031 | 21139 | + |
| scaffold_102 | 21195 | 21279 | + |
| scaffold_102 | 21997 | 22743 | + |
| scaffold_102 | 22789 | 23096 | + |
| scaffold_102 | 23378 | 23721 | + |
| scaffold_102 | 24232 | 24589 | + |
| scaffold_102 | 24979 | 25232 | + |
| scaffold_102 | 265 | 359 | + |
| scaffold_102 | 510 | 1176 | + |
| scaffold_102 | 5169 | 5169 | + |
| scaffold_102 | 5461 | 5768 | + |
| scaffold_102 | 5830 | 7242 | + |
| scaffold_102 | 60 | 79 | + |
| scaffold_102 | 7273 | 7388 | + |
| scaffold_102 | 7434 | 7456 | + |
| scaffold_102 | 7573 | 8807 | + |
| scaffold_102 | 8856 | 8945 | + |
| scaffold_102 | 9071 | 10605 | + |
| scaffold_103 | 10517 | 10611 | + |
| scaffold_103 | 10765 | 12565 | + |
| scaffold_103 | 13563 | 15393 | + |
| scaffold_103 | 15488 | 16018 | + |
| scaffold_103 | 17884 | 17969 | + |
| scaffold_103 | 18179 | 19063 | + |
| scaffold_103 | 19166 | 19449 | + |
| scaffold_103 | 19637 | 19953 | + |
| scaffold_103 | 1977 | 1978 | + |
| scaffold_103 | 20032 | 20865 | + |
| scaffold_103 | 21064 | 21538 | + |
| scaffold_103 | 21638 | 23479 | + |
| scaffold_103 | 2178 | 6692 | + |
| scaffold_103 | 6726 | 7547 | + |
| scaffold_103 | 7811 | 10456 | + |
| scaffold_105 | 10614 | 11228 | + |
| scaffold_105 | 12150 | 13164 | + |
| scaffold_105 | 13418 | 14296 | + |
| scaffold_105 | 14422 | 14569 | + |
| scaffold_105 | 14642 | 15170 | + |
| scaffold_105 | 15230 | 15814 | + |
| scaffold_105 | 16280 | 16360 | + |
| scaffold_105 | 18194 | 18487 | + |
| scaffold_105 | 18535 | 18555 | + |
| scaffold_105 | 18969 | 19263 | + |
| scaffold_105 | 19347 | 19605 | + |
| scaffold_105 | 20027 | 20609 | + |
| scaffold_105 | 20681 | 21470 | + |
| scaffold_105 | 6442 | 6443 | + |
| scaffold_106 | 14409 | 14707 | + |
| scaffold_106 | 759 | 759 | + |
| scaffold_107 | 9939 | 19787 | + |
| scaffold_108 | 1024 | 1024 | + |
| scaffold_108 | 1094 | 6487 | + |
| scaffold_108 | 17660 | 17695 | + |
| scaffold_108 | 18546 | 18783 | + |
| scaffold_108 | 6584 | 7523 | + |
| scaffold_108 | 7650 | 7684 | + |
| scaffold_109 | 10134 | 10853 | + |
| scaffold_109 | 10993 | 11158 | + |
| scaffold_109 | 12679 | 12853 | + |
| scaffold_109 | 13029 | 13072 | + |
| scaffold_109 | 13436 | 13794 | + |
| scaffold_109 | 14214 | 14217 | + |
| scaffold_109 | 14677 | 15404 | + |
| scaffold_109 | 15520 | 15532 | + |
| scaffold_109 | 15614 | 15726 | + |
| scaffold_109 | 15876 | 16396 | + |
| scaffold_109 | 16587 | 17334 | + |
| scaffold_109 | 17553 | 18256 | + |
| scaffold_109 | 180 | 557 | + |
| scaffold_109 | 18337 | 18440 | + |
| scaffold_109 | 2311 | 3208 | + |
| scaffold_109 | 3451 | 3667 | + |
| scaffold_109 | 3916 | 7078 | + |
| scaffold_109 | 57 | 131 | + |
| scaffold_109 | 606 | 1810 | + |
| scaffold_109 | 7165 | 7190 | + |
| scaffold_109 | 7255 | 8679 | + |
| scaffold_11 | 1194603 | 1194610 | + |
| scaffold_11 | 1194697 | 1194699 | + |
| scaffold_11 | 119587 | 119587 | + |
| scaffold_11 | 119724 | 119726 | + |
| scaffold_11 | 1373286 | 1373293 | + |
| scaffold_11 | 1373434 | 1373442 | + |
| scaffold_11 | 186843 | 186843 | + |
| scaffold_11 | 288999 | 289002 | + |
| scaffold_11 | 289024 | 289024 | + |
| scaffold_11 | 322365 | 322365 | + |
| scaffold_11 | 322496 | 322505 | + |
| scaffold_11 | 394492 | 394494 | + |
| scaffold_11 | 425147 | 425605 | + |
| scaffold_11 | 425666 | 430160 | + |
| scaffold_11 | 434788 | 434789 | + |
| scaffold_11 | 439601 | 439601 | + |
| scaffold_11 | 439811 | 439817 | + |
| scaffold_11 | 447384 | 447385 | + |
| scaffold_11 | 447530 | 447533 | + |
| scaffold_11 | 451929 | 451932 | + |
| scaffold_11 | 454533 | 454536 | + |
| scaffold_11 | 487588 | 487591 | + |
| scaffold_11 | 517151 | 517153 | + |
| scaffold_11 | 543571 | 543576 | + |
| scaffold_11 | 544061 | 544063 | + |
| scaffold_11 | 546242 | 546248 | + |
| scaffold_11 | 546429 | 546435 | + |
| scaffold_11 | 623287 | 623297 | + |
| scaffold_11 | 623340 | 623346 | + |
| scaffold_11 | 637049 | 637052 | + |
| scaffold_11 | 638845 | 638848 | + |
| scaffold_11 | 644555 | 644558 | + |
| scaffold_11 | 765565 | 765565 | + |
| scaffold_11 | 768964 | 768964 | + |
| scaffold_11 | 936262 | 936265 | + |
| scaffold_11 | 94632 | 94633 | + |
| scaffold_110 | 58 | 59 | + |
| scaffold_110 | 8368 | 8371 | + |
| scaffold_112 | 10379 | 10466 | + |
| scaffold_112 | 11752 | 12164 | + |
| scaffold_112 | 1554 | 1915 | + |
| scaffold_112 | 15615 | 16115 | + |
| scaffold_112 | 16366 | 16511 | + |
| scaffold_112 | 16727 | 16809 | + |
| scaffold_112 | 3785 | 4565 | + |
| scaffold_112 | 4817 | 5482 | + |
| scaffold_112 | 60 | 1417 | + |
| scaffold_112 | 6643 | 10299 | + |
| scaffold_113 | 1 | 9 | + |
| scaffold_113 | 8728 | 8730 | + |
| scaffold_114 | 10037 | 10434 | + |
| scaffold_114 | 10865 | 10866 | + |
| scaffold_114 | 1095 | 2869 | + |
| scaffold_114 | 11522 | 11919 | + |
| scaffold_114 | 12168 | 12278 | + |
| scaffold_114 | 12335 | 12676 | + |
| scaffold_114 | 13137 | 13861 | + |
| scaffold_114 | 13923 | 13974 | + |
| scaffold_114 | 14031 | 14245 | + |
| scaffold_114 | 14453 | 14700 | + |
| scaffold_114 | 15328 | 15341 | + |
| scaffold_114 | 3826 | 3858 | + |
| scaffold_114 | 3943 | 4124 | + |
| scaffold_114 | 4380 | 4422 | + |
| scaffold_114 | 4678 | 4859 | + |
| scaffold_114 | 4944 | 4978 | + |
| scaffold_114 | 6488 | 6499 | + |
| scaffold_114 | 6678 | 6680 | + |
| scaffold_114 | 7256 | 7503 | + |
| scaffold_114 | 7711 | 7925 | + |
| scaffold_114 | 7982 | 8033 | + |
| scaffold_114 | 8095 | 8819 | + |
| scaffold_114 | 9280 | 9621 | + |
| scaffold_114 | 9678 | 9788 | + |
| scaffold_115 | 16192 | 16298 | + |
| scaffold_116 | 10276 | 10277 | + |
| scaffold_116 | 10744 | 10745 | + |
| scaffold_116 | 11212 | 11213 | + |
| scaffold_116 | 11680 | 11681 | + |
| scaffold_116 | 12148 | 12149 | + |
| scaffold_116 | 12616 | 12617 | + |
| scaffold_116 | 13084 | 13085 | + |
| scaffold_116 | 13552 | 13553 | + |
| scaffold_116 | 14020 | 14021 | + |
| scaffold_116 | 14488 | 14489 | + |
| scaffold_116 | 14956 | 14957 | + |
| scaffold_116 | 15542 | 15543 | + |
| scaffold_116 | 6064 | 6065 | + |
| scaffold_116 | 6532 | 6533 | + |
| scaffold_116 | 7000 | 7001 | + |
| scaffold_116 | 7468 | 7469 | + |
| scaffold_116 | 7936 | 7937 | + |
| scaffold_116 | 8404 | 8405 | + |
| scaffold_116 | 8872 | 8873 | + |
| scaffold_116 | 9340 | 9341 | + |
| scaffold_116 | 9808 | 9809 | + |
| scaffold_117 | 9947 | 11152 | + |
| scaffold_118 | 644 | 647 | + |
| scaffold_12 | 102274 | 102274 | + |
| scaffold_12 | 116920 | 117452 | + |
| scaffold_12 | 117569 | 119769 | + |
| scaffold_12 | 119875 | 120624 | + |
| scaffold_12 | 120777 | 120789 | + |
| scaffold_12 | 12192 | 12193 | + |
| scaffold_12 | 138274 | 138286 | + |
| scaffold_12 | 180446 | 180465 | + |
| scaffold_12 | 181244 | 181245 | + |
| scaffold_12 | 206549 | 206578 | + |
| scaffold_12 | 269997 | 270018 | + |
| scaffold_12 | 295039 | 295131 | + |
| scaffold_12 | 303526 | 308168 | + |
| scaffold_12 | 308267 | 308935 | + |
| scaffold_12 | 309392 | 310516 | + |
| scaffold_12 | 318249 | 319344 | + |
| scaffold_12 | 373210 | 373210 | + |
| scaffold_12 | 466175 | 466187 | + |
| scaffold_12 | 476579 | 479100 | + |
| scaffold_12 | 540357 | 540358 | + |
| scaffold_12 | 540426 | 540429 | + |
| scaffold_12 | 739288 | 739289 | + |
| scaffold_12 | 763709 | 763710 | + |
| scaffold_12 | 766011 | 766012 | + |
| scaffold_12 | 784145 | 784148 | + |
| scaffold_12 | 784163 | 784163 | + |
| scaffold_12 | 85948 | 85975 | + |
| scaffold_12 | 86282 | 86766 | + |
| scaffold_12 | 867398 | 867401 | + |
| scaffold_12 | 867560 | 867562 | + |
| scaffold_12 | 921657 | 921658 | + |
| scaffold_120 | 10961 | 12670 | + |
| scaffold_120 | 13291 | 13291 | + |
| scaffold_120 | 13726 | 14188 | + |
| scaffold_120 | 14251 | 14273 | + |
| scaffold_120 | 14344 | 14382 | + |
| scaffold_120 | 14419 | 14469 | + |
| scaffold_120 | 6996 | 10086 | + |
| scaffold_121 | 12027 | 14187 | + |
| scaffold_122 | 1 | 290 | + |
| scaffold_122 | 10630 | 11035 | + |
| scaffold_122 | 12203 | 12645 | + |
| scaffold_122 | 12789 | 12888 | + |
| scaffold_122 | 13928 | 14009 | + |
| scaffold_122 | 4239 | 5522 | + |
| scaffold_122 | 8498 | 9051 | + |
| scaffold_122 | 9216 | 9263 | + |
| scaffold_122 | 9741 | 9759 | + |
| scaffold_122 | 9847 | 9932 | + |
| scaffold_123 | 2544 | 2545 | + |
| scaffold_123 | 2940 | 3094 | + |
| scaffold_123 | 3230 | 3830 | + |
| scaffold_123 | 4299 | 4299 | + |
| scaffold_124 | 10879 | 11008 | + |
| scaffold_124 | 11081 | 11260 | + |
| scaffold_124 | 11383 | 12131 | + |
| scaffold_124 | 117 | 473 | + |
| scaffold_124 | 5754 | 5758 | + |
| scaffold_124 | 8654 | 8952 | + |
| scaffold_124 | 9547 | 10702 | + |
| scaffold_125 | 1 | 76 | + |
| scaffold_125 | 10573 | 10594 | + |
| scaffold_125 | 1954 | 2107 | + |
| scaffold_125 | 3013 | 3073 | + |
| scaffold_125 | 4091 | 4122 | + |
| scaffold_125 | 5652 | 5683 | + |
| scaffold_127 | 1 | 4 | + |
| scaffold_127 | 10616 | 10738 | + |
| scaffold_127 | 10806 | 10818 | + |
| scaffold_127 | 10942 | 10953 | + |
| scaffold_127 | 1618 | 1876 | + |
| scaffold_127 | 3538 | 3651 | + |
| scaffold_127 | 3753 | 6846 | + |
| scaffold_13 | 1027934 | 1027997 | + |
| scaffold_13 | 1029293 | 1029392 | + |
| scaffold_13 | 103994 | 103996 | + |
| scaffold_13 | 156117 | 156118 | + |
| scaffold_13 | 1599 | 2475 | + |
| scaffold_13 | 299016 | 299017 | + |
| scaffold_13 | 375267 | 375276 | + |
| scaffold_13 | 377036 | 377044 | + |
| scaffold_13 | 459837 | 459841 | + |
| scaffold_13 | 503134 | 503140 | + |
| scaffold_13 | 503413 | 503415 | + |
| scaffold_13 | 505150 | 505158 | + |
| scaffold_13 | 505259 | 505259 | + |
| scaffold_13 | 506320 | 506322 | + |
| scaffold_13 | 532039 | 532047 | + |
| scaffold_13 | 532153 | 532777 | + |
| scaffold_13 | 532846 | 533350 | + |
| scaffold_13 | 534987 | 534988 | + |
| scaffold_13 | 535455 | 535456 | + |
| scaffold_13 | 535923 | 535924 | + |
| scaffold_13 | 536391 | 536392 | + |
| scaffold_13 | 536859 | 536860 | + |
| scaffold_13 | 537327 | 537328 | + |
| scaffold_13 | 537795 | 537796 | + |
| scaffold_13 | 538263 | 538264 | + |
| scaffold_13 | 538731 | 538732 | + |
| scaffold_13 | 539199 | 539200 | + |
| scaffold_13 | 539354 | 539415 | + |
| scaffold_13 | 540490 | 540990 | + |
| scaffold_13 | 548098 | 553134 | + |
| scaffold_13 | 556077 | 556097 | + |
| scaffold_13 | 568833 | 568839 | + |
| scaffold_13 | 568876 | 568876 | + |
| scaffold_13 | 613036 | 613105 | + |
| scaffold_13 | 614408 | 614409 | + |
| scaffold_13 | 619859 | 619876 | + |
| scaffold_13 | 643925 | 643928 | + |
| scaffold_13 | 693193 | 693199 | + |
| scaffold_13 | 717922 | 717924 | + |
| scaffold_13 | 772074 | 772076 | + |
| scaffold_13 | 786966 | 786967 | + |
| scaffold_13 | 820796 | 820798 | + |
| scaffold_13 | 820936 | 820938 | + |
| scaffold_13 | 865989 | 865997 | + |
| scaffold_13 | 866086 | 866140 | + |
| scaffold_13 | 934108 | 934111 | + |
| scaffold_13 | 990549 | 990551 | + |
| scaffold_130 | 1 | 315 | + |
| scaffold_130 | 10156 | 10582 | + |
| scaffold_130 | 10760 | 10807 | + |
| scaffold_130 | 10996 | 10999 | + |
| scaffold_130 | 1246 | 1246 | + |
| scaffold_130 | 1631 | 1792 | + |
| scaffold_130 | 3247 | 4027 | + |
| scaffold_130 | 377 | 684 | + |
| scaffold_130 | 4245 | 4304 | + |
| scaffold_130 | 4774 | 4823 | + |
| scaffold_130 | 5423 | 5808 | + |
| scaffold_130 | 7588 | 8062 | + |
| scaffold_130 | 8238 | 8349 | + |
| scaffold_130 | 8868 | 8931 | + |
| scaffold_131 | 5558 | 5579 | + |
| scaffold_133 | 1 | 201 | + |
| scaffold_133 | 10278 | 10293 | + |
| scaffold_133 | 1287 | 1291 | + |
| scaffold_133 | 2058 | 2058 | + |
| scaffold_133 | 2309 | 2599 | + |
| scaffold_133 | 3639 | 3738 | + |
| scaffold_133 | 3882 | 4324 | + |
| scaffold_133 | 5507 | 5912 | + |
| scaffold_133 | 6607 | 6692 | + |
| scaffold_133 | 6780 | 6798 | + |
| scaffold_133 | 7276 | 7323 | + |
| scaffold_133 | 741 | 834 | + |
| scaffold_133 | 7488 | 7626 | + |
| scaffold_133 | 9402 | 9471 | + |
| scaffold_134 | 1981 | 2407 | + |
| scaffold_134 | 2490 | 2507 | + |
| scaffold_134 | 2622 | 2767 | + |
| scaffold_134 | 2979 | 3823 | + |
| scaffold_134 | 5474 | 5815 | + |
| scaffold_134 | 6061 | 7014 | + |
| scaffold_134 | 7228 | 7318 | + |
| scaffold_134 | 7402 | 7918 | + |
| scaffold_134 | 8028 | 10749 | + |
| scaffold_135 | 10307 | 10568 | + |
| scaffold_135 | 1972 | 1974 | + |
| scaffold_135 | 2273 | 2506 | + |
| scaffold_135 | 258 | 258 | + |
| scaffold_135 | 3084 | 3087 | + |
| scaffold_135 | 3127 | 3475 | + |
| scaffold_135 | 423 | 458 | + |
| scaffold_135 | 582 | 597 | + |
| scaffold_135 | 7818 | 10251 | + |
| scaffold_138 | 1331 | 1333 | + |
| scaffold_138 | 1381 | 1428 | + |
| scaffold_138 | 1922 | 1927 | + |
| scaffold_138 | 2160 | 2169 | + |
| scaffold_138 | 2354 | 2383 | + |
| scaffold_138 | 2568 | 2700 | + |
| scaffold_138 | 2918 | 3169 | + |
| scaffold_138 | 3422 | 3434 | + |
| scaffold_138 | 3749 | 4527 | + |
| scaffold_138 | 4701 | 5072 | + |
| scaffold_138 | 5122 | 5170 | + |
| scaffold_138 | 5504 | 5764 | + |
| scaffold_138 | 988 | 1134 | + |
| scaffold_139 | 1 | 239 | + |
| scaffold_139 | 2363 | 2495 | + |
| scaffold_139 | 2558 | 3508 | + |
| scaffold_139 | 293 | 2097 | + |
| scaffold_139 | 3549 | 4196 | + |
| scaffold_139 | 5202 | 5223 | + |
| scaffold_139 | 5379 | 8843 | + |
| scaffold_139 | 9074 | 9205 | + |
| scaffold_139 | 9261 | 9421 | + |
| scaffold_139 | 9479 | 9618 | + |
| scaffold_139 | 9679 | 9679 | + |
| scaffold_139 | 9774 | 9827 | + |
| scaffold_14 | 105214 | 105216 | + |
| scaffold_14 | 12782 | 12784 | + |
| scaffold_14 | 14225 | 14235 | + |
| scaffold_14 | 148691 | 148692 | + |
| scaffold_14 | 220124 | 220835 | + |
| scaffold_14 | 246937 | 247020 | + |
| scaffold_14 | 247714 | 248292 | + |
| scaffold_14 | 248564 | 249111 | + |
| scaffold_14 | 250315 | 251020 | + |
| scaffold_14 | 251359 | 251393 | + |
| scaffold_14 | 252284 | 252420 | + |
| scaffold_14 | 258850 | 259039 | + |
| scaffold_14 | 259763 | 259772 | + |
| scaffold_14 | 260553 | 261244 | + |
| scaffold_14 | 261275 | 261784 | + |
| scaffold_14 | 261961 | 262052 | + |
| scaffold_14 | 262342 | 262700 | + |
| scaffold_14 | 262786 | 262937 | + |
| scaffold_14 | 263194 | 263307 | + |
| scaffold_14 | 263387 | 263457 | + |
| scaffold_14 | 263545 | 264171 | + |
| scaffold_14 | 264467 | 264817 | + |
| scaffold_14 | 264954 | 265270 | + |
| scaffold_14 | 265999 | 266099 | + |
| scaffold_14 | 275613 | 275961 | + |
| scaffold_14 | 276001 | 276004 | + |
| scaffold_14 | 277988 | 277995 | + |
| scaffold_14 | 279288 | 279300 | + |
| scaffold_14 | 282337 | 282629 | + |
| scaffold_14 | 282669 | 282672 | + |
| scaffold_14 | 283166 | 283193 | + |
| scaffold_14 | 283500 | 283992 | + |
| scaffold_14 | 284254 | 284769 | + |
| scaffold_14 | 284883 | 285346 | + |
| scaffold_14 | 285386 | 285813 | + |
| scaffold_14 | 286057 | 286063 | + |
| scaffold_14 | 286179 | 286205 | + |
| scaffold_14 | 286256 | 286648 | + |
| scaffold_14 | 287130 | 288065 | + |
| scaffold_14 | 288217 | 290457 | + |
| scaffold_14 | 290646 | 292592 | + |
| scaffold_14 | 293093 | 293454 | + |
| scaffold_14 | 293486 | 293690 | + |
| scaffold_14 | 299843 | 299888 | + |
| scaffold_14 | 300506 | 300878 | + |
| scaffold_14 | 301525 | 301833 | + |
| scaffold_14 | 302392 | 302499 | + |
| scaffold_14 | 302547 | 302606 | + |
| scaffold_14 | 302903 | 304434 | + |
| scaffold_14 | 304520 | 304606 | + |
| scaffold_14 | 304860 | 306056 | + |
| scaffold_14 | 306470 | 306697 | + |
| scaffold_14 | 306768 | 306918 | + |
| scaffold_14 | 307117 | 307581 | + |
| scaffold_14 | 307716 | 307900 | + |
| scaffold_14 | 307962 | 308136 | + |
| scaffold_14 | 308338 | 308757 | + |
| scaffold_14 | 309005 | 310741 | + |
| scaffold_14 | 310788 | 315951 | + |
| scaffold_14 | 316009 | 316976 | + |
| scaffold_14 | 317107 | 323859 | + |
| scaffold_14 | 324381 | 324597 | + |
| scaffold_14 | 324734 | 328182 | + |
| scaffold_14 | 328404 | 330165 | + |
| scaffold_14 | 330279 | 330339 | + |
| scaffold_14 | 330371 | 331070 | + |
| scaffold_14 | 331170 | 332305 | + |
| scaffold_14 | 332464 | 332471 | + |
| scaffold_14 | 332534 | 332554 | + |
| scaffold_14 | 358267 | 358267 | + |
| scaffold_14 | 449807 | 449810 | + |
| scaffold_14 | 635047 | 635060 | + |
| scaffold_14 | 639172 | 639395 | + |
| scaffold_14 | 664904 | 664907 | + |
| scaffold_14 | 722125 | 722217 | + |
| scaffold_14 | 763660 | 763763 | + |
| scaffold_14 | 777768 | 782299 | + |
| scaffold_14 | 815497 | 816174 | + |
| scaffold_14 | 816189 | 816375 | + |
| scaffold_14 | 816476 | 817027 | + |
| scaffold_14 | 916578 | 917738 | + |
| scaffold_14 | 919398 | 920227 | + |
| scaffold_14 | 920603 | 920668 | + |
| scaffold_14 | 920759 | 920867 | + |
| scaffold_14 | 920925 | 921011 | + |
| scaffold_14 | 921047 | 922104 | + |
| scaffold_14 | 922262 | 922811 | + |
| scaffold_14 | 924755 | 924756 | + |
| scaffold_14 | 925223 | 925224 | + |
| scaffold_14 | 925691 | 925692 | + |
| scaffold_14 | 926159 | 926160 | + |
| scaffold_14 | 926745 | 926746 | + |
| scaffold_140 | 242 | 616 | + |
| scaffold_140 | 4835 | 4909 | + |
| scaffold_140 | 750 | 752 | + |
| scaffold_142 | 3133 | 3801 | + |
| scaffold_143 | 1 | 343 | + |
| scaffold_143 | 4210 | 4211 | + |
| scaffold_143 | 448 | 491 | + |
| scaffold_143 | 4678 | 4679 | + |
| scaffold_143 | 5146 | 5147 | + |
| scaffold_143 | 5614 | 5615 | + |
| scaffold_143 | 565 | 617 | + |
| scaffold_143 | 6082 | 6083 | + |
| scaffold_143 | 6550 | 6551 | + |
| scaffold_143 | 7018 | 7019 | + |
| scaffold_143 | 7486 | 7487 | + |
| scaffold_143 | 7954 | 7955 | + |
| scaffold_143 | 8282 | 8438 | + |
| scaffold_143 | 8949 | 8971 | + |
| scaffold_145 | 110 | 4174 | + |
| scaffold_145 | 4224 | 4593 | + |
| scaffold_145 | 4632 | 4632 | + |
| scaffold_145 | 4735 | 4949 | + |
| scaffold_145 | 4983 | 5510 | + |
| scaffold_145 | 5590 | 6364 | + |
| scaffold_145 | 6434 | 6481 | + |
| scaffold_145 | 6543 | 6597 | + |
| scaffold_145 | 7223 | 8138 | + |
| scaffold_146 | 1591 | 3458 | + |
| scaffold_146 | 3643 | 3776 | + |
| scaffold_146 | 3875 | 4287 | + |
| scaffold_146 | 4350 | 6116 | + |
| scaffold_146 | 8285 | 8465 | + |
| scaffold_146 | 892 | 893 | + |
| scaffold_148 | 1 | 1316 | + |
| scaffold_148 | 1371 | 5130 | + |
| scaffold_148 | 5490 | 6343 | + |
| scaffold_148 | 6500 | 6518 | + |
| scaffold_148 | 7714 | 7714 | + |
| scaffold_149 | 7628 | 7859 | + |
| scaffold_15 | 214788 | 214793 | + |
| scaffold_15 | 242942 | 242942 | + |
| scaffold_15 | 262150 | 262152 | + |
| scaffold_15 | 262326 | 262327 | + |
| scaffold_15 | 299544 | 299561 | + |
| scaffold_15 | 340375 | 340383 | + |
| scaffold_15 | 389303 | 389304 | + |
| scaffold_15 | 419342 | 419349 | + |
| scaffold_15 | 42294 | 42295 | + |
| scaffold_15 | 42954 | 43177 | + |
| scaffold_15 | 43793 | 43795 | + |
| scaffold_15 | 492098 | 492103 | + |
| scaffold_15 | 508869 | 508872 | + |
| scaffold_15 | 51522 | 51603 | + |
| scaffold_15 | 51680 | 52000 | + |
| scaffold_15 | 52055 | 52197 | + |
| scaffold_15 | 52271 | 52473 | + |
| scaffold_15 | 57882 | 57883 | + |
| scaffold_15 | 58875 | 58885 | + |
| scaffold_15 | 59657 | 59664 | + |
| scaffold_15 | 63039 | 64058 | + |
| scaffold_15 | 648201 | 648208 | + |
| scaffold_15 | 648456 | 648460 | + |
| scaffold_15 | 746187 | 746207 | + |
| scaffold_15 | 773748 | 774443 | + |
| scaffold_15 | 775198 | 775208 | + |
| scaffold_15 | 780506 | 780950 | + |
| scaffold_15 | 782521 | 782524 | + |
| scaffold_15 | 782731 | 782798 | + |
| scaffold_15 | 782868 | 782960 | + |
| scaffold_15 | 783077 | 783577 | + |
| scaffold_15 | 785309 | 785324 | + |
| scaffold_15 | 785573 | 785594 | + |
| scaffold_15 | 785911 | 786063 | + |
| scaffold_15 | 95783 | 95787 | + |
| scaffold_150 | 1136 | 1145 | + |
| scaffold_151 | 4344 | 4359 | + |
| scaffold_153 | 1257 | 1299 | + |
| scaffold_153 | 3777 | 4248 | + |
| scaffold_153 | 5047 | 5110 | + |
| scaffold_153 | 61 | 938 | + |
| scaffold_153 | 6341 | 6763 | + |
| scaffold_153 | 6960 | 7065 | + |
| scaffold_154 | 3260 | 3300 | + |
| scaffold_154 | 3684 | 3742 | + |
| scaffold_154 | 3872 | 3968 | + |
| scaffold_154 | 4086 | 6136 | + |
| scaffold_155 | 1 | 950 | + |
| scaffold_155 | 1142 | 1164 | + |
| scaffold_155 | 1281 | 4225 | + |
| scaffold_155 | 4755 | 4784 | + |
| scaffold_155 | 5410 | 6695 | + |
| scaffold_155 | 980 | 1096 | + |
| scaffold_156 | 1 | 2823 | + |
| scaffold_156 | 3275 | 4201 | + |
| scaffold_156 | 4346 | 4600 | + |
| scaffold_156 | 4780 | 5039 | + |
| scaffold_156 | 5384 | 5388 | + |
| scaffold_157 | 3341 | 5047 | + |
| scaffold_158 | 5956 | 5974 | + |
| scaffold_159 | 1169 | 1172 | + |
| scaffold_159 | 1183 | 1773 | + |
| scaffold_159 | 2219 | 2447 | + |
| scaffold_159 | 2531 | 2810 | + |
| scaffold_159 | 4304 | 4394 | + |
| scaffold_159 | 4844 | 5847 | + |
| scaffold_159 | 57 | 1124 | + |
| scaffold_159 | 5916 | 6219 | + |
| scaffold_159 | 6312 | 6389 | + |
| scaffold_16 | 161483 | 161483 | + |
| scaffold_16 | 224052 | 224057 | + |
| scaffold_16 | 313883 | 313886 | + |
| scaffold_16 | 411168 | 411168 | + |
| scaffold_16 | 449037 | 449037 | + |
| scaffold_16 | 542117 | 542118 | + |
| scaffold_16 | 544721 | 544725 | + |
| scaffold_16 | 561 | 760 | + |
| scaffold_16 | 614947 | 614962 | + |
| scaffold_16 | 697718 | 697718 | + |
| scaffold_16 | 697759 | 697770 | + |
| scaffold_16 | 700463 | 701000 | + |
| scaffold_16 | 701556 | 702238 | + |
| scaffold_16 | 702682 | 702892 | + |
| scaffold_16 | 703350 | 703365 | + |
| scaffold_16 | 800 | 803 | + |
| scaffold_16 | 93074 | 93074 | + |
| scaffold_160 | 1 | 248 | + |
| scaffold_160 | 1060 | 1254 | + |
| scaffold_160 | 1604 | 2529 | + |
| scaffold_160 | 2820 | 2912 | + |
| scaffold_160 | 3011 | 3331 | + |
| scaffold_160 | 330 | 429 | + |
| scaffold_160 | 4210 | 4211 | + |
| scaffold_160 | 653 | 983 | + |
| scaffold_161 | 4004 | 4205 | + |
| scaffold_162 | 1 | 3462 | + |
| scaffold_162 | 3562 | 5227 | + |
| scaffold_162 | 6126 | 6169 | + |
| scaffold_163 | 1 | 82 | + |
| scaffold_163 | 1062 | 1430 | + |
| scaffold_163 | 1822 | 1845 | + |
| scaffold_163 | 1931 | 2064 | + |
| scaffold_163 | 2163 | 2411 | + |
| scaffold_163 | 2667 | 2848 | + |
| scaffold_163 | 2933 | 2965 | + |
| scaffold_163 | 4479 | 4490 | + |
| scaffold_163 | 4582 | 4685 | + |
| scaffold_163 | 4759 | 4830 | + |
| scaffold_163 | 4958 | 5271 | + |
| scaffold_163 | 5405 | 5711 | + |
| scaffold_163 | 6057 | 6152 | + |
| scaffold_165 | 1 | 165 | + |
| scaffold_165 | 1389 | 1481 | + |
| scaffold_165 | 1753 | 1764 | + |
| scaffold_165 | 1978 | 2047 | + |
| scaffold_165 | 2237 | 2506 | + |
| scaffold_165 | 2571 | 2750 | + |
| scaffold_165 | 3036 | 3109 | + |
| scaffold_165 | 3706 | 5140 | + |
| scaffold_165 | 6042 | 6078 | + |
| scaffold_165 | 932 | 936 | + |
| scaffold_166 | 4676 | 4824 | + |
| scaffold_166 | 5546 | 5554 | + |
| scaffold_166 | 5845 | 5999 | + |
| scaffold_168 | 3179 | 3194 | + |
| scaffold_168 | 3318 | 3353 | + |
| scaffold_168 | 5957 | 5964 | + |
| scaffold_169 | 159 | 190 | + |
| scaffold_169 | 267 | 710 | + |
| scaffold_169 | 3622 | 3623 | + |
| scaffold_169 | 5825 | 5825 | + |
| scaffold_169 | 758 | 1288 | + |
| scaffold_17 | 101892 | 101897 | + |
| scaffold_17 | 10791 | 10791 | + |
| scaffold_17 | 124014 | 124014 | + |
| scaffold_17 | 137275 | 137284 | + |
| scaffold_17 | 137389 | 137398 | + |
| scaffold_17 | 192690 | 192706 | + |
| scaffold_17 | 206841 | 206851 | + |
| scaffold_17 | 256449 | 256449 | + |
| scaffold_17 | 256463 | 256463 | + |
| scaffold_17 | 315901 | 315901 | + |
| scaffold_17 | 3587 | 3587 | + |
| scaffold_17 | 42065 | 42067 | + |
| scaffold_17 | 421236 | 421637 | + |
| scaffold_17 | 43531 | 43532 | + |
| scaffold_17 | 472607 | 472624 | + |
| scaffold_17 | 472679 | 472682 | + |
| scaffold_17 | 50844 | 50851 | + |
| scaffold_17 | 527671 | 527671 | + |
| scaffold_17 | 564145 | 564155 | + |
| scaffold_17 | 627129 | 627130 | + |
| scaffold_17 | 63128 | 63132 | + |
| scaffold_17 | 631642 | 631642 | + |
| scaffold_17 | 92176 | 92176 | + |
| scaffold_17 | 92212 | 92216 | + |
| scaffold_170 | 1401 | 2835 | + |
| scaffold_170 | 3432 | 3507 | + |
| scaffold_170 | 3791 | 3970 | + |
| scaffold_170 | 4035 | 4304 | + |
| scaffold_170 | 4494 | 4563 | + |
| scaffold_170 | 5431 | 5435 | + |
| scaffold_170 | 5730 | 5759 | + |
| scaffold_171 | 3079 | 5661 | + |
| scaffold_171 | 56 | 3030 | + |
| scaffold_172 | 1 | 5551 | + |
| scaffold_173 | 1654 | 3035 | + |
| scaffold_173 | 3260 | 3347 | + |
| scaffold_173 | 3390 | 4273 | + |
| scaffold_173 | 5014 | 5014 | + |
| scaffold_174 | 1902 | 2162 | + |
| scaffold_174 | 3274 | 3274 | + |
| scaffold_174 | 4175 | 4300 | + |
| scaffold_174 | 4679 | 4680 | + |
| scaffold_174 | 4767 | 4776 | + |
| scaffold_174 | 4815 | 4846 | + |
| scaffold_174 | 4925 | 4931 | + |
| scaffold_174 | 4980 | 5175 | + |
| scaffold_175 | 1698 | 2353 | + |
| scaffold_175 | 2401 | 2539 | + |
| scaffold_175 | 2576 | 2576 | + |
| scaffold_175 | 3068 | 5483 | + |
| scaffold_177 | 1 | 479 | + |
| scaffold_177 | 1319 | 2018 | + |
| scaffold_177 | 930 | 1234 | + |
| scaffold_178 | 3709 | 3709 | + |
| scaffold_18 | 1 | 181 | + |
| scaffold_18 | 11539 | 11728 | + |
| scaffold_18 | 1164 | 1533 | + |
| scaffold_18 | 12749 | 12842 | + |
| scaffold_18 | 13558 | 13575 | + |
| scaffold_18 | 14028 | 14030 | + |
| scaffold_18 | 14099 | 14193 | + |
| scaffold_18 | 14440 | 14580 | + |
| scaffold_18 | 14980 | 15029 | + |
| scaffold_18 | 15964 | 16003 | + |
| scaffold_18 | 192149 | 192152 | + |
| scaffold_18 | 1924 | 2513 | + |
| scaffold_18 | 198300 | 198301 | + |
| scaffold_18 | 218329 | 218421 | + |
| scaffold_18 | 239431 | 239523 | + |
| scaffold_18 | 247047 | 247150 | + |
| scaffold_18 | 250242 | 250334 | + |
| scaffold_18 | 254323 | 254326 | + |
| scaffold_18 | 264282 | 264395 | + |
| scaffold_18 | 268892 | 268910 | + |
| scaffold_18 | 272639 | 273188 | + |
| scaffold_18 | 276304 | 276311 | + |
| scaffold_18 | 2769 | 2950 | + |
| scaffold_18 | 280474 | 280627 | + |
| scaffold_18 | 3035 | 3067 | + |
| scaffold_18 | 338506 | 338513 | + |
| scaffold_18 | 364217 | 364229 | + |
| scaffold_18 | 367573 | 367805 | + |
| scaffold_18 | 378394 | 378471 | + |
| scaffold_18 | 378625 | 378626 | + |
| scaffold_18 | 379093 | 379094 | + |
| scaffold_18 | 402057 | 404489 | + |
| scaffold_18 | 404951 | 406997 | + |
| scaffold_18 | 411902 | 411902 | + |
| scaffold_18 | 4579 | 4590 | + |
| scaffold_18 | 463446 | 463734 | + |
| scaffold_18 | 467216 | 467290 | + |
| scaffold_18 | 477769 | 477785 | + |
| scaffold_18 | 478321 | 478941 | + |
| scaffold_18 | 490337 | 490353 | + |
| scaffold_18 | 492080 | 492089 | + |
| scaffold_18 | 492363 | 492500 | + |
| scaffold_18 | 499598 | 499751 | + |
| scaffold_18 | 507653 | 507654 | + |
| scaffold_18 | 5147 | 5163 | + |
| scaffold_18 | 537610 | 537613 | + |
| scaffold_18 | 537653 | 538001 | + |
| scaffold_18 | 540088 | 540091 | + |
| scaffold_18 | 559277 | 559430 | + |
| scaffold_18 | 561523 | 565095 | + |
| scaffold_18 | 565564 | 567168 | + |
| scaffold_18 | 573918 | 573918 | + |
| scaffold_18 | 573994 | 574002 | + |
| scaffold_18 | 57949 | 57951 | + |
| scaffold_18 | 5797 | 5910 | + |
| scaffold_18 | 590712 | 590804 | + |
| scaffold_18 | 599677 | 599695 | + |
| scaffold_18 | 6044 | 6390 | + |
| scaffold_18 | 614528 | 614895 | + |
| scaffold_18 | 616725 | 616733 | + |
| scaffold_18 | 617247 | 617432 | + |
| scaffold_18 | 6696 | 6765 | + |
| scaffold_18 | 7147 | 7192 | + |
| scaffold_18 | 9506 | 9531 | + |
| scaffold_180 | 4420 | 4836 | + |
| scaffold_180 | 5004 | 5193 | + |
| scaffold_180 | 532 | 617 | + |
| scaffold_181 | 4294 | 4827 | + |
| scaffold_182 | 4587 | 5040 | + |
| scaffold_183 | 1838 | 1839 | + |
| scaffold_183 | 4326 | 5092 | + |
| scaffold_184 | 1237 | 1240 | + |
| scaffold_184 | 1280 | 1628 | + |
| scaffold_186 | 1 | 4206 | + |
| scaffold_186 | 4322 | 4367 | + |
| scaffold_186 | 4467 | 4864 | + |
| scaffold_188 | 1 | 1 | + |
| scaffold_188 | 1238 | 1507 | + |
| scaffold_188 | 1697 | 1764 | + |
| scaffold_188 | 3243 | 3243 | + |
| scaffold_188 | 3494 | 3784 | + |
| scaffold_188 | 4755 | 4784 | + |
| scaffold_188 | 635 | 710 | + |
| scaffold_188 | 994 | 1173 | + |
| scaffold_189 | 1 | 678 | + |
| scaffold_189 | 1247 | 2220 | + |
| scaffold_189 | 2621 | 2646 | + |
| scaffold_189 | 2709 | 2829 | + |
| scaffold_189 | 4332 | 4726 | + |
| scaffold_189 | 790 | 968 | + |
| scaffold_19 | 115442 | 115445 | + |
| scaffold_19 | 120589 | 120591 | + |
| scaffold_19 | 260862 | 260862 | + |
| scaffold_19 | 290954 | 290954 | + |
| scaffold_19 | 343919 | 343929 | + |
| scaffold_19 | 42378 | 42380 | + |
| scaffold_19 | 424613 | 424618 | + |
| scaffold_19 | 424666 | 424670 | + |
| scaffold_19 | 430782 | 430786 | + |
| scaffold_19 | 487114 | 487115 | + |
| scaffold_19 | 494804 | 494812 | + |
| scaffold_19 | 500785 | 500786 | + |
| scaffold_19 | 52545 | 52545 | + |
| scaffold_19 | 536207 | 536207 | + |
| scaffold_19 | 536455 | 536456 | + |
| scaffold_19 | 536923 | 536924 | + |
| scaffold_19 | 537391 | 537392 | + |
| scaffold_19 | 537859 | 537860 | + |
| scaffold_19 | 538327 | 538328 | + |
| scaffold_19 | 538795 | 538796 | + |
| scaffold_19 | 539263 | 539264 | + |
| scaffold_19 | 539731 | 539732 | + |
| scaffold_192 | 1 | 3380 | + |
| scaffold_192 | 3423 | 3534 | + |
| scaffold_192 | 3657 | 4335 | + |
| scaffold_194 | 1707 | 1785 | + |
| scaffold_194 | 1866 | 2348 | + |
| scaffold_194 | 2405 | 3970 | + |
| scaffold_194 | 990 | 1217 | + |
| scaffold_197 | 1 | 495 | + |
| scaffold_197 | 1243 | 1298 | + |
| scaffold_197 | 1346 | 3512 | + |
| scaffold_197 | 543 | 681 | + |
| scaffold_199 | 1 | 611 | + |
| scaffold_199 | 1105 | 3210 | + |
| scaffold_2 | 1087884 | 1087888 | + |
| scaffold_2 | 1157570 | 1157572 | + |
| scaffold_2 | 1157780 | 1157781 | + |
| scaffold_2 | 1163381 | 1163387 | + |
| scaffold_2 | 1163523 | 1163540 | + |
| scaffold_2 | 1170840 | 1170859 | + |
| scaffold_2 | 1180176 | 1180176 | + |
| scaffold_2 | 1200984 | 1200996 | + |
| scaffold_2 | 1271972 | 1272075 | + |
| scaffold_2 | 1291318 | 1291322 | + |
| scaffold_2 | 1295013 | 1295021 | + |
| scaffold_2 | 131324 | 131326 | + |
| scaffold_2 | 1321553 | 1321553 | + |
| scaffold_2 | 1324299 | 1324304 | + |
| scaffold_2 | 1331738 | 1331738 | + |
| scaffold_2 | 1339280 | 1339372 | + |
| scaffold_2 | 134679 | 134689 | + |
| scaffold_2 | 1384477 | 1384482 | + |
| scaffold_2 | 1388630 | 1388630 | + |
| scaffold_2 | 1416399 | 1416399 | + |
| scaffold_2 | 1416555 | 1416556 | + |
| scaffold_2 | 1456982 | 1456989 | + |
| scaffold_2 | 1465779 | 1465780 | + |
| scaffold_2 | 1496594 | 1496594 | + |
| scaffold_2 | 1496622 | 1496633 | + |
| scaffold_2 | 1584883 | 1584884 | + |
| scaffold_2 | 1584997 | 1585010 | + |
| scaffold_2 | 1650579 | 1650580 | + |
| scaffold_2 | 1719965 | 1719971 | + |
| scaffold_2 | 1719989 | 1719997 | + |
| scaffold_2 | 1738144 | 1738159 | + |
| scaffold_2 | 1738226 | 1738226 | + |
| scaffold_2 | 1788062 | 1788065 | + |
| scaffold_2 | 1788112 | 1788114 | + |
| scaffold_2 | 183052 | 183056 | + |
| scaffold_2 | 183083 | 183084 | + |
| scaffold_2 | 1959303 | 1959303 | + |
| scaffold_2 | 1968061 | 1968066 | + |
| scaffold_2 | 2000915 | 2000915 | + |
| scaffold_2 | 2027456 | 2027465 | + |
| scaffold_2 | 206193 | 206194 | + |
| scaffold_2 | 2077445 | 2077451 | + |
| scaffold_2 | 2077529 | 2077530 | + |
| scaffold_2 | 2126864 | 2126866 | + |
| scaffold_2 | 2167502 | 2167509 | + |
| scaffold_2 | 2201339 | 2201350 | + |
| scaffold_2 | 2293904 | 2293906 | + |
| scaffold_2 | 2404016 | 2404016 | + |
| scaffold_2 | 2483312 | 2483463 | + |
| scaffold_2 | 2533485 | 2533486 | + |
| scaffold_2 | 2549036 | 2549040 | + |
| scaffold_2 | 2634317 | 2634330 | + |
| scaffold_2 | 2671128 | 2674787 | + |
| scaffold_2 | 2681128 | 2683070 | + |
| scaffold_2 | 2683244 | 2684795 | + |
| scaffold_2 | 2685911 | 2686191 | + |
| scaffold_2 | 2686247 | 2686266 | + |
| scaffold_2 | 2723497 | 2723520 | + |
| scaffold_2 | 2768273 | 2768374 | + |
| scaffold_2 | 289716 | 289716 | + |
| scaffold_2 | 496437 | 496441 | + |
| scaffold_2 | 581919 | 581926 | + |
| scaffold_2 | 581996 | 582008 | + |
| scaffold_2 | 692 | 1312 | + |
| scaffold_2 | 730158 | 730162 | + |
| scaffold_2 | 901502 | 901529 | + |
| scaffold_2 | 94 | 472 | + |
| scaffold_2 | 968466 | 968469 | + |
| scaffold_2 | 982955 | 982955 | + |
| scaffold_20 | 124833 | 124841 | + |
| scaffold_20 | 134708 | 134709 | + |
| scaffold_20 | 170013 | 170020 | + |
| scaffold_20 | 180603 | 180603 | + |
| scaffold_20 | 257089 | 257094 | + |
| scaffold_20 | 369825 | 372151 | + |
| scaffold_20 | 527278 | 527281 | + |
| scaffold_201 | 1 | 2858 | + |
| scaffold_201 | 3247 | 3251 | + |
| scaffold_201 | 3298 | 3301 | + |
| scaffold_202 | 1 | 3215 | + |
| scaffold_205 | 1 | 268 | + |
| scaffold_205 | 2190 | 2971 | + |
| scaffold_205 | 382 | 2111 | + |
| scaffold_208 | 1741 | 1762 | + |
| scaffold_208 | 310 | 325 | + |
| scaffold_21 | 126924 | 126932 | + |
| scaffold_21 | 133304 | 133306 | + |
| scaffold_21 | 136373 | 136470 | + |
| scaffold_21 | 1445 | 1455 | + |
| scaffold_21 | 269445 | 269446 | + |
| scaffold_21 | 281480 | 281480 | + |
| scaffold_21 | 288703 | 288706 | + |
| scaffold_21 | 30355 | 30366 | + |
| scaffold_21 | 308380 | 308380 | + |
| scaffold_21 | 312324 | 312324 | + |
| scaffold_21 | 340830 | 340830 | + |
| scaffold_21 | 366873 | 366965 | + |
| scaffold_21 | 385170 | 385181 | + |
| scaffold_21 | 394920 | 394920 | + |
| scaffold_21 | 402409 | 402433 | + |
| scaffold_21 | 464928 | 464938 | + |
| scaffold_21 | 469325 | 469341 | + |
| scaffold_21 | 469880 | 469883 | + |
| scaffold_21 | 479797 | 479900 | + |
| scaffold_21 | 481199 | 481206 | + |
| scaffold_21 | 88569 | 88569 | + |
| scaffold_210 | 2031 | 2242 | + |
| scaffold_210 | 2315 | 2751 | + |
| scaffold_210 | 86 | 1077 | + |
| scaffold_213 | 1 | 112 | + |
| scaffold_213 | 1677 | 2527 | + |
| scaffold_213 | 172 | 587 | + |
| scaffold_213 | 683 | 1521 | + |
| scaffold_214 | 112 | 304 | + |
| scaffold_214 | 1927 | 2046 | + |
| scaffold_214 | 2238 | 2358 | + |
| scaffold_215 | 1 | 541 | + |
| scaffold_215 | 1011 | 1545 | + |
| scaffold_215 | 1882 | 2354 | + |
| scaffold_215 | 633 | 950 | + |
| scaffold_216 | 1 | 2246 | + |
| scaffold_218 | 1193 | 1284 | + |
| scaffold_218 | 1372 | 1919 | + |
| scaffold_218 | 2071 | 2134 | + |
| scaffold_218 | 428 | 1094 | + |
| scaffold_218 | 56 | 367 | + |
| scaffold_219 | 2176 | 2182 | + |
| scaffold_22 | 18195 | 18207 | + |
| scaffold_22 | 252938 | 252938 | + |
| scaffold_22 | 29701 | 32975 | + |
| scaffold_22 | 33148 | 34516 | + |
| scaffold_22 | 351436 | 351446 | + |
| scaffold_22 | 36225 | 37896 | + |
| scaffold_22 | 381744 | 381818 | + |
| scaffold_22 | 388505 | 388546 | + |
| scaffold_22 | 388648 | 388688 | + |
| scaffold_22 | 396098 | 396098 | + |
| scaffold_22 | 396223 | 396256 | + |
| scaffold_22 | 396371 | 396371 | + |
| scaffold_22 | 396935 | 396957 | + |
| scaffold_22 | 397060 | 397250 | + |
| scaffold_22 | 397368 | 397526 | + |
| scaffold_22 | 397968 | 398075 | + |
| scaffold_22 | 398617 | 398700 | + |
| scaffold_22 | 398757 | 398916 | + |
| scaffold_22 | 399680 | 399769 | + |
| scaffold_22 | 400272 | 400506 | + |
| scaffold_22 | 401506 | 401698 | + |
| scaffold_22 | 401889 | 401895 | + |
| scaffold_22 | 402360 | 402516 | + |
| scaffold_22 | 402749 | 402900 | + |
| scaffold_22 | 404164 | 404170 | + |
| scaffold_22 | 404314 | 404329 | + |
| scaffold_22 | 405707 | 405808 | + |
| scaffold_22 | 406179 | 406231 | + |
| scaffold_22 | 406721 | 409384 | + |
| scaffold_22 | 409663 | 409873 | + |
| scaffold_22 | 410074 | 410481 | + |
| scaffold_22 | 410931 | 411160 | + |
| scaffold_22 | 411221 | 411514 | + |
| scaffold_22 | 411681 | 412233 | + |
| scaffold_22 | 412305 | 412428 | + |
| scaffold_22 | 412544 | 417864 | + |
| scaffold_22 | 417919 | 419409 | + |
| scaffold_22 | 419452 | 421048 | + |
| scaffold_22 | 421128 | 422180 | + |
| scaffold_22 | 422232 | 425530 | + |
| scaffold_22 | 425865 | 427514 | + |
| scaffold_22 | 427548 | 428315 | + |
| scaffold_22 | 428692 | 429448 | + |
| scaffold_22 | 429680 | 429727 | + |
| scaffold_22 | 429837 | 430761 | + |
| scaffold_22 | 431390 | 431404 | + |
| scaffold_22 | 431538 | 431575 | + |
| scaffold_22 | 432302 | 432643 | + |
| scaffold_22 | 432940 | 433713 | + |
| scaffold_22 | 433763 | 434621 | + |
| scaffold_22 | 434764 | 436396 | + |
| scaffold_22 | 436648 | 436926 | + |
| scaffold_22 | 437019 | 437224 | + |
| scaffold_22 | 437572 | 437637 | + |
| scaffold_22 | 437690 | 444861 | + |
| scaffold_22 | 445086 | 445226 | + |
| scaffold_22 | 445323 | 446407 | + |
| scaffold_22 | 446489 | 447381 | + |
| scaffold_22 | 447446 | 447646 | + |
| scaffold_22 | 447852 | 450153 | + |
| scaffold_22 | 450417 | 450640 | + |
| scaffold_22 | 451336 | 451977 | + |
| scaffold_22 | 452295 | 453270 | + |
| scaffold_22 | 453326 | 453587 | + |
| scaffold_22 | 460989 | 461148 | + |
| scaffold_22 | 461260 | 462283 | + |
| scaffold_22 | 462606 | 462694 | + |
| scaffold_22 | 462839 | 462928 | + |
| scaffold_22 | 463284 | 463289 | + |
| scaffold_22 | 465591 | 466824 | + |
| scaffold_22 | 470666 | 470689 | + |
| scaffold_22 | 470968 | 471092 | + |
| scaffold_220 | 1570 | 2131 | + |
| scaffold_220 | 385 | 450 | + |
| scaffold_223 | 56 | 1714 | + |
| scaffold_225 | 1 | 600 | + |
| scaffold_225 | 652 | 1857 | + |
| scaffold_226 | 1181 | 1368 | + |
| scaffold_226 | 1814 | 2032 | + |
| scaffold_226 | 763 | 1056 | + |
| scaffold_228 | 1 | 1965 | + |
| scaffold_23 | 100328 | 100329 | + |
| scaffold_23 | 100448 | 100455 | + |
| scaffold_23 | 101105 | 101105 | + |
| scaffold_23 | 106691 | 106691 | + |
| scaffold_23 | 124320 | 124340 | + |
| scaffold_23 | 124373 | 124376 | + |
| scaffold_23 | 124681 | 124760 | + |
| scaffold_23 | 126638 | 126639 | + |
| scaffold_23 | 128000 | 130592 | + |
| scaffold_23 | 13485 | 13638 | + |
| scaffold_23 | 160873 | 160889 | + |
| scaffold_23 | 166931 | 166932 | + |
| scaffold_23 | 193913 | 194440 | + |
| scaffold_23 | 206476 | 206626 | + |
| scaffold_23 | 206779 | 206867 | + |
| scaffold_23 | 207079 | 207652 | + |
| scaffold_23 | 29496 | 29586 | + |
| scaffold_23 | 302930 | 302933 | + |
| scaffold_23 | 307098 | 307098 | + |
| scaffold_23 | 316745 | 316753 | + |
| scaffold_23 | 334369 | 334383 | + |
| scaffold_23 | 339369 | 339482 | + |
| scaffold_23 | 382563 | 382573 | + |
| scaffold_23 | 410950 | 411053 | + |
| scaffold_23 | 45376 | 45378 | + |
| scaffold_23 | 94712 | 94713 | + |
| scaffold_23 | 95180 | 95181 | + |
| scaffold_23 | 95648 | 95649 | + |
| scaffold_23 | 96116 | 96117 | + |
| scaffold_23 | 96584 | 96585 | + |
| scaffold_23 | 97052 | 97053 | + |
| scaffold_23 | 97520 | 97521 | + |
| scaffold_23 | 9758 | 9768 | + |
| scaffold_23 | 97988 | 97989 | + |
| scaffold_23 | 98456 | 98457 | + |
| scaffold_23 | 98924 | 98925 | + |
| scaffold_23 | 99392 | 99393 | + |
| scaffold_23 | 99860 | 99861 | + |
| scaffold_232 | 1 | 16 | + |
| scaffold_232 | 56 | 1568 | + |
| scaffold_233 | 1 | 695 | + |
| scaffold_233 | 743 | 1792 | + |
| scaffold_238 | 1 | 1713 | + |
| scaffold_24 | 160880 | 160917 | + |
| scaffold_24 | 173744 | 173747 | + |
| scaffold_24 | 204608 | 204609 | + |
| scaffold_24 | 210573 | 210573 | + |
| scaffold_24 | 231320 | 231354 | + |
| scaffold_24 | 232968 | 232970 | + |
| scaffold_24 | 274217 | 274231 | + |
| scaffold_24 | 28375 | 28375 | + |
| scaffold_24 | 293488 | 293502 | + |
| scaffold_24 | 302349 | 302350 | + |
| scaffold_24 | 314681 | 317886 | + |
| scaffold_24 | 321994 | 321995 | + |
| scaffold_24 | 363092 | 363092 | + |
| scaffold_24 | 53793 | 53895 | + |
| scaffold_24 | 56781 | 57634 | + |
| scaffold_240 | 118 | 179 | + |
| scaffold_240 | 280 | 410 | + |
| scaffold_240 | 732 | 759 | + |
| scaffold_241 | 1 | 56 | + |
| scaffold_241 | 1226 | 1228 | + |
| scaffold_243 | 1 | 984 | + |
| scaffold_243 | 1025 | 1030 | + |
| scaffold_243 | 1130 | 1541 | + |
| scaffold_246 | 1 | 1486 | + |
| scaffold_25 | 114014 | 114015 | + |
| scaffold_25 | 140382 | 140436 | + |
| scaffold_25 | 151240 | 151259 | + |
| scaffold_25 | 239447 | 241066 | + |
| scaffold_25 | 241864 | 241866 | + |
| scaffold_25 | 243649 | 243681 | + |
| scaffold_25 | 252165 | 252188 | + |
| scaffold_25 | 252725 | 252741 | + |
| scaffold_25 | 265196 | 265309 | + |
| scaffold_25 | 270293 | 270503 | + |
| scaffold_25 | 272930 | 273083 | + |
| scaffold_25 | 284150 | 284303 | + |
| scaffold_25 | 302174 | 302805 | + |
| scaffold_25 | 303275 | 303325 | + |
| scaffold_25 | 304620 | 304996 | + |
| scaffold_25 | 305091 | 305215 | + |
| scaffold_25 | 305479 | 305555 | + |
| scaffold_25 | 305869 | 305920 | + |
| scaffold_25 | 307358 | 307395 | + |
| scaffold_25 | 307894 | 307904 | + |
| scaffold_25 | 309068 | 309069 | + |
| scaffold_25 | 314735 | 314738 | + |
| scaffold_25 | 314863 | 315904 | + |
| scaffold_25 | 316059 | 317860 | + |
| scaffold_25 | 327239 | 327241 | + |
| scaffold_25 | 327260 | 327266 | + |
| scaffold_25 | 399941 | 399951 | + |
| scaffold_25 | 71519 | 71523 | + |
| scaffold_251 | 102 | 522 | + |
| scaffold_251 | 572 | 1420 | + |
| scaffold_252 | 146 | 520 | + |
| scaffold_252 | 834 | 1018 | + |
| scaffold_255 | 1320 | 1320 | + |
| scaffold_255 | 56 | 92 | + |
| scaffold_257 | 286 | 376 | + |
| scaffold_257 | 861 | 917 | + |
| scaffold_257 | 91 | 169 | + |
| scaffold_26 | 111265 | 111273 | + |
| scaffold_26 | 117680 | 117687 | + |
| scaffold_26 | 154886 | 154886 | + |
| scaffold_26 | 269105 | 269106 | + |
| scaffold_26 | 269573 | 269574 | + |
| scaffold_26 | 270041 | 270042 | + |
| scaffold_26 | 270509 | 270510 | + |
| scaffold_26 | 270977 | 270978 | + |
| scaffold_26 | 271445 | 271446 | + |
| scaffold_26 | 271913 | 271914 | + |
| scaffold_26 | 272381 | 272382 | + |
| scaffold_26 | 272849 | 272850 | + |
| scaffold_26 | 273317 | 273318 | + |
| scaffold_26 | 273785 | 273786 | + |
| scaffold_26 | 274253 | 274254 | + |
| scaffold_26 | 353754 | 353958 | + |
| scaffold_26 | 49 | 1076 | + |
| scaffold_26 | 53808 | 53826 | + |
| scaffold_26 | 74380 | 74381 | + |
| scaffold_261 | 1 | 13 | + |
| scaffold_261 | 93 | 1299 | + |
| scaffold_263 | 1 | 4 | + |
| scaffold_263 | 1151 | 1154 | + |
| scaffold_265 | 334 | 415 | + |
| scaffold_265 | 58 | 183 | + |
| scaffold_268 | 1 | 2 | + |
| scaffold_268 | 1214 | 1220 | + |
| scaffold_268 | 708 | 716 | + |
| scaffold_268 | 776 | 1039 | + |
| scaffold_27 | 109195 | 109201 | + |
| scaffold_27 | 109212 | 109215 | + |
| scaffold_27 | 115722 | 115733 | + |
| scaffold_27 | 116303 | 116312 | + |
| scaffold_27 | 125270 | 125282 | + |
| scaffold_27 | 125343 | 125344 | + |
| scaffold_27 | 163065 | 163068 | + |
| scaffold_27 | 170174 | 170177 | + |
| scaffold_27 | 253571 | 253588 | + |
| scaffold_27 | 254264 | 257346 | + |
| scaffold_27 | 263289 | 263305 | + |
| scaffold_27 | 267738 | 267748 | + |
| scaffold_27 | 270529 | 270536 | + |
| scaffold_27 | 273396 | 273397 | + |
| scaffold_27 | 298787 | 298792 | + |
| scaffold_27 | 6535 | 6538 | + |
| scaffold_27 | 6578 | 6926 | + |
| scaffold_278 | 1 | 16 | + |
| scaffold_28 | 1024 | 1025 | + |
| scaffold_28 | 105587 | 105588 | + |
| scaffold_28 | 1492 | 1493 | + |
| scaffold_28 | 178885 | 179038 | + |
| scaffold_28 | 1960 | 1961 | + |
| scaffold_28 | 2428 | 2429 | + |
| scaffold_28 | 263401 | 263419 | + |
| scaffold_28 | 264235 | 264246 | + |
| scaffold_28 | 264892 | 264893 | + |
| scaffold_28 | 265360 | 265361 | + |
| scaffold_28 | 265828 | 265829 | + |
| scaffold_28 | 266296 | 266297 | + |
| scaffold_28 | 266764 | 266765 | + |
| scaffold_28 | 267232 | 267233 | + |
| scaffold_28 | 267700 | 267701 | + |
| scaffold_28 | 268168 | 268169 | + |
| scaffold_28 | 268636 | 268637 | + |
| scaffold_28 | 269104 | 269105 | + |
| scaffold_28 | 269572 | 269573 | + |
| scaffold_28 | 270040 | 270041 | + |
| scaffold_28 | 270508 | 270509 | + |
| scaffold_28 | 2896 | 2897 | + |
| scaffold_28 | 293376 | 293376 | + |
| scaffold_28 | 325233 | 325336 | + |
| scaffold_28 | 3364 | 3365 | + |
| scaffold_28 | 3832 | 3833 | + |
| scaffold_28 | 4300 | 4301 | + |
| scaffold_28 | 4768 | 4769 | + |
| scaffold_28 | 5236 | 5241 | + |
| scaffold_28 | 54755 | 54756 | + |
| scaffold_28 | 556 | 557 | + |
| scaffold_28 | 97750 | 97753 | + |
| scaffold_280 | 1 | 1116 | + |
| scaffold_281 | 1 | 8 | + |
| scaffold_281 | 1106 | 1106 | + |
| scaffold_283 | 230 | 730 | + |
| scaffold_284 | 1010 | 1098 | + |
| scaffold_284 | 635 | 959 | + |
| scaffold_284 | 95 | 185 | + |
| scaffold_285 | 1 | 26 | + |
| scaffold_285 | 1060 | 1074 | + |
| scaffold_285 | 149 | 787 | + |
| scaffold_285 | 818 | 941 | + |
| scaffold_288 | 1 | 35 | + |
| scaffold_288 | 130 | 1061 | + |
| scaffold_29 | 10183 | 11474 | + |
| scaffold_29 | 11906 | 11998 | + |
| scaffold_29 | 13016 | 13931 | + |
| scaffold_29 | 14065 | 15155 | + |
| scaffold_29 | 1803 | 2340 | + |
| scaffold_29 | 181185 | 181192 | + |
| scaffold_29 | 185600 | 185603 | + |
| scaffold_29 | 185657 | 185658 | + |
| scaffold_29 | 195095 | 195098 | + |
| scaffold_29 | 200927 | 201030 | + |
| scaffold_29 | 262543 | 262545 | + |
| scaffold_29 | 275866 | 276026 | + |
| scaffold_29 | 276319 | 278245 | + |
| scaffold_29 | 279718 | 290607 | + |
| scaffold_29 | 290673 | 292405 | + |
| scaffold_29 | 292499 | 292814 | + |
| scaffold_29 | 294646 | 294680 | + |
| scaffold_29 | 305189 | 305279 | + |
| scaffold_29 | 305728 | 307114 | + |
| scaffold_29 | 307200 | 307871 | + |
| scaffold_29 | 308145 | 309171 | + |
| scaffold_29 | 3678 | 3681 | + |
| scaffold_29 | 5247 | 5864 | + |
| scaffold_29 | 6164 | 6308 | + |
| scaffold_29 | 6417 | 6561 | + |
| scaffold_29 | 8233 | 8238 | + |
| scaffold_29 | 8475 | 9825 | + |
| scaffold_29 | 976 | 986 | + |
| scaffold_29 | 9957 | 9963 | + |
| scaffold_290 | 1048 | 1049 | + |
| scaffold_292 | 60 | 948 | + |
| scaffold_3 | 1022628 | 1022631 | + |
| scaffold_3 | 1037930 | 1037930 | + |
| scaffold_3 | 1088659 | 1088673 | + |
| scaffold_3 | 1090655 | 1090656 | + |
| scaffold_3 | 1090667 | 1090667 | + |
| scaffold_3 | 1099529 | 1099529 | + |
| scaffold_3 | 1134772 | 1134778 | + |
| scaffold_3 | 119717 | 119732 | + |
| scaffold_3 | 1211450 | 1211571 | + |
| scaffold_3 | 12578 | 12580 | + |
| scaffold_3 | 1259 | 1369 | + |
| scaffold_3 | 1280415 | 1280419 | + |
| scaffold_3 | 1414098 | 1414105 | + |
| scaffold_3 | 143157 | 143251 | + |
| scaffold_3 | 14357 | 14357 | + |
| scaffold_3 | 1466352 | 1466354 | + |
| scaffold_3 | 1482853 | 1482871 | + |
| scaffold_3 | 1484616 | 1484623 | + |
| scaffold_3 | 1484664 | 1484667 | + |
| scaffold_3 | 1491062 | 1491063 | + |
| scaffold_3 | 1496863 | 1496870 | + |
| scaffold_3 | 1541076 | 1541086 | + |
| scaffold_3 | 1555516 | 1555524 | + |
| scaffold_3 | 1555578 | 1555595 | + |
| scaffold_3 | 1582 | 1593 | + |
| scaffold_3 | 1595708 | 1595711 | + |
| scaffold_3 | 1614153 | 1614158 | + |
| scaffold_3 | 1638517 | 1638518 | + |
| scaffold_3 | 1654908 | 1654915 | + |
| scaffold_3 | 1674247 | 1674252 | + |
| scaffold_3 | 1674344 | 1674345 | + |
| scaffold_3 | 1792389 | 1792389 | + |
| scaffold_3 | 1808602 | 1808615 | + |
| scaffold_3 | 18422 | 18443 | + |
| scaffold_3 | 18497 | 18517 | + |
| scaffold_3 | 1934792 | 1934795 | + |
| scaffold_3 | 1941375 | 1941379 | + |
| scaffold_3 | 2133009 | 2135213 | + |
| scaffold_3 | 2166223 | 2166225 | + |
| scaffold_3 | 2166381 | 2166575 | + |
| scaffold_3 | 2166792 | 2166807 | + |
| scaffold_3 | 2178779 | 2178871 | + |
| scaffold_3 | 2213 | 2234 | + |
| scaffold_3 | 2328917 | 2328969 | + |
| scaffold_3 | 2780 | 2787 | + |
| scaffold_3 | 28343 | 28371 | + |
| scaffold_3 | 315933 | 316215 | + |
| scaffold_3 | 318719 | 318729 | + |
| scaffold_3 | 319521 | 319637 | + |
| scaffold_3 | 327127 | 327397 | + |
| scaffold_3 | 327479 | 329435 | + |
| scaffold_3 | 329492 | 330108 | + |
| scaffold_3 | 330650 | 330656 | + |
| scaffold_3 | 331374 | 331802 | + |
| scaffold_3 | 33167 | 33766 | + |
| scaffold_3 | 332328 | 333287 | + |
| scaffold_3 | 333362 | 333722 | + |
| scaffold_3 | 333797 | 333804 | + |
| scaffold_3 | 335723 | 335793 | + |
| scaffold_3 | 336532 | 337615 | + |
| scaffold_3 | 339030 | 339060 | + |
| scaffold_3 | 339342 | 339585 | + |
| scaffold_3 | 341621 | 344435 | + |
| scaffold_3 | 344728 | 344969 | + |
| scaffold_3 | 345057 | 345213 | + |
| scaffold_3 | 345282 | 345576 | + |
| scaffold_3 | 345911 | 346284 | + |
| scaffold_3 | 346377 | 346834 | + |
| scaffold_3 | 347023 | 347975 | + |
| scaffold_3 | 348037 | 348390 | + |
| scaffold_3 | 348878 | 358058 | + |
| scaffold_3 | 358897 | 359007 | + |
| scaffold_3 | 359125 | 359172 | + |
| scaffold_3 | 359261 | 359311 | + |
| scaffold_3 | 361018 | 365837 | + |
| scaffold_3 | 36225 | 36228 | + |
| scaffold_3 | 372779 | 380655 | + |
| scaffold_3 | 37795 | 40016 | + |
| scaffold_3 | 380825 | 380863 | + |
| scaffold_3 | 380976 | 381464 | + |
| scaffold_3 | 381596 | 384158 | + |
| scaffold_3 | 384491 | 384543 | + |
| scaffold_3 | 384597 | 384639 | + |
| scaffold_3 | 384684 | 385672 | + |
| scaffold_3 | 385983 | 385995 | + |
| scaffold_3 | 386106 | 388080 | + |
| scaffold_3 | 389358 | 389502 | + |
| scaffold_3 | 389784 | 389892 | + |
| scaffold_3 | 390084 | 390344 | + |
| scaffold_3 | 391251 | 391326 | + |
| scaffold_3 | 392109 | 392143 | + |
| scaffold_3 | 392572 | 392707 | + |
| scaffold_3 | 393091 | 393263 | + |
| scaffold_3 | 393517 | 393570 | + |
| scaffold_3 | 393714 | 394171 | + |
| scaffold_3 | 395372 | 395603 | + |
| scaffold_3 | 395860 | 396006 | + |
| scaffold_3 | 396135 | 396653 | + |
| scaffold_3 | 396749 | 399521 | + |
| scaffold_3 | 399609 | 400125 | + |
| scaffold_3 | 400184 | 400280 | + |
| scaffold_3 | 40057 | 41335 | + |
| scaffold_3 | 4060 | 4110 | + |
| scaffold_3 | 41448 | 42068 | + |
| scaffold_3 | 42275 | 42480 | + |
| scaffold_3 | 44755 | 44758 | + |
| scaffold_3 | 531581 | 531590 | + |
| scaffold_3 | 533 | 642 | + |
| scaffold_3 | 555215 | 555215 | + |
| scaffold_3 | 574727 | 574730 | + |
| scaffold_3 | 579806 | 579810 | + |
| scaffold_3 | 579871 | 579884 | + |
| scaffold_3 | 58 | 90 | + |
| scaffold_3 | 581208 | 581220 | + |
| scaffold_3 | 581325 | 581332 | + |
| scaffold_3 | 634877 | 634878 | + |
| scaffold_3 | 681851 | 681853 | + |
| scaffold_3 | 681872 | 681876 | + |
| scaffold_3 | 697075 | 697082 | + |
| scaffold_3 | 697143 | 697159 | + |
| scaffold_3 | 710416 | 710418 | + |
| scaffold_3 | 832482 | 832487 | + |
| scaffold_3 | 860941 | 860945 | + |
| scaffold_3 | 893709 | 893712 | + |
| scaffold_3 | 893823 | 893845 | + |
| scaffold_3 | 899200 | 899296 | + |
| scaffold_3 | 97561 | 97578 | + |
| scaffold_30 | 105636 | 105642 | + |
| scaffold_30 | 146444 | 147043 | + |
| scaffold_30 | 226744 | 226749 | + |
| scaffold_30 | 226760 | 226763 | + |
| scaffold_30 | 266776 | 266779 | + |
| scaffold_30 | 281884 | 281892 | + |
| scaffold_30 | 291319 | 291480 | + |
| scaffold_30 | 299130 | 299629 | + |
| scaffold_30 | 299820 | 300567 | + |
| scaffold_30 | 97202 | 97261 | + |
| scaffold_31 | 10075 | 10149 | + |
| scaffold_31 | 10621 | 19069 | + |
| scaffold_31 | 127890 | 127894 | + |
| scaffold_31 | 186225 | 186225 | + |
| scaffold_31 | 1911 | 2967 | + |
| scaffold_31 | 19205 | 19982 | + |
| scaffold_31 | 23371 | 23396 | + |
| scaffold_31 | 27406 | 28396 | + |
| scaffold_31 | 28911 | 30513 | + |
| scaffold_31 | 3147 | 3508 | + |
| scaffold_31 | 32318 | 32425 | + |
| scaffold_31 | 32934 | 33045 | + |
| scaffold_31 | 34269 | 35328 | + |
| scaffold_31 | 3580 | 5572 | + |
| scaffold_31 | 36196 | 36203 | + |
| scaffold_31 | 36765 | 36799 | + |
| scaffold_31 | 37279 | 37582 | + |
| scaffold_31 | 37652 | 38303 | + |
| scaffold_31 | 38793 | 39191 | + |
| scaffold_31 | 39255 | 39446 | + |
| scaffold_31 | 39944 | 42117 | + |
| scaffold_31 | 45990 | 47261 | + |
| scaffold_31 | 47440 | 47447 | + |
| scaffold_31 | 53704 | 53762 | + |
| scaffold_31 | 54 | 1756 | + |
| scaffold_31 | 54353 | 54541 | + |
| scaffold_31 | 55613 | 55704 | + |
| scaffold_31 | 56331 | 56448 | + |
| scaffold_31 | 5922 | 6216 | + |
| scaffold_31 | 65028 | 65030 | + |
| scaffold_31 | 65667 | 66015 | + |
| scaffold_31 | 66055 | 66058 | + |
| scaffold_31 | 6724 | 6777 | + |
| scaffold_31 | 7310 | 7601 | + |
| scaffold_31 | 8927 | 8959 | + |
| scaffold_31 | 9141 | 9166 | + |
| scaffold_31 | 9226 | 9304 | + |
| scaffold_31 | 9433 | 9572 | + |
| scaffold_31 | 9825 | 10020 | + |
| scaffold_32 | 110501 | 110504 | + |
| scaffold_32 | 155130 | 155136 | + |
| scaffold_32 | 180192 | 180192 | + |
| scaffold_32 | 236299 | 236300 | + |
| scaffold_32 | 237904 | 237905 | + |
| scaffold_32 | 238372 | 238373 | + |
| scaffold_32 | 238840 | 238841 | + |
| scaffold_32 | 239308 | 239309 | + |
| scaffold_32 | 239776 | 239777 | + |
| scaffold_32 | 240244 | 240245 | + |
| scaffold_32 | 240712 | 240713 | + |
| scaffold_32 | 241180 | 241181 | + |
| scaffold_32 | 241648 | 241649 | + |
| scaffold_32 | 242116 | 242117 | + |
| scaffold_32 | 242584 | 242585 | + |
| scaffold_32 | 243052 | 243053 | + |
| scaffold_32 | 243520 | 243521 | + |
| scaffold_32 | 253463 | 253776 | + |
| scaffold_32 | 253832 | 253833 | + |
| scaffold_32 | 253884 | 258784 | + |
| scaffold_32 | 258879 | 259576 | + |
| scaffold_32 | 259672 | 261516 | + |
| scaffold_32 | 261677 | 262132 | + |
| scaffold_32 | 262312 | 262585 | + |
| scaffold_32 | 262682 | 264487 | + |
| scaffold_32 | 42307 | 42307 | + |
| scaffold_33 | 106406 | 106507 | + |
| scaffold_33 | 110158 | 110174 | + |
| scaffold_33 | 116348 | 116349 | + |
| scaffold_33 | 119895 | 120000 | + |
| scaffold_33 | 120228 | 120480 | + |
| scaffold_33 | 121756 | 121756 | + |
| scaffold_33 | 13376 | 13403 | + |
| scaffold_33 | 13710 | 14194 | + |
| scaffold_33 | 182457 | 184225 | + |
| scaffold_33 | 18983 | 18983 | + |
| scaffold_33 | 239994 | 241244 | + |
| scaffold_33 | 24008 | 25047 | + |
| scaffold_33 | 25119 | 25839 | + |
| scaffold_33 | 48327 | 48551 | + |
| scaffold_33 | 8142 | 8152 | + |
| scaffold_33 | 98056 | 98263 | + |
| scaffold_34 | 109783 | 109787 | + |
| scaffold_34 | 129087 | 129100 | + |
| scaffold_34 | 138892 | 138895 | + |
| scaffold_34 | 139155 | 139157 | + |
| scaffold_34 | 26593 | 26595 | + |
| scaffold_34 | 62295 | 62298 | + |
| scaffold_35 | 100253 | 100439 | + |
| scaffold_35 | 100501 | 102903 | + |
| scaffold_35 | 102966 | 108824 | + |
| scaffold_35 | 178979 | 179007 | + |
| scaffold_35 | 179092 | 179153 | + |
| scaffold_35 | 217568 | 218860 | + |
| scaffold_35 | 219054 | 219087 | + |
| scaffold_35 | 220951 | 220954 | + |
| scaffold_35 | 222035 | 222272 | + |
| scaffold_35 | 41419 | 41874 | + |
| scaffold_35 | 46909 | 47774 | + |
| scaffold_35 | 47896 | 48307 | + |
| scaffold_35 | 48424 | 49669 | + |
| scaffold_35 | 49715 | 49788 | + |
| scaffold_35 | 55136 | 55482 | + |
| scaffold_35 | 55558 | 56757 | + |
| scaffold_35 | 56813 | 56821 | + |
| scaffold_35 | 56881 | 58236 | + |
| scaffold_35 | 58466 | 60556 | + |
| scaffold_35 | 60608 | 64430 | + |
| scaffold_35 | 65026 | 65030 | + |
| scaffold_35 | 65250 | 65271 | + |
| scaffold_35 | 65521 | 65536 | + |
| scaffold_35 | 66809 | 66809 | + |
| scaffold_35 | 67010 | 67011 | + |
| scaffold_35 | 67523 | 67554 | + |
| scaffold_35 | 68469 | 68477 | + |
| scaffold_35 | 69888 | 69893 | + |
| scaffold_35 | 7388 | 7779 | + |
| scaffold_35 | 76765 | 76768 | + |
| scaffold_35 | 77134 | 77291 | + |
| scaffold_35 | 77398 | 78324 | + |
| scaffold_35 | 78608 | 78765 | + |
| scaffold_35 | 79473 | 79912 | + |
| scaffold_35 | 80393 | 80448 | + |
| scaffold_35 | 81528 | 81578 | + |
| scaffold_35 | 81688 | 81772 | + |
| scaffold_35 | 82151 | 83391 | + |
| scaffold_35 | 8245 | 8296 | + |
| scaffold_35 | 84411 | 84479 | + |
| scaffold_35 | 84666 | 85738 | + |
| scaffold_35 | 86543 | 86808 | + |
| scaffold_35 | 88151 | 88236 | + |
| scaffold_35 | 88323 | 89039 | + |
| scaffold_35 | 89098 | 89381 | + |
| scaffold_35 | 89784 | 89865 | + |
| scaffold_35 | 89933 | 90012 | + |
| scaffold_35 | 90208 | 91312 | + |
| scaffold_35 | 91353 | 92150 | + |
| scaffold_35 | 92217 | 92282 | + |
| scaffold_35 | 92789 | 92880 | + |
| scaffold_35 | 93580 | 94350 | + |
| scaffold_35 | 94469 | 96244 | + |
| scaffold_35 | 98098 | 98163 | + |
| scaffold_35 | 98377 | 98462 | + |
| scaffold_35 | 98567 | 98652 | + |
| scaffold_35 | 99005 | 100142 | + |
| scaffold_36 | 160363 | 160365 | + |
| scaffold_36 | 178850 | 178866 | + |
| scaffold_36 | 206100 | 206103 | + |
| scaffold_36 | 5271 | 5280 | + |
| scaffold_36 | 72526 | 72579 | + |
| scaffold_37 | 152654 | 152757 | + |
| scaffold_37 | 164204 | 164296 | + |
| scaffold_37 | 173489 | 173490 | + |
| scaffold_37 | 180343 | 180353 | + |
| scaffold_37 | 185632 | 185639 | + |
| scaffold_37 | 194510 | 194526 | + |
| scaffold_37 | 196015 | 196039 | + |
| scaffold_37 | 196342 | 196352 | + |
| scaffold_37 | 196546 | 196552 | + |
| scaffold_37 | 196632 | 200543 | + |
| scaffold_37 | 200656 | 202380 | + |
| scaffold_37 | 202428 | 206913 | + |
| scaffold_38 | 151365 | 151368 | + |
| scaffold_38 | 207872 | 207876 | + |
| scaffold_38 | 48699 | 48702 | + |
| scaffold_38 | 67351 | 67359 | + |
| scaffold_38 | 67505 | 67505 | + |
| scaffold_39 | 177758 | 177759 | + |
| scaffold_39 | 204192 | 204193 | + |
| scaffold_4 | 101149 | 101151 | + |
| scaffold_4 | 101741 | 101752 | + |
| scaffold_4 | 1088915 | 1088922 | + |
| scaffold_4 | 1095763 | 1095763 | + |
| scaffold_4 | 1169845 | 1169948 | + |
| scaffold_4 | 1242607 | 1242617 | + |
| scaffold_4 | 1243711 | 1243713 | + |
| scaffold_4 | 1243882 | 1243882 | + |
| scaffold_4 | 1304192 | 1304193 | + |
| scaffold_4 | 1308899 | 1308946 | + |
| scaffold_4 | 1387574 | 1387578 | + |
| scaffold_4 | 141745 | 141757 | + |
| scaffold_4 | 141795 | 141807 | + |
| scaffold_4 | 1420669 | 1420675 | + |
| scaffold_4 | 1468693 | 1468695 | + |
| scaffold_4 | 1468716 | 1468723 | + |
| scaffold_4 | 1551500 | 1551503 | + |
| scaffold_4 | 1561830 | 1561830 | + |
| scaffold_4 | 1586826 | 1586829 | + |
| scaffold_4 | 159783 | 159936 | + |
| scaffold_4 | 1609455 | 1609457 | + |
| scaffold_4 | 1690611 | 1690614 | + |
| scaffold_4 | 1704019 | 1704023 | + |
| scaffold_4 | 172724 | 172724 | + |
| scaffold_4 | 1786289 | 1786292 | + |
| scaffold_4 | 1816635 | 1816662 | + |
| scaffold_4 | 1816969 | 1817453 | + |
| scaffold_4 | 1820688 | 1820690 | + |
| scaffold_4 | 1830833 | 1831014 | + |
| scaffold_4 | 1831088 | 1831105 | + |
| scaffold_4 | 1831378 | 1831438 | + |
| scaffold_4 | 1939771 | 1939774 | + |
| scaffold_4 | 202722 | 202732 | + |
| scaffold_4 | 2039915 | 2039917 | + |
| scaffold_4 | 217392 | 218938 | + |
| scaffold_4 | 219026 | 225802 | + |
| scaffold_4 | 2196453 | 2196467 | + |
| scaffold_4 | 2229573 | 2229575 | + |
| scaffold_4 | 2229601 | 2229604 | + |
| scaffold_4 | 225863 | 231566 | + |
| scaffold_4 | 2271444 | 2271597 | + |
| scaffold_4 | 2272653 | 2272680 | + |
| scaffold_4 | 2272987 | 2273471 | + |
| scaffold_4 | 231671 | 233163 | + |
| scaffold_4 | 233311 | 233352 | + |
| scaffold_4 | 234772 | 234830 | + |
| scaffold_4 | 234924 | 237899 | + |
| scaffold_4 | 238950 | 238963 | + |
| scaffold_4 | 239351 | 239362 | + |
| scaffold_4 | 239750 | 240152 | + |
| scaffold_4 | 241717 | 241720 | + |
| scaffold_4 | 247822 | 248314 | + |
| scaffold_4 | 248392 | 248623 | + |
| scaffold_4 | 248711 | 249089 | + |
| scaffold_4 | 249179 | 249493 | + |
| scaffold_4 | 249550 | 250066 | + |
| scaffold_4 | 250114 | 251376 | + |
| scaffold_4 | 252011 | 252011 | + |
| scaffold_4 | 252578 | 254797 | + |
| scaffold_4 | 255283 | 255460 | + |
| scaffold_4 | 255654 | 255763 | + |
| scaffold_4 | 351278 | 351285 | + |
| scaffold_4 | 386581 | 386588 | + |
| scaffold_4 | 386606 | 386607 | + |
| scaffold_4 | 426667 | 426669 | + |
| scaffold_4 | 426743 | 426744 | + |
| scaffold_4 | 52875 | 52877 | + |
| scaffold_4 | 588303 | 588307 | + |
| scaffold_4 | 610553 | 610594 | + |
| scaffold_4 | 620941 | 620943 | + |
| scaffold_4 | 626260 | 626284 | + |
| scaffold_4 | 648498 | 648500 | + |
| scaffold_4 | 691513 | 691520 | + |
| scaffold_4 | 691531 | 691538 | + |
| scaffold_4 | 718455 | 718455 | + |
| scaffold_4 | 737284 | 737294 | + |
| scaffold_4 | 783031 | 783032 | + |
| scaffold_4 | 783241 | 783245 | + |
| scaffold_4 | 799096 | 799099 | + |
| scaffold_4 | 799178 | 799184 | + |
| scaffold_4 | 832722 | 832722 | + |
| scaffold_4 | 876159 | 876167 | + |
| scaffold_4 | 887608 | 887613 | + |
| scaffold_4 | 887643 | 887643 | + |
| scaffold_4 | 90757 | 90759 | + |
| scaffold_4 | 90802 | 90803 | + |
| scaffold_4 | 945354 | 945360 | + |
| scaffold_4 | 973345 | 973345 | + |
| scaffold_40 | 132461 | 132461 | + |
| scaffold_40 | 137307 | 137308 | + |
| scaffold_40 | 137775 | 137776 | + |
| scaffold_40 | 138243 | 138244 | + |
| scaffold_40 | 138711 | 138712 | + |
| scaffold_40 | 139179 | 139180 | + |
| scaffold_40 | 139647 | 139648 | + |
| scaffold_40 | 147334 | 147437 | + |
| scaffold_40 | 148559 | 148575 | + |
| scaffold_40 | 148792 | 148810 | + |
| scaffold_40 | 149132 | 155847 | + |
| scaffold_40 | 155885 | 156538 | + |
| scaffold_40 | 156768 | 157442 | + |
| scaffold_40 | 158580 | 158580 | + |
| scaffold_40 | 159476 | 159656 | + |
| scaffold_40 | 159977 | 159998 | + |
| scaffold_40 | 160492 | 160494 | + |
| scaffold_40 | 160716 | 160724 | + |
| scaffold_40 | 160926 | 162233 | + |
| scaffold_40 | 162727 | 162849 | + |
| scaffold_40 | 163057 | 163292 | + |
| scaffold_40 | 163474 | 163644 | + |
| scaffold_40 | 163899 | 164397 | + |
| scaffold_40 | 164453 | 164881 | + |
| scaffold_40 | 164999 | 166055 | + |
| scaffold_40 | 166289 | 166315 | + |
| scaffold_40 | 166891 | 166895 | + |
| scaffold_40 | 170438 | 170439 | + |
| scaffold_40 | 174846 | 174872 | + |
| scaffold_40 | 176889 | 177018 | + |
| scaffold_40 | 178480 | 178560 | + |
| scaffold_40 | 178650 | 179119 | + |
| scaffold_40 | 179175 | 179239 | + |
| scaffold_40 | 179323 | 183812 | + |
| scaffold_40 | 187756 | 187989 | + |
| scaffold_40 | 188696 | 188896 | + |
| scaffold_40 | 189387 | 189894 | + |
| scaffold_40 | 191651 | 191654 | + |
| scaffold_40 | 191940 | 192357 | + |
| scaffold_40 | 192567 | 192651 | + |
| scaffold_40 | 193083 | 194118 | + |
| scaffold_40 | 194801 | 194849 | + |
| scaffold_40 | 194935 | 196411 | + |
| scaffold_40 | 196842 | 196852 | + |
| scaffold_40 | 198439 | 199303 | + |
| scaffold_40 | 199699 | 201249 | + |
| scaffold_40 | 201455 | 202405 | + |
| scaffold_40 | 203076 | 204360 | + |
| scaffold_40 | 26059 | 27276 | + |
| scaffold_40 | 27329 | 27525 | + |
| scaffold_40 | 27953 | 29088 | + |
| scaffold_40 | 29163 | 29671 | + |
| scaffold_40 | 54287 | 58211 | + |
| scaffold_41 | 115072 | 115076 | + |
| scaffold_41 | 12932 | 12932 | + |
| scaffold_41 | 1329 | 2076 | + |
| scaffold_41 | 158328 | 160441 | + |
| scaffold_41 | 181595 | 181598 | + |
| scaffold_41 | 2267 | 2766 | + |
| scaffold_41 | 36505 | 36597 | + |
| scaffold_41 | 57676 | 57677 | + |
| scaffold_41 | 84366 | 84369 | + |
| scaffold_42 | 127192 | 127194 | + |
| scaffold_42 | 153414 | 153414 | + |
| scaffold_42 | 153453 | 153455 | + |
| scaffold_42 | 174142 | 174152 | + |
| scaffold_42 | 176787 | 177564 | + |
| scaffold_43 | 128034 | 128345 | + |
| scaffold_44 | 110148 | 113867 | + |
| scaffold_44 | 114013 | 115790 | + |
| scaffold_44 | 116278 | 116770 | + |
| scaffold_44 | 120315 | 120316 | + |
| scaffold_44 | 1445 | 3106 | + |
| scaffold_44 | 16154 | 16642 | + |
| scaffold_44 | 161816 | 161870 | + |
| scaffold_44 | 162467 | 163645 | + |
| scaffold_44 | 3198 | 3333 | + |
| scaffold_44 | 3499 | 3979 | + |
| scaffold_44 | 35328 | 35330 | + |
| scaffold_44 | 92245 | 92251 | + |
| scaffold_45 | 18822 | 18935 | + |
| scaffold_45 | 22355 | 22397 | + |
| scaffold_45 | 22902 | 23001 | + |
| scaffold_45 | 25382 | 25382 | + |
| scaffold_45 | 25974 | 26895 | + |
| scaffold_45 | 27113 | 27228 | + |
| scaffold_45 | 28142 | 28268 | + |
| scaffold_45 | 28358 | 28365 | + |
| scaffold_45 | 28483 | 28813 | + |
| scaffold_45 | 66273 | 66274 | + |
| scaffold_45 | 66741 | 66742 | + |
| scaffold_45 | 67209 | 67210 | + |
| scaffold_45 | 67677 | 67678 | + |
| scaffold_45 | 67832 | 67881 | + |
| scaffold_45 | 85684 | 85712 | + |
| scaffold_46 | 124725 | 124728 | + |
| scaffold_46 | 147147 | 147239 | + |
| scaffold_46 | 152447 | 152448 | + |
| scaffold_46 | 156447 | 156600 | + |
| scaffold_46 | 2329 | 2329 | + |
| scaffold_46 | 34307 | 34312 | + |
| scaffold_46 | 52142 | 52148 | + |
| scaffold_46 | 52678 | 52688 | + |
| scaffold_46 | 58288 | 58289 | + |
| scaffold_46 | 6265 | 6268 | + |
| scaffold_46 | 68283 | 70117 | + |
| scaffold_47 | 16109 | 16587 | + |
| scaffold_47 | 16640 | 16956 | + |
| scaffold_47 | 17093 | 17185 | + |
| scaffold_47 | 17285 | 17403 | + |
| scaffold_47 | 17526 | 17563 | + |
| scaffold_47 | 17628 | 18048 | + |
| scaffold_47 | 22458 | 22493 | + |
| scaffold_47 | 32522 | 32572 | + |
| scaffold_47 | 3364 | 3369 | + |
| scaffold_47 | 37644 | 37644 | + |
| scaffold_47 | 42980 | 43072 | + |
| scaffold_47 | 53233 | 54231 | + |
| scaffold_47 | 71027 | 71130 | + |
| scaffold_48 | 123126 | 123215 | + |
| scaffold_49 | 101375 | 101379 | + |
| scaffold_49 | 101864 | 102569 | + |
| scaffold_49 | 112567 | 112576 | + |
| scaffold_49 | 124279 | 124280 | + |
| scaffold_49 | 3624 | 3634 | + |
| scaffold_49 | 81138 | 81355 | + |
| scaffold_49 | 98170 | 98171 | + |
| scaffold_5 | 1010 | 1011 | + |
| scaffold_5 | 1010064 | 1010074 | + |
| scaffold_5 | 1114129 | 1114129 | + |
| scaffold_5 | 1270472 | 1270867 | + |
| scaffold_5 | 1270924 | 1271083 | + |
| scaffold_5 | 1292260 | 1292262 | + |
| scaffold_5 | 1386923 | 1387295 | + |
| scaffold_5 | 1387340 | 1387569 | + |
| scaffold_5 | 1388078 | 1388285 | + |
| scaffold_5 | 1388693 | 1388869 | + |
| scaffold_5 | 1389287 | 1389519 | + |
| scaffold_5 | 1389597 | 1389945 | + |
| scaffold_5 | 1403696 | 1403697 | + |
| scaffold_5 | 1450515 | 1450517 | + |
| scaffold_5 | 1457953 | 1457956 | + |
| scaffold_5 | 1461517 | 1461520 | + |
| scaffold_5 | 1465971 | 1465982 | + |
| scaffold_5 | 1478 | 1479 | + |
| scaffold_5 | 1550742 | 1550748 | + |
| scaffold_5 | 1572005 | 1572020 | + |
| scaffold_5 | 1579153 | 1579153 | + |
| scaffold_5 | 1597097 | 1597113 | + |
| scaffold_5 | 1600595 | 1600600 | + |
| scaffold_5 | 1600624 | 1600635 | + |
| scaffold_5 | 1633618 | 1633630 | + |
| scaffold_5 | 1689254 | 1689259 | + |
| scaffold_5 | 1749607 | 1749607 | + |
| scaffold_5 | 1749743 | 1749751 | + |
| scaffold_5 | 176719 | 176727 | + |
| scaffold_5 | 1783606 | 1783607 | + |
| scaffold_5 | 1784028 | 1784033 | + |
| scaffold_5 | 1805011 | 1805015 | + |
| scaffold_5 | 1805026 | 1805032 | + |
| scaffold_5 | 1932903 | 1932940 | + |
| scaffold_5 | 1946 | 1947 | + |
| scaffold_5 | 1961069 | 1961460 | + |
| scaffold_5 | 1962557 | 1962583 | + |
| scaffold_5 | 1963064 | 1964205 | + |
| scaffold_5 | 1968666 | 1973536 | + |
| scaffold_5 | 1974466 | 1974468 | + |
| scaffold_5 | 1974629 | 1976425 | + |
| scaffold_5 | 1977161 | 1978077 | + |
| scaffold_5 | 2052339 | 2052360 | + |
| scaffold_5 | 2107450 | 2107450 | + |
| scaffold_5 | 2109635 | 2109636 | + |
| scaffold_5 | 221108 | 221111 | + |
| scaffold_5 | 2414 | 2415 | + |
| scaffold_5 | 2882 | 2883 | + |
| scaffold_5 | 3350 | 3351 | + |
| scaffold_5 | 381140 | 381145 | + |
| scaffold_5 | 3818 | 3819 | + |
| scaffold_5 | 417806 | 417807 | + |
| scaffold_5 | 424 | 425 | + |
| scaffold_5 | 4286 | 4287 | + |
| scaffold_5 | 453103 | 453104 | + |
| scaffold_5 | 453139 | 453144 | + |
| scaffold_5 | 460111 | 460120 | + |
| scaffold_5 | 4754 | 4755 | + |
| scaffold_5 | 477427 | 477432 | + |
| scaffold_5 | 477443 | 477462 | + |
| scaffold_5 | 5222 | 5223 | + |
| scaffold_5 | 5690 | 5691 | + |
| scaffold_5 | 577835 | 577836 | + |
| scaffold_5 | 5804 | 5806 | + |
| scaffold_5 | 685490 | 685586 | + |
| scaffold_5 | 793608 | 793616 | + |
| scaffold_5 | 804890 | 804894 | + |
| scaffold_5 | 820238 | 820246 | + |
| scaffold_5 | 820343 | 820347 | + |
| scaffold_5 | 899307 | 899310 | + |
| scaffold_5 | 932547 | 932551 | + |
| scaffold_5 | 96606 | 96613 | + |
| scaffold_5 | 997049 | 997065 | + |
| scaffold_50 | 1 | 1604 | + |
| scaffold_50 | 100108 | 100111 | + |
| scaffold_50 | 10308 | 16049 | + |
| scaffold_50 | 107206 | 107217 | + |
| scaffold_50 | 110719 | 111001 | + |
| scaffold_50 | 129587 | 129588 | + |
| scaffold_50 | 130377 | 130385 | + |
| scaffold_50 | 137189 | 137192 | + |
| scaffold_50 | 16091 | 27116 | + |
| scaffold_50 | 1655 | 3678 | + |
| scaffold_50 | 3733 | 5355 | + |
| scaffold_50 | 5602 | 8436 | + |
| scaffold_50 | 70239 | 77551 | + |
| scaffold_50 | 95275 | 95845 | + |
| scaffold_51 | 107258 | 107290 | + |
| scaffold_51 | 109358 | 109460 | + |
| scaffold_51 | 110572 | 110573 | + |
| scaffold_51 | 114704 | 114728 | + |
| scaffold_51 | 115010 | 115187 | + |
| scaffold_51 | 115305 | 115403 | + |
| scaffold_51 | 120224 | 123171 | + |
| scaffold_51 | 15265 | 17198 | + |
| scaffold_51 | 17268 | 17787 | + |
| scaffold_51 | 17920 | 18657 | + |
| scaffold_51 | 18789 | 19215 | + |
| scaffold_51 | 19316 | 19458 | + |
| scaffold_51 | 19506 | 19553 | + |
| scaffold_51 | 19623 | 19683 | + |
| scaffold_51 | 19780 | 19812 | + |
| scaffold_51 | 19933 | 19946 | + |
| scaffold_51 | 20425 | 20728 | + |
| scaffold_51 | 21008 | 21238 | + |
| scaffold_51 | 21441 | 21804 | + |
| scaffold_51 | 21864 | 21895 | + |
| scaffold_51 | 22024 | 22576 | + |
| scaffold_51 | 22796 | 23792 | + |
| scaffold_51 | 24746 | 25545 | + |
| scaffold_51 | 25650 | 25694 | + |
| scaffold_51 | 26045 | 27392 | + |
| scaffold_51 | 27610 | 27784 | + |
| scaffold_51 | 27877 | 28153 | + |
| scaffold_51 | 28232 | 28263 | + |
| scaffold_51 | 28895 | 28957 | + |
| scaffold_51 | 29295 | 29906 | + |
| scaffold_51 | 30038 | 32092 | + |
| scaffold_51 | 32324 | 35880 | + |
| scaffold_51 | 36036 | 38841 | + |
| scaffold_51 | 39264 | 39439 | + |
| scaffold_51 | 39577 | 39607 | + |
| scaffold_51 | 39714 | 39792 | + |
| scaffold_51 | 39947 | 41658 | + |
| scaffold_51 | 4076 | 4076 | + |
| scaffold_51 | 4138 | 6126 | + |
| scaffold_51 | 41738 | 42774 | + |
| scaffold_51 | 42837 | 50162 | + |
| scaffold_51 | 50318 | 53149 | + |
| scaffold_51 | 53255 | 53382 | + |
| scaffold_51 | 54454 | 54828 | + |
| scaffold_51 | 54916 | 54956 | + |
| scaffold_51 | 55047 | 55470 | + |
| scaffold_51 | 55542 | 55817 | + |
| scaffold_51 | 56122 | 56440 | + |
| scaffold_51 | 56600 | 56634 | + |
| scaffold_51 | 56686 | 57813 | + |
| scaffold_51 | 57846 | 57855 | + |
| scaffold_51 | 57943 | 58628 | + |
| scaffold_51 | 58994 | 59048 | + |
| scaffold_51 | 60176 | 60260 | + |
| scaffold_51 | 60350 | 60486 | + |
| scaffold_51 | 60542 | 61359 | + |
| scaffold_51 | 61418 | 64093 | + |
| scaffold_51 | 6191 | 6793 | + |
| scaffold_51 | 64173 | 65698 | + |
| scaffold_51 | 66209 | 67065 | + |
| scaffold_51 | 67103 | 67921 | + |
| scaffold_51 | 68034 | 68363 | + |
| scaffold_51 | 68405 | 82470 | + |
| scaffold_51 | 6934 | 7224 | + |
| scaffold_51 | 82539 | 83683 | + |
| scaffold_51 | 83770 | 83879 | + |
| scaffold_51 | 83950 | 85881 | + |
| scaffold_51 | 86301 | 94003 | + |
| scaffold_51 | 95242 | 95298 | + |
| scaffold_51 | 95783 | 95873 | + |
| scaffold_51 | 95990 | 96068 | + |
| scaffold_52 | 127793 | 128290 | + |
| scaffold_52 | 128368 | 129798 | + |
| scaffold_52 | 130535 | 136696 | + |
| scaffold_52 | 136767 | 136791 | + |
| scaffold_52 | 137446 | 138181 | + |
| scaffold_52 | 75753 | 77101 | + |
| scaffold_52 | 77323 | 78788 | + |
| scaffold_53 | 115193 | 115194 | + |
| scaffold_53 | 119984 | 120154 | + |
| scaffold_53 | 127511 | 127555 | + |
| scaffold_53 | 1788 | 1918 | + |
| scaffold_53 | 2031 | 6681 | + |
| scaffold_53 | 629 | 1698 | + |
| scaffold_53 | 6835 | 10530 | + |
| scaffold_53 | 91800 | 91831 | + |
| scaffold_53 | 93349 | 93352 | + |
| scaffold_53 | 93392 | 93740 | + |
| scaffold_54 | 103530 | 103531 | + |
| scaffold_54 | 110908 | 110911 | + |
| scaffold_54 | 110951 | 111299 | + |
| scaffold_54 | 111789 | 111789 | + |
| scaffold_54 | 113007 | 113496 | + |
| scaffold_54 | 113528 | 113576 | + |
| scaffold_54 | 114009 | 114101 | + |
| scaffold_54 | 114877 | 115011 | + |
| scaffold_54 | 116197 | 116705 | + |
| scaffold_54 | 116803 | 116845 | + |
| scaffold_54 | 116997 | 118664 | + |
| scaffold_54 | 118977 | 119062 | + |
| scaffold_54 | 119222 | 119278 | + |
| scaffold_54 | 119335 | 119466 | + |
| scaffold_54 | 121035 | 121035 | + |
| scaffold_54 | 123343 | 123434 | + |
| scaffold_54 | 123883 | 124699 | + |
| scaffold_54 | 70030 | 70032 | + |
| scaffold_54 | 7072 | 8118 | + |
| scaffold_54 | 73947 | 73948 | + |
| scaffold_54 | 76220 | 76224 | + |
| scaffold_54 | 8165 | 8190 | + |
| scaffold_54 | 82320 | 82338 | + |
| scaffold_54 | 8322 | 9848 | + |
| scaffold_54 | 83679 | 86218 | + |
| scaffold_55 | 100127 | 100498 | + |
| scaffold_55 | 117771 | 117973 | + |
| scaffold_55 | 42244 | 45694 | + |
| scaffold_55 | 45944 | 46130 | + |
| scaffold_55 | 46238 | 46299 | + |
| scaffold_55 | 46358 | 46496 | + |
| scaffold_55 | 46550 | 47593 | + |
| scaffold_55 | 49219 | 49219 | + |
| scaffold_55 | 51115 | 51972 | + |
| scaffold_55 | 52172 | 52232 | + |
| scaffold_55 | 53103 | 53633 | + |
| scaffold_55 | 53701 | 55908 | + |
| scaffold_55 | 56081 | 56178 | + |
| scaffold_55 | 56701 | 56709 | + |
| scaffold_55 | 96420 | 96442 | + |
| scaffold_55 | 96695 | 96696 | + |
| scaffold_55 | 98596 | 99962 | + |
| scaffold_56 | 113138 | 113197 | + |
| scaffold_56 | 113745 | 113910 | + |
| scaffold_56 | 11556 | 11559 | + |
| scaffold_56 | 12083 | 12099 | + |
| scaffold_56 | 22003 | 22013 | + |
| scaffold_56 | 22997 | 22998 | + |
| scaffold_56 | 43461 | 43473 | + |
| scaffold_56 | 5517 | 5518 | + |
| scaffold_56 | 56 | 66 | + |
| scaffold_56 | 664 | 664 | + |
| scaffold_56 | 85900 | 85901 | + |
| scaffold_56 | 881 | 895 | + |
| scaffold_57 | 25595 | 25598 | + |
| scaffold_57 | 25638 | 25986 | + |
| scaffold_57 | 87747 | 87759 | + |
| scaffold_57 | 88452 | 88452 | + |
| scaffold_57 | 96315 | 96358 | + |
| scaffold_58 | 101580 | 101769 | + |
| scaffold_58 | 101862 | 101945 | + |
| scaffold_58 | 98029 | 101479 | + |
| scaffold_59 | 3328 | 3329 | + |
| scaffold_59 | 3494 | 3496 | + |
| scaffold_59 | 40137 | 40137 | + |
| scaffold_59 | 70474 | 70476 | + |
| scaffold_59 | 86017 | 86018 | + |
| scaffold_59 | 90953 | 91121 | + |
| scaffold_59 | 91390 | 91471 | + |
| scaffold_59 | 97804 | 97809 | + |
| scaffold_6 | 1002296 | 1002303 | + |
| scaffold_6 | 1006377 | 1006378 | + |
| scaffold_6 | 1037166 | 1037169 | + |
| scaffold_6 | 106010 | 107228 | + |
| scaffold_6 | 1071705 | 1071705 | + |
| scaffold_6 | 1071763 | 1071769 | + |
| scaffold_6 | 107562 | 108061 | + |
| scaffold_6 | 1080175 | 1080180 | + |
| scaffold_6 | 1080488 | 1080501 | + |
| scaffold_6 | 108247 | 108330 | + |
| scaffold_6 | 108799 | 109120 | + |
| scaffold_6 | 109694 | 109776 | + |
| scaffold_6 | 109949 | 109961 | + |
| scaffold_6 | 110258 | 110392 | + |
| scaffold_6 | 110657 | 113056 | + |
| scaffold_6 | 113194 | 115227 | + |
| scaffold_6 | 115675 | 116218 | + |
| scaffold_6 | 116314 | 116413 | + |
| scaffold_6 | 116795 | 117077 | + |
| scaffold_6 | 1172795 | 1172831 | + |
| scaffold_6 | 1179772 | 1179914 | + |
| scaffold_6 | 118491 | 118740 | + |
| scaffold_6 | 118890 | 119257 | + |
| scaffold_6 | 119303 | 119430 | + |
| scaffold_6 | 119702 | 119958 | + |
| scaffold_6 | 120052 | 120132 | + |
| scaffold_6 | 120218 | 120242 | + |
| scaffold_6 | 1205634 | 1205635 | + |
| scaffold_6 | 1206488 | 1206490 | + |
| scaffold_6 | 120800 | 120826 | + |
| scaffold_6 | 121795 | 121958 | + |
| scaffold_6 | 122145 | 122238 | + |
| scaffold_6 | 122808 | 123404 | + |
| scaffold_6 | 1229147 | 1229239 | + |
| scaffold_6 | 1266717 | 1266717 | + |
| scaffold_6 | 1266811 | 1266816 | + |
| scaffold_6 | 1276193 | 1276199 | + |
| scaffold_6 | 1305078 | 1305081 | + |
| scaffold_6 | 1346027 | 1346029 | + |
| scaffold_6 | 1352660 | 1352660 | + |
| scaffold_6 | 1456689 | 1456696 | + |
| scaffold_6 | 1531653 | 1531654 | + |
| scaffold_6 | 1531677 | 1531679 | + |
| scaffold_6 | 1535100 | 1535101 | + |
| scaffold_6 | 1535174 | 1535176 | + |
| scaffold_6 | 1543944 | 1543946 | + |
| scaffold_6 | 1629933 | 1630746 | + |
| scaffold_6 | 1630830 | 1632953 | + |
| scaffold_6 | 1687981 | 1688015 | + |
| scaffold_6 | 1717791 | 1717793 | + |
| scaffold_6 | 1717909 | 1717911 | + |
| scaffold_6 | 1791422 | 1791425 | + |
| scaffold_6 | 1791519 | 1791519 | + |
| scaffold_6 | 1820956 | 1820970 | + |
| scaffold_6 | 1829113 | 1829113 | + |
| scaffold_6 | 1829710 | 1829797 | + |
| scaffold_6 | 1838295 | 1838296 | + |
| scaffold_6 | 198268 | 198269 | + |
| scaffold_6 | 209965 | 209977 | + |
| scaffold_6 | 216080 | 216085 | + |
| scaffold_6 | 242615 | 242618 | + |
| scaffold_6 | 26135 | 26135 | + |
| scaffold_6 | 266408 | 266419 | + |
| scaffold_6 | 266688 | 266691 | + |
| scaffold_6 | 340704 | 340711 | + |
| scaffold_6 | 386797 | 386800 | + |
| scaffold_6 | 436499 | 436505 | + |
| scaffold_6 | 482782 | 482784 | + |
| scaffold_6 | 511020 | 511025 | + |
| scaffold_6 | 52017 | 52230 | + |
| scaffold_6 | 527316 | 527325 | + |
| scaffold_6 | 528469 | 528814 | + |
| scaffold_6 | 52904 | 53248 | + |
| scaffold_6 | 54366 | 54494 | + |
| scaffold_6 | 54604 | 55043 | + |
| scaffold_6 | 55121 | 55143 | + |
| scaffold_6 | 55244 | 55289 | + |
| scaffold_6 | 55413 | 55674 | + |
| scaffold_6 | 55773 | 55875 | + |
| scaffold_6 | 55944 | 56050 | + |
| scaffold_6 | 56382 | 57992 | + |
| scaffold_6 | 58090 | 59062 | + |
| scaffold_6 | 60116 | 61074 | + |
| scaffold_6 | 623 | 905 | + |
| scaffold_6 | 634364 | 634364 | + |
| scaffold_6 | 648842 | 648842 | + |
| scaffold_6 | 663103 | 663105 | + |
| scaffold_6 | 663213 | 663221 | + |
| scaffold_6 | 671902 | 671905 | + |
| scaffold_6 | 672285 | 672294 | + |
| scaffold_6 | 676547 | 676547 | + |
| scaffold_6 | 68335 | 68476 | + |
| scaffold_6 | 685486 | 685508 | + |
| scaffold_6 | 68776 | 69346 | + |
| scaffold_6 | 69480 | 69482 | + |
| scaffold_6 | 697355 | 697356 | + |
| scaffold_6 | 72175 | 72176 | + |
| scaffold_6 | 72653 | 72853 | + |
| scaffold_6 | 72994 | 73302 | + |
| scaffold_6 | 734708 | 734708 | + |
| scaffold_6 | 734879 | 734889 | + |
| scaffold_6 | 73503 | 75594 | + |
| scaffold_6 | 749592 | 749595 | + |
| scaffold_6 | 749620 | 749627 | + |
| scaffold_6 | 75663 | 75833 | + |
| scaffold_6 | 75956 | 77045 | + |
| scaffold_6 | 77167 | 78057 | + |
| scaffold_6 | 78239 | 78337 | + |
| scaffold_6 | 78504 | 82831 | + |
| scaffold_6 | 822741 | 822743 | + |
| scaffold_6 | 827294 | 827295 | + |
| scaffold_6 | 83154 | 83224 | + |
| scaffold_6 | 835155 | 835156 | + |
| scaffold_6 | 84620 | 86991 | + |
| scaffold_6 | 857450 | 857454 | + |
| scaffold_6 | 87078 | 87335 | + |
| scaffold_6 | 87448 | 87997 | + |
| scaffold_6 | 89635 | 89846 | + |
| scaffold_6 | 89968 | 92985 | + |
| scaffold_6 | 93061 | 93454 | + |
| scaffold_6 | 93522 | 94008 | + |
| scaffold_6 | 94180 | 94258 | + |
| scaffold_6 | 94299 | 94523 | + |
| scaffold_6 | 94852 | 95321 | + |
| scaffold_6 | 95416 | 95777 | + |
| scaffold_6 | 95958 | 96295 | + |
| scaffold_6 | 96605 | 105973 | + |
| scaffold_6 | 970139 | 970173 | + |
| scaffold_6 | 979171 | 979175 | + |
| scaffold_6 | 994269 | 994269 | + |
| scaffold_60 | 1242 | 1243 | + |
| scaffold_60 | 41572 | 43348 | + |
| scaffold_60 | 43464 | 52351 | + |
| scaffold_60 | 52722 | 53137 | + |
| scaffold_60 | 53273 | 53675 | + |
| scaffold_60 | 53894 | 53908 | + |
| scaffold_60 | 53954 | 54230 | + |
| scaffold_60 | 56044 | 56060 | + |
| scaffold_60 | 56391 | 56729 | + |
| scaffold_60 | 56851 | 58113 | + |
| scaffold_60 | 58624 | 60180 | + |
| scaffold_60 | 60379 | 61008 | + |
| scaffold_60 | 61205 | 62613 | + |
| scaffold_60 | 62873 | 63678 | + |
| scaffold_60 | 64014 | 64097 | + |
| scaffold_60 | 64386 | 65348 | + |
| scaffold_60 | 71916 | 71919 | + |
| scaffold_60 | 71958 | 72384 | + |
| scaffold_60 | 72554 | 73727 | + |
| scaffold_60 | 73865 | 74745 | + |
| scaffold_60 | 74817 | 75080 | + |
| scaffold_60 | 75143 | 75837 | + |
| scaffold_60 | 79627 | 81311 | + |
| scaffold_60 | 83080 | 84238 | + |
| scaffold_60 | 84853 | 84906 | + |
| scaffold_60 | 85402 | 85607 | + |
| scaffold_60 | 86246 | 86362 | + |
| scaffold_60 | 86903 | 86942 | + |
| scaffold_60 | 87966 | 88502 | + |
| scaffold_60 | 90861 | 91415 | + |
| scaffold_60 | 93069 | 93072 | + |
| scaffold_60 | 93238 | 93326 | + |
| scaffold_60 | 94027 | 94301 | + |
| scaffold_61 | 10259 | 16765 | + |
| scaffold_61 | 91113 | 91116 | + |
| scaffold_62 | 1870 | 1875 | + |
| scaffold_62 | 22261 | 22294 | + |
| scaffold_62 | 22398 | 22653 | + |
| scaffold_62 | 22780 | 23108 | + |
| scaffold_62 | 25127 | 25437 | + |
| scaffold_62 | 26324 | 26381 | + |
| scaffold_62 | 27151 | 27169 | + |
| scaffold_62 | 27446 | 27484 | + |
| scaffold_62 | 28571 | 28815 | + |
| scaffold_62 | 29281 | 29693 | + |
| scaffold_62 | 32348 | 32351 | + |
| scaffold_62 | 36267 | 36824 | + |
| scaffold_62 | 3864 | 3882 | + |
| scaffold_62 | 39063 | 39067 | + |
| scaffold_62 | 39275 | 39395 | + |
| scaffold_62 | 39925 | 39941 | + |
| scaffold_62 | 44507 | 44870 | + |
| scaffold_62 | 44926 | 45347 | + |
| scaffold_62 | 45443 | 45641 | + |
| scaffold_62 | 7680 | 13864 | + |
| scaffold_62 | 90203 | 90203 | + |
| scaffold_62 | 91313 | 91342 | + |
| scaffold_62 | 91916 | 91995 | + |
| scaffold_64 | 55702 | 55794 | + |
| scaffold_64 | 64978 | 64985 | + |
| scaffold_64 | 7808 | 7808 | + |
| scaffold_64 | 88750 | 88766 | + |
| scaffold_65 | 10565 | 12046 | + |
| scaffold_65 | 12113 | 13134 | + |
| scaffold_65 | 13382 | 18240 | + |
| scaffold_65 | 18748 | 19424 | + |
| scaffold_65 | 19494 | 19774 | + |
| scaffold_65 | 20596 | 22752 | + |
| scaffold_65 | 22835 | 24080 | + |
| scaffold_65 | 24123 | 24491 | + |
| scaffold_65 | 24874 | 25011 | + |
| scaffold_65 | 25083 | 26127 | + |
| scaffold_65 | 26258 | 27337 | + |
| scaffold_65 | 27611 | 28041 | + |
| scaffold_65 | 30204 | 30631 | + |
| scaffold_65 | 31239 | 31454 | + |
| scaffold_65 | 32106 | 35054 | + |
| scaffold_65 | 35542 | 35679 | + |
| scaffold_65 | 35750 | 36076 | + |
| scaffold_65 | 36259 | 36460 | + |
| scaffold_65 | 36794 | 39891 | + |
| scaffold_65 | 40398 | 40901 | + |
| scaffold_65 | 40977 | 41411 | + |
| scaffold_65 | 43104 | 43774 | + |
| scaffold_65 | 43890 | 44242 | + |
| scaffold_65 | 46124 | 46277 | + |
| scaffold_65 | 46748 | 48042 | + |
| scaffold_65 | 5391 | 6531 | + |
| scaffold_65 | 54467 | 55808 | + |
| scaffold_65 | 56064 | 56064 | + |
| scaffold_65 | 56810 | 57239 | + |
| scaffold_65 | 58233 | 58235 | + |
| scaffold_65 | 58594 | 58600 | + |
| scaffold_65 | 58834 | 59548 | + |
| scaffold_65 | 59689 | 59714 | + |
| scaffold_65 | 59895 | 60616 | + |
| scaffold_65 | 61517 | 61647 | + |
| scaffold_65 | 62773 | 64869 | + |
| scaffold_65 | 65612 | 65693 | + |
| scaffold_65 | 6579 | 7444 | + |
| scaffold_65 | 67960 | 68212 | + |
| scaffold_65 | 69213 | 69699 | + |
| scaffold_65 | 69822 | 73191 | + |
| scaffold_65 | 75206 | 75206 | + |
| scaffold_65 | 7560 | 8666 | + |
| scaffold_65 | 75892 | 78253 | + |
| scaffold_65 | 78409 | 79076 | + |
| scaffold_65 | 79171 | 79538 | + |
| scaffold_65 | 79656 | 80726 | + |
| scaffold_65 | 80948 | 81082 | + |
| scaffold_65 | 81325 | 81333 | + |
| scaffold_65 | 84540 | 84705 | + |
| scaffold_65 | 84841 | 84975 | + |
| scaffold_65 | 8705 | 10468 | + |
| scaffold_66 | 10419 | 20641 | + |
| scaffold_66 | 1563 | 8608 | + |
| scaffold_66 | 22513 | 23018 | + |
| scaffold_66 | 23166 | 23224 | + |
| scaffold_66 | 23292 | 24847 | + |
| scaffold_66 | 25184 | 25207 | + |
| scaffold_66 | 25572 | 25588 | + |
| scaffold_66 | 26586 | 26602 | + |
| scaffold_66 | 26657 | 27957 | + |
| scaffold_66 | 29615 | 30600 | + |
| scaffold_66 | 30964 | 30965 | + |
| scaffold_66 | 31432 | 31433 | + |
| scaffold_66 | 31797 | 31797 | + |
| scaffold_66 | 32750 | 33977 | + |
| scaffold_66 | 34053 | 34410 | + |
| scaffold_66 | 34596 | 36393 | + |
| scaffold_66 | 36852 | 38554 | + |
| scaffold_66 | 40044 | 40994 | + |
| scaffold_66 | 41066 | 41069 | + |
| scaffold_66 | 41378 | 41825 | + |
| scaffold_66 | 41929 | 41971 | + |
| scaffold_66 | 42028 | 43222 | + |
| scaffold_66 | 43312 | 43396 | + |
| scaffold_66 | 43559 | 43641 | + |
| scaffold_66 | 43818 | 43845 | + |
| scaffold_66 | 44376 | 44668 | + |
| scaffold_66 | 44750 | 45138 | + |
| scaffold_66 | 45735 | 45753 | + |
| scaffold_66 | 49249 | 49486 | + |
| scaffold_66 | 49661 | 49994 | + |
| scaffold_66 | 51723 | 54884 | + |
| scaffold_66 | 53 | 65 | + |
| scaffold_66 | 54969 | 55777 | + |
| scaffold_66 | 58014 | 58589 | + |
| scaffold_66 | 58926 | 59460 | + |
| scaffold_66 | 59521 | 59838 | + |
| scaffold_66 | 59930 | 62042 | + |
| scaffold_66 | 62158 | 62391 | + |
| scaffold_66 | 62690 | 62692 | + |
| scaffold_66 | 62965 | 62991 | + |
| scaffold_66 | 63148 | 63917 | + |
| scaffold_66 | 63993 | 64785 | + |
| scaffold_66 | 65023 | 65438 | + |
| scaffold_66 | 65748 | 65799 | + |
| scaffold_66 | 65937 | 67934 | + |
| scaffold_66 | 68015 | 68115 | + |
| scaffold_66 | 68197 | 70373 | + |
| scaffold_66 | 70424 | 71460 | + |
| scaffold_66 | 71532 | 71656 | + |
| scaffold_66 | 71702 | 71842 | + |
| scaffold_66 | 71974 | 74744 | + |
| scaffold_66 | 74868 | 74880 | + |
| scaffold_66 | 75132 | 75361 | + |
| scaffold_66 | 75592 | 76829 | + |
| scaffold_66 | 77063 | 77237 | + |
| scaffold_66 | 77450 | 77689 | + |
| scaffold_66 | 79962 | 80213 | + |
| scaffold_66 | 80261 | 80350 | + |
| scaffold_66 | 80444 | 80723 | + |
| scaffold_66 | 80771 | 80789 | + |
| scaffold_66 | 81131 | 81150 | + |
| scaffold_66 | 83815 | 83876 | + |
| scaffold_66 | 8682 | 9937 | + |
| scaffold_67 | 82736 | 82754 | + |
| scaffold_68 | 17133 | 17141 | + |
| scaffold_68 | 19895 | 20911 | + |
| scaffold_68 | 21585 | 21645 | + |
| scaffold_68 | 21763 | 23521 | + |
| scaffold_68 | 29654 | 29767 | + |
| scaffold_68 | 30778 | 31003 | + |
| scaffold_68 | 31100 | 31463 | + |
| scaffold_69 | 70735 | 70752 | + |
| scaffold_69 | 71076 | 71102 | + |
| scaffold_69 | 71484 | 75503 | + |
| scaffold_69 | 7358 | 7358 | + |
| scaffold_69 | 75546 | 75596 | + |
| scaffold_69 | 75788 | 76138 | + |
| scaffold_69 | 76278 | 76598 | + |
| scaffold_69 | 77397 | 77986 | + |
| scaffold_7 | 1039186 | 1039201 | + |
| scaffold_7 | 1179793 | 1179795 | + |
| scaffold_7 | 1197531 | 1197533 | + |
| scaffold_7 | 1233059 | 1233059 | + |
| scaffold_7 | 1233078 | 1233080 | + |
| scaffold_7 | 1272391 | 1272397 | + |
| scaffold_7 | 1291817 | 1291848 | + |
| scaffold_7 | 1395059 | 1395059 | + |
| scaffold_7 | 1440075 | 1440077 | + |
| scaffold_7 | 1440165 | 1440170 | + |
| scaffold_7 | 1616605 | 1616648 | + |
| scaffold_7 | 1625704 | 1626118 | + |
| scaffold_7 | 1641237 | 1641861 | + |
| scaffold_7 | 1642322 | 1642919 | + |
| scaffold_7 | 1645123 | 1645125 | + |
| scaffold_7 | 1654966 | 1654968 | + |
| scaffold_7 | 1655643 | 1655643 | + |
| scaffold_7 | 1714859 | 1714860 | + |
| scaffold_7 | 1726782 | 1726792 | + |
| scaffold_7 | 1730192 | 1730192 | + |
| scaffold_7 | 1803246 | 1803246 | + |
| scaffold_7 | 1814675 | 1817415 | + |
| scaffold_7 | 1819004 | 1819761 | + |
| scaffold_7 | 183094 | 183099 | + |
| scaffold_7 | 183121 | 183124 | + |
| scaffold_7 | 18759 | 19753 | + |
| scaffold_7 | 250436 | 250437 | + |
| scaffold_7 | 32808 | 32808 | + |
| scaffold_7 | 352070 | 352071 | + |
| scaffold_7 | 36696 | 36696 | + |
| scaffold_7 | 38768 | 38768 | + |
| scaffold_7 | 426988 | 426991 | + |
| scaffold_7 | 44161 | 44375 | + |
| scaffold_7 | 44575 | 45947 | + |
| scaffold_7 | 452298 | 452571 | + |
| scaffold_7 | 50223 | 50223 | + |
| scaffold_7 | 527843 | 527846 | + |
| scaffold_7 | 529869 | 529882 | + |
| scaffold_7 | 531527 | 531530 | + |
| scaffold_7 | 531653 | 531664 | + |
| scaffold_7 | 565713 | 565720 | + |
| scaffold_7 | 565836 | 565844 | + |
| scaffold_7 | 569056 | 569168 | + |
| scaffold_7 | 570331 | 570528 | + |
| scaffold_7 | 632160 | 632164 | + |
| scaffold_7 | 661902 | 661903 | + |
| scaffold_7 | 72439 | 72442 | + |
| scaffold_7 | 731789 | 731797 | + |
| scaffold_7 | 74319 | 74407 | + |
| scaffold_7 | 746373 | 746373 | + |
| scaffold_7 | 76558 | 76579 | + |
| scaffold_7 | 782664 | 782665 | + |
| scaffold_7 | 789112 | 789114 | + |
| scaffold_7 | 83199 | 83590 | + |
| scaffold_7 | 87858 | 87868 | + |
| scaffold_7 | 96975 | 97067 | + |
| scaffold_7 | 972588 | 972590 | + |
| scaffold_7 | 978070 | 978080 | + |
| scaffold_7 | 978177 | 978180 | + |
| scaffold_7 | 99711 | 99864 | + |
| scaffold_70 | 1 | 1 | + |
| scaffold_70 | 10468 | 18498 | + |
| scaffold_70 | 18659 | 22634 | + |
| scaffold_70 | 22770 | 22771 | + |
| scaffold_70 | 22809 | 26421 | + |
| scaffold_70 | 26503 | 27768 | + |
| scaffold_70 | 2757 | 2835 | + |
| scaffold_70 | 27816 | 28704 | + |
| scaffold_70 | 28834 | 29073 | + |
| scaffold_70 | 29155 | 29777 | + |
| scaffold_70 | 30219 | 30300 | + |
| scaffold_70 | 31842 | 31868 | + |
| scaffold_70 | 32886 | 32946 | + |
| scaffold_70 | 35036 | 35037 | + |
| scaffold_70 | 3659 | 3719 | + |
| scaffold_70 | 3846 | 3872 | + |
| scaffold_70 | 3945 | 3955 | + |
| scaffold_70 | 4012 | 4046 | + |
| scaffold_70 | 41097 | 41530 | + |
| scaffold_70 | 4145 | 5768 | + |
| scaffold_70 | 44073 | 44435 | + |
| scaffold_70 | 44478 | 44735 | + |
| scaffold_70 | 44811 | 44826 | + |
| scaffold_70 | 45260 | 45287 | + |
| scaffold_70 | 48727 | 48855 | + |
| scaffold_70 | 48959 | 49018 | + |
| scaffold_70 | 49076 | 49133 | + |
| scaffold_70 | 49215 | 49793 | + |
| scaffold_70 | 50212 | 56097 | + |
| scaffold_70 | 56173 | 56608 | + |
| scaffold_70 | 56954 | 57853 | + |
| scaffold_70 | 5916 | 6817 | + |
| scaffold_70 | 60238 | 61630 | + |
| scaffold_70 | 626 | 2530 | + |
| scaffold_70 | 66439 | 67588 | + |
| scaffold_70 | 67655 | 67664 | + |
| scaffold_70 | 7134 | 7286 | + |
| scaffold_70 | 7556 | 9136 | + |
| scaffold_70 | 9304 | 10393 | + |
| scaffold_71 | 13390 | 13543 | + |
| scaffold_71 | 36578 | 37839 | + |
| scaffold_72 | 40581 | 40584 | + |
| scaffold_72 | 63537 | 63550 | + |
| scaffold_72 | 67097 | 67105 | + |
| scaffold_72 | 8826 | 8842 | + |
| scaffold_74 | 19419 | 19511 | + |
| scaffold_74 | 22889 | 22889 | + |
| scaffold_74 | 23274 | 23435 | + |
| scaffold_74 | 24904 | 25485 | + |
| scaffold_74 | 25703 | 25762 | + |
| scaffold_74 | 26232 | 26281 | + |
| scaffold_74 | 26881 | 27266 | + |
| scaffold_74 | 30929 | 30935 | + |
| scaffold_74 | 49831 | 49833 | + |
| scaffold_74 | 49994 | 50048 | + |
| scaffold_74 | 50122 | 50943 | + |
| scaffold_74 | 51230 | 52290 | + |
| scaffold_74 | 5402 | 5684 | + |
| scaffold_74 | 54385 | 54538 | + |
| scaffold_74 | 62153 | 62399 | + |
| scaffold_74 | 62561 | 63049 | + |
| scaffold_75 | 12999 | 15036 | + |
| scaffold_75 | 15603 | 15603 | + |
| scaffold_75 | 19578 | 19579 | + |
| scaffold_75 | 21909 | 22300 | + |
| scaffold_75 | 22387 | 22388 | + |
| scaffold_75 | 22811 | 22899 | + |
| scaffold_75 | 24484 | 24569 | + |
| scaffold_75 | 24617 | 24728 | + |
| scaffold_75 | 24837 | 25107 | + |
| scaffold_75 | 25175 | 25308 | + |
| scaffold_75 | 25385 | 25622 | + |
| scaffold_75 | 25689 | 25818 | + |
| scaffold_75 | 25895 | 26696 | + |
| scaffold_75 | 28900 | 29550 | + |
| scaffold_75 | 29816 | 29818 | + |
| scaffold_75 | 3363 | 3476 | + |
| scaffold_75 | 37842 | 38556 | + |
| scaffold_75 | 38719 | 43072 | + |
| scaffold_75 | 55113 | 56244 | + |
| scaffold_75 | 5768 | 5858 | + |
| scaffold_75 | 57763 | 57856 | + |
| scaffold_75 | 58753 | 59602 | + |
| scaffold_75 | 59674 | 60054 | + |
| scaffold_75 | 61 | 204 | + |
| scaffold_75 | 62993 | 63003 | + |
| scaffold_75 | 8019 | 8040 | + |
| scaffold_75 | 8090 | 8102 | + |
| scaffold_75 | 8309 | 12814 | + |
| scaffold_76 | 10245 | 10283 | + |
| scaffold_76 | 10501 | 10753 | + |
| scaffold_76 | 10917 | 14146 | + |
| scaffold_76 | 14466 | 14518 | + |
| scaffold_76 | 16605 | 16944 | + |
| scaffold_76 | 17043 | 17125 | + |
| scaffold_76 | 17271 | 17312 | + |
| scaffold_76 | 17398 | 17716 | + |
| scaffold_76 | 17967 | 18811 | + |
| scaffold_76 | 18959 | 19774 | + |
| scaffold_76 | 217 | 250 | + |
| scaffold_76 | 22383 | 23223 | + |
| scaffold_76 | 23284 | 29925 | + |
| scaffold_76 | 288 | 322 | + |
| scaffold_76 | 39335 | 39336 | + |
| scaffold_76 | 4843 | 4870 | + |
| scaffold_76 | 5207 | 5358 | + |
| scaffold_76 | 54352 | 54468 | + |
| scaffold_76 | 54582 | 54596 | + |
| scaffold_76 | 54976 | 55056 | + |
| scaffold_76 | 55099 | 55545 | + |
| scaffold_76 | 5547 | 5671 | + |
| scaffold_76 | 55587 | 56404 | + |
| scaffold_76 | 56462 | 56516 | + |
| scaffold_76 | 57586 | 57586 | + |
| scaffold_76 | 5766 | 6142 | + |
| scaffold_76 | 58183 | 58270 | + |
| scaffold_76 | 59448 | 59454 | + |
| scaffold_76 | 60345 | 60399 | + |
| scaffold_76 | 703 | 1467 | + |
| scaffold_76 | 7337 | 7793 | + |
| scaffold_76 | 7864 | 8443 | + |
| scaffold_76 | 8920 | 8942 | + |
| scaffold_77 | 10558 | 10808 | + |
| scaffold_77 | 11213 | 11277 | + |
| scaffold_77 | 12884 | 12940 | + |
| scaffold_77 | 13732 | 13815 | + |
| scaffold_77 | 13901 | 13922 | + |
| scaffold_77 | 13978 | 14460 | + |
| scaffold_77 | 14775 | 14775 | + |
| scaffold_77 | 15599 | 16736 | + |
| scaffold_77 | 16929 | 17158 | + |
| scaffold_77 | 17464 | 17482 | + |
| scaffold_77 | 17832 | 19207 | + |
| scaffold_77 | 1834 | 2113 | + |
| scaffold_77 | 19804 | 27155 | + |
| scaffold_77 | 2227 | 2233 | + |
| scaffold_77 | 2544 | 3631 | + |
| scaffold_77 | 269 | 1629 | + |
| scaffold_77 | 27324 | 34159 | + |
| scaffold_77 | 34213 | 34804 | + |
| scaffold_77 | 35008 | 35145 | + |
| scaffold_77 | 35217 | 36300 | + |
| scaffold_77 | 36359 | 37438 | + |
| scaffold_77 | 37712 | 38422 | + |
| scaffold_77 | 50652 | 50653 | + |
| scaffold_77 | 5866 | 5911 | + |
| scaffold_77 | 6820 | 7155 | + |
| scaffold_77 | 7595 | 8080 | + |
| scaffold_77 | 8547 | 8626 | + |
| scaffold_77 | 9219 | 10081 | + |
| scaffold_78 | 10204 | 10205 | + |
| scaffold_78 | 10672 | 10673 | + |
| scaffold_78 | 11140 | 11141 | + |
| scaffold_78 | 11608 | 11609 | + |
| scaffold_78 | 12076 | 12077 | + |
| scaffold_78 | 12544 | 12549 | + |
| scaffold_78 | 12898 | 16693 | + |
| scaffold_78 | 18567 | 18720 | + |
| scaffold_78 | 19156 | 23026 | + |
| scaffold_78 | 23523 | 23730 | + |
| scaffold_78 | 23780 | 23817 | + |
| scaffold_78 | 23902 | 24427 | + |
| scaffold_78 | 24506 | 27273 | + |
| scaffold_78 | 27815 | 29433 | + |
| scaffold_78 | 35429 | 35955 | + |
| scaffold_78 | 36257 | 36330 | + |
| scaffold_78 | 36404 | 36719 | + |
| scaffold_78 | 36992 | 37215 | + |
| scaffold_78 | 38148 | 39384 | + |
| scaffold_78 | 39435 | 39504 | + |
| scaffold_78 | 39915 | 43734 | + |
| scaffold_78 | 44235 | 46385 | + |
| scaffold_78 | 46734 | 46803 | + |
| scaffold_78 | 47055 | 47199 | + |
| scaffold_78 | 47941 | 48031 | + |
| scaffold_78 | 48102 | 48107 | + |
| scaffold_78 | 7016 | 7066 | + |
| scaffold_78 | 7503 | 7517 | + |
| scaffold_78 | 8332 | 8333 | + |
| scaffold_78 | 8800 | 8801 | + |
| scaffold_78 | 9268 | 9269 | + |
| scaffold_78 | 9736 | 9737 | + |
| scaffold_79 | 21171 | 21187 | + |
| scaffold_79 | 24078 | 24083 | + |
| scaffold_79 | 24287 | 24291 | + |
| scaffold_79 | 32780 | 33221 | + |
| scaffold_79 | 33303 | 33449 | + |
| scaffold_79 | 33502 | 36527 | + |
| scaffold_79 | 36719 | 38521 | + |
| scaffold_79 | 38721 | 38748 | + |
| scaffold_79 | 41567 | 41577 | + |
| scaffold_79 | 46995 | 47005 | + |
| scaffold_79 | 47342 | 47358 | + |
| scaffold_79 | 6928 | 7334 | + |
| scaffold_79 | 9910 | 11629 | + |
| scaffold_8 | 1013882 | 1013983 | + |
| scaffold_8 | 1160899 | 1160904 | + |
| scaffold_8 | 1205012 | 1205013 | + |
| scaffold_8 | 121366 | 121368 | + |
| scaffold_8 | 1220616 | 1220620 | + |
| scaffold_8 | 135324 | 135326 | + |
| scaffold_8 | 1383456 | 1383456 | + |
| scaffold_8 | 1414343 | 1415046 | + |
| scaffold_8 | 1453474 | 1453480 | + |
| scaffold_8 | 1453613 | 1453625 | + |
| scaffold_8 | 1508991 | 1508991 | + |
| scaffold_8 | 1550842 | 1550848 | + |
| scaffold_8 | 1597743 | 1597743 | + |
| scaffold_8 | 1607886 | 1607923 | + |
| scaffold_8 | 1608422 | 1608432 | + |
| scaffold_8 | 1613849 | 1613862 | + |
| scaffold_8 | 1670898 | 1670898 | + |
| scaffold_8 | 1681750 | 1681762 | + |
| scaffold_8 | 1681838 | 1681843 | + |
| scaffold_8 | 1709806 | 1709812 | + |
| scaffold_8 | 1719862 | 1721462 | + |
| scaffold_8 | 1733241 | 1733244 | + |
| scaffold_8 | 1736255 | 1736263 | + |
| scaffold_8 | 1738962 | 1738964 | + |
| scaffold_8 | 1739158 | 1739161 | + |
| scaffold_8 | 1750243 | 1750244 | + |
| scaffold_8 | 1750704 | 1750716 | + |
| scaffold_8 | 1751188 | 1751214 | + |
| scaffold_8 | 1751393 | 1751499 | + |
| scaffold_8 | 1752714 | 1752715 | + |
| scaffold_8 | 1753182 | 1753183 | + |
| scaffold_8 | 1753650 | 1753651 | + |
| scaffold_8 | 1754118 | 1754119 | + |
| scaffold_8 | 1754586 | 1754587 | + |
| scaffold_8 | 1755054 | 1755055 | + |
| scaffold_8 | 1755522 | 1755523 | + |
| scaffold_8 | 1755990 | 1755991 | + |
| scaffold_8 | 1756458 | 1756459 | + |
| scaffold_8 | 1756926 | 1756927 | + |
| scaffold_8 | 1757394 | 1757395 | + |
| scaffold_8 | 1757888 | 1757916 | + |
| scaffold_8 | 1758007 | 1760754 | + |
| scaffold_8 | 1761111 | 1761183 | + |
| scaffold_8 | 1761216 | 1762007 | + |
| scaffold_8 | 1763355 | 1764204 | + |
| scaffold_8 | 1764821 | 1765842 | + |
| scaffold_8 | 1766112 | 1766243 | + |
| scaffold_8 | 1766701 | 1767715 | + |
| scaffold_8 | 1767878 | 1767971 | + |
| scaffold_8 | 1768096 | 1768188 | + |
| scaffold_8 | 1768275 | 1768427 | + |
| scaffold_8 | 1770098 | 1772491 | + |
| scaffold_8 | 1772992 | 1773238 | + |
| scaffold_8 | 1773676 | 1773784 | + |
| scaffold_8 | 1773935 | 1774512 | + |
| scaffold_8 | 1774680 | 1776044 | + |
| scaffold_8 | 1783828 | 1783981 | + |
| scaffold_8 | 180752 | 180755 | + |
| scaffold_8 | 180980 | 180989 | + |
| scaffold_8 | 20445 | 20445 | + |
| scaffold_8 | 236789 | 236790 | + |
| scaffold_8 | 237130 | 237131 | + |
| scaffold_8 | 263298 | 263299 | + |
| scaffold_8 | 263525 | 263531 | + |
| scaffold_8 | 322540 | 322550 | + |
| scaffold_8 | 392923 | 392924 | + |
| scaffold_8 | 429448 | 429458 | + |
| scaffold_8 | 474201 | 474202 | + |
| scaffold_8 | 474559 | 474561 | + |
| scaffold_8 | 516829 | 516837 | + |
| scaffold_8 | 712230 | 712333 | + |
| scaffold_8 | 743330 | 743341 | + |
| scaffold_8 | 812721 | 812722 | + |
| scaffold_8 | 922896 | 922899 | + |
| scaffold_8 | 985083 | 985086 | + |
| scaffold_80 | 21333 | 21334 | + |
| scaffold_80 | 34154 | 34155 | + |
| scaffold_80 | 34904 | 34904 | + |
| scaffold_80 | 36323 | 36323 | + |
| scaffold_80 | 38614 | 38725 | + |
| scaffold_80 | 39424 | 40418 | + |
| scaffold_80 | 44861 | 44866 | + |
| scaffold_81 | 10265 | 12118 | + |
| scaffold_81 | 1307 | 1355 | + |
| scaffold_81 | 17310 | 17311 | + |
| scaffold_81 | 17419 | 17421 | + |
| scaffold_81 | 18637 | 18897 | + |
| scaffold_81 | 23129 | 23929 | + |
| scaffold_81 | 25080 | 25176 | + |
| scaffold_81 | 27202 | 27204 | + |
| scaffold_81 | 28213 | 28334 | + |
| scaffold_81 | 28410 | 28446 | + |
| scaffold_81 | 28649 | 28658 | + |
| scaffold_81 | 28779 | 28781 | + |
| scaffold_81 | 28845 | 28854 | + |
| scaffold_81 | 32764 | 33176 | + |
| scaffold_81 | 33642 | 34766 | + |
| scaffold_81 | 36635 | 37996 | + |
| scaffold_81 | 38081 | 38905 | + |
| scaffold_81 | 39235 | 39810 | + |
| scaffold_81 | 40030 | 40033 | + |
| scaffold_81 | 42334 | 43307 | + |
| scaffold_81 | 43427 | 43505 | + |
| scaffold_81 | 5168 | 5281 | + |
| scaffold_81 | 582 | 1090 | + |
| scaffold_81 | 91 | 239 | + |
| scaffold_82 | 15927 | 15928 | + |
| scaffold_82 | 17160 | 17572 | + |
| scaffold_82 | 39819 | 39820 | + |
| scaffold_82 | 594 | 610 | + |
| scaffold_83 | 12244 | 12504 | + |
| scaffold_83 | 13727 | 14501 | + |
| scaffold_83 | 17278 | 17538 | + |
| scaffold_83 | 18714 | 19535 | + |
| scaffold_83 | 20413 | 20922 | + |
| scaffold_83 | 20973 | 21079 | + |
| scaffold_83 | 21161 | 22437 | + |
| scaffold_83 | 25390 | 25391 | + |
| scaffold_83 | 25858 | 25859 | + |
| scaffold_83 | 26326 | 26327 | + |
| scaffold_83 | 26794 | 26795 | + |
| scaffold_83 | 26841 | 27199 | + |
| scaffold_83 | 8847 | 10452 | + |
| scaffold_84 | 10280 | 10311 | + |
| scaffold_84 | 10684 | 10758 | + |
| scaffold_84 | 24993 | 24994 | + |
| scaffold_84 | 27913 | 27933 | + |
| scaffold_84 | 27993 | 28256 | + |
| scaffold_84 | 28431 | 28509 | + |
| scaffold_84 | 29019 | 29217 | + |
| scaffold_84 | 29372 | 30037 | + |
| scaffold_84 | 30240 | 30579 | + |
| scaffold_84 | 30727 | 30881 | + |
| scaffold_84 | 31005 | 31520 | + |
| scaffold_84 | 31761 | 31953 | + |
| scaffold_84 | 32507 | 32523 | + |
| scaffold_84 | 37197 | 38410 | + |
| scaffold_84 | 38499 | 39980 | + |
| scaffold_84 | 4223 | 7055 | + |
| scaffold_84 | 56 | 189 | + |
| scaffold_84 | 7136 | 7374 | + |
| scaffold_84 | 735 | 4131 | + |
| scaffold_85 | 22130 | 22489 | + |
| scaffold_86 | 8475 | 8478 | + |
| scaffold_87 | 11890 | 11899 | + |
| scaffold_87 | 1478 | 1500 | + |
| scaffold_87 | 14865 | 14968 | + |
| scaffold_87 | 15196 | 15352 | + |
| scaffold_87 | 15845 | 15896 | + |
| scaffold_87 | 15981 | 16410 | + |
| scaffold_87 | 1641 | 2010 | + |
| scaffold_87 | 16584 | 16856 | + |
| scaffold_87 | 17060 | 17359 | + |
| scaffold_87 | 18290 | 18640 | + |
| scaffold_87 | 18798 | 18990 | + |
| scaffold_87 | 19059 | 20788 | + |
| scaffold_87 | 21250 | 21530 | + |
| scaffold_87 | 23315 | 23712 | + |
| scaffold_87 | 23961 | 24469 | + |
| scaffold_87 | 24930 | 25654 | + |
| scaffold_87 | 25716 | 26038 | + |
| scaffold_87 | 26087 | 26493 | + |
| scaffold_87 | 29112 | 30740 | + |
| scaffold_87 | 30838 | 30983 | + |
| scaffold_87 | 31023 | 31825 | + |
| scaffold_87 | 31944 | 35690 | + |
| scaffold_87 | 4128 | 4254 | + |
| scaffold_88 | 11682 | 11769 | + |
| scaffold_88 | 15064 | 15489 | + |
| scaffold_88 | 15703 | 15788 | + |
| scaffold_88 | 15961 | 19128 | + |
| scaffold_88 | 28635 | 28661 | + |
| scaffold_88 | 28761 | 31958 | + |
| scaffold_88 | 32474 | 32529 | + |
| scaffold_88 | 33299 | 33323 | + |
| scaffold_88 | 33419 | 33430 | + |
| scaffold_88 | 33498 | 33890 | + |
| scaffold_88 | 33995 | 34013 | + |
| scaffold_88 | 34116 | 34170 | + |
| scaffold_88 | 34329 | 34443 | + |
| scaffold_88 | 34555 | 34556 | + |
| scaffold_88 | 34609 | 34615 | + |
| scaffold_88 | 35059 | 35059 | + |
| scaffold_88 | 35242 | 35293 | + |
| scaffold_88 | 35410 | 35498 | + |
| scaffold_88 | 4726 | 5450 | + |
| scaffold_88 | 5512 | 5834 | + |
| scaffold_88 | 6042 | 6289 | + |
| scaffold_88 | 6917 | 6926 | + |
| scaffold_88 | 8357 | 8662 | + |
| scaffold_88 | 8716 | 9566 | + |
| scaffold_88 | 9624 | 9681 | + |
| scaffold_89 | 14921 | 14921 | + |
| scaffold_89 | 20752 | 20752 | + |
| scaffold_89 | 23687 | 23845 | + |
| scaffold_89 | 26611 | 30178 | + |
| scaffold_89 | 30408 | 31608 | + |
| scaffold_89 | 31656 | 32099 | + |
| scaffold_89 | 32176 | 32207 | + |
| scaffold_89 | 32434 | 32435 | + |
| scaffold_89 | 32521 | 32951 | + |
| scaffold_89 | 33589 | 33840 | + |
| scaffold_89 | 33897 | 34901 | + |
| scaffold_89 | 35009 | 35162 | + |
| scaffold_89 | 5898 | 6881 | + |
| scaffold_9 | 1000108 | 1000109 | + |
| scaffold_9 | 1018760 | 1018813 | + |
| scaffold_9 | 10406 | 10565 | + |
| scaffold_9 | 10945 | 10945 | + |
| scaffold_9 | 1145474 | 1145494 | + |
| scaffold_9 | 1150708 | 1150710 | + |
| scaffold_9 | 1150863 | 1150873 | + |
| scaffold_9 | 1178934 | 1178945 | + |
| scaffold_9 | 1300312 | 1300312 | + |
| scaffold_9 | 1474895 | 1474897 | + |
| scaffold_9 | 1478544 | 1478551 | + |
| scaffold_9 | 1478576 | 1478576 | + |
| scaffold_9 | 1565068 | 1565077 | + |
| scaffold_9 | 15811 | 15817 | + |
| scaffold_9 | 1590855 | 1590859 | + |
| scaffold_9 | 1590871 | 1590874 | + |
| scaffold_9 | 1613264 | 1613351 | + |
| scaffold_9 | 16548 | 16551 | + |
| scaffold_9 | 180105 | 180106 | + |
| scaffold_9 | 286739 | 286744 | + |
| scaffold_9 | 30585 | 30592 | + |
| scaffold_9 | 423034 | 423036 | + |
| scaffold_9 | 452556 | 453444 | + |
| scaffold_9 | 459068 | 459676 | + |
| scaffold_9 | 485926 | 485944 | + |
| scaffold_9 | 527478 | 527480 | + |
| scaffold_9 | 527508 | 527508 | + |
| scaffold_9 | 55 | 5657 | + |
| scaffold_9 | 576534 | 576552 | + |
| scaffold_9 | 5887 | 5941 | + |
| scaffold_9 | 601845 | 601854 | + |
| scaffold_9 | 6206 | 6211 | + |
| scaffold_9 | 6414 | 6631 | + |
| scaffold_9 | 7143 | 7223 | + |
| scaffold_9 | 7500 | 7593 | + |
| scaffold_9 | 7671 | 8944 | + |
| scaffold_9 | 770332 | 770368 | + |
| scaffold_9 | 771872 | 771873 | + |
| scaffold_9 | 812125 | 812126 | + |
| scaffold_9 | 82238 | 82238 | + |
| scaffold_9 | 948166 | 948166 | + |
| scaffold_9 | 953314 | 953338 | + |
| scaffold_9 | 954555 | 954559 | + |
| scaffold_9 | 969223 | 969225 | + |
| scaffold_9 | 969236 | 969243 | + |
| scaffold_90 | 22391 | 22453 | + |
| scaffold_90 | 25550 | 25653 | + |
| scaffold_90 | 25734 | 26437 | + |
| scaffold_90 | 26656 | 27403 | + |
| scaffold_90 | 27594 | 27651 | + |
| scaffold_91 | 10137 | 16101 | + |
| scaffold_91 | 16485 | 16655 | + |
| scaffold_91 | 16774 | 17576 | + |
| scaffold_91 | 17616 | 17761 | + |
| scaffold_91 | 17859 | 18174 | + |
| scaffold_91 | 18262 | 19459 | + |
| scaffold_91 | 19567 | 19712 | + |
| scaffold_91 | 22842 | 23089 | + |
| scaffold_91 | 23297 | 23619 | + |
| scaffold_91 | 23681 | 24405 | + |
| scaffold_91 | 24866 | 25330 | + |
| scaffold_91 | 26756 | 26764 | + |
| scaffold_91 | 27130 | 27175 | + |
| scaffold_91 | 27557 | 27626 | + |
| scaffold_91 | 27972 | 28278 | + |
| scaffold_91 | 28412 | 28723 | + |
| scaffold_91 | 28851 | 28894 | + |
| scaffold_91 | 29420 | 29436 | + |
| scaffold_91 | 29915 | 30060 | + |
| scaffold_91 | 30732 | 30799 | + |
| scaffold_91 | 31021 | 31049 | + |
| scaffold_91 | 31134 | 31315 | + |
| scaffold_91 | 31571 | 31819 | + |
| scaffold_91 | 31918 | 32051 | + |
| scaffold_91 | 32137 | 32160 | + |
| scaffold_91 | 32309 | 32317 | + |
| scaffold_91 | 3540 | 3551 | + |
| scaffold_91 | 3806 | 4173 | + |
| scaffold_91 | 4864 | 4966 | + |
| scaffold_91 | 5005 | 5443 | + |
| scaffold_91 | 5488 | 8485 | + |
| scaffold_91 | 8765 | 9489 | + |
| scaffold_93 | 11482 | 11486 | + |
| scaffold_93 | 11574 | 11734 | + |
| scaffold_93 | 13552 | 13807 | + |
| scaffold_93 | 13965 | 14182 | + |
| scaffold_93 | 1594 | 1594 | + |
| scaffold_93 | 16056 | 17221 | + |
| scaffold_93 | 1669 | 2376 | + |
| scaffold_93 | 21592 | 21660 | + |
| scaffold_93 | 21758 | 22171 | + |
| scaffold_93 | 22404 | 22790 | + |
| scaffold_93 | 24445 | 24565 | + |
| scaffold_93 | 24789 | 26889 | + |
| scaffold_93 | 2640 | 2652 | + |
| scaffold_93 | 2935 | 3114 | + |
| scaffold_93 | 6549 | 6607 | + |
| scaffold_93 | 7279 | 7504 | + |
| scaffold_93 | 7760 | 7760 | + |
| scaffold_93 | 8441 | 8444 | + |
| scaffold_94 | 1 | 29 | + |
| scaffold_94 | 11259 | 11824 | + |
| scaffold_94 | 12395 | 12575 | + |
| scaffold_94 | 13287 | 14101 | + |
| scaffold_94 | 1534 | 1750 | + |
| scaffold_94 | 2166 | 2227 | + |
| scaffold_94 | 2294 | 2502 | + |
| scaffold_94 | 24304 | 24305 | + |
| scaffold_94 | 24772 | 24773 | + |
| scaffold_94 | 25240 | 25241 | + |
| scaffold_94 | 25708 | 25709 | + |
| scaffold_94 | 26176 | 26177 | + |
| scaffold_94 | 2648 | 3201 | + |
| scaffold_94 | 26644 | 26645 | + |
| scaffold_94 | 27112 | 27113 | + |
| scaffold_94 | 27580 | 27581 | + |
| scaffold_94 | 28048 | 28049 | + |
| scaffold_94 | 28516 | 28517 | + |
| scaffold_94 | 28984 | 28985 | + |
| scaffold_94 | 29452 | 29453 | + |
| scaffold_94 | 29790 | 30313 | + |
| scaffold_94 | 30529 | 30563 | + |
| scaffold_94 | 30621 | 30632 | + |
| scaffold_94 | 30774 | 30848 | + |
| scaffold_94 | 30900 | 31070 | + |
| scaffold_94 | 445 | 506 | + |
| scaffold_94 | 573 | 781 | + |
| scaffold_94 | 927 | 1480 | + |
| scaffold_95 | 1 | 2461 | + |
| scaffold_95 | 10112 | 10188 | + |
| scaffold_95 | 10653 | 13630 | + |
| scaffold_95 | 13809 | 15915 | + |
| scaffold_95 | 16035 | 18410 | + |
| scaffold_95 | 19572 | 19713 | + |
| scaffold_95 | 22654 | 22654 | + |
| scaffold_95 | 23171 | 23539 | + |
| scaffold_95 | 23570 | 27315 | + |
| scaffold_95 | 4515 | 4515 | + |
| scaffold_95 | 5330 | 5830 | + |
| scaffold_95 | 6604 | 6697 | + |
| scaffold_95 | 8187 | 9749 | + |
| scaffold_95 | 9838 | 10069 | + |
| scaffold_96 | 18481 | 18481 | + |
| scaffold_96 | 22358 | 22789 | + |
| scaffold_97 | 15845 | 15871 | + |
| scaffold_97 | 23443 | 24580 | + |
| scaffold_97 | 25217 | 25880 | + |
| scaffold_97 | 26072 | 26717 | + |
| scaffold_97 | 26972 | 27141 | + |
| scaffold_97 | 27323 | 27558 | + |
| scaffold_97 | 27766 | 27887 | + |
| scaffold_97 | 28880 | 28982 | + |
| scaffold_97 | 29021 | 29147 | + |
| scaffold_97 | 29224 | 29263 | + |
| scaffold_97 | 29356 | 29874 | + |
| scaffold_97 | 5238 | 5415 | + |
| scaffold_97 | 5624 | 5989 | + |
| scaffold_97 | 660 | 883 | + |
| scaffold_98 | 1030 | 2604 | + |
| scaffold_98 | 11579 | 11579 | + |
| scaffold_98 | 11670 | 12017 | + |
| scaffold_98 | 12683 | 12826 | + |
| scaffold_98 | 13592 | 15292 | + |
| scaffold_98 | 20807 | 22919 | + |
| scaffold_98 | 23060 | 23148 | + |
| scaffold_98 | 23201 | 23394 | + |
| scaffold_98 | 23495 | 24127 | + |
| scaffold_98 | 236 | 244 | + |
| scaffold_98 | 24208 | 25062 | + |
| scaffold_98 | 25192 | 25528 | + |
| scaffold_98 | 27792 | 27847 | + |
| scaffold_98 | 27945 | 28775 | + |
| scaffold_98 | 29032 | 29050 | + |
| scaffold_98 | 3064 | 4346 | + |
| scaffold_98 | 8652 | 8684 | + |
| scaffold_98 | 892 | 909 | + |
| scaffold_98 | 9152 | 9231 | + |
| scaffold_98 | 9266 | 9309 | + |
| scaffold_98 | 9544 | 11178 | + |
| scaffold_99 | 1278 | 5240 | + |
| scaffold_99 | 13405 | 13905 | + |
| scaffold_99 | 14679 | 14741 | + |
| scaffold_99 | 18291 | 20724 | + |
| scaffold_99 | 20780 | 21424 | + |
| scaffold_99 | 21685 | 21854 | + |
| scaffold_99 | 23968 | 23972 | + |
| scaffold_99 | 24225 | 24402 | + |
| scaffold_99 | 24527 | 24715 | + |
| scaffold_99 | 24773 | 24989 | + |
| scaffold_99 | 26406 | 26496 | + |
| scaffold_99 | 26946 | 27631 | + |
| scaffold_99 | 27755 | 28366 | + |
| scaffold_99 | 5339 | 5355 | + |
| scaffold_99 | 59 | 578 | + |
| scaffold_99 | 6346 | 6349 | + |
| scaffold_99 | 699 | 1136 | + |
| scaffold_99 | 8320 | 8353 | + |
| scaffold_99 | 8547 | 12746 | + |
