# Supplementary material for: Global Genome and Transcriptome Analyses of Magnaporthe oryzae Epidemic Isolate 98-06 Uncover Novel Effectors and Pathogenicity-Related Genes, Revealing Gene Gain and Lose Dynamics in Genome Evolution
Source: PLoS Pathog. 2015 Apr 2;11(4):e1004801. doi: 10.1371/journal.ppat.1004801 (PMC4383609; doi:10.1371/journal.ppat.1004801)
Supplement: S3 Table — (DOC) [file ppat.1004801.s018.doc]

**Table S3** **Isolate-unique genes in 98-06 compared to 70-15**.

| **Gene ID** | **SP^a^** | **P131^b^** | **Y34^c^** | **Annotation** |
| --- | --- | --- | --- | --- |
| Mo_GLEAN_10000026 | N | N | Y | predicted protein |
| Mo_GLEAN_10000033 | N | Y | Y | hypothetical protein |
| Mo_GLEAN_10000047 | N | N | Y | polyketide synthase |
| Mo_GLEAN_10000064 | N | N | N | hypothetical protein |
| Mo_GLEAN_10000092 | N | Y | N | hypothetical protein |
| Mo_GLEAN_10000096 | N | Y | N | mitochondrial chaperone BCS1 |
| Mo_GLEAN_10000098 | N | Y | Y | hypothetical protein |
| Mo_GLEAN_10000119 | N | N | Y | hypothetical protein |
| Mo_GLEAN_10000124 | N | N | Y | predicted protein |
| Mo_GLEAN_10000126 | N | Y | Y | hypothetical protein |
| Mo_GLEAN_10000129 | N | Y | Y | hypothetical protein |
| Mo_GLEAN_10000133 | N | Y | Y | hypothetical protein |
| Mo_GLEAN_10000165 | N | Y | N | hypothetical protein |
| Mo_GLEAN_10000182 | Y | Y | N | hypothetical protein |
| Mo_GLEAN_10000201 | N | Y | Y | hypothetical protein |
| Mo_GLEAN_10000202 | N | Y | Y | hypothetical protein |
| Mo_GLEAN_10000237 | N | N | Y | hypothetical protein |
| Mo_GLEAN_10000262 | N | Y | N | NA |
| Mo_GLEAN_10000337 | N | Y | Y | IBR finger domain-containing protein |
| Mo_GLEAN_10000339 | N | Y | Y | glycosyl hydrolase family 3 N terminal domain-containing protein |
| Mo_GLEAN_10000344 | N | Y | Y | hypothetical protein |
| Mo_GLEAN_10000349 | N | N | Y | hypothetical protein |
| Mo_GLEAN_10000356 | Y | N | N | lipase 2 |
| Mo_GLEAN_10000357 | N | Y | N | short chain dehydrogenase |
| Mo_GLEAN_10000360 | N | N | N | short chain dehydrogenase |
| Mo_GLEAN_10000374 | Y | Y | Y | hypothetical protein |
| Mo_GLEAN_10000381 | N | N | Y | hypothetical protein |
| Mo_GLEAN_10000382 | N | N | N | hypothetical protein |
| Mo_GLEAN_10000383 | N | Y | N | hypothetical protein |
| Mo_GLEAN_10000384 | N | Y | Y | hypothetical protein |
| Mo_GLEAN_10000386 | N | N | N | NA |
| Mo_GLEAN_10000388 | N | Y | Y | E3 ubiquitin ligase complex SCF subunit |
| Mo_GLEAN_10000389 | N | N | Y | hypothetical protein |
| Mo_GLEAN_10000396 | N | Y | Y | hypothetical protein |
| Mo_GLEAN_10000399 | N | Y | Y | hypothetical protein |
| Mo_GLEAN_10000403 | N | N | N | hypothetical protein |
| Mo_GLEAN_10000404 | N | Y | Y | hypothetical protein |
| Mo_GLEAN_10000412 | N | Y | Y | hypothetical protein |
| Mo_GLEAN_10000421 | N | Y | Y | hypothetical protein |
| Mo_GLEAN_10000424 | N | N | Y | hypothetical protein |
| Mo_GLEAN_10000439 | N | N | N | hypothetical protein |
| Mo_GLEAN_10000441 | N | Y | Y | NA |
| Mo_GLEAN_10000442 | N | Y | Y | NA |
| Mo_GLEAN_10000469 | N | N | Y | hypothetical protein |
| Mo_GLEAN_10000473 | N | N | N | hypothetical protein |
| Mo_GLEAN_10000486 | N | Y | Y | cell surface glycoprotein |
| Mo_GLEAN_10000497 | N | Y | Y | hypothetical protein |
| Mo_GLEAN_10000506 | N | Y | Y | hypothetical protein |
| Mo_GLEAN_10000535 | N | N | N | hypothetical protein |
| Mo_GLEAN_10000553 | N | Y | Y | unknown protein |
| Mo_GLEAN_10000602 | N | Y | Y | hypothetical protein |
| Mo_GLEAN_10000618 | Y | Y | Y | secretory lipase |
| Mo_GLEAN_10000637 | N | Y | Y | hypothetical protein |
| Mo_GLEAN_10000638 | N | Y | Y | hypothetical protein |
| Mo_GLEAN_10000645 | N | Y | Y | hypothetical protein |
| Mo_GLEAN_10000646 | N | Y | Y | NA |
| Mo_GLEAN_10000648 | N | Y | Y | hypothetical protein |
| Mo_GLEAN_10000650 | N | Y | N | hypothetical protein |
| Mo_GLEAN_10000654 | N | Y | Y | hypothetical protein |
| Mo_GLEAN_10000657 | N | Y | N | hypothetical protein |
| Mo_GLEAN_10000690 | N | Y | Y | hypothetical protein |
| Mo_GLEAN_10000733 | N | N | N | NA |
| Mo_GLEAN_10000773 | N | Y | Y | NA |
| Mo_GLEAN_10000774 | N | N | Y | hypothetical protein |
| Mo_GLEAN_10000778 | N | Y | N | hypothetical protein |
| Mo_GLEAN_10000780 | N | N | N | hypothetical protein |
| Mo_GLEAN_10000815 | Y | N | N | hypothetical protein |
| Mo_GLEAN_10000825 | N | Y | N | hypothetical protein |
| Mo_GLEAN_10000876 | N | N | Y | hypothetical protein |
| Mo_GLEAN_10000877 | N | N | Y | geranylgeranyl pyrophosphate synthetase |
| Mo_GLEAN_10000878 | N | Y | Y | interferon-induced GTP-binding protein |
| Mo_GLEAN_10000882 | N | Y | Y | hypothetical protein |
| Mo_GLEAN_10000883 | N | N | Y | HD family protein |
| Mo_GLEAN_10000884 | N | Y | Y | hypothetical protein |
| Mo_GLEAN_10000887 | N | Y | Y | hypothetical protein |
| Mo_GLEAN_10000891 | N | Y | Y | hypothetical protein |
| Mo_GLEAN_10001043 | N | Y | Y | hypothetical protein |
| Mo_GLEAN_10001047 | N | Y | Y | hypothetical protein |
| Mo_GLEAN_10001052 | N | Y | Y | hypothetical protein |
| Mo_GLEAN_10001128 | Y | Y | Y | AVR-Pik |
| Mo_GLEAN_10001129 | N | Y | Y | putative transposase |
| Mo_GLEAN_10001237 | N | Y | Y | hypothetical protein |
| Mo_GLEAN_10001242 | N | N | N | hypothetical protein L |
| Mo_GLEAN_10001302 | N | Y | Y | predicted protein |
| Mo_GLEAN_10001312 | N | Y | N | hypothetical protein |
| Mo_GLEAN_10001314 | Y | Y | Y | hypothetical protein |
| Mo_GLEAN_10001317 | N | Y | Y | cytochrome P450 monooxygenase |
| Mo_GLEAN_10001318 | N | Y | Y | hypothetical protein |
| Mo_GLEAN_10001358 | N | Y | Y | hypothetical protein |
| Mo_GLEAN_10001402 | N | N | N | hypothetical protein |
| Mo_GLEAN_10001403 | N | Y | Y | hypothetical protein |
| Mo_GLEAN_10001407 | N | N | Y | hypothetical protein |
| Mo_GLEAN_10001411 | N | N | Y | hypothetical protein |
| Mo_GLEAN_10001414 | N | Y | Y | hypothetical protein |
| Mo_GLEAN_10001415 | N | N | Y | hypothetical protein |
| Mo_GLEAN_10001416 | N | Y | Y | hypothetical protein |
| Mo_GLEAN_10001418 | N | Y | Y | hydrolase |
| Mo_GLEAN_10001420 | N | Y | Y | hypothetical protein |
| Mo_GLEAN_10001424 | N | Y | Y | hypothetical protein |
| Mo_GLEAN_10001426 | N | Y | Y | hypothetical protein |
| Mo_GLEAN_10001428 | N | N | N | predicted protein |
| Mo_GLEAN_10001429 | N | Y | Y | initiation-specific alpha-1,6-mannosyltransferase |
| Mo_GLEAN_10001445 | N | N | Y | polyketide synthase, putative |
| Mo_GLEAN_10001446 | N | N | Y | Geranylgeranyl pyrophosphate synthase |
| Mo_GLEAN_10001448 | N | N | Y | hypothetical protein |
| Mo_GLEAN_10001449 | N | Y | Y | hypothetical protein |
| Mo_GLEAN_10001450 | N | Y | Y | terpene synthase family protein |
| Mo_GLEAN_10001451 | N | Y | Y | hypothetical protein |
| Mo_GLEAN_10001590 | N | Y | Y | hypothetical protein |
| Mo_GLEAN_10001694 | N | N | N | hypothetical protein |
| Mo_GLEAN_10001733 | Y | N | Y | NA |
| Mo_GLEAN_10001972 | N | Y | Y | glycoside hydrolase family 61 protein |
| Mo_GLEAN_10001973 | Y | Y | Y | NA |
| Mo_GLEAN_10002006 | N | Y | Y | hypothetical protein |
| Mo_GLEAN_10002050 | N | Y | Y | hypothetical protein |
| Mo_GLEAN_10002051 | N | Y | Y | hypothetical protein |
| Mo_GLEAN_10002054 | N | Y | Y | hypothetical protein |
| Mo_GLEAN_10002059 | N | Y | Y | hypothetical protein |
| Mo_GLEAN_10002094 | N | N | N | Pc20g08910 |
| Mo_GLEAN_10002276 | Y | Y | Y | hypothetical protein |
| Mo_GLEAN_10002277 | N | Y | Y | hypothetical protein |
| Mo_GLEAN_10002278 | N | Y | Y | hypothetical protein |
| Mo_GLEAN_10002374 | N | Y | Y | hypothetical protein |
| Mo_GLEAN_10002376 | N | Y | Y | hypothetical protein |
| Mo_GLEAN_10002378 | N | Y | Y | NA |
| Mo_GLEAN_10002439 | N | N | N | diphosphomevalonate decarboxylase |
| Mo_GLEAN_10002584 | N | Y | Y | hypothetical protein |
| Mo_GLEAN_10002661 | N | Y | Y | hypothetical protein |
| Mo_GLEAN_10002662 | N | Y | Y | hypothetical protein |
| Mo_GLEAN_10002924 | N | N | N | hypothetical protein |
| Mo_GLEAN_10002950 | Y | Y | Y | hypothetical protein |
| Mo_GLEAN_10002992 | N | Y | Y | cell surface glycoprotein |
| Mo_GLEAN_10003359 | N | N | N | hypothetical protein |
| Mo_GLEAN_10003456 | N | Y | Y | kinesin light chain, variant |
| Mo_GLEAN_10003714 | N | Y | N | NA |
| Mo_GLEAN_10003768 | N | Y | Y | hypothetical protein |
| Mo_GLEAN_10003769 | N | Y | Y | ent-kaurene synthase |
| Mo_GLEAN_10004417 | N | Y | Y | hypothetical protein |
| Mo_GLEAN_10005013 | N | N | N | predicted protein |
| Mo_GLEAN_10005156 | N | N | N | hypothetical protein |
| Mo_GLEAN_10005578 | N | Y | Y | chitinase |
| Mo_GLEAN_10005580 | N | Y | Y | cell surface glycoprotein |
| Mo_GLEAN_10006017 | N | Y | Y | cell surface glycoprotein |
| Mo_GLEAN_10006029 | N | Y | Y | cytochrome P450 |
| Mo_GLEAN_10006082 | N | Y | Y | class III chitinase, variant |
| Mo_GLEAN_10006140 | Y | Y | Y | Lys |
| Mo_GLEAN_10006518 | Y | Y | Y | hypothetical protein |
| Mo_GLEAN_10006519 | N | Y | Y | hypothetical protein |
| Mo_GLEAN_10007207 | N | N | N | pol polyprotein |
| Mo_GLEAN_10007359 | N | N | N | hypothetical protein |
| Mo_GLEAN_10007627 | N | N | N | hypothetical protein |
| Mo_GLEAN_10008490 | N | Y | Y | hypothetical protein |
| Mo_GLEAN_10008497 | N | Y | Y | hypothetical protein |
| Mo_GLEAN_10010144 | N | Y | Y | hypothetical protein |
| Mo_GLEAN_10010240 | N | N | N | NA |
| Mo_GLEAN_10010482 | N | Y | Y | calcium-translocating P-type ATPase |
| Mo_GLEAN_10010492 | Y | Y | Y | hypothetical protein |
| Mo_GLEAN_10010501 | N | N | N | NA |
| Mo_GLEAN_10011166 | N | Y | Y | hypothetical protein |
| Mo_GLEAN_10011224 | N | N | N | hypothetical protein |
| Mo_GLEAN_10011891 | N | N | N | hypothetical protein |
| Mo_GLEAN_10012807 | N | N | N | short chain dehydrogenase |
| Mo_GLEAN_10013905 | N | N | N | glycosyltransferase PglE |
| Mo_GLEAN_10000028 | N | Y | Y | hypothetical protein |
| Mo_GLEAN_10000036 | N | Y | N | hypothetical protein |
| Mo_GLEAN_10000050 | N | Y | N | hypothetical protein |
| Mo_GLEAN_10000056 | N | Y | Y | hypothetical protein |
| Mo_GLEAN_10000090 | N | Y | N | hypothetical protein |
| Mo_GLEAN_10000159 | N | Y | Y | hypothetical protein |
| Mo_GLEAN_10000162 | N | Y | Y | phosphorylase superfamily protein |
| Mo_GLEAN_10000261 | N | Y | Y | NA |
| Mo_GLEAN_10000323 | N | Y | Y | NA |
| Mo_GLEAN_10000358 | N | Y | N | hypothetical protein GLRG_11804 |
| Mo_GLEAN_10000359 | N | N | N | hypothetical protein CH063_01135 |
| Mo_GLEAN_10000380 | N | N | Y | NA |
| Mo_GLEAN_10000385 | N | Y | Y | NA |
| Mo_GLEAN_10000410 | N | Y | Y | NA |
| Mo_GLEAN_10000438 | N | Y | Y | phosphorylase superfamily protein |
| Mo_GLEAN_10000440 | N | Y | Y | NA |
| Mo_GLEAN_10000505 | N | Y | Y | hypothetical protein |
| Mo_GLEAN_10000617 | Y | N | N | NA |
| Mo_GLEAN_10000632 | N | N | N | predicted protein |
| Mo_GLEAN_10000647 | N | N | Y | hypothetical protein |
| Mo_GLEAN_10000674 | N | N | N | hypothetical protein |
| Mo_GLEAN_10000675 | N | N | N | Pc22g21300 |
| Mo_GLEAN_10000765 | Y | N | N | hypothetical protein |
| Mo_GLEAN_10000779 | N | Y | Y | hypothetical protein |
| Mo_GLEAN_10000827 | N | Y | N | NA |
| Mo_GLEAN_10000872 | N | Y | N | hypothetical protein |
| Mo_GLEAN_10000873 | N | Y | Y | hypothetical protein |
| Mo_GLEAN_10000886 | N | Y | Y | YALI0B16566p |
| Mo_GLEAN_10000960 | Y | N | N | hypothetical protein |
| Mo_GLEAN_10000989 | N | Y | Y | hypothetical protein |
| Mo_GLEAN_10000990 | N | Y | Y | NA |
| Mo_GLEAN_10000991 | N | Y | Y | HET domain-containing protein, partial |
| Mo_GLEAN_10000992 | Y | Y | Y | NA |
| Mo_GLEAN_10001315 | N | Y | Y | BcABA3 |
| Mo_GLEAN_10001316 | N | Y | Y | pisatin demethylase |
| Mo_GLEAN_10001384 | N | Y | Y | AVR-Pita1 |
| Mo_GLEAN_10001404 | N | N | N | hypothetical protein |
| Mo_GLEAN_10001410 | N | N | Y | hypothetical protein |
| Mo_GLEAN_10001413 | N | Y | Y | serine/threonine-protein kinase |
| Mo_GLEAN_10001417 | N | Y | N | hypothetical protein |
| Mo_GLEAN_10001421 | N | Y | Y | NA |
| Mo_GLEAN_10001422 | N | Y | N | hypothetical protein |
| Mo_GLEAN_10001427 | N | Y | Y | hypothetical protein |
| Mo_GLEAN_10001447 | N | N | Y | transcription factor |
| Mo_GLEAN_10001452 | N | Y | Y | hypothetical protein |
| Mo_GLEAN_10001456 | N | Y | N | predicted protein |
| Mo_GLEAN_10001766 | N | Y | Y | major facilitator superfamily transporter |
| Mo_GLEAN_10001767 | N | Y | Y | hypothetical protein |
| Mo_GLEAN_10001768 | N | Y | Y | hypothetical protein |
| Mo_GLEAN_10002057 | N | N | N | hypothetical protein |
| Mo_GLEAN_10002375 | N | Y | Y | hypothetical protein |
| Mo_GLEAN_10002377 | N | Y | Y | C6 transcription factor |
| Mo_GLEAN_10002660 | N | Y | Y | predicted protein |
| Mo_GLEAN_10002663 | N | Y | Y | hypothetical protein |
| Mo_GLEAN_10002850 | N | Y | N | NA |
| Mo_GLEAN_10002939 | N | N | Y | hypothetical protein |
| Mo_GLEAN_10002940 | N | N | Y | hypothetical protein |
| Mo_GLEAN_10002941 | N | N | Y | NA |
| Mo_GLEAN_10003442 | N | Y | Y | NA |
| Mo_GLEAN_10003589 | Y | Y | Y | NA |
| Mo_GLEAN_10004558 | Y | Y | N | NA |
| Mo_GLEAN_10004595 | N | Y | N | NA |
| Mo_GLEAN_10005055 | Y | Y | Y | hypothetical protein |
| Mo_GLEAN_10005322 | N | N | N | NA |
| Mo_GLEAN_10005403 | N | Y | Y | hypothetical protein |
| Mo_GLEAN_10006081 | N | Y | Y | hypothetical protein |
| Mo_GLEAN_10007314 | Y | N | Y | hypothetical protein |
| Mo_GLEAN_10010345 | N | Y | Y | hypothetical protein |
| Mo_GLEAN_10010483 | N | Y | Y | hypothetical protein |
| Mo_GLEAN_10010484 | Y | Y | Y | NA |
| Mo_GLEAN_10010485 | Y | Y | Y | hypothetical protein |
| Mo_GLEAN_10010486 | Y | Y | Y | hypothetical protein |
| Mo_GLEAN_10011832 | Y | Y | Y | hypothetical protein |
| Mo_GLEAN_10012805 | N | N | N | hypothetical protein |
| Mo_GLEAN_10012806 | N | Y | N | hypothetical protein |

**a:** signal peptide prediction. “Y” indicates secreted, “N” indicates unsecreted.

**b, c:** 98-06 isolate-unique genes were blasted against genome of P131 or Y34. “Y” indicates containing in P131 or Y34; “N” indicates not containing in P131 or Y34.
