# Supplementary material for: Global Genome and Transcriptome Analyses of Magnaporthe oryzae Epidemic Isolate 98-06 Uncover Novel Effectors and Pathogenicity-Related Genes, Revealing Gene Gain and Lose Dynamics in Genome Evolution
Source: PLoS Pathog. 2015 Apr 2;11(4):e1004801. doi: 10.1371/journal.ppat.1004801 (PMC4383609; doi:10.1371/journal.ppat.1004801)
Supplement: S5 Table — (DOC) [file ppat.1004801.s020.doc]

**Table S5. T****ransposable elements identified in isolate 98-06, P131, Y34, and 70-15**.

| **Transposons** | **98-06** | **P131** | **Y34** | **70-15** |
| --- | --- | --- | --- | --- |
| **DNA** | 1,141 | 485 | 583 | 422 |
| **LINE** | 338 | 127 | 141 | 187 |
| **LTR** | 1,297 | 846 | 961 | 1,148 |
| **SINE** | 0 | 179 | 195 | 176 |
| **Other** | 0 | 418 | 442 | 364 |
| **Total** | 2,776 | 2,055 | 2,322 | 2,297 |

LTR, long terminal repeat; LINE, long interspersed repeat element; SINE, short interspersed repeat element.
