# Supplementary material for: Global Genome and Transcriptome Analyses of Magnaporthe oryzae Epidemic Isolate 98-06 Uncover Novel Effectors and Pathogenicity-Related Genes, Revealing Gene Gain and Lose Dynamics in Genome Evolution
Source: PLoS Pathog. 2015 Apr 2;11(4):e1004801. doi: 10.1371/journal.ppat.1004801 (PMC4383609; doi:10.1371/journal.ppat.1004801)
Supplement: S6 Table — (DOC) [file ppat.1004801.s021.doc]

**Table S6** **Secreted protein genes of 98-06 disrupted by TE.**

| **Scaffold** | **TE** | **Gene ID** | **TE-start** | **TE-end** | **Gene-start** | **Gene-end** |
| --- | --- | --- | --- | --- | --- | --- |
| scaffold_16 | LINE/R1 | Mo_GLEAN_10004846 | 132010 | 132156 | 131893 | 133086 |
| scaffold_16 | LTR/Gypsy | Mo_GLEAN_10004910 | 355470 | 355543 | 354302 | 357009 |
| scaffold_16 | DNA/Maverick | Mo_GLEAN_10004935 | 424893 | 424995 | 424320 | 425015 |
| scaffold_17 | LINE/Jockey | Mo_GLEAN_10004751 | 492990 | 493115 | 492826 | 495092 |
| scaffold_18 | LTR/Gypsy | Mo_GLEAN_10004503 | 241981 | 242043 | 241572 | 243070 |
| scaffold_19 | DNA/Academ | Mo_GLEAN_10004283 | 154731 | 154786 | 154224 | 155793 |
| scaffold_21 | LTR/Gypsy | Mo_GLEAN_10004023 | 387834 | 387901 | 387495 | 388070 |
| scaffold_22 | LINE/R1 | Mo_GLEAN_10003761 | 16874 | 16978 | 16807 | 17553 |
| scaffold_23 | LTR/DIRS | Mo_GLEAN_10003664 | 174358 | 174369 | 173095 | 174445 |
| scaffold_23 | LINE/R1 | Mo_GLEAN_10003706 | 281297 | 281412 | 281207 | 282388 |
| scaffold_23 | DNA/TcMar-m44 | Mo_GLEAN_10003745 | 397570 | 397703 | 397598 | 398773 |
| scaffold_24 | LINE/Tad1 | Mo_GLEAN_10003496 | 27822 | 28055 | 25957 | 27825 |
| scaffold_24 | DNA/TcMar | Mo_GLEAN_10003543 | 166370 | 166551 | 165791 | 167026 |
| scaffold_24 | LTR/Ngaro | Mo_GLEAN_10003581 | 276124 | 276223 | 275801 | 276586 |
| scaffold_25 | DNA/Sola | Mo_GLEAN_10003367 | 22367 | 22448 | 20160 | 22574 |
| scaffold_25 | LINE/RTE-X | Mo_GLEAN_10003465 | 344860 | 344916 | 344911 | 347241 |
| scaffold_26 | LINE/R1 | Mo_GLEAN_10003273 | 173597 | 173783 | 173046 | 174047 |
| scaffold_26 | LTR/Gypsy | Mo_GLEAN_10003328 | 299305 | 299350 | 298286 | 299631 |
| scaffold_27 | DNA/MULE-MuDR | Mo_GLEAN_10003102 | 28809 | 28885 | 28756 | 28884 |
| scaffold_27 | LINE/Tad1 | Mo_GLEAN_10003138 | 132076 | 132309 | 131149 | 133137 |
| scaffold_27 | LINE/Jockey | Mo_GLEAN_10003158 | 185447 | 185544 | 184067 | 185662 |
| scaffold_30 | LINE/CR1 | Mo_GLEAN_10002746 | 2219 | 2369 | 1897 | 3084 |
| scaffold_30 | LTR/Copia | Mo_GLEAN_10002748 | 10944 | 11020 | 10978 | 12615 |
| scaffold_30 | LINE/R1 | Mo_GLEAN_10002779 | 97035 | 97346 | 96548 | 97849 |
| scaffold_30 | DNA/TcMar-Tc1 | Mo_GLEAN_10002806 | 178609 | 178689 | 177976 | 178826 |
| scaffold_31 | LTR/Copia | Mo_GLEAN_10002743 | 280915 | 280929 | 279835 | 280940 |
| scaffold_32 | LINE/R1 | Mo_GLEAN_10002608 | 36587 | 36728 | 35766 | 36872 |
| scaffold_34 | DNA/Sola | Mo_GLEAN_10002436 | 63815 | 63939 | 63517 | 64056 |
| scaffold_34 | DNA/TcMar-Sagan | Mo_GLEAN_10002474 | 170503 | 170567 | 169637 | 172228 |
| scaffold_36 | DNA/PIF-Harbinger | Mo_GLEAN_10002322 | 123190 | 123205 | 122979 | 123950 |
| scaffold_38 | LTR/Gypsy | Mo_GLEAN_10002170 | 110566 | 110603 | 110157 | 110843 |
| scaffold_40 | DNA/TcMar-Sagan | Mo_GLEAN_10002016 | 75645 | 75732 | 75442 | 76515 |
| scaffold_40 | LINE/Tad1 | Mo_GLEAN_10002055 | 172301 | 172524 | 172474 | 173289 |
| scaffold_41 | LINE/R1 | Mo_GLEAN_10001934 | 53388 | 53523 | 52815 | 54679 |
| scaffold_43 | LTR/Gypsy | Mo_GLEAN_10001798 | 23495 | 23545 | 21133 | 25052 |
| scaffold_48 | LTR/Gypsy | Mo_GLEAN_10001521 | 69 | 200 | 4 | 510 |
| scaffold_49 | LTR/Gypsy | Mo_GLEAN_10001472 | 2396 | 2815 | 2769 | 3008 |
| scaffold_49 | LINE/R1 | Mo_GLEAN_10001486 | 55030 | 55101 | 49548 | 56696 |
| scaffold_49 | LTR/Gypsy | Mo_GLEAN_10001518 | 143481 | 143673 | 142621 | 144329 |
| scaffold_57 | LINE/Tad1 | Mo_GLEAN_10001176 | 96067 | 96300 | 94918 | 97450 |
| scaffold_58 | LTR/Gypsy | Mo_GLEAN_10001093 | 13044 | 13105 | 12405 | 14720 |
| scaffold_61 | DNA/CMC-EnSpm | Mo_GLEAN_10000992 | 16605 | 16647 | 16423 | 16692 |
| scaffold_63 | LTR/Copia | Mo_GLEAN_10000951 | 64669 | 64721 | 64651 | 67124 |
| scaffold_64 | LTR/Gypsy | Mo_GLEAN_10000897 | 236 | 690 | 300 | 788 |
| scaffold_64 | LTR/Gypsy | Mo_GLEAN_10000914 | 51774 | 51797 | 51328 | 52055 |
| scaffold_69 | DNA/DNA | Mo_GLEAN_10000794 | 23953 | 24084 | 23858 | 24727 |
| scaffold_73 | LTR/Gypsy | Mo_GLEAN_10000709 | 13849 | 13922 | 13615 | 14160 |
| scaffold_73 | LTR/Gypsy | Mo_GLEAN_10000712 | 25839 | 25887 | 24025 | 26015 |
| scaffold_73 | LINE/R1 | Mo_GLEAN_10000718 | 40295 | 40381 | 39756 | 41807 |
| scaffold_73 | LINE/Jockey | Mo_GLEAN_10000719 | 46070 | 46218 | 45797 | 46669 |
| scaffold_86 | DNA/CMC-EnSpm | Mo_GLEAN_10000518 | 7178 | 7262 | 6551 | 7198 |
| scaffold_115 | LTR/Gypsy | Mo_GLEAN_10000313 | 15573 | 15619 | 14808 | 15582 |
| scaffold_136 | LINE/Tad1 | Mo_GLEAN_10000210 | 2665 | 3161 | 2274 | 2746 |
| scaffold_141 | LINE/I | Mo_GLEAN_10000188 | 2983 | 3021 | 2518 | 3373 |
| scaffold_198 | LTR/Gypsy | Mo_GLEAN_10000049 | 2565 | 2620 | 1810 | 3270 |
| scaffold_1 | LINE/RTE-X | Mo_GLEAN_10012868 | 49299 | 49493 | 49180 | 50094 |
| scaffold_1 | LINE/RTE-X | Mo_GLEAN_10012873 | 65577 | 65664 | 64697 | 65881 |
| scaffold_1 | LTR/Gypsy | Mo_GLEAN_10013072 | 707644 | 707827 | 707199 | 707833 |
| scaffold_1 | LINE/R1 | Mo_GLEAN_10013891 | 3094497 | 3094574 | 3094395 | 3095367 |
| scaffold_1 | LINE/Tad1 | Mo_GLEAN_10014012 | 3461101 | 3461523 | 3460707 | 3461126 |
| scaffold_2 | LINE/R1 | Mo_GLEAN_10012018 | 327571 | 327611 | 327161 | 327790 |
| scaffold_2 | LTR/Gypsy | Mo_GLEAN_10012022 | 343814 | 343872 | 342961 | 343878 |
| scaffold_2 | LINE/R1 | Mo_GLEAN_10012050 | 410901 | 410961 | 410646 | 411134 |
| scaffold_2 | LTR/Copia | Mo_GLEAN_10012315 | 1208330 | 1208387 | 1207402 | 1208688 |
| scaffold_2 | LTR/Gypsy | Mo_GLEAN_10012375 | 1372088 | 1372201 | 1371367 | 1372574 |
| scaffold_2 | DNA/CMC-EnSpm | Mo_GLEAN_10012502 | 1757666 | 1757751 | 1756489 | 1758474 |
| scaffold_3 | LTR/Gypsy | Mo_GLEAN_10011220 | 180424 | 180467 | 180125 | 180727 |
| scaffold_3 | LTR/Pao | Mo_GLEAN_10011281 | 431836 | 431875 | 430866 | 432107 |
| scaffold_3 | LINE/Jockey | Mo_GLEAN_10011452 | 915012 | 915272 | 912094 | 916534 |
| scaffold_4 | LTR/Pao | Mo_GLEAN_10010822 | 1253592 | 1253644 | 1253233 | 1254680 |
| scaffold_4 | DNA/CMC-Transib | Mo_GLEAN_10010862 | 1382188 | 1382280 | 1381631 | 1382980 |
| scaffold_4 | LTR/Gypsy | Mo_GLEAN_10010966 | 1686990 | 1687160 | 1686893 | 1687566 |
| scaffold_4 | LTR/Gypsy | Mo_GLEAN_10010989 | 1762277 | 1762342 | 1760421 | 1763148 |
| scaffold_4 | LTR/LTR | Mo_GLEAN_10010999 | 1791483 | 1791639 | 1791636 | 1791968 |
| scaffold_5 | LINE/R1 | Mo_GLEAN_10009739 | 96641 | 96760 | 96395 | 97571 |
| scaffold_5 | DNA/TcMar-Tc1 | Mo_GLEAN_10009812 | 340804 | 340882 | 340458 | 342698 |
| scaffold_5 | LTR/Pao | Mo_GLEAN_10010033 | 1081487 | 1081557 | 1081213 | 1081891 |
| scaffold_5 | DNA/hAT-Charlie | Mo_GLEAN_10010234 | 1643650 | 1643686 | 1642466 | 1644836 |
| scaffold_5 | LTR/Copia | Mo_GLEAN_10010284 | 1794462 | 1794514 | 1794157 | 1796164 |
| scaffold_5 | SINE/tRNA-Lys | Mo_GLEAN_10010364 | 2023972 | 2024064 | 2023554 | 2025372 |
| scaffold_6 | LTR/Gypsy | Mo_GLEAN_10009322 | 757844 | 757942 | 757830 | 758210 |
| scaffold_6 | LTR/Copia | Mo_GLEAN_10009337 | 796031 | 796083 | 795426 | 797033 |
| scaffold_6 | DNA/Maverick | Mo_GLEAN_10009359 | 861671 | 861751 | 861734 | 862936 |
| scaffold_6 | LTR/Gypsy | Mo_GLEAN_10009425 | 1106872 | 1106998 | 1106219 | 1107631 |
| scaffold_6 | DNA/Novosib | Mo_GLEAN_10009441 | 1153410 | 1153490 | 1152913 | 1154000 |
| scaffold_6 | LTR/Gypsy | Mo_GLEAN_10009493 | 1312352 | 1312461 | 1312247 | 1315054 |
| scaffold_6 | DNA/CMC-EnSpm | Mo_GLEAN_10009645 | 1749782 | 1749833 | 1747416 | 1750561 |
| scaffold_7 | LINE/R1 | Mo_GLEAN_10008850 | 1064463 | 1064572 | 1064173 | 1064518 |
| scaffold_7 | LINE/Jockey | Mo_GLEAN_10008964 | 1422168 | 1422373 | 1421823 | 1423536 |
| scaffold_8 | LTR/ERV1 | Mo_GLEAN_10008059 | 478996 | 479131 | 478602 | 481064 |
| scaffold_8 | LTR/ERVK | Mo_GLEAN_10008356 | 1399244 | 1399309 | 1398312 | 1399354 |
| scaffold_8 | LTR/Gypsy | Mo_GLEAN_10008421 | 1596923 | 1597028 | 1596008 | 1597136 |
| scaffold_8 | LINE/Ambal | Mo_GLEAN_10008463 | 1727706 | 1727791 | 1727475 | 1728218 |
| scaffold_8 | LTR/Pao | Mo_GLEAN_10008467 | 1746345 | 1746389 | 1745839 | 1747071 |
| scaffold_10 | DNA/Sola | Mo_GLEAN_10006895 | 30330 | 30386 | 29282 | 30803 |
| scaffold_10 | DNA/hAT | Mo_GLEAN_10006988 | 322072 | 322164 | 321948 | 322974 |
| scaffold_10 | DNA/TcMar | Mo_GLEAN_10007057 | 517745 | 517756 | 517536 | 518338 |
| scaffold_10 | LTR/Gypsy | Mo_GLEAN_10007110 | 685465 | 685538 | 684871 | 687104 |
| scaffold_10 | DNA/CMC-EnSpm | Mo_GLEAN_10007112 | 689654 | 689713 | 688790 | 689708 |
| scaffold_10 | LTR/Gypsy | Mo_GLEAN_10007216 | 1041484 | 1041549 | 1039797 | 1041952 |
| scaffold_10 | LINE/R1 | Mo_GLEAN_10007257 | 1186860 | 1186920 | 1186511 | 1187808 |
| scaffold_10 | LINE/R1 | Mo_GLEAN_10007294 | 1284976 | 1285229 | 1284899 | 1286086 |
| scaffold_9 | DNA/TcMar-m44 | Mo_GLEAN_10007634 | 814145 | 814241 | 813809 | 814678 |
| scaffold_9 | LTR/Gypsy | Mo_GLEAN_10007648 | 851321 | 851378 | 851235 | 851870 |
| scaffold_9 | LINE/Jockey | Mo_GLEAN_10007665 | 896611 | 896653 | 895842 | 896972 |
| scaffold_9 | LINE/Jockey | Mo_GLEAN_10007666 | 898570 | 898872 | 898458 | 899360 |
| scaffold_11 | DNA/CMC-EnSpm | Mo_GLEAN_10006383 | 41632 | 41708 | 41567 | 42104 |
| scaffold_11 | LINE/R1 | Mo_GLEAN_10006647 | 804646 | 804730 | 803830 | 805755 |
| scaffold_13 | LTR/Copia | Mo_GLEAN_10005758 | 386288 | 386341 | 386093 | 387764 |
| scaffold_13 | LTR/Gypsy | Mo_GLEAN_10005788 | 471791 | 472003 | 470993 | 472070 |
| scaffold_13 | LINE/Jockey | Mo_GLEAN_10005792 | 485052 | 485175 | 484435 | 485469 |
| scaffold_13 | DNA/CMC-EnSpm | Mo_GLEAN_10005851 | 638134 | 638181 | 637955 | 639240 |
| scaffold_13 | LINE/R1 | Mo_GLEAN_10005906 | 795616 | 795757 | 794599 | 796307 |
| scaffold_14 | DNA/TcMar | Mo_GLEAN_10005390 | 223904 | 224006 | 223205 | 224129 |
| scaffold_14 | DNA/Sola | Mo_GLEAN_10005419 | 368515 | 368634 | 366525 | 369062 |
| scaffold_14 | DNA/CMC-EnSpm | Mo_GLEAN_10005529 | 657458 | 657541 | 655102 | 658165 |
| scaffold_14 | LTR/Gypsy | Mo_GLEAN_10005544 | 697057 | 697806 | 696010 | 698466 |
| scaffold_14 | DNA/MULE-MuDR | Mo_GLEAN_10005548 | 711575 | 711773 | 710292 | 711893 |
| scaffold_14 | LTR/Copia | Mo_GLEAN_10005551 | 720165 | 720193 | 718744 | 720249 |
| scaffold_14 | LINE/R1 | Mo_GLEAN_10005561 | 749562 | 749647 | 748932 | 749709 |
| scaffold_15 | LTR/Gypsy | Mo_GLEAN_10005089 | 151104 | 151172 | 150661 | 151173 |
| scaffold_17 | DNA/TcMar-Fot1 | Mo_GLEAN_10004797 | 632482 | 632715 | 629435 | 633493 |
| scaffold_26 | LTR/Gypsy | Mo_GLEAN_10003345 | 343806 | 344006 | 342972 | 344147 |
| scaffold_28 | DNA/TcMar-Fot1 | Mo_GLEAN_10003050 | 263421 | 264095 | 261421 | 264421 |
| scaffold_49 | DNA/hAT | Mo_GLEAN_10001488 | 63333 | 63980 | 63012 | 64028 |
| scaffold_52 | DNA/TcMar-Fot1 | Mo_GLEAN_10001361 | 8948 | 9286 | 8948 | 9295 |
| scaffold_71 | LTR/Gypsy | Mo_GLEAN_10000755 | 11254 | 11349 | 10476 | 11700 |
| scaffold_1 | LTR/Gypsy | Mo_GLEAN_10013059 | 661599 | 662330 | 661070 | 661611 |
| scaffold_1 | DNA/P | Mo_GLEAN_10013420 | 1759077 | 1759235 | 1758672 | 1759983 |
| scaffold_10 | LTR/Gypsy | Mo_GLEAN_10007296 | 1296352 | 1297671 | 1288985 | 1296641 |
| scaffold_10 | DNA/TcMar-Ant1 | Mo_GLEAN_10007314 | 1360465 | 1360599 | 1360593 | 1360826 |
| scaffold_14 | DNA/TcMar-Fot1 | Mo_GLEAN_10005581 | 799579 | 801183 | 799289 | 801615 |
