# Supplementary material for: Global Genome and Transcriptome Analyses of Magnaporthe oryzae Epidemic Isolate 98-06 Uncover Novel Effectors and Pathogenicity-Related Genes, Revealing Gene Gain and Lose Dynamics in Genome Evolution
Source: PLoS Pathog. 2015 Apr 2;11(4):e1004801. doi: 10.1371/journal.ppat.1004801 (PMC4383609; doi:10.1371/journal.ppat.1004801)
Supplement: S7 Table — (DOC) [file ppat.1004801.s022.doc]

**Table S7** **645 small candidate effector proteins.**

| **Gene_ID** |
| --- |
| Mo_GLEAN_10000004 |
| Mo_GLEAN_10000021 |
| Mo_GLEAN_10000022 |
| Mo_GLEAN_10000034 |
| Mo_GLEAN_10000043 |
| Mo_GLEAN_10000045 |
| Mo_GLEAN_10000052 |
| Mo_GLEAN_10000053 |
| Mo_GLEAN_10000059 |
| Mo_GLEAN_10000106 |
| Mo_GLEAN_10000109 |
| Mo_GLEAN_10000148 |
| Mo_GLEAN_10000154 |
| Mo_GLEAN_10000182 |
| Mo_GLEAN_10000188 |
| Mo_GLEAN_10000191 |
| Mo_GLEAN_10000195 |
| Mo_GLEAN_10000210 |
| Mo_GLEAN_10000227 |
| Mo_GLEAN_10000247 |
| Mo_GLEAN_10000256 |
| Mo_GLEAN_10000276 |
| Mo_GLEAN_10000355 |
| Mo_GLEAN_10000374 |
| Mo_GLEAN_10000426 |
| Mo_GLEAN_10000501 |
| Mo_GLEAN_10000529 |
| Mo_GLEAN_10000533 |
| Mo_GLEAN_10000541 |
| Mo_GLEAN_10000543 |
| Mo_GLEAN_10000560 |
| Mo_GLEAN_10000561 |
| Mo_GLEAN_10000566 |
| Mo_GLEAN_10000613 |
| Mo_GLEAN_10000617 |
| Mo_GLEAN_10000664 |
| Mo_GLEAN_10000687 |
| Mo_GLEAN_10000709 |
| Mo_GLEAN_10000714 |
| Mo_GLEAN_10000730 |
| Mo_GLEAN_10000740 |
| Mo_GLEAN_10000751 |
| Mo_GLEAN_10000758 |
| Mo_GLEAN_10000765 |
| Mo_GLEAN_10000769 |
| Mo_GLEAN_10000786 |
| Mo_GLEAN_10000806 |
| Mo_GLEAN_10000831 |
| Mo_GLEAN_10000858 |
| Mo_GLEAN_10000897 |
| Mo_GLEAN_10000902 |
| Mo_GLEAN_10000908 |
| Mo_GLEAN_10000914 |
| Mo_GLEAN_10000918 |
| Mo_GLEAN_10000919 |
| Mo_GLEAN_10000926 |
| Mo_GLEAN_10000949 |
| Mo_GLEAN_10000952 |
| Mo_GLEAN_10000959 |
| Mo_GLEAN_10000960 |
| Mo_GLEAN_10000981 |
| Mo_GLEAN_10000987 |
| Mo_GLEAN_10000992 |
| Mo_GLEAN_10000996 |
| Mo_GLEAN_10001002 |
| Mo_GLEAN_10001004 |
| Mo_GLEAN_10001021 |
| Mo_GLEAN_10001030 |
| Mo_GLEAN_10001049 |
| Mo_GLEAN_10001058 |
| Mo_GLEAN_10001082 |
| Mo_GLEAN_10001097 |
| Mo_GLEAN_10001105 |
| Mo_GLEAN_10001116 |
| Mo_GLEAN_10001124 |
| Mo_GLEAN_10001126 |
| Mo_GLEAN_10001128 |
| Mo_GLEAN_10001131 |
| Mo_GLEAN_10001135 |
| Mo_GLEAN_10001143 |
| Mo_GLEAN_10001160 |
| Mo_GLEAN_10001172 |
| Mo_GLEAN_10001173 |
| Mo_GLEAN_10001174 |
| Mo_GLEAN_10001178 |
| Mo_GLEAN_10001194 |
| Mo_GLEAN_10001228 |
| Mo_GLEAN_10001231 |
| Mo_GLEAN_10001245 |
| Mo_GLEAN_10001247 |
| Mo_GLEAN_10001256 |
| Mo_GLEAN_10001260 |
| Mo_GLEAN_10001270 |
| Mo_GLEAN_10001274 |
| Mo_GLEAN_10001305 |
| Mo_GLEAN_10001314 |
| Mo_GLEAN_10001323 |
| Mo_GLEAN_10001327 |
| Mo_GLEAN_10001331 |
| Mo_GLEAN_10001352 |
| Mo_GLEAN_10001361 |
| Mo_GLEAN_10001378 |
| Mo_GLEAN_10001379 |
| Mo_GLEAN_10001463 |
| Mo_GLEAN_10001472 |
| Mo_GLEAN_10001475 |
| Mo_GLEAN_10001495 |
| Mo_GLEAN_10001496 |
| Mo_GLEAN_10001499 |
| Mo_GLEAN_10001503 |
| Mo_GLEAN_10001518 |
| Mo_GLEAN_10001521 |
| Mo_GLEAN_10001539 |
| Mo_GLEAN_10001541 |
| Mo_GLEAN_10001542 |
| Mo_GLEAN_10001543 |
| Mo_GLEAN_10001550 |
| Mo_GLEAN_10001570 |
| Mo_GLEAN_10001577 |
| Mo_GLEAN_10001611 |
| Mo_GLEAN_10001636 |
| Mo_GLEAN_10001638 |
| Mo_GLEAN_10001666 |
| Mo_GLEAN_10001675 |
| Mo_GLEAN_10001679 |
| Mo_GLEAN_10001680 |
| Mo_GLEAN_10001725 |
| Mo_GLEAN_10001733 |
| Mo_GLEAN_10001735 |
| Mo_GLEAN_10001736 |
| Mo_GLEAN_10001748 |
| Mo_GLEAN_10001804 |
| Mo_GLEAN_10001809 |
| Mo_GLEAN_10001825 |
| Mo_GLEAN_10001835 |
| Mo_GLEAN_10001840 |
| Mo_GLEAN_10001841 |
| Mo_GLEAN_10001882 |
| Mo_GLEAN_10001933 |
| Mo_GLEAN_10001939 |
| Mo_GLEAN_10001950 |
| Mo_GLEAN_10001970 |
| Mo_GLEAN_10001973 |
| Mo_GLEAN_10001994 |
| Mo_GLEAN_10002008 |
| Mo_GLEAN_10002106 |
| Mo_GLEAN_10002157 |
| Mo_GLEAN_10002203 |
| Mo_GLEAN_10002228 |
| Mo_GLEAN_10002267 |
| Mo_GLEAN_10002269 |
| Mo_GLEAN_10002272 |
| Mo_GLEAN_10002296 |
| Mo_GLEAN_10002316 |
| Mo_GLEAN_10002321 |
| Mo_GLEAN_10002343 |
| Mo_GLEAN_10002436 |
| Mo_GLEAN_10002464 |
| Mo_GLEAN_10002466 |
| Mo_GLEAN_10002481 |
| Mo_GLEAN_10002499 |
| Mo_GLEAN_10002512 |
| Mo_GLEAN_10002528 |
| Mo_GLEAN_10002544 |
| Mo_GLEAN_10002547 |
| Mo_GLEAN_10002566 |
| Mo_GLEAN_10002596 |
| Mo_GLEAN_10002597 |
| Mo_GLEAN_10002619 |
| Mo_GLEAN_10002716 |
| Mo_GLEAN_10002722 |
| Mo_GLEAN_10002723 |
| Mo_GLEAN_10002742 |
| Mo_GLEAN_10002773 |
| Mo_GLEAN_10002784 |
| Mo_GLEAN_10002826 |
| Mo_GLEAN_10002861 |
| Mo_GLEAN_10002876 |
| Mo_GLEAN_10002901 |
| Mo_GLEAN_10002904 |
| Mo_GLEAN_10002906 |
| Mo_GLEAN_10002925 |
| Mo_GLEAN_10002950 |
| Mo_GLEAN_10002989 |
| Mo_GLEAN_10002991 |
| Mo_GLEAN_10002993 |
| Mo_GLEAN_10003006 |
| Mo_GLEAN_10003021 |
| Mo_GLEAN_10003024 |
| Mo_GLEAN_10003044 |
| Mo_GLEAN_10003047 |
| Mo_GLEAN_10003049 |
| Mo_GLEAN_10003068 |
| Mo_GLEAN_10003072 |
| Mo_GLEAN_10003078 |
| Mo_GLEAN_10003079 |
| Mo_GLEAN_10003086 |
| Mo_GLEAN_10003088 |
| Mo_GLEAN_10003102 |
| Mo_GLEAN_10003110 |
| Mo_GLEAN_10003111 |
| Mo_GLEAN_10003137 |
| Mo_GLEAN_10003140 |
| Mo_GLEAN_10003146 |
| Mo_GLEAN_10003180 |
| Mo_GLEAN_10003187 |
| Mo_GLEAN_10003208 |
| Mo_GLEAN_10003209 |
| Mo_GLEAN_10003210 |
| Mo_GLEAN_10003216 |
| Mo_GLEAN_10003226 |
| Mo_GLEAN_10003229 |
| Mo_GLEAN_10003231 |
| Mo_GLEAN_10003240 |
| Mo_GLEAN_10003247 |
| Mo_GLEAN_10003253 |
| Mo_GLEAN_10003258 |
| Mo_GLEAN_10003292 |
| Mo_GLEAN_10003300 |
| Mo_GLEAN_10003306 |
| Mo_GLEAN_10003333 |
| Mo_GLEAN_10003342 |
| Mo_GLEAN_10003370 |
| Mo_GLEAN_10003377 |
| Mo_GLEAN_10003409 |
| Mo_GLEAN_10003413 |
| Mo_GLEAN_10003416 |
| Mo_GLEAN_10003417 |
| Mo_GLEAN_10003434 |
| Mo_GLEAN_10003436 |
| Mo_GLEAN_10003463 |
| Mo_GLEAN_10003469 |
| Mo_GLEAN_10003487 |
| Mo_GLEAN_10003489 |
| Mo_GLEAN_10003490 |
| Mo_GLEAN_10003505 |
| Mo_GLEAN_10003508 |
| Mo_GLEAN_10003513 |
| Mo_GLEAN_10003521 |
| Mo_GLEAN_10003554 |
| Mo_GLEAN_10003555 |
| Mo_GLEAN_10003561 |
| Mo_GLEAN_10003562 |
| Mo_GLEAN_10003569 |
| Mo_GLEAN_10003571 |
| Mo_GLEAN_10003572 |
| Mo_GLEAN_10003576 |
| Mo_GLEAN_10003641 |
| Mo_GLEAN_10003655 |
| Mo_GLEAN_10003669 |
| Mo_GLEAN_10003680 |
| Mo_GLEAN_10003752 |
| Mo_GLEAN_10003757 |
| Mo_GLEAN_10003761 |
| Mo_GLEAN_10003799 |
| Mo_GLEAN_10003805 |
| Mo_GLEAN_10003808 |
| Mo_GLEAN_10003830 |
| Mo_GLEAN_10003865 |
| Mo_GLEAN_10003896 |
| Mo_GLEAN_10003899 |
| Mo_GLEAN_10003900 |
| Mo_GLEAN_10003901 |
| Mo_GLEAN_10003935 |
| Mo_GLEAN_10003936 |
| Mo_GLEAN_10003937 |
| Mo_GLEAN_10003938 |
| Mo_GLEAN_10004021 |
| Mo_GLEAN_10004023 |
| Mo_GLEAN_10004027 |
| Mo_GLEAN_10004029 |
| Mo_GLEAN_10004030 |
| Mo_GLEAN_10004032 |
| Mo_GLEAN_10004050 |
| Mo_GLEAN_10004061 |
| Mo_GLEAN_10004065 |
| Mo_GLEAN_10004093 |
| Mo_GLEAN_10004096 |
| Mo_GLEAN_10004132 |
| Mo_GLEAN_10004134 |
| Mo_GLEAN_10004166 |
| Mo_GLEAN_10004226 |
| Mo_GLEAN_10004227 |
| Mo_GLEAN_10004319 |
| Mo_GLEAN_10004342 |
| Mo_GLEAN_10004370 |
| Mo_GLEAN_10004480 |
| Mo_GLEAN_10004481 |
| Mo_GLEAN_10004485 |
| Mo_GLEAN_10004486 |
| Mo_GLEAN_10004492 |
| Mo_GLEAN_10004507 |
| Mo_GLEAN_10004514 |
| Mo_GLEAN_10004518 |
| Mo_GLEAN_10004538 |
| Mo_GLEAN_10004541 |
| Mo_GLEAN_10004558 |
| Mo_GLEAN_10004567 |
| Mo_GLEAN_10004570 |
| Mo_GLEAN_10004583 |
| Mo_GLEAN_10004584 |
| Mo_GLEAN_10004742 |
| Mo_GLEAN_10004754 |
| Mo_GLEAN_10004765 |
| Mo_GLEAN_10004771 |
| Mo_GLEAN_10004811 |
| Mo_GLEAN_10004813 |
| Mo_GLEAN_10004814 |
| Mo_GLEAN_10004828 |
| Mo_GLEAN_10004908 |
| Mo_GLEAN_10004912 |
| Mo_GLEAN_10004913 |
| Mo_GLEAN_10004942 |
| Mo_GLEAN_10004953 |
| Mo_GLEAN_10005015 |
| Mo_GLEAN_10005019 |
| Mo_GLEAN_10005034 |
| Mo_GLEAN_10005055 |
| Mo_GLEAN_10005063 |
| Mo_GLEAN_10005083 |
| Mo_GLEAN_10005089 |
| Mo_GLEAN_10005093 |
| Mo_GLEAN_10005134 |
| Mo_GLEAN_10005147 |
| Mo_GLEAN_10005220 |
| Mo_GLEAN_10005222 |
| Mo_GLEAN_10005229 |
| Mo_GLEAN_10005253 |
| Mo_GLEAN_10005258 |
| Mo_GLEAN_10005262 |
| Mo_GLEAN_10005263 |
| Mo_GLEAN_10005276 |
| Mo_GLEAN_10005333 |
| Mo_GLEAN_10005351 |
| Mo_GLEAN_10005353 |
| Mo_GLEAN_10005360 |
| Mo_GLEAN_10005399 |
| Mo_GLEAN_10005406 |
| Mo_GLEAN_10005422 |
| Mo_GLEAN_10005434 |
| Mo_GLEAN_10005443 |
| Mo_GLEAN_10005493 |
| Mo_GLEAN_10005505 |
| Mo_GLEAN_10005522 |
| Mo_GLEAN_10005523 |
| Mo_GLEAN_10005524 |
| Mo_GLEAN_10005527 |
| Mo_GLEAN_10005532 |
| Mo_GLEAN_10005581 |
| Mo_GLEAN_10005654 |
| Mo_GLEAN_10005677 |
| Mo_GLEAN_10005689 |
| Mo_GLEAN_10005693 |
| Mo_GLEAN_10005694 |
| Mo_GLEAN_10005731 |
| Mo_GLEAN_10005785 |
| Mo_GLEAN_10005828 |
| Mo_GLEAN_10005907 |
| Mo_GLEAN_10005925 |
| Mo_GLEAN_10005931 |
| Mo_GLEAN_10005990 |
| Mo_GLEAN_10006003 |
| Mo_GLEAN_10006047 |
| Mo_GLEAN_10006053 |
| Mo_GLEAN_10006076 |
| Mo_GLEAN_10006115 |
| Mo_GLEAN_10006129 |
| Mo_GLEAN_10006137 |
| Mo_GLEAN_10006152 |
| Mo_GLEAN_10006247 |
| Mo_GLEAN_10006276 |
| Mo_GLEAN_10006298 |
| Mo_GLEAN_10006303 |
| Mo_GLEAN_10006317 |
| Mo_GLEAN_10006341 |
| Mo_GLEAN_10006383 |
| Mo_GLEAN_10006395 |
| Mo_GLEAN_10006396 |
| Mo_GLEAN_10006424 |
| Mo_GLEAN_10006504 |
| Mo_GLEAN_10006505 |
| Mo_GLEAN_10006610 |
| Mo_GLEAN_10006627 |
| Mo_GLEAN_10006641 |
| Mo_GLEAN_10006765 |
| Mo_GLEAN_10006800 |
| Mo_GLEAN_10006804 |
| Mo_GLEAN_10006812 |
| Mo_GLEAN_10006826 |
| Mo_GLEAN_10006838 |
| Mo_GLEAN_10006849 |
| Mo_GLEAN_10006925 |
| Mo_GLEAN_10006953 |
| Mo_GLEAN_10007044 |
| Mo_GLEAN_10007116 |
| Mo_GLEAN_10007154 |
| Mo_GLEAN_10007199 |
| Mo_GLEAN_10007214 |
| Mo_GLEAN_10007222 |
| Mo_GLEAN_10007263 |
| Mo_GLEAN_10007269 |
| Mo_GLEAN_10007296 |
| Mo_GLEAN_10007307 |
| Mo_GLEAN_10007314 |
| Mo_GLEAN_10007320 |
| Mo_GLEAN_10007322 |
| Mo_GLEAN_10007329 |
| Mo_GLEAN_10007331 |
| Mo_GLEAN_10007334 |
| Mo_GLEAN_10007351 |
| Mo_GLEAN_10007354 |
| Mo_GLEAN_10007360 |
| Mo_GLEAN_10007498 |
| Mo_GLEAN_10007501 |
| Mo_GLEAN_10007559 |
| Mo_GLEAN_10007593 |
| Mo_GLEAN_10007624 |
| Mo_GLEAN_10007626 |
| Mo_GLEAN_10007653 |
| Mo_GLEAN_10007655 |
| Mo_GLEAN_10007671 |
| Mo_GLEAN_10007675 |
| Mo_GLEAN_10007712 |
| Mo_GLEAN_10007714 |
| Mo_GLEAN_10007832 |
| Mo_GLEAN_10007862 |
| Mo_GLEAN_10007865 |
| Mo_GLEAN_10007874 |
| Mo_GLEAN_10007903 |
| Mo_GLEAN_10007906 |
| Mo_GLEAN_10007910 |
| Mo_GLEAN_10007919 |
| Mo_GLEAN_10007927 |
| Mo_GLEAN_10007942 |
| Mo_GLEAN_10007945 |
| Mo_GLEAN_10008044 |
| Mo_GLEAN_10008153 |
| Mo_GLEAN_10008167 |
| Mo_GLEAN_10008168 |
| Mo_GLEAN_10008191 |
| Mo_GLEAN_10008223 |
| Mo_GLEAN_10008274 |
| Mo_GLEAN_10008283 |
| Mo_GLEAN_10008301 |
| Mo_GLEAN_10008323 |
| Mo_GLEAN_10008356 |
| Mo_GLEAN_10008362 |
| Mo_GLEAN_10008365 |
| Mo_GLEAN_10008379 |
| Mo_GLEAN_10008391 |
| Mo_GLEAN_10008422 |
| Mo_GLEAN_10008423 |
| Mo_GLEAN_10008426 |
| Mo_GLEAN_10008447 |
| Mo_GLEAN_10008452 |
| Mo_GLEAN_10008454 |
| Mo_GLEAN_10008459 |
| Mo_GLEAN_10008483 |
| Mo_GLEAN_10008487 |
| Mo_GLEAN_10008494 |
| Mo_GLEAN_10008543 |
| Mo_GLEAN_10008560 |
| Mo_GLEAN_10008600 |
| Mo_GLEAN_10008667 |
| Mo_GLEAN_10008676 |
| Mo_GLEAN_10008726 |
| Mo_GLEAN_10008850 |
| Mo_GLEAN_10008899 |
| Mo_GLEAN_10008900 |
| Mo_GLEAN_10008990 |
| Mo_GLEAN_10009020 |
| Mo_GLEAN_10009021 |
| Mo_GLEAN_10009024 |
| Mo_GLEAN_10009030 |
| Mo_GLEAN_10009172 |
| Mo_GLEAN_10009322 |
| Mo_GLEAN_10009328 |
| Mo_GLEAN_10009335 |
| Mo_GLEAN_10009443 |
| Mo_GLEAN_10009507 |
| Mo_GLEAN_10009509 |
| Mo_GLEAN_10009573 |
| Mo_GLEAN_10009622 |
| Mo_GLEAN_10009648 |
| Mo_GLEAN_10009649 |
| Mo_GLEAN_10009664 |
| Mo_GLEAN_10009686 |
| Mo_GLEAN_10009706 |
| Mo_GLEAN_10009707 |
| Mo_GLEAN_10009727 |
| Mo_GLEAN_10009732 |
| Mo_GLEAN_10009734 |
| Mo_GLEAN_10009737 |
| Mo_GLEAN_10009738 |
| Mo_GLEAN_10009739 |
| Mo_GLEAN_10009741 |
| Mo_GLEAN_10009746 |
| Mo_GLEAN_10009781 |
| Mo_GLEAN_10009795 |
| Mo_GLEAN_10009845 |
| Mo_GLEAN_10009860 |
| Mo_GLEAN_10009874 |
| Mo_GLEAN_10009939 |
| Mo_GLEAN_10010033 |
| Mo_GLEAN_10010098 |
| Mo_GLEAN_10010103 |
| Mo_GLEAN_10010157 |
| Mo_GLEAN_10010160 |
| Mo_GLEAN_10010210 |
| Mo_GLEAN_10010217 |
| Mo_GLEAN_10010286 |
| Mo_GLEAN_10010312 |
| Mo_GLEAN_10010350 |
| Mo_GLEAN_10010354 |
| Mo_GLEAN_10010386 |
| Mo_GLEAN_10010390 |
| Mo_GLEAN_10010391 |
| Mo_GLEAN_10010400 |
| Mo_GLEAN_10010406 |
| Mo_GLEAN_10010407 |
| Mo_GLEAN_10010428 |
| Mo_GLEAN_10010480 |
| Mo_GLEAN_10010481 |
| Mo_GLEAN_10010486 |
| Mo_GLEAN_10010496 |
| Mo_GLEAN_10010497 |
| Mo_GLEAN_10010500 |
| Mo_GLEAN_10010502 |
| Mo_GLEAN_10010503 |
| Mo_GLEAN_10010512 |
| Mo_GLEAN_10010513 |
| Mo_GLEAN_10010531 |
| Mo_GLEAN_10010557 |
| Mo_GLEAN_10010564 |
| Mo_GLEAN_10010566 |
| Mo_GLEAN_10010612 |
| Mo_GLEAN_10010662 |
| Mo_GLEAN_10010743 |
| Mo_GLEAN_10010744 |
| Mo_GLEAN_10010785 |
| Mo_GLEAN_10010818 |
| Mo_GLEAN_10010830 |
| Mo_GLEAN_10010905 |
| Mo_GLEAN_10010906 |
| Mo_GLEAN_10010965 |
| Mo_GLEAN_10010966 |
| Mo_GLEAN_10010999 |
| Mo_GLEAN_10011002 |
| Mo_GLEAN_10011004 |
| Mo_GLEAN_10011005 |
| Mo_GLEAN_10011017 |
| Mo_GLEAN_10011065 |
| Mo_GLEAN_10011070 |
| Mo_GLEAN_10011115 |
| Mo_GLEAN_10011125 |
| Mo_GLEAN_10011213 |
| Mo_GLEAN_10011214 |
| Mo_GLEAN_10011217 |
| Mo_GLEAN_10011220 |
| Mo_GLEAN_10011225 |
| Mo_GLEAN_10011228 |
| Mo_GLEAN_10011301 |
| Mo_GLEAN_10011305 |
| Mo_GLEAN_10011324 |
| Mo_GLEAN_10011325 |
| Mo_GLEAN_10011332 |
| Mo_GLEAN_10011357 |
| Mo_GLEAN_10011360 |
| Mo_GLEAN_10011386 |
| Mo_GLEAN_10011408 |
| Mo_GLEAN_10011460 |
| Mo_GLEAN_10011610 |
| Mo_GLEAN_10011832 |
| Mo_GLEAN_10011837 |
| Mo_GLEAN_10011841 |
| Mo_GLEAN_10011842 |
| Mo_GLEAN_10011852 |
| Mo_GLEAN_10011861 |
| Mo_GLEAN_10011881 |
| Mo_GLEAN_10011882 |
| Mo_GLEAN_10011984 |
| Mo_GLEAN_10011996 |
| Mo_GLEAN_10012030 |
| Mo_GLEAN_10012050 |
| Mo_GLEAN_10012074 |
| Mo_GLEAN_10012205 |
| Mo_GLEAN_10012308 |
| Mo_GLEAN_10012321 |
| Mo_GLEAN_10012356 |
| Mo_GLEAN_10012419 |
| Mo_GLEAN_10012561 |
| Mo_GLEAN_10012652 |
| Mo_GLEAN_10012661 |
| Mo_GLEAN_10012759 |
| Mo_GLEAN_10012782 |
| Mo_GLEAN_10012787 |
| Mo_GLEAN_10012796 |
| Mo_GLEAN_10012798 |
| Mo_GLEAN_10012808 |
| Mo_GLEAN_10012812 |
| Mo_GLEAN_10012819 |
| Mo_GLEAN_10012830 |
| Mo_GLEAN_10012833 |
| Mo_GLEAN_10012841 |
| Mo_GLEAN_10012854 |
| Mo_GLEAN_10012857 |
| Mo_GLEAN_10012874 |
| Mo_GLEAN_10012876 |
| Mo_GLEAN_10012907 |
| Mo_GLEAN_10012968 |
| Mo_GLEAN_10013006 |
| Mo_GLEAN_10013059 |
| Mo_GLEAN_10013072 |
| Mo_GLEAN_10013113 |
| Mo_GLEAN_10013141 |
| Mo_GLEAN_10013232 |
| Mo_GLEAN_10013234 |
| Mo_GLEAN_10013249 |
| Mo_GLEAN_10013298 |
| Mo_GLEAN_10013302 |
| Mo_GLEAN_10013334 |
| Mo_GLEAN_10013347 |
| Mo_GLEAN_10013401 |
| Mo_GLEAN_10013407 |
| Mo_GLEAN_10013452 |
| Mo_GLEAN_10013531 |
| Mo_GLEAN_10013551 |
| Mo_GLEAN_10013619 |
| Mo_GLEAN_10013624 |
| Mo_GLEAN_10013651 |
| Mo_GLEAN_10013718 |
| Mo_GLEAN_10013806 |
| Mo_GLEAN_10013808 |
| Mo_GLEAN_10013815 |
| Mo_GLEAN_10013840 |
| Mo_GLEAN_10013914 |
| Mo_GLEAN_10013931 |
| Mo_GLEAN_10013958 |
| Mo_GLEAN_10013962 |
| Mo_GLEAN_10013978 |
| Mo_GLEAN_10013987 |
| Mo_GLEAN_10013998 |
| Mo_GLEAN_10014005 |
| Mo_GLEAN_10014012 |
| Mo_GLEAN_10014023 |
