# Supplementary material for: Global Genome and Transcriptome Analyses of Magnaporthe oryzae Epidemic Isolate 98-06 Uncover Novel Effectors and Pathogenicity-Related Genes, Revealing Gene Gain and Lose Dynamics in Genome Evolution
Source: PLoS Pathog. 2015 Apr 2;11(4):e1004801. doi: 10.1371/journal.ppat.1004801 (PMC4383609; doi:10.1371/journal.ppat.1004801)
Supplement: S10 Table — (DOC) [file ppat.1004801.s025.doc]

**Table S10** **Transcriptome data of 64 known** **pathogenicity genes and 10 effectors of *M. oryzae*.**

| **GeneID** | **GeneID** | **MY** | **co-0h** | **co-8h** | **co-24h** | **co-48h** | **co-72h** | **cluster** |
| --- | --- | --- | --- | --- | --- | --- | --- | --- |
| Mo_GLEAN_10003260 | *VAM7* | 5.51 | 4.58 | 2.81 | 0.00 | 4.63 | 5.46 | a |
| Mo_GLEAN_10013106 | *SSO1* | 7.97 | 6.63 | 6.89 | 0.00 | 8.02 | 6.88 | a |
| Mo_GLEAN_10003882 | *PIC5* | 6.29 | 7.02 | 7.16 | 0.00 | 6.96 | 6.50 | a |
| Mo_GLEAN_10004839 | *SOM1* | 5.93 | 7.55 | 6.85 | 0.00 | 6.74 | 5.98 | a |
| Mo_GLEAN_10006209 | *HYR1* | 9.03 | 8.12 | 8.11 | 0.00 | 9.65 | 9.25 | a |
| Mo_GLEAN_10006493 | MGG_09560 | 4.80 | 3.99 | 5.42 | 0.00 | 4.89 | 4.75 | a |
| Mo_GLEAN_10006498 | MGG_09565 | 7.99 | 7.11 | 7.99 | 0.00 | 7.46 | 8.21 | a |
| Mo_GLEAN_10006745 | *MSB2* | 7.32 | 6.34 | 7.10 | 0.00 | 6.94 | 7.42 | a |
| Mo_GLEAN_10007138 | *DUO1* | 4.32 | 3.99 | 3.46 | 0.00 | 5.66 | 6.18 | a |
| Mo_GLEAN_10007146 | *CDC15* | 4.74 | 2.95 | 5.81 | 0.00 | 4.72 | 4.67 | a |
| Mo_GLEAN_10007618 | *CRZ1* | 8.08 | 8.31 | 6.38 | 0.00 | 4.68 | 6.08 | a |
| Mo_GLEAN_10007842 | *SPM1* | 7.47 | 6.30 | 6.46 | 0.00 | 6.44 | 6.88 | a |
| Mo_GLEAN_10008542 | *MGTA1* | 3.99 | 0.99 | 3.21 | 0.00 | 3.57 | 3.44 | a |
| Mo_GLEAN_10008554 | *TPS1* | 6.80 | 5.22 | 7.84 | 0.00 | 7.70 | 5.95 | a |
| Mo_GLEAN_10008707 | MGG_03977 | 1.19 | 3.79 | 3.35 | 0.00 | 2.47 | 5.08 | a |
| Mo_GLEAN_10008915 | *DES1* | 4.31 | 3.42 | 4.07 | 0.00 | 4.20 | 5.68 | a |
| Mo_GLEAN_10009197 | *CMK1* | 7.35 | 6.57 | 6.81 | 0.00 | 6.56 | 6.33 | a |
| Mo_GLEAN_10009783 | *HIK1* | 5.21 | 6.85 | 5.72 | 0.00 | 5.68 | 4.40 | a |
| Mo_GLEAN_10010637 | *HEX1* | 11.27 | 7.04 | 9.59 | 0.00 | 10.56 | 11.56 | a |
| Mo_GLEAN_10010710 | *NUT1* | 5.72 | 7.46 | 4.48 | 0.00 | 5.43 | 5.43 | a |
| Mo_GLEAN_10010734 | *MCM1* | 6.99 | 6.29 | 5.36 | 0.00 | 7.02 | 6.19 | a |
| Mo_GLEAN_10011076 | *GLK1* | 7.23 | 5.96 | 7.37 | 0.00 | 7.35 | 6.85 | a |
| Mo_GLEAN_10013326 | *MST7* | 6.64 | 7.07 | 6.35 | 0.00 | 6.55 | 7.03 | a |
| Mo_GLEAN_10010818 | *MC69* | 6.40 | 6.24 | 6.05 | 0.00 | 6.44 | 7.29 | a |
| Mo_GLEAN_10000043 | *BAS1* | 0.00 | 0.00 | 0.00 | 11.22 | 8.37 | 8.14 | b |
| Mo_GLEAN_10001463 | *BAS4* | 0.00 | 0.00 | 0.00 | 11.57 | 9.48 | 6.31 | b |
| Mo_GLEAN_10004708 | MGG_01822 | 6.75 | 6.21 | 7.47 | 7.78 | 9.09 | 8.20 | b |
| Mo_GLEAN_10007110 | MGG_02503 | 9.59 | 8.60 | 8.71 | 10.13 | 9.81 | 10.51 | b |
| Mo_GLEAN_10008494 | *SLP1* | 0.00 | 0.00 | 0.00 | 8.91 | 9.39 | 8.96 | b |
| Mo_GLEAN_10013958 | *BAS3* | 0.00 | 0.00 | 0.00 | 11.01 | 10.42 | 11.15 | b |
| Mo_GLEAN_10002512 | *Avr-Pizt* | 0.00 | 0.00 | 0.00 | 6.66 | 7.71 | 0.00 | b |
| Mo_GLEAN_10002287 | *MTP1* | 5.51 | 7.13 | 6.95 | 0.00 | 5.60 | 0.00 | c |
| Mo_GLEAN_10002673 | *PdeL* | 4.18 | 4.05 | 5.26 | 0.00 | 3.98 | 0.00 | c |
| Mo_GLEAN_10003704 | *PTH11* | 0.15 | 1.27 | 7.59 | 0.00 | 4.61 | 0.00 | c |
| Mo_GLEAN_10004178 | *HOX2* | 5.79 | 3.88 | 5.44 | 0.00 | 5.19 | 0.00 | c |
| Mo_GLEAN_10004972 | *Mnh6* | 9.53 | 10.14 | 9.03 | 0.00 | 9.26 | 0.00 | c |
| Mo_GLEAN_10006450 | MGG_09519 | 2.11 | 2.76 | 5.41 | 0.00 | 3.88 | 0.00 | c |
| Mo_GLEAN_10007062 | *LHS1* | 6.54 | 6.37 | 6.43 | 6.30 | 6.97 | 6.03 | c |
| Mo_GLEAN_10007433 | *CON7p* | 5.75 | 9.37 | 9.06 | 0.00 | 8.19 | 0.00 | c |
| Mo_GLEAN_10008306 | *PEX7* | 4.32 | 3.06 | 4.93 | 0.00 | 5.53 | 0.00 | c |
| Mo_GLEAN_10010288 | *SSD1* | 6.91 | 6.71 | 7.76 | 6.87 | 7.54 | 6.59 | c |
| Mo_GLEAN_10012596 | *ATG1* | 4.35 | 5.61 | 5.81 | 0.00 | 5.04 | 0.00 | c |
| Mo_GLEAN_10003140 | *MPG1* | 5.06 | 12.43 | 11.90 | 11.24 | 4.38 | 6.18 | d |
| Mo_GLEAN_10005188 | *PdeH* | 4.94 | 5.80 | 5.69 | 7.54 | 6.06 | 4.33 | d |
| Mo_GLEAN_10005779 | MGG_03580 | 5.37 | 7.05 | 6.99 | 7.32 | 5.91 | 6.06 | d |
| Mo_GLEAN_10005906 | *SPM1* | 9.48 | 10.71 | 11.30 | 10.65 | 10.23 | 9.34 | d |
| Mo_GLEAN_10007978 | *COM1* | 6.34 | 7.23 | 6.56 | 7.66 | 6.54 | 5.73 | d |
| Mo_GLEAN_10009486 | *SFL1* | 5.16 | 7.34 | 6.39 | 7.07 | 5.76 | 4.83 | d |
| Mo_GLEAN_10012208 | *TIG1* | 6.04 | 6.75 | 6.62 | 6.84 | 6.84 | 6.16 | d |
| Mo_GLEAN_10012724 | *HOX7* | 0.43 | 1.14 | 6.00 | 9.77 | 5.17 | 0.00 | d |
| Mo_GLEAN_10013222 | MGG_00883 | 5.03 | 6.60 | 5.65 | 5.64 | 5.04 | 4.01 | d |
| Mo_GLEAN_10013322 | MGG_00803 | 5.76 | 6.38 | 6.55 | 7.93 | 6.35 | 5.36 | d |
| Mo_GLEAN_10013858 | *MAGB* | 6.43 | 7.98 | 7.18 | 7.79 | 7.19 | 6.53 | d |
| Mo_GLEAN_10000004 | *PWL1* | 0.00 | 0.00 | 0.00 | 0.00 | 0.00 | 6.81 | e |
| Mo_GLEAN_10002499 | *BAS2* | 1.60 | 0.00 | 0.00 | 0.00 | 8.14 | 8.62 | e |
| Mo_GLEAN_10006037 | Cutinase2 | 0.00 | 0.70 | 4.77 | 0.00 | 5.01 | 7.56 | e |
| Mo_GLEAN_10013668 | *EMP1* | 9.37 | 6.32 | 9.43 | 9.14 | 10.25 | 10.80 | e |
| Mo_GLEAN_10004084 | *PDE1* | 3.63 | 2.20 | 2.89 | 6.01 | 2.21 | 4.76 | f |
| Mo_GLEAN_10006200 | *MCNA* | 6.37 | 7.22 | 6.91 | 8.08 | 6.29 | 7.72 | f |
| Mo_GLEAN_10010691 | *RBP35* | 5.83 | 6.57 | 6.51 | 7.53 | 5.43 | 6.27 | f |
| Mo_GLEAN_10004317 | *MoAAT* | 8.99 | 6.24 | 5.11 | 7.29 | 6.69 | 7.18 | g |
| Mo_GLEAN_10010405 | MGG_02531 | 4.96 | 1.31 | 0.00 | 0.00 | 0.00 | 0.00 | g |
| Mo_GLEAN_10011383 | *ACE1* | 9.04 | 7.27 | 7.05 | 7.32 | 6.37 | 7.27 | g |
| Mo_GLEAN_10004680 | *CHS1* | 6.79 | 8.68 | 7.51 | 6.99 | 7.06 | 6.83 | h |
| Mo_GLEAN_10010681 | *RAC1* | 7.63 | 8.74 | 8.12 | 7.62 | 7.58 | 7.93 | h |
| Mo_GLEAN_10012451 | *AP1* | 7.20 | 9.30 | 8.17 | 7.09 | 7.32 | 6.99 | h |
| Mo_GLEAN_10007781 | *MST12* | 6.13 | 7.26 | 6.06 | 6.84 | 6.80 | 6.89 | i |
| Mo_GLEAN_10007966 | *MIG1* | 6.02 | 7.62 | 5.31 | 6.81 | 6.93 | 5.55 | i |
| Mo_GLEAN_10008000 | *SSADH* | 8.04 | 5.98 | 7.44 | 7.78 | 7.52 | 7.82 | j |
| Mo_GLEAN_10008790 | *SEC22* | 5.81 | 5.25 | 5.73 | 0.00 | 0.00 | 6.24 | k |
| Mo_GLEAN_10010862 | *FLP1* | 0.00 | 0.82 | 0.00 | 0.00 | 4.33 | 0.00 | l |
| Mo_GLEAN_10007927 | *MHP1* | 0.59 | 0.00 | 6.28 | 0.00 | 0.00 | 7.30 | m |
| Mo_GLEAN_10002296 | *MSP1* | 11.76 | 9.98 | 11.69 | 11.73 | 12.45 | 10.04 | n |
| Mo_GLEAN_10000560 | *Avr-Pik* | 0.00 | 0.00 | 10.48 | 10.19 | 10.91 | 0.00 | n |
| Mo_GLEAN_10008712 | *ACTIN* | 10.64 | 9.08 | 10.30 | 10.16 | 10.39 | 10.58 |  |

a: Gene ID of 70-15 homologous to genes of 98-06. The E value ≤ 7e-70.

b: The value is log_2_(RPKM +1).
