# Supplementary material for: Global Genome and Transcriptome Analyses of Magnaporthe oryzae Epidemic Isolate 98-06 Uncover Novel Effectors and Pathogenicity-Related Genes, Revealing Gene Gain and Lose Dynamics in Genome Evolution
Source: PLoS Pathog. 2015 Apr 2;11(4):e1004801. doi: 10.1371/journal.ppat.1004801 (PMC4383609; doi:10.1371/journal.ppat.1004801)
Supplement: S11 Table — (DOC) [file ppat.1004801.s026.doc]

**Table S11** **CAST assay of 21** **SNARE genes.**

| **Gene Name** | **MY ^a^** | **co-0h** | **co-8h** | **co-24h** | **co-48h** | **co-72h** | **Cluster^b^** | |
| --- | --- | --- | --- | --- | --- | --- | --- | --- |
| *MoSEC9* | 7.70 | 8.27 | 7.63 | 8.40 | 6.86 | 7.30 | a |  |
| *MoSSO2* | 7.10 | 7.78 | 6.19 | 7.29 | 5.35 | 7.33 | a |  |
| *MoSSO1* | 7.97 | 6.63 | 6.89 | 0.00 | 8.02 | 6.88 | b |  |
| *MoVTI1* | 5.93 | 6.61 | 6.54 | 0.00 | 4.33 | 5.15 | b |  |
| *MoSEC20* | 4.42 | 4.63 | 5.73 | 0.00 | 5.40 | 5.26 | b |  |
| *MoNYV1* | 5.05 | 7.10 | 5.60 | 0.00 | 7.33 | 6.11 | b |  |
| *MoVAM3* | 6.64 | 6.79 | 6.87 | 0.00 | 7.14 | 6.49 | b |  |
| *MoSEC22* | 5.81 | 5.25 | 5.73 | 0.00 | 0.00 | 6.24 | c |  |
| *MoUFE1* | 4.92 | 3.38 | 4.97 | 0.00 | 3.99 | 6.77 | b |  |
| *MoVAM7* | 5.51 | 4.58 | 2.81 | 0.00 | 4.63 | 5.46 | b |  |
| *MoYKT6* | 7.13 | 7.85 | 7.59 | 0.00 | 7.88 | 7.93 | b |  |
| *MoTLG2* | 4.97 | 5.53 | 6.32 | 0.00 | 5.84 | 5.53 | b |  |
| *MoBET1* | 6.48 | 7.07 | 5.65 | 0.00 | 6.90 | 5.32 | b |  |
| *MoUSE1* | 4.26 | 3.21 | 4.32 | 0.00 | 3.75 | 5.53 | b |  |
| *MoSED5* | 5.28 | 4.73 | 6.55 | 0.00 | 5.78 | 0.00 | d |  |
| *MoSFT1* | 6.05 | 6.56 | 7.70 | 0.00 | 6.12 | 0.00 | d |  |
| *MoSYN8* | 4.43 | 3.28 | 4.89 | 0.00 | 3.18 | 0.00 | d |  |
| *MoTLG1* | 5.44 | 5.93 | 5.86 | 0.00 | 5.73 | 0.00 | d |  |
| *MoBOS1* | 4.84 | 4.80 | 3.92 | 0.00 | 5.26 | 0.00 | d |  |
| *MoGOS1* | 5.20 | 5.29 | 6.41 | 9.01 | 4.93 | 0.00 | e |  |
| *MoSNC1* | 8.43 | 8.88 | 8.95 | 10.28 | 8.91 | 8.33 | e |  |

a: The value is log_2_(RPKM +1).

b: SNARE genes due to five different expression patterns respectively.
