# Supplementary material for: Global Genome and Transcriptome Analyses of Magnaporthe oryzae Epidemic Isolate 98-06 Uncover Novel Effectors and Pathogenicity-Related Genes, Revealing Gene Gain and Lose Dynamics in Genome Evolution
Source: PLoS Pathog. 2015 Apr 2;11(4):e1004801. doi: 10.1371/journal.ppat.1004801 (PMC4383609; doi:10.1371/journal.ppat.1004801)
Supplement: S12 Table — (DOC) [file ppat.1004801.s027.doc]

**Table S12** **CAST assay of 35 endocytosis-related genes.**

| **Gene ID** | **70-15 ^a^** | **Annotation** | **MY ^b^** | **co-0h** | **co-8h** | **co-24h** | **co-48h** | **co-72h** | **Cluster^c^** |
| --- | --- | --- | --- | --- | --- | --- | --- | --- | --- |
| Mo_GLEAN_10013525 | MGG_00631 | conserved hypothetical protein | 8.11 | 3.99 | 3.32 | 0.00 | 4.88 | 4.74 | a |
| Mo_GLEAN_10013390 | MGG_00748 | myosin-1 | 6.14 | 6.28 | 6.84 | 6.99 | 5.74 | 6.31 | b |
| Mo_GLEAN_10012944 | MGG_01121 | vesicular-fusion protein SEC17 | 7.14 | 5.48 | 7.71 | 0.00 | 7.19 | 6.80 | a |
| Mo_GLEAN_10008411 | MGG_01574 | ADP-ribosylation factor | 4.89 | 4.81 | 4.68 | 0.00 | 5.63 | 0.00 | c |
| Mo_GLEAN_10007219 | MGG_02418 | vesicular-fusion protein SEC18 | 5.60 | 5.50 | 6.58 | 6.56 | 6.19 | 4.91 | d |
| Mo_GLEAN_10007104 | MGG_02506 | hypothetical protein | 4.03 | 3.35 | 4.90 | 0.00 | 4.17 | 5.40 | a |
| Mo_GLEAN_10010768 | MGG_02802 | predicted protein | 5.48 | 5.64 | 6.06 | 6.91 | 4.82 | 5.65 | b |
| Mo_GLEAN_10010951 | MGG_02949 | cytoskeleton assembly control protein Sla2 | 6.42 | 5.33 | 6.11 | 7.23 | 6.02 | 6.55 | b |
| Mo_GLEAN_10011100 | MGG_03060 | myosin type II heavy chain | 3.29 | 1.83 | 3.50 | 5.06 | 3.36 | 2.95 | f |
| Mo_GLEAN_10012068 | MGG_03313 | vacuolar assembly protein | 4.18 | 2.83 | 5.30 | 5.87 | 4.12 | 3.69 | b |
| Mo_GLEAN_10008585 | MGG_03879 | actin-like protein 3 | 7.37 | 4.54 | 7.68 | 0.00 | 7.60 | 6.54 | b |
| Mo_GLEAN_10008838 | MGG_04095 | vacuolar-sorting protein SNF7 | 7.30 | 7.36 | 7.45 | 0.00 | 7.55 | 7.97 | a |
| Mo_GLEAN_10011394 | MGG_04438 | ADP-ribosylation factor | 9.36 | 9.54 | 9.42 | 7.74 | 9.69 | 9.63 | a |
| Mo_GLEAN_10004985 | MGG_04478 | fimbrin | 7.64 | 5.23 | 8.24 | 5.93 | 7.78 | 6.65 | a |
| Mo_GLEAN_10011487 | MGG_04616 | epsin-1 | 7.23 | 6.86 | 7.48 | 7.04 | 7.16 | 7.57 | e |
| Mo_GLEAN_10007803 | MGG_04976 | ADP-ribosylation factor-like protein 1 | 6.09 | 6.86 | 5.11 | 0.00 | 6.91 | 6.59 | e |
| Mo_GLEAN_10001157 | MGG_05528 | hob3 | 6.39 | 6.00 | 6.82 | 0.00 | 7.14 | 7.49 | a |
| Mo_GLEAN_10005237 | MGG_05626 | cytoskeleton assembly control protein SLA1p | 5.33 | 6.71 | 5.96 | 6.18 | 6.34 | 5.91 | a |
| Mo_GLEAN_10006570 | MGG_06180 | actin cytoskeleton-regulatory complex protein END3 | 5.51 | 4.16 | 6.17 | 0.00 | 6.10 | 6.68 | a |
| Mo_GLEAN_10012638 | MGG_06358 | amylase-binding protein AbpA | 6.84 | 4.10 | 6.90 | 0.00 | 7.11 | 6.35 | a |
| Mo_GLEAN_10012635 | MGG_06361 | dynamin-A | 5.78 | 6.92 | 5.91 | 0.00 | 6.08 | 6.93 | a |
| Mo_GLEAN_10012602 | MGG_06389 | coronin-6 | 6.89 | 6.33 | 6.87 | 0.00 | 6.68 | 6.32 | a |
| Mo_GLEAN_10009601 | MGG_06876 | clathrin light chain | 6.69 | 6.39 | 6.45 | 0.00 | 6.43 | 6.12 | a |
| Mo_GLEAN_10002744 | MGG_07768 | clathrin heavy chain | 6.81 | 7.24 | 7.05 | 6.55 | 7.05 | 6.53 | c |
| Mo_GLEAN_10011740 | MGG_08859 | ADP-ribosylation factor family protein | 5.26 | 5.25 | 4.54 | 0.00 | 4.22 | 5.04 | a |
| Mo_GLEAN_10006448 | MGG_09517 | vacuolar protein sorting-associated protein 1 | 6.32 | 6.19 | 6.57 | 0.00 | 6.22 | 6.71 | a |
| Mo_GLEAN_10005342 | MGG_09837 | vacuolar segregation protein pep7 | 5.28 | 5.81 | 6.97 | 0.00 | 5.93 | 0.00 | c |
| Mo_GLEAN_10009210 | MGG_09902 | F-actin-capping protein subunit beta | 6.82 | 3.76 | 5.99 | 0.00 | 6.14 | 6.41 | a |
| Mo_GLEAN_10003598 | MGG_10676 | ADP-ribosylation factor 6 | 5.89 | 5.15 | 3.71 | 0.00 | 5.92 | 0.00 | c |
| Mo_GLEAN_10008057 | MGG_11243 | hypothetical protein | 6.00 | 5.97 | 6.34 | 8.39 | 4.89 | 5.15 | b |
| Mo_GLEAN_10012462 | MGG_12818 | F-actin-capping protein subunit alpha | 7.15 | 4.05 | 7.33 | 8.16 | 6.74 | 8.05 | g |
| Mo_GLEAN_10007405 | MGG_12887 | ADP-ribosylation factor 1 | 5.02 | 0.00 | 0.00 | 0.00 | 5.30 | 7.13 | h |
| Mo_GLEAN_10003597 | MGG_13931 | CMGC/CDK/CDK8 protein kinase | 5.11 | 3.46 | 3.49 | 0.00 | 3.47 | 0.00 | c |
| Mo_GLEAN_10004209 | MGG_14713 | hypothetical protein | 4.22 | 5.08 | 4.34 | 7.38 | 4.32 | 3.64 | b |
| Mo_GLEAN_10003613 | MGG_17001 | actin-like protein 2 | 7.13 | 4.38 | 6.73 | 0.00 | 6.09 | 8.15 | a |

a: Gene ID of 70-15 homologous to genes of 98-06. The E value ≤ 2e-34.

b: The value is log_2_ (RPKM +1).

c: Genes due to eight different expression patterns respectively.
