# Supplementary material for: Global Genome and Transcriptome Analyses of Magnaporthe oryzae Epidemic Isolate 98-06 Uncover Novel Effectors and Pathogenicity-Related Genes, Revealing Gene Gain and Lose Dynamics in Genome Evolution
Source: PLoS Pathog. 2015 Apr 2;11(4):e1004801. doi: 10.1371/journal.ppat.1004801 (PMC4383609; doi:10.1371/journal.ppat.1004801)
Supplement: S13 Table — (DOC) [file ppat.1004801.s028.doc]

**Table S13** **134 candidate effectors.**

| **Gene ID** | **MY ^a^** | **co-0h** | **co-8h** | **co-24h** | **co-48h** | **co-72h** | **70-15 ^b^** |
| --- | --- | --- | --- | --- | --- | --- | --- |
| Mo_GLEAN_10000004 | 0.00 | 0.00 | 0.00 | 0.00 | 0.00 | 6.81 | MGG_04301 |
| Mo_GLEAN_10000043 | 0.00 | 0.00 | 0.00 | 11.22 | 8.37 | 8.14 | MGG_04795 |
| Mo_GLEAN_10000059 | 0.64 | 0.00 | 0.00 | 8.71 | 8.49 | 7.03 | MGG_16171 |
| Mo_GLEAN_10000191 | 0.00 | 0.00 | 0.00 | 9.76 | 10.09 | 9.49 | - |
| Mo_GLEAN_10000195 | 2.50 | 0.00 | 3.47 | 9.45 | 7.78 | 7.76 | - |
| Mo_GLEAN_10000256 | 0.00 | 1.59 | 0.00 | 0.00 | 8.06 | 8.24 | MGG_14725 |
| Mo_GLEAN_10000529 | 0.00 | 0.00 | 0.00 | 0.00 | 0.00 | 6.59 | - |
| *Avr-Pik/km/kp* | 0.00 | 0.00 | 0.00 | 10.76 | 10.19 | 10.97 | MGG_15972 |
| Mo_GLEAN_10000613 | 0.00 | 0.00 | 0.00 | 0.00 | 6.34 | 6.19 | MGG_17556 |
| Mo_GLEAN_10000617 | 0.00 | 0.00 | 0.00 | 0.00 | 10.15 | 10.88 | - |
| Mo_GLEAN_10000664 | 0.00 | 0.00 | 0.00 | 8.90 | 9.86 | 10.32 | MGG_17301 |
| Mo_GLEAN_10000714 | 0.00 | 0.00 | 0.00 | 10.28 | 11.14 | 11.01 | MGG_14156 |
| Mo_GLEAN_10000730 | 0.00 | 0.00 | 0.00 | 0.00 | 4.48 | 6.28 | - |
| Mo_GLEAN_10000765 | 2.90 | 0.00 | 3.90 | 10.50 | 7.60 | 0.00 | - |
| Mo_GLEAN_10000831 | 6.38 | 6.59 | 5.63 | 0.00 | 10.91 | 11.53 | MGG_15267 |
| Mo_GLEAN_10000858 | 0.00 | 0.00 | 0.00 | 9.88 | 10.92 | 10.19 | MGG_16570 |
| Mo_GLEAN_10000914 | 3.56 | 0.00 | 0.00 | 0.00 | 5.30 | 5.57 | MGG_18108 |
| Mo_GLEAN_10000919 | 1.46 | 5.60 | 7.65 | 9.95 | 8.32 | 8.09 | MGG_09134 |
| Mo_GLEAN_10000926 | 0.00 | 0.00 | 0.00 | 0.00 | 0.00 | 6.06 | MGG_18105 |
| Mo_GLEAN_10000959 | 4.61 | 1.47 | 0.00 | 0.00 | 9.27 | 9.73 | MGG_14523 |
| Mo_GLEAN_10000960 | 0.00 | 0.00 | 0.00 | 8.54 | 7.88 | 7.27 | - |
| Mo_GLEAN_10000981 | 0.00 | 0.00 | 0.00 | 8.94 | 8.61 | 9.24 | MGG_16811 |
| Mo_GLEAN_10001004 | 0.00 | 0.00 | 0.00 | 0.00 | 3.81 | 5.58 | MGG_02239 |
| Mo_GLEAN_10001126 | 0.00 | 0.00 | 0.00 | 0.00 | 5.74 | 6.99 | MGG_16693 |
| Mo_GLEAN_10001172 | 0.00 | 0.00 | 0.00 | 0.00 | 5.80 | 5.66 | MGG_05538 |
| Mo_GLEAN_10001323 | 0.00 | 0.00 | 0.00 | 0.00 | 5.63 | 5.90 | MGG_16977 |
| Mo_GLEAN_10001331 | 9.39 | 1.21 | 0.00 | 0.00 | 7.78 | 8.90 | MGG_07969 |
| Mo_GLEAN_10001463 | 0.00 | 0.00 | 0.00 | 11.57 | 9.48 | 6.31 | MGG_10914 |
| Mo_GLEAN_10001495 | 0.00 | 0.00 | 0.00 | 9.20 | 6.67 | 0.00 | MGG_10020 |
| Mo_GLEAN_10001503 | 0.00 | 0.00 | 0.00 | 0.00 | 4.71 | 6.52 | MGG_17244 |
| Mo_GLEAN_10001521 | 6.15 | 4.72 | 2.94 | 0.00 | 8.05 | 5.61 | - |
| Mo_GLEAN_10001542 | 0.89 | 1.96 | 0.00 | 0.00 | 6.61 | 7.04 | MGG_07624 |
| Mo_GLEAN_10001543 | 3.74 | 2.01 | 0.00 | 0.00 | 8.05 | 8.68 | MGG_07625 |
| Mo_GLEAN_10001550 | 9.77 | 1.71 | 0.00 | 8.38 | 11.09 | 12.00 | MGG_07630 |
| Mo_GLEAN_10001611 | 0.38 | 0.00 | 2.91 | 7.83 | 7.28 | 7.55 | MGG_04776 |
| Mo_GLEAN_10001679 | 0.00 | 0.00 | 0.00 | 8.41 | 6.62 | 6.15 | MGG_01953 |
| Mo_GLEAN_10001825 | 2.63 | 0.00 | 0.00 | 0.00 | 4.17 | 6.96 | MGG_01974 |
| Mo_GLEAN_10001939 | 0.00 | 0.00 | 0.00 | 9.41 | 8.09 | 7.73 | MGG_15459 |
| Mo_GLEAN_10001994 | 0.00 | 0.00 | 0.00 | 0.00 | 6.58 | 8.83 | MGG_07900 |
| Mo_GLEAN_10002499 | 1.60 | 0.00 | 0.00 | 0.00 | 8.14 | 8.62 | MGG_09693 |
| *Avr-Pizt* | 0.00 | 0.00 | 0.00 | 0.00 | 6.71 | 7.82 | MGG_18041 |
| Mo_GLEAN_10002544 | 0.00 | 0.00 | 0.00 | 0.00 | 8.81 | 8.27 | - |
| Mo_GLEAN_10002566 | 5.62 | 1.25 | 2.77 | 0.00 | 5.87 | 6.40 | MGG_09019 |
| Mo_GLEAN_10002596 | 0.00 | 0.00 | 0.00 | 8.08 | 7.86 | 6.81 | MGG_17249 |
| Mo_GLEAN_10002773 | 8.95 | 1.78 | 4.82 | 9.47 | 8.88 | 10.41 | MGG_08300 |
| Mo_GLEAN_10002901 | 0.00 | 0.00 | 5.06 | 0.00 | 7.01 | 7.86 | MGG_00047 |
| Mo_GLEAN_10002904 | 0.00 | 0.00 | 0.00 | 9.21 | 5.12 | 0.00 | MGG_00043 |
| Mo_GLEAN_10002989 | 3.11 | 0.00 | 0.00 | 0.00 | 8.09 | 8.63 | MGG_08348 |
| Mo_GLEAN_10003006 | 5.72 | 1.75 | 0.00 | 0.00 | 9.94 | 10.79 | MGG_08355 |
| Mo_GLEAN_10003024 | 1.12 | 0.00 | 3.25 | 0.00 | 4.15 | 5.95 | MGG_08376 |
| Mo_GLEAN_10003072 | 0.00 | 0.00 | 0.00 | 7.63 | 3.62 | 0.00 | MGG_08411 |
| Mo_GLEAN_10003110 | 0.00 | 1.12 | 0.00 | 0.00 | 8.19 | 8.46 | MGG_15443 |
| Mo_GLEAN_10003137 | 8.20 | 0.00 | 0.00 | 0.00 | 10.57 | 12.09 | MGG_10318 |
| Mo_GLEAN_10003187 | 0.00 | 0.00 | 0.00 | 9.21 | 8.19 | 8.35 | MGG_10276 |
| Mo_GLEAN_10003208 | 4.08 | 0.00 | 3.78 | 0.00 | 7.24 | 6.52 | MGG_10259 |
| Mo_GLEAN_10003209 | 0.00 | 0.00 | 0.00 | 8.48 | 5.38 | 7.21 | MGG_05389 |
| Mo_GLEAN_10003229 | 0.00 | 0.00 | 0.00 | 9.03 | 9.94 | 11.07 | MGG_16353 |
| Mo_GLEAN_10003240 | 0.00 | 0.00 | 0.00 | 9.83 | 8.40 | 7.56 | MGG_05410 |
| Mo_GLEAN_10003247 | 0.00 | 0.00 | 0.00 | 8.05 | 5.53 | 6.78 | MGG_05416 |
| Mo_GLEAN_10003292 | 4.92 | 4.73 | 2.76 | 0.00 | 8.11 | 9.77 | MGG_05456 |
| Mo_GLEAN_10003434 | 0.00 | 2.23 | 0.00 | 0.00 | 8.17 | 6.23 | MGG_02154 |
| Mo_GLEAN_10003436 | 0.00 | 0.00 | 0.00 | 8.56 | 4.49 | 6.30 | MGG_02166 |
| Mo_GLEAN_10003490 | 0.00 | 0.00 | 0.00 | 7.88 | 8.21 | 0.00 | MGG_08506 |
| Mo_GLEAN_10003505 | 0.00 | 0.00 | 0.00 | 9.33 | 5.81 | 0.00 | MGG_08491 |
| Mo_GLEAN_10003508 | 0.79 | 0.00 | 0.00 | 8.10 | 7.15 | 5.85 | MGG_08482 |
| Mo_GLEAN_10003562 | 0.00 | 0.00 | 0.00 | 8.79 | 6.48 | 5.54 | MGG_08441 |
| Mo_GLEAN_10003576 | 0.55 | 1.14 | 0.00 | 0.00 | 0.00 | 6.19 | MGG_08428 |
| Mo_GLEAN_10003752 | 9.61 | 0.00 | 4.27 | 9.30 | 9.98 | 11.11 | MGG_16714 |
| Mo_GLEAN_10003761 | 0.00 | 0.00 | 0.00 | 0.00 | 4.59 | 6.40 | MGG_08515 |
| Mo_GLEAN_10003808 | 0.48 | 3.73 | 0.00 | 0.00 | 5.13 | 9.41 | MGG_08546 |
| Mo_GLEAN_10003865 | 0.00 | 2.69 | 0.00 | 0.00 | 4.96 | 6.78 | MGG_14006 |
| Mo_GLEAN_10003900 | 0.00 | 0.00 | 0.00 | 9.85 | 9.09 | 8.32 | MGG_11072 |
| Mo_GLEAN_10003936 | 0.00 | 0.00 | 0.00 | 10.73 | 8.19 | 8.20 | MGG_08715 |
| Mo_GLEAN_10004032 | 1.88 | 0.00 | 0.00 | 0.00 | 6.59 | 8.44 | MGG_08799 |
| Mo_GLEAN_10004050 | 4.77 | 0.00 | 0.00 | 0.00 | 9.36 | 9.55 | MGG_08817 |
| Mo_GLEAN_10004096 | 1.38 | 0.00 | 0.00 | 0.00 | 6.53 | 8.54 | MGG_00120 |
| Mo_GLEAN_10004492 | 0.00 | 0.00 | 0.00 | 0.00 | 4.40 | 6.20 | MGG_09675 |
| Mo_GLEAN_10004507 | 3.11 | 2.22 | 0.00 | 0.00 | 7.35 | 7.79 | MGG_09657 |
| Mo_GLEAN_10004538 | 0.00 | 0.00 | 0.00 | 0.00 | 8.47 | 7.42 | MGG_09629 |
| Mo_GLEAN_10004541 | 0.00 | 0.00 | 0.00 | 0.00 | 6.84 | 7.28 | MGG_09627 |
| Mo_GLEAN_10004570 | 0.00 | 0.00 | 0.00 | 8.74 | 9.04 | 7.79 | MGG_09605 |
| Mo_GLEAN_10004913 | 0.00 | 0.00 | 0.00 | 0.00 | 0.00 | 5.51 | MGG_04579 |
| Mo_GLEAN_10004942 | 0.00 | 0.00 | 0.00 | 0.00 | 6.44 | 7.29 | MGG_04546 |
| Mo_GLEAN_10005055 | 1.10 | 0.00 | 0.00 | 9.73 | 8.93 | 8.46 | - |
| Mo_GLEAN_10005083 | 3.52 | 3.47 | 4.47 | 0.00 | 6.15 | 7.25 | MGG_05751 |
| Mo_GLEAN_10005262 | 0.00 | 0.00 | 0.00 | 9.20 | 5.11 | 0.00 | MGG_13133 |
| Mo_GLEAN_10005434 | 6.86 | 6.50 | 8.11 | 9.76 | 8.91 | 9.07 | MGG_10720 |
| Mo_GLEAN_10005523 | 1.67 | 1.52 | 0.00 | 0.00 | 8.66 | 8.40 | MGG_09378 |
| Mo_GLEAN_10005524 | 0.00 | 0.00 | 0.00 | 0.00 | 9.44 | 8.90 | MGG_09379 |
| Mo_GLEAN_10005785 | 0.46 | 0.00 | 0.00 | 0.00 | 5.61 | 6.86 | MGG_03585 |
| Mo_GLEAN_10005990 | 1.59 | 2.62 | 4.91 | 10.55 | 10.67 | 12.31 | MGG_16585 |
| Mo_GLEAN_10006247 | 1.32 | 0.00 | 0.00 | 0.00 | 6.02 | 6.86 | MGG_16647 |
| Mo_GLEAN_10007044 | 4.76 | 5.57 | 5.02 | 8.09 | 6.97 | 6.82 | - |
| Mo_GLEAN_10007320 | 1.82 | 0.00 | 0.00 | 0.00 | 7.98 | 8.61 | MGG_16058 |
| Mo_GLEAN_10007351 | 0.00 | 0.00 | 0.00 | 0.00 | 6.67 | 6.20 | MGG_02220 |
| Mo_GLEAN_10007354 | 0.53 | 0.00 | 0.00 | 0.00 | 5.84 | 7.10 | MGG_02223 |
| Mo_GLEAN_10007593 | 2.43 | 1.85 | 0.00 | 0.00 | 7.44 | 8.29 | MGG_12930 |
| Mo_GLEAN_10007624 | 0.00 | 0.00 | 0.00 | 0.00 | 3.89 | 5.67 | MGG_05127 |
| Mo_GLEAN_10007653 | 4.71 | 2.84 | 4.75 | 0.00 | 6.11 | 5.97 | MGG_16869 |
| Mo_GLEAN_10007675 | 6.37 | 5.49 | 7.41 | 9.06 | 8.39 | 8.11 | MGG_05083 |
| Mo_GLEAN_10007910 | 2.97 | 0.00 | 3.36 | 9.33 | 9.98 | 10.30 | MGG_16026 |
| Mo_GLEAN_10008274 | 0.64 | 0.00 | 3.70 | 0.00 | 5.92 | 5.46 | MGG_01455 |
| Mo_GLEAN_10008423 | 1.68 | 0.00 | 0.00 | 9.11 | 7.31 | 6.84 | MGG_17239 |
| Mo_GLEAN_10008494 | 0.00 | 0.00 | 0.00 | 8.91 | 9.39 | 8.96 | MGG_10097 |
| Mo_GLEAN_10008560 | 9.02 | 2.69 | 4.02 | 9.05 | 8.24 | 9.58 | MGG_03865 |
| Mo_GLEAN_10009024 | 1.51 | 0.00 | 0.00 | 0.00 | 7.18 | 5.60 | MGG_04263 |
| Mo_GLEAN_10009507 | 8.00 | 3.76 | 0.00 | 0.00 | 6.03 | 7.87 | MGG_06953 |
| Mo_GLEAN_10009686 | 0.00 | 0.00 | 0.00 | 0.00 | 7.88 | 7.74 | MGG_16175 |
| Mo_GLEAN_10009734 | 1.93 | 1.49 | 0.00 | 0.00 | 0.00 | 6.77 | MGG_07355 |
| Mo_GLEAN_10009741 | 0.00 | 0.00 | 0.00 | 0.00 | 5.40 | 7.23 | MGG_07348 |
| Mo_GLEAN_10010354 | 6.44 | 3.14 | 4.52 | 0.00 | 6.45 | 7.30 | MGG_08024 |
| Mo_GLEAN_10010406 | 0.00 | 0.00 | 0.00 | 11.11 | 8.40 | 9.84 | MGG_18036 |
| Mo_GLEAN_10010428 | 0.00 | 0.00 | 0.00 | 0.00 | 6.26 | 7.78 | MGG_02546 |
| Mo_GLEAN_10010500 | 1.44 | 1.30 | 0.00 | 10.32 | 9.92 | 9.92 | MGG_12655 |
| Mo_GLEAN_10010743 | 0.66 | 1.31 | 4.70 | 11.66 | 6.64 | 8.48 | MGG_02778 |
| Mo_GLEAN_10011004 | 0.00 | 0.00 | 0.00 | 0.00 | 9.67 | 9.11 | MGG_17896 |
| Mo_GLEAN_10011213 | 0.34 | 0.00 | 0.00 | 8.63 | 4.56 | 0.00 | MGG_10455 |
| Mo_GLEAN_10011220 | 0.60 | 0.00 | 6.51 | 9.93 | 3.60 | 6.35 | MGG_10460 |
| Mo_GLEAN_10011842 | 4.65 | 0.00 | 0.00 | 0.00 | 9.36 | 10.73 | MGG_08944 |
| Mo_GLEAN_10011861 | 1.40 | 0.00 | 0.00 | 0.00 | 0.00 | 7.41 | MGG_08971 |
| Mo_GLEAN_10011882 | 0.00 | 0.00 | 0.00 | 0.00 | 5.40 | 6.24 | MGG_08992 |
| Mo_GLEAN_10012050 | 10.40 | 6.66 | 0.00 | 0.00 | 9.41 | 11.19 | MGG_03326 |
| Mo_GLEAN_10012356 | 0.61 | 0.00 | 0.00 | 0.00 | 4.56 | 7.36 | MGG_17155 |
| Mo_GLEAN_10012652 | 2.64 | 0.88 | 3.01 | 0.00 | 6.40 | 7.99 | MGG_06347 |
| Mo_GLEAN_10012798 | 0.00 | 0.00 | 0.00 | 8.18 | 5.66 | 6.91 | MGG_06224 |
| Mo_GLEAN_10012808 | 0.55 | 0.00 | 0.00 | 0.00 | 0.00 | 7.18 | - |
| Mo_GLEAN_10012876 | 0.00 | 1.59 | 0.00 | 0.00 | 0.00 | 5.36 | MGG_10798 |
| Mo_GLEAN_10013072 | 7.96 | 5.91 | 8.24 | 10.57 | 8.11 | 9.17 | MGG_01009 |
| Mo_GLEAN_10013551 | 1.70 | 1.96 | 0.00 | 0.00 | 5.05 | 8.19 | MGG_00614 |
| Mo_GLEAN_10013619 | 4.73 | 0.00 | 0.00 | 0.00 | 7.71 | 6.57 | MGG_17425 |
| Mo_GLEAN_10013815 | 1.55 | 0.00 | 0.00 | 0.00 | 0.00 | 6.65 | MGG_17464 |
| Mo_GLEAN_10013840 | 0.00 | 0.00 | 0.00 | 8.00 | 5.89 | 6.74 | MGG_00380 |
| Mo_GLEAN_10013958 | 0.00 | 0.00 | 0.00 | 11.01 | 10.42 | 11.15 | MGG_11610 |
| Mo_GLEAN_10013962 | 12.62 | 2.70 | 4.03 | 0.00 | 11.18 | 11.66 | MGG_00283 |
| Mo_GLEAN_10013998 | 5.80 | 2.77 | 5.81 | 0.00 | 7.93 | 9.71 | MGG_00245 |
| Mo_GLEAN_10014005 | 0.40 | 0.00 | 0.00 | 9.90 | 8.90 | 8.95 | MGG_00230 |

a: log_2_ (RPKM +1).

b: Gene ID of 70-15 homologous to genes of 98-06. “-“ : Isolate-unique genes of 98-06.
