# Supplementary material for: Global Genome and Transcriptome Analyses of Magnaporthe oryzae Epidemic Isolate 98-06 Uncover Novel Effectors and Pathogenicity-Related Genes, Revealing Gene Gain and Lose Dynamics in Genome Evolution
Source: PLoS Pathog. 2015 Apr 2;11(4):e1004801. doi: 10.1371/journal.ppat.1004801 (PMC4383609; doi:10.1371/journal.ppat.1004801)
Supplement: S15 Table — (DOC) [file ppat.1004801.s030.doc]

**Table S15** **Primers used in this study.**

| **Primer Name** | **Sequence(5’-3’)** | **Purpose** |
| --- | --- | --- |
| Mo_GLEAN_10009978-RT-F | GTAGAGATGGTGCGTGAGCA | qRT-PCR for validation of the RNA-Seq profiling |
| Mo_GLEAN_10009978-RT-R | TGGCCAGAAAGCAAACTTCT | qRT-PCR for validation of the RNA-Seq profiling |
| Mo_GLEAN_10004630-RT-F | CTCTTTTCCAAGCCACTTGC | qRT-PCR for validation of the RNA-Seq profiling |
| Mo_GLEAN_10004630-RT-R | TATGAGCTCGCCAAGTGATG | qRT-PCR for validation of the RNA-Seq profiling |
| Mo_GLEAN_10013735-RT-F | CAACATAAACTGGCGGACCT | qRT-PCR for validation of the RNA-Seq profiling |
| Mo_GLEAN_10013735-RT-R | TTCCCTCCTCAGCTCTGTGT | qRT-PCR for validation of the RNA-Seq profiling |
| Mo_GLEAN_10012162-RT-F | GTGTTGTCGATGCCATGTTC | qRT-PCR for validation of the RNA-Seq profiling |
| Mo_GLEAN_10012162-RT-R | CGGAAGCTGCTCAGATAACC | qRT-PCR for validation of the RNA-Seq profiling |
| Mo_GLEAN_10003589-RT-F | GCTTTGTCGATACCCAAGGA | qRT-PCR for validation of the RNA-Seq profiling |
| Mo_GLEAN_10003589-RT-R | CGCATTTTCCTCTTCCTCAG | qRT-PCR for validation of the RNA-Seq profiling |
| BGIOSGA028607-RT-F | GTACGAAGGATTGGGCTTGA | qRT-PCR for validation of the RNA-Seq profiling |
| BGIOSGA028607-RT-R | CCAAGCTCCAAGAACAGAGG | qRT-PCR for validation of the RNA-Seq profiling |
| BGIOSGA014872-RT-F | GCTCAGGAAAAAGGTTGCTG | qRT-PCR for validation of the RNA-Seq profiling |
| BGIOSGA014872-RT-R | GAGGAGCGAGAACAGGATTG | qRT-PCR for validation of the RNA-Seq profiling |
| BGIOSGA006999-RT-F | GCTCAGGAAAAAGGTTGCTG | qRT-PCR for validation of the RNA-Seq profiling |
| BGIOSGA006999-RT-R | CACCTACGCACAATGATCCA | qRT-PCR for validation of the RNA-Seq profiling |
| BGIOSGA019638-RT-F | TGGGGACTCCATTGTTTCTC | qRT-PCR for validation of the RNA-Seq profiling |
| BGIOSGA019638-RT-R | AAAAAGGCATTCCGATTGTG | qRT-PCR for validation of the RNA-Seq profiling |
| rice-actin-F | GCGTGGACAAAGTTTTCAACCG | qRT-PCR for validation of the RNA-Seq profiling |
| rice-actin-R | TCTGGTACCCTCATCAGGCATC | qRT-PCR for validation of the RNA-Seq profiling |
| PR1a-RT-F | GGAAGTACGGCGAGAACATC | qRT-PCR for defense-related genes in rice |
| PR1a-RT-R | GGCGAGTAGTTGCAGGTGAT | qRT-PCR for defense-related genes in rice |
| Cht1-RT-F | CGTGGTGACCAACATCATCA | qRT-PCR for defense-related genes in rice |
| Cht1-RT-R | GAGTTGAAAGGCCTCTGGTTGT | qRT-PCR for defense-related genes in rice |
| Mo-ACTIN-F | CCATGTACCCTGGTCTTTCG | qRT-PCR for validation of the RNA-Seq profiling |
| Mo-ACTIN-R | TTCGAGATCCACATCTGCTG | qRT-PCR for validation of the RNA-Seq profiling |
| iug6-U-F | TAA CTCGAG ATTGGCTCAAACGACAGATG | amplify *IUG6* 5’ flank sequence |
| iug6-U-R | TAA GAATTC CTTGTGGATTGGAGGTGTT | amplify *IUG6* 5’ flank sequence |
| iug6-D-F | TAA ACTAGT ATATCCGCACGCGAAAACT | amplify *IUG6* 3’ flank sequence |
| iug6-D-R | TAA GAGCTC GGACCTATGCTGGTTGTC | amplify *IUG6* 3’ flank sequence |
| iug9-U-F | TAA CTCGAG TCGCAAGTCATACAAGCTCC | amplify *IUG9* 5’ flank sequence |
| iug9-U-R | TAA GAATTC CGGAGATCAAAGCTGTAGAG | amplify *IUG9* 5’ flank sequence |
| iug9-D-F | TAA ACTAGT GCTACCCAGTAAATGTGTGG | amplify *IUG9* 3’ flank sequence |
| iug9-D-R | TAA GAGCTC GTCCTGCTTACAGGTTTGTTC | amplify *IUG9* 3’ flank sequence |
| iug17-U-F | TAA CTCGAG GATCGGAGCTCATTTAGAGC | amplify *IUG17* 5’ flank sequence |
| iug17-U-R | TAA CTCGAG GATCGGAGCTCATTTAGAGC | amplify *IUG17* 5’ flank sequence |
| iug17-D-F | TAA ACTAGT TATTTAACTGGAGCCGGGAT | amplify *IUG17* 3’ flank sequence |
| iug17-D-R | TAA CCGCGG ATTTCCAAGCACACTCTGTC | amplify *IUG17* 3’ flank sequence |
| iug18-U-F | TAA CTCGAG TCCTGGTGAACCACGTTATT | amplify *IUG18* 5’ flank sequence |
| iug18-U-R | TAA GAATTC GCCTCCTTTGCCGATCTAAT | amplify *IUG18* 5’ flank sequence |
| iug18-D-F | TAA ACTAGT TTCCATGAGCAGGTACTAAT | amplify *IUG18* 3’ flank sequence |
| iug18-D-R | TAA CCGCGG ATCCGATTGTTTCCAGTTCG | amplify *IUG18* 3’ flank sequence |
| Iug34-U-F | TAA CTCGAG GCAACCTAAGGCTATGGAAT | amplify *IUG34* 3’ flank sequence |
| Iug34-U-R | TAA GAATTC GCTGACGATGTTTTGTACG | amplify *IUG34* 3’ flank sequence |
| Iug34-D-F | TAA GGATCC ATTGGATCAGGGTGTGTTG | amplify *IUG34* 3’ flank sequence |
| Iug34-D-R | TAA ACTAGT TTGTGGTCACCAATCAGCTC | amplify *IUG34* 3’ flank sequence |
| Iug37-U-F | TAA CTCGAG ACTTTCTCCTCGATGGTGAG | amplify *IUG37* 3’ flank sequence |
| Iug37-U-R | TAA GAATTC GCCAAGTCATTTGTTATGGG | amplify *IUG37* 3’ flank sequence |
| Iug37-D-F | TAA ACTAGT AATAATCCAGCTGCAGCCAG | amplify *IUG37* 3’ flank sequence |
| Iug37-D-R | TAA GAGCTC TGTGAGGACGAGTCTAGTTG | amplify *IUG37* 3’ flank sequence |
| iug6-yeast-F | ACTCACTATAGGGCGAATTGGGTACTCAAATTGGTTTTGGCTCAAACGACAGATGC | *IUG6* complementation; *IUG6*:GFP localization |
| iug6-yeast-RP27-F | TTT CGT AGG AAC CCA ATC TTC AAA ATGCAGTTCTCCACGATCCA | *IUG6* overexpression |
| iug6-yeast-R2 | CACCACCCCGGTGAACAGCTCCTCGCCCTTGCTCACGGTGCTGGAGCAGTATTTG | *IUG6* complementation; *IUG6*:GFP localization; *IUG6* overexpression |
| iug9-yeast-F | ACTCACTATAGGGCGAATTGGGTACTCAAATTGGTTGATGGATGACGATATAAGGC | *IUG9* complementation; *IUG9*:GFP localization |
| iug9-yeast-RP27-F | TTT CGT AGG AAC CCA ATC TTC AAAATGAAGTTTATTTACTCTACAGC | *IUG9* overexpression |
| iug9-yeast-R2 | CACCACCCCGGTGAACAGCTCCTCGCCCTTGCTCACCTGGGTAGCTTTCCCACCTT | *IUG9* complementation; *IUG9*:GFP localization; *IUG9* overexpression |
| iug18-yeast-F | ACTCACTATAGGGCGAATTGGGTACTCAAATTGGTTTCCTGGTGAACCACGTTATT | *IUG18* complementation; *IUG18*:GFP localization |
| iug18-yeast-R | CACCACCCCGGTGAACAGCTCCTCGCCCTTGCTCACATATTTAAATAGTCCGGGAAC | *IUG18* complementation; *IUG18*:GFP localization |
| AvrPiz-t-yeast-F | ACTCACTATAGGGCGAATTGGGTACTCAAATTGGTTCGCAGTGATAATCGGAATAC | AvrPiz-t:GFP localization |
| AvrPiz-t -yeast-R2 | CACCACCCCGGTGAACAGCTCCTCGCCCTTGCTCACTTGGCGCTGAGCCTGAGG | AvrPiz-t:GFP localization |
| iug6-R | TTAGGTGCTGGAGCAGTATT | amplify *IUG6* probe sequence |
| iug9-F | ACTCTACAGCTTTGATCTCC | amplify *IUG9* probe sequence |
| iug9-R | AGCTTTCCCACCTTCAGCAG | amplify *IUG9* probe sequence |
| iug17-F | TTCCACTGCTGCCAAAAGCT | amplify *IUG17* probe sequence |
| iug17-R | CCTTGGCTTTCCATGAAAGC | amplify *IUG17* probe sequence |
| iug18-F | GAGTCAGGTTCAGGATGACT | amplify *IUG18* probe sequence |
| iug18-R | TTCGAGAGCGTCCATTAGT | amplify *IUG18* probe sequence |
| Iug34-F | ATCTCGAAGACGCTTCTCGT | amplify *IUG34* probe sequence |
| Iug34-R | GATGATGTCTTCGATCTGGT | amplify *IUG34* probe sequence |
| Iug37-F | CACTTCAAGTCAAGACAC | amplify *IUG37* probe sequence |
| Iug37-R | ATCTCAACCTCGACGACAT | amplify *IUG37* probe sequence |
| iug6-RT-F | TCTCCACGATCCAGCTTTTC | quantitative RT-PCR analysis of *IUG6* |
| iug6-RT-R | TGAGCCACTTGAACCTCTCA | quantitative RT-PCR analysis of *IUG6* |
| iug9-RT-F | AGCTTTGATCTCCGTTCTGG | quantitative RT-PCR analysis of *IUG9* |
| iug9-RT-R | CTTTCGCGCTTCTTCTTTTG | quantitative RT-PCR analysis of *IUG9* |
| iug18-RT-F | TCAACAAGCAGGCAGAGCTA | quantitative RT-PCR analysis of *IUG18* |
| iug18-RT-R | GCCTGTTCAAAGCAGTCTCC | quantitative RT-PCR analysis of *IUG18* |
| iug6-ssp-F | TAAGAATTCAACACCTCCAATCCACAAG | amplify signal peptide of Iug6 |
| iug6-ssp-R | TAACTCGAG GTTGGCCATGGCGCCGAC | amplify signal peptide of Iug6 |
| iug9-ssp-F | TAAGAATTCTCGTTTATTAACAAGAAGCAAA | amplify signal peptide of Iug9 |
| iug9-ssp-R | TAACTCGAG TAAACCGCGGCCGGTGG | amplify signal peptide of Iug9 |
| iug6-sma I -F | GGGATGCAGTTCTCCACGATCC | Clone Iug6 for expression in N. benthamiana |
| iug6-sp-F2 | GGGATGACCGTTGCCCCCGCCGA | Clone Iug6^Δsp^ for expression in N. benthamiana |
| iug6-sma I -R | GGG GGTGCTGGAGCAGTATTTG | Clone Iug6 for expression in N. benthamiana |
| iug9-sma I -F | GGGATGAAGTTTATTTACTCTAC | Clone Iug9 for expression in N. benthamiana |
| iug9-sma I -R | GGG CTGGGTAGCTTTCCCACCTT | Clone Iug9 for expression in N. benthamiana |
| iug9-sp-sma I -F2 | GGGATGACTTCTGGCCTTATGAGAC | Clone Iug9^Δsp^ for expression in N. benthamiana |
| Nup1- Cla I -F | ATCGATATGTACGCCTTCAACTTCT | Clone Nup1 for expression in N. benthamiana |
| Nup1--sp- Cla I -F2 | TAAATCGATATGTCACCAGTCGCAATCG | Clone Nup1^Δsp^ for expression in N. benthamiana |
| Nup1--sal I -R | GTCGACGGCCTTTGGAGGGCAG | Clone Nup1 for expression in N. benthamiana |
| Nup2- Cla I -F | ATCGATATGATCTTCAACGTTTTTAC | Clone Nup2 for expression in N. benthamiana |
| Nup2--sp- Cla I -F2 | ATCGATATGGCCCCGGCCTCCCCCG | Clone Nup2^Δsp^ for expression in N. benthamiana |
| Nup2--sma I -R | GGGCTGCTTGGGAGGAGGG | Clone Nup2 for expression in N. benthamiana |
| Nup3- Cla I -F | ATCGATATGCGAGCCACCACAGCCTT | Clone Nup3 for expression in N. benthamiana |
| Nup3--sp- Cla I -F2 | ATCGATATGGCTCCCACCGGTCTGG | Clone Nup3^Δsp^ for expression in N. benthamiana |
| Nup3--sal I -R | GTCGACCTGCAGCAAGACGTCC | Clone Nup3 for expression in N. benthamiana |
| Nup1-Poly-F | GTACGCCTTCAACTTCTTCG | amplify candidate effectors for presence /absence polymorphism |
| Nup1-Poly-R | CAGGTACGGAACGTTCTTGC | amplify candidate effectors for presence /absence polymorphism |
| Nup2-Poly-F | CGTCATCATGATCTTCAACG | amplify candidate effectors for presence /absence polymorphism |
| Nup2-Poly-R | ATCGCATGGCTTTTACTGCT | amplify candidate effectors for presence /absence polymorphism |
| Nup3-Poly-F | CCACCACAGCCTTTCAGGTT | amplify candidate effectors for presence /absence polymorphism |
| Nup3-Poly-R | AGGCTACTGCAGCAAGACGT | amplify candidate effectors for presence /absence polymorphism |
| E001-Poly-F | ATCCATCATGTCGCCCCTAT | amplify candidate effectors for presence /absence polymorphism |
| E001-Poly-R | GTAGACAAAGTCATGACCG | amplify candidate effectors for presence /absence polymorphism |
| E002-Poly-F | ATTTCTGCGGCCTTGGCTTT | amplify candidate effectors for presence /absence polymorphism |
| E002-Poly-R | GGACTACTGCCACGGATTAT | amplify candidate effectors for presence /absence polymorphism |
| E003-Poly-F | CACCAATATACGAACGTCTACC | amplify candidate effectors for presence /absence polymorphism |
| E003-Poly-R | CTGGCCGTGACAGAAACTAT | amplify candidate effectors for presence /absence polymorphism |
| E004-Poly-F | CGACTTCTCAACATGCAGATC | amplify candidate effectors for presence /absence polymorphism |
| E004-Poly-R | CTCCTTGTTGCTGTCGCTTT | amplify candidate effectors for presence /absence polymorphism |
| E005-Poly-F | CCCTTTTCAATTCCCTCACC | amplify candidate effectors for presence /absence polymorphism |
| E005-Poly-R | ACGTTTCCAACACTTGAGCG | amplify candidate effectors for presence /absence polymorphism |
| E006-Poly-F | CTTTGTTACTCGACTTGCC | amplify candidate effectors for presence /absence polymorphism |
| E006-Poly-R | ACTCTGCCATCTTGAAAGCG | amplify candidate effectors for presence /absence polymorphism |
| E007-Poly-F | CTTGTTGCTCTTTTCGCCCT | amplify candidate effectors for presence /absence polymorphism |
| E007-Poly-R | CTTTTCAAGGGTGTACCACG | amplify candidate effectors for presence /absence polymorphism |
| E008-Poly-F | TATCTCACCTTCGGGCTTGT | amplify candidate effectors for presence /absence polymorphism |
| E008-Poly-R | ATGCATGTAGCCCACTATCC | amplify candidate effectors for presence /absence polymorphism |
| E009-Poly-F | CGCTAGATCGCTTGCAAT | amplify candidate effectors for presence /absence polymorphism |
| E009-Poly-R | CACTTCAGTTAGCACATCC | amplify candidate effectors for presence /absence polymorphism |
| E010-Poly-F | AATCCCAAACCCGCAACCAT | amplify candidate effectors for presence /absence polymorphism |
| E010-Poly-R | CTCAACCGAGAATCTCCTAG | amplify candidate effectors for presence /absence polymorphism |
| E011-Poly-F | CAACCGGCAAAATGCAACTC | amplify candidate effectors for presence /absence polymorphism |
| E011-Poly-R | CTTGTATTATACAGCCCAGCG | amplify candidate effectors for presence /absence polymorphism |
| E012-Poly-F | CCTTTCTGTACGAAATGCGG | amplify candidate effectors for presence /absence polymorphism |
| E012-Poly-R | CGAATATTCTGGAACACAGCG | amplify candidate effectors for presence /absence polymorphism |
| E013-Poly-F | CCAACATGCAGATCAAGG | amplify candidate effectors for presence /absence polymorphism |
| E013-Poly-R | CAGAACGGCTGGTTTAATGG | amplify candidate effectors for presence /absence polymorphism |
| E014-Poly-F: | GGATAGAATGCAGCTCTCC | amplify candidate effectors for presence /absence polymorphism |
| E014-Poly-R | GTTTGTGGGTTTAGTCCCTC | amplify candidate effectors for presence /absence polymorphism |
| E015-Poly-F | ACCATCATCACCTTGGCAGT | amplify candidate effectors for presence /absence polymorphism |
| E015-Poly-R | CTCGCCCTCAACAATCAAAC | amplify candidate effectors for presence /absence polymorphism |
| E016-P0ly-F: | CGCTTCCTTTCCCTCGTTTTCG | amplify candidate effectors for presence /absence polymorphism |
| E016-P0ly-R: | CTCCGCGCATCCAATGAATCCA | amplify candidate effectors for presence /absence polymorphism |
| E017-P0ly-F | AAGTTCTCTGCCGCCCTTTTG | amplify candidate effectors for presence /absence polymorphism |
| E017-P0ly-R | TCAAAGATTGTGTTGCACTCGG | amplify candidate effectors for presence /absence polymorphism |
| E018-P0ly-F | CATATCGTCGCATTCATCACCG | amplify candidate effectors for presence /absence polymorphism |
| E018-P0ly-R | GGTCACCAAGTCGATTCCATC | amplify candidate effectors for presence /absence polymorphism |
| E019-P0ly-F | CAATTGAAATCCGCACTCTGCT | amplify candidate effectors for presence /absence polymorphism |
| E019-P0ly-R | CTGTCCGAGACTTCCGTCATCC | amplify candidate effectors for presence /absence polymorphism |
| E020-P0ly-F | TATACCTGCTTCCCGCCATCT | amplify candidate effectors for presence /absence polymorphism |
| E020-P0ly-R | ACCCGGAGCGTCCAGCCTCTT | amplify candidate effectors for presence /absence polymorphism |
| E021-P0ly-F | CGCTTTCCATCACTCGTCTTCT | amplify candidate effectors for presence /absence polymorphism |
| E021-P0ly-R | CGAATATTCTTCTTTGGGATCC | amplify candidate effectors for presence /absence polymorphism |
| E022-P0ly-F | GTCCGCATCACTATCGCAGCTG | amplify candidate effectors for presence /absence polymorphism |
| E022-P0ly-R | GAAACCCTGGCGCTGGACCT | amplify candidate effectors for presence /absence polymorphism |
| E023-P0ly-F | CACGCTTTCAAATTCGTTCA | amplify candidate effectors for presence /absence polymorphism |
| E023-P0ly-R | TTCTGGCCTCTTCCCTTTGCC | amplify candidate effectors for presence /absence polymorphism |
| E024-P0ly-F | GTTTTCCCTGGCCCTTTTCG | amplify candidate effectors for presence /absence polymorphism |
| E024-P0ly-R | TTAGTGCAAAAGCCGTTGCTAG | amplify candidate effectors for presence /absence polymorphism |
| E026-P0ly-F: | TGAGCAACTATGCACCCAAG | amplify candidate effectors for presence /absence polymorphism |
| E026-P0ly-R: | GATGAAGTAGTCGTGGTACC | amplify candidate effectors for presence /absence polymorphism |
| E027-P0ly-F | GCTGCTACACCAGTAACAATG | amplify candidate effectors for presence /absence polymorphism |
| E027-P0ly-R | GTGTTGACGGTGTTAACAGTC | amplify candidate effectors for presence /absence polymorphism |
| E028-P0ly-F | ATGCAGTTCTCTTTCGCCAC | amplify candidate effectors for presence /absence polymorphism |
| E028-P0ly-R | TTGACTCTGGAGTCCGTCAG | amplify candidate effectors for presence /absence polymorphism |
| E029-P0ly-F | AGATCACCAAGATCCTCCAG | amplify candidate effectors for presence /absence polymorphism |
| E029-P0ly-R | TCTCTGGTCGCCTAGTTTTG | amplify candidate effectors for presence /absence polymorphism |
| E030-P0ly-F | TTGACACCGACCGTTATAGC | amplify candidate effectors for presence /absence polymorphism |
| E030-P0ly-R | GCGAGGATGGATTGTACGAC | amplify candidate effectors for presence /absence polymorphism |
| E031-P0ly-F | TGTACCTCATCAACCTGATC | amplify candidate effectors for presence /absence polymorphism |
| E031-P0ly-R | GGCTGCCATCTCGAATTACT | amplify candidate effectors for presence /absence polymorphism |
| E032-P0ly-F | ATCGAGTGGCAAGCAAGAAT | amplify candidate effectors for presence /absence polymorphism |
| E032-P0ly-R | GTACCCCGGACAATTTGTTG | amplify candidate effectors for presence /absence polymorphism |
| E034-P0ly-F | AACATTGTTCAGGTCCTCGG | amplify candidate effectors for presence /absence polymorphism |
| E034-P0ly-R | CCTCGCTGTAATTCGACTC | amplify candidate effectors for presence /absence polymorphism |
| E035-P0ly-F | CAACCTTGGTTGCAGCAACC | amplify candidate effectors for presence /absence polymorphism |
| E035-P0ly-R | AGCTAGCTGACCTTGCCTTC | amplify candidate effectors for presence /absence polymorphism |
| E036-P0ly-F | TTGGAGTCGCTGTCATCTTG | amplify candidate effectors for presence /absence polymorphism |
| E036-P0ly-R | TATAGGGTCTGACCACCTTC | amplify candidate effectors for presence /absence polymorphism |
| E037-P0ly-F | ATGCAGTTCTCCATCTACGC | amplify candidate effectors for presence /absence polymorphism |
| E037-P0ly-R | GTGCTGGCAGTAAACTTCAG | amplify candidate effectors for presence /absence polymorphism |
| E038-P0ly-F | CGCTTCTCCACCATCTTCAT | amplify candidate effectors for presence /absence polymorphism |
| E038-P0ly-R | TAGACCAGGAGCATGGCAAC | amplify candidate effectors for presence /absence polymorphism |
| E039-P0ly-F | TCAATTGCCTGCACCATTGC | amplify candidate effectors for presence /absence polymorphism |
| E039-P0ly-R | GGTCTAAGGATGGACCGATT | amplify candidate effectors for presence /absence polymorphism |
| E040-P0ly-F | CACTTCTCCAAGATCTCCGT | amplify candidate effectors for presence /absence polymorphism |
| E040-P0ly-R | TTAATTGCCAGAGACCAGGC | amplify candidate effectors for presence /absence polymorphism |
| E041-P0ly-F | GCTTGCCTACAACGTCATTG | amplify candidate effectors for presence /absence polymorphism |
| E041-P0ly-R | GGCACTGCTTGCCGTAAAAG | amplify candidate effectors for presence /absence polymorphism |
| E042-P0ly-F | ATGAGAGTCAGCACCGCCTT | amplify candidate effectors for presence /absence polymorphism |
| E042-P0ly-R | ATCCACGCACATGATCTTCG | amplify candidate effectors for presence /absence polymorphism |
| E043-P0ly-F | AAAATGCGCATCACCAGCC | amplify candidate effectors for presence /absence polymorphism |
| E043-P0ly-R | GTCCTTCTTCTGGGTTTCCT | amplify candidate effectors for presence /absence polymorphism |
| E044-P0ly-F: | GCACGTTAAGCAATCGACTT | amplify candidate effectors for presence /absence polymorphism |
| E044-P0ly-R | CAACGTTCCCACAGTATACC | amplify candidate effectors for presence /absence polymorphism |
| E045-P0ly-F | CACCATGAAGATCAACAACG | amplify candidate effectors for presence /absence polymorphism |
| E045-P0ly-R | CAATGACCTAGACGGTACCG | amplify candidate effectors for presence /absence polymorphism |
| E046-P0ly-F | GCCAAAATGCAGCTTTCAAAC | amplify candidate effectors for presence /absence polymorphism |
| E046-P0ly-R | AGGTTCTTCAGAGCTGGACC | amplify candidate effectors for presence /absence polymorphism |
| E047-P0ly-F | CCATGATGCAAGTTCTCAAG | amplify candidate effectors for presence /absence polymorphism |
| E047-P0ly-R | CATGCCTATGATCTTCGACC | amplify candidate effectors for presence /absence polymorphism |
| E048-P0ly-F | TGCGTCCCCAATTCATCATC | amplify candidate effectors for presence /absence polymorphism |
| E048-P0ly-R | TACCGACGCTCTAATTCCTG | amplify candidate effectors for presence /absence polymorphism |
| E049-P0ly-F | CACTTATTGCTGCAGTCCTG | amplify candidate effectors for presence /absence polymorphism |
| E049-P0ly-R | GTCTCGATTTCAGGGGATC | amplify candidate effectors for presence /absence polymorphism |
| E050-P0ly-F: | GAGCAAACTTTTCCTGTCCG | amplify candidate effectors for presence /absence polymorphism |
| E050-P0ly-R | TTCTCCATGCCTTGTTCTCG | amplify candidate effectors for presence /absence polymorphism |
| E051-P0ly-F: | TTATCATTGCCGTGCTGGCT | amplify candidate effectors for presence /absence polymorphism |
| E051-P0ly-R | CTTGCCACCTCGACTTCTAC | amplify candidate effectors for presence /absence polymorphism |
| E052-P0ly-F: | CAGAGCATCAACGATGTTCC | amplify candidate effectors for presence /absence polymorphism |
| E052-Poly-R | AATTTACAGAGCGAGGGCAC | amplify candidate effectors for presence /absence polymorphism |
| E053-Poly-F: | TTCAATCCTCCACATTGCC | amplify candidate effectors for presence /absence polymorphism |
| E053-Poly-R | GCACTTCTTCTCGCAGTAGT | amplify candidate effectors for presence /absence polymorphism |
| E054-Poly-F: | GATGCGTTTCCAAACCATCC | amplify candidate effectors for presence /absence polymorphism |
| E054-Poly-R | AAAGCAACGCAGAGGTTCGT | amplify candidate effectors for presence /absence polymorphism |
| E055-Poly-F: | CGATTCTCGACCGTTTTCCT | amplify candidate effectors for presence /absence polymorphism |
| E055-Poly-R | TCCGACCGAAACGACCAAGT | amplify candidate effectors for presence /absence polymorphism |
| E056-Poly-F: | AGTTTGTCATCTCCTGCCTC | amplify candidate effectors for presence /absence polymorphism |
| E056-Poly-R | CCATGTTTGCACTCGCAATG | amplify candidate effectors for presence /absence polymorphism |
| E057-Poly-F: | AGGATCTTACCTGACGGTC | amplify candidate effectors for presence /absence polymorphism |
| E057-Poly-R | TGAGCCTTCAACTGCTCGAG | amplify candidate effectors for presence /absence polymorphism |
| E058-Poly-F: | CGTTTCCAAGCGGTTGTCAT | amplify candidate effectors for presence /absence polymorphism |
| E058-Poly-R | ACGACCATAGGCAGACTCTT | amplify candidate effectors for presence /absence polymorphism |
| E059-Poly-F: | GCAGCTTCAGTCAATCATCG | amplify candidate effectors for presence /absence polymorphism |
| E059-Poly-R | AACTCCAGCACAGTTTGCGT | amplify candidate effectors for presence /absence polymorphism |
| E060-Poly-F: | GTCCCTACTACCTCTGTATC | amplify candidate effectors for presence /absence polymorphism |
| E060-Poly-R | TTGCAGCATCCGTCAAGATC | amplify candidate effectors for presence /absence polymorphism |
| E061-Poly-F: | AAGATGCAGTTCCGTCAGAC | amplify candidate effectors for presence /absence polymorphism |
| E061-Poly-R | ATCTCTGGGATAGCAGAGTG | amplify candidate effectors for presence /absence polymorphism |
| E062-Poly-F: | ATGGTCTCCTTCACCCACGT | amplify candidate effectors for presence /absence polymorphism |
| E062-Poly-R | CAAAGGAGCGTCCACTTG | amplify candidate effectors for presence /absence polymorphism |
| E063-Poly-F: | AGAACATCTTCGTCCTCGCT | amplify candidate effectors for presence /absence polymorphism |
| E063-Poly-R | CGCAGTTAACATTGCCCTTG | amplify candidate effectors for presence /absence polymorphism |
| E064-Poly-F: | GCCAAAATGCAGATCAAGAC | amplify candidate effectors for presence /absence polymorphism |
| E064-Poly-R | TAGTAGGTGCAAGTGCACTC | amplify candidate effectors for presence /absence polymorphism |
| E065-Poly-F: | CTCAGATGGAGGATACTCAG | amplify candidate effectors for presence /absence polymorphism |
| E065-Poly-R | CCATGAAAACACTCCTCCTC | amplify candidate effectors for presence /absence polymorphism |
| E066-Poly-F: | TTGCTGGCAAGCTCTACTGT | amplify candidate effectors for presence /absence polymorphism |
| E066-Poly-R | ATCATCGACCTTATCGGAGC | amplify candidate effectors for presence /absence polymorphism |
| E067-Poly-F: | ATGCAGTTCAGCAGCGTCTT | amplify candidate effectors for presence /absence polymorphism |
| E067-Poly-R | CACCGTGCACTTCTCAACTT | amplify candidate effectors for presence /absence polymorphism |
| E068-Poly-F: | GTCTCCCAATCGTACCTCCT | amplify candidate effectors for presence /absence polymorphism |
| E068-Poly-R | CAGCAGCACGACCAGAACTT | amplify candidate effectors for presence /absence polymorphism |
| E069-Poly-F: | CCATGCAGCTCTCTTCAG | amplify candidate effectors for presence /absence polymorphism |
| E069-Poly-R | CTGAACATGACTCACGCAAC | amplify candidate effectors for presence /absence polymorphism |
| E070-Poly-F: | ATGCAGATCTCCCACATTGC | amplify candidate effectors for presence /absence polymorphism |
| E070-Poly-R | TCCTACTTCTGCTCCTGGTT | amplify candidate effectors for presence /absence polymorphism |
| E071-Poly-F: | CATCCCTCGTCGTTTCTCAT | amplify candidate effectors for presence /absence polymorphism |
| E071-Poly-R | ATGCGGTAAACGTCCTTGTC | amplify candidate effectors for presence /absence polymorphism |
| E072-Poly-F: | CAGCATCTTGATGCAAATGGC | amplify candidate effectors for presence /absence polymorphism |
| E072-Poly-R | TGCAGAGTTTATCCAGGCAG | amplify candidate effectors for presence /absence polymorphism |
| E073-Poly-F: | ATGCCTAGCTTCAGCAAGAC | amplify candidate effectors for presence /absence polymorphism |
| E073-Poly-R | TTAGTGAGCCGCACCATTAG | amplify candidate effectors for presence /absence polymorphism |
| E074-Poly-F: | GCACTCTTCTCTCATTCTCC | amplify candidate effectors for presence /absence polymorphism |
| E074-Poly-R | CTCCTCAGGAGTCAAAATTCC | amplify candidate effectors for presence /absence polymorphism |
| E076-Poly-F: | TTTTCATTCCTGCTGCCCTG | amplify candidate effectors for presence /absence polymorphism |
| E076-Poly-R | CGATCCCTCAATATGACCC | amplify candidate effectors for presence /absence polymorphism |
| E077-Poly-F: | GCAGTTCAAGACGATATTCAC | amplify candidate effectors for presence /absence polymorphism |
| E077-Poly-R | ACGCCTCCACTCATTGAATG | amplify candidate effectors for presence /absence polymorphism |
| E078-Poly-F | GATGCATTACCACAGCCTGT | amplify candidate effectors for presence /absence polymorphism |
| E078-Poly-R | CGCACTCATGCAACAACCTT | amplify candidate effectors for presence /absence polymorphism |
| E079-Poly-F | ATCATGATCGGCCTCAAGTC | amplify candidate effectors for presence /absence polymorphism |
| E079-Poly-R | ATGCCTCGTCACATAGCAAC | amplify candidate effectors for presence /absence polymorphism |
| E080-Poly-F | CTCTCAAAACCCTCCTGCTT | amplify candidate effectors for presence /absence polymorphism |
| E080-Poly-R | CCTAAGAACAACACGCTGGT | amplify candidate effectors for presence /absence polymorphism |
| E081-Poly-F | ATAAAATGCGGTCCCAAGCC | amplify candidate effectors for presence /absence polymorphism |
| E081-Poly-R | AACCTGGTCCCAATACTCAG | amplify candidate effectors for presence /absence polymorphism |
| E082-Poly-F | ACGCCCAGCAGTACATCATT | amplify candidate effectors for presence /absence polymorphism |
| E082-Poly-R | AACAGCGCCTACTTGCACTT | amplify candidate effectors for presence /absence polymorphism |
| E083-Poly-F | CCTCACAACCATGCAGTTTTC | amplify candidate effectors for presence /absence polymorphism |
| E083-Poly-R | TACAGCTTCCTTCCATCGCT | amplify candidate effectors for presence /absence polymorphism |
| E084-Poly-F | ATGCAGTTCTCCACCATCCT | amplify candidate effectors for presence /absence polymorphism |
| E084-Poly-R | CAAGCTCTCCTCATCTAAGG | amplify candidate effectors for presence /absence polymorphism |
| E085-Poly-F | CACTCCTTTTCTCTCTCCTC | amplify candidate effectors for presence /absence polymorphism |
| E085-Poly-R | TTCAGCAGGACAAGCGCAAT | amplify candidate effectors for presence /absence polymorphism |
| E086-Poly-F | GTTGGTCTTACCTATCACCG | amplify candidate effectors for presence /absence polymorphism |
| E086-Poly-R | GTCTCCAAACCCACAGTTAG | amplify candidate effectors for presence /absence polymorphism |
| E087-Poly-F | ATGCAGTTCTCTACCATCGC | amplify candidate effectors for presence /absence polymorphism |
| E087-Poly-R | TCTACTCTTCGGAATCCCTG | amplify candidate effectors for presence /absence polymorphism |
| E088-Poly-F | AAATGCAGATCTCCGTCTCC | amplify candidate effectors for presence /absence polymorphism |
| E088-Poly-R | GGCTTGAAACCACTCAGTTG | amplify candidate effectors for presence /absence polymorphism |
| E089-Poly-F | GATAAGCCATGCTGCCTTTG | amplify candidate effectors for presence /absence polymorphism |
| E089-Poly-R | CCAGTCTAACTGCAGATAGG | amplify candidate effectors for presence /absence polymorphism |
| E090-Poly-F | TTTGCTGTTCGCTCCTCCT | amplify candidate effectors for presence /absence polymorphism |
| E090-Poly-R | TGCACAATTCGTCTCCATGC | amplify candidate effectors for presence /absence polymorphism |
| E091-Poly-F | GCAACTCATCAACATCCTCC | amplify candidate effectors for presence /absence polymorphism |
| E091-Poly-R | CCCTCAATGCAACAATGCTTG | amplify candidate effectors for presence /absence polymorphism |
| E092-Poly-F | CGCACAACAACACGGAAAC | amplify candidate effectors for presence /absence polymorphism |
| E092-Poly-R | TTCTACAGCAAGACAACGG | amplify candidate effectors for presence /absence polymorphism |
| E093-Poly-F | ATGCGAACCCAAGCCTTCTT | amplify candidate effectors for presence /absence polymorphism |
| E093-Poly-R | GGTGGTGCAATAGTAGTTGC | amplify candidate effectors for presence /absence polymorphism |
| E094-Poly-F | CCCTTGTGGCCTTTTCACTT | amplify candidate effectors for presence /absence polymorphism |
| E094-Poly-R | CCTCATAAGCGAAGCAGAAG | amplify candidate effectors for presence /absence polymorphism |
| E095-Poly-F | AACACCAACCGCAAACGATC | amplify candidate effectors for presence /absence polymorphism |
| E095-Poly-R | CTCGACACACTTAGTCCATG | amplify candidate effectors for presence /absence polymorphism |
| E096-Poly-F | TGCAGTACTCTTTCGTCACC | amplify candidate effectors for presence /absence polymorphism |
| E096-Poly-R | CGGTTGTTGAGTCCATTCAC | amplify candidate effectors for presence /absence polymorphism |
| E097-Poly-F | CTCAAGATGCGCGCTTCATT | amplify candidate effectors for presence /absence polymorphism |
| E097-Poly-R | GTAGGATTGCCTGCTTAGCT | amplify candidate effectors for presence /absence polymorphism |
| E098-Poly-F | GTCAACATGAAGGTCATCGC | amplify candidate effectors for presence /absence polymorphism |
| E098-Poly-R | GCGTCGTCGAAGACATTTAC | amplify candidate effectors for presence /absence polymorphism |
| E099-Poly-F | CCACCATTCAAAATGTATTCG | amplify candidate effectors for presence /absence polymorphism |
| E099-Poly-R | GAATAGATTTCTGCGTCGCT | amplify candidate effectors for presence /absence polymorphism |
| E100-Poly-F | CCTTGCAACCACAACAAC | amplify candidate effectors for presence /absence polymorphism |
| E100-Poly-R | CAACCGCCAAAGCCAAATGT | amplify candidate effectors for presence /absence polymorphism |
| E101-Poly-F | GCACTTCTCAAAGCTTCTAG | amplify candidate effectors for presence /absence polymorphism |
| E101-Poly-R | TCATATACTTCCACTTCCAC | amplify candidate effectors for presence /absence polymorphism |
| E103-Poly-F | ATGCGTTTCTCAACCATTGC | amplify candidate effectors for presence /absence polymorphism |
| E103-Poly-R | TCTCGGTCACTTTAACGCTT | amplify candidate effectors for presence /absence polymorphism |
| E104-Poly-F | TGTACAAGACACGCGTTCAC | amplify candidate effectors for presence /absence polymorphism |
| E104-Poly-R | AAATCAACGTGGAGCTGGAT | amplify candidate effectors for presence /absence polymorphism |
| E105-Poly-F | CAGTTCTCTCAGATCCTCAC | amplify candidate effectors for presence /absence polymorphism |
| E105-Poly-R | TTCCTCTACCAGTGCGTCTT | amplify candidate effectors for presence /absence polymorphism |
| E106-Poly-F | TCAACATGCAGCTCAAGACC | amplify candidate effectors for presence /absence polymorphism |
| E106-Poly-R | AACAACAAGAGCAACGGCAC | amplify candidate effectors for presence /absence polymorphism |
| E107-Poly-F | CGTTTCCAGACTGCCATTCT | amplify candidate effectors for presence /absence polymorphism |
| E107-Poly-R | ACCTGTCGGGTTTGGACCTT | amplify candidate effectors for presence /absence polymorphism |
| E108-Poly-F | AGATGCAGATCTTTGCCACC | amplify candidate effectors for presence /absence polymorphism |
| E108-Poly-R | GTTAATCTAGCACCTCCACG | amplify candidate effectors for presence /absence polymorphism |
| E109-Poly-F | CGTTTCGAGACCACAGTCAT | amplify candidate effectors for presence /absence polymorphism |
| E109-Poly-R | GTCCATCATTCCAAAGACCT | amplify candidate effectors for presence /absence polymorphism |
| E110-Poly-F | AAATGCACTTCACCACCGTC | amplify candidate effectors for presence /absence polymorphism |
| E110-Poly-R | GTTTCTGCAGATCAAGCCTG | amplify candidate effectors for presence /absence polymorphism |
| E111-Poly-F | AAGCTATCATCATGGCCTCC | amplify candidate effectors for presence /absence polymorphism |
| E111-Poly-R | TAAGAGCGGTAGACGACCAG | amplify candidate effectors for presence /absence polymorphism |
| E112-Poly-F | TCCACTCGCACACTACAATG | amplify candidate effectors for presence /absence polymorphism |
| E112-Poly-R | CAGCGAGCTAGTCCAGCTTT | amplify candidate effectors for presence /absence polymorphism |
| E113-Poly-F | AAGTTCTCCATCGCCTCCAC | amplify candidate effectors for presence /absence polymorphism |
| E113-Poly-R | CGAGAAGCGTTTAGTCTTGC | amplify candidate effectors for presence /absence polymorphism |
| E114-Poly-F | TCTCTCCAGACAACTACCGT | amplify candidate effectors for presence /absence polymorphism |
| E114-Poly-R | AGCACATCCAACAAATCCATC | amplify candidate effectors for presence /absence polymorphism |
| E115-Poly-F | CTGTGGCATGTTTGGATTGG | amplify candidate effectors for presence /absence polymorphism |
| E115-Poly-R | TCAGTTGTCGAACCACTCG | amplify candidate effectors for presence /absence polymorphism |
| E116-Poly-F | AAATGCGCACCACTTCGATC | amplify candidate effectors for presence /absence polymorphism |
| E116-Poly-R | TAACTGCTTCCTCAGTCGTC | amplify candidate effectors for presence /absence polymorphism |
| E117-Poly-F | CACCAAACTCGTTTGCAGCT | amplify candidate effectors for presence /absence polymorphism |
| E117-Poly-R | ACCTTGATCGTAACACCGAG | amplify candidate effectors for presence /absence polymorphism |
| E118-Poly-F | ATGCAGGTCGCTACCATCCT | amplify candidate effectors for presence /absence polymorphism |
| E118-Poly-R | CTAGCAGGTGTTGAGCATGT | amplify candidate effectors for presence /absence polymorphism |
| E119-Poly-F | TGAAGAGCTTCTTCGTCCTC | amplify candidate effectors for presence /absence polymorphism |
| E119-Poly-R | TACAAGACAGCAACGACGAC | amplify candidate effectors for presence /absence polymorphism |
| E120-Poly-F | AATGCACGTCAAGACCACCT | amplify candidate effectors for presence /absence polymorphism |
| E120-Poly-R | TTACTCCTTTCCCTCGTTGC | amplify candidate effectors for presence /absence polymorphism |
| E121-Poly-F | TCGCAAGCTCACTCGACTTT | amplify candidate effectors for presence /absence polymorphism |
| E121-Poly-R | TGTTAAGAGCCTGGACAGTC | amplify candidate effectors for presence /absence polymorphism |
| E122-Poly-F | AACATGAAGTTCTCAGCCAC | amplify candidate effectors for presence /absence polymorphism |
| E122-Poly-R | GCCATCAAATGTCATTCTCC | amplify candidate effectors for presence /absence polymorphism |
| E123-Poly-F | AGCTCGTCCTCAAACTTTCC | amplify candidate effectors for presence /absence polymorphism |
| E123-Poly-R | TAGTGAGGTCTGAAGTCGTG | amplify candidate effectors for presence /absence polymorphism |
| E124-Poly-F | TCAGTTGAACCACCACCCAC | amplify candidate effectors for presence /absence polymorphism |
| E124-Poly-R | TCAAGACCTCGTCTGCTGCT | amplify candidate effectors for presence /absence polymorphism |
| E125-Poly-F | GAGCTTGATTAGCTTCGACT | amplify candidate effectors for presence /absence polymorphism |
| E125-Poly-R | TCCCTGGACAAAGTCAACAC | amplify candidate effectors for presence /absence polymorphism |
| E126-Poly-F | GCAATACCAGATTCTGAACG | amplify candidate effectors for presence /absence polymorphism |
| E126-Poly-R | TAGCTGAGCTTGGTCTTCTG | amplify candidate effectors for presence /absence polymorphism |
|  |  |  |
|  |  |  |
